# Disentangling First and Second Sphere Effects in Iron–Sulfur Cubanes

**DOI:** 10.1021/jacsau.5c01171

**Published:** 2025-12-18

**Authors:** Liam Grunwald, Katja-Sophia Csizi, Daniel Klose, Vladimir Pelmenschikov, Martin Clémancey, Hongxin Wang, Micha L. Weber, Henrik Seng, Yoshitaka Yoda, Daniel F. Abbott, Patrick Dubourdeaux, Stephen P. Cramer, Markus Reiher, Geneviève Blondin, Victor Mougel

**Affiliations:** † Department of Chemistry and Applied Biosciences (D-CHAB), Swiss Federal Institute of Technology Zürich (ETHZ), Zürich CH-8093, Switzerland; ‡ Institute of Chemistry, 26524 Technical University of Berlin (TUB), Berlin DE-10623, Germany; § CNRS, Commissariat a l’energie Atomique et Aux Energies Alternatives (CEA), Institut de Recherche Interdisciplinaire de Grenoble (IRIG), Laboratoire de Chimie et Biologie des Metaux, Physicochimie des Metaux en Biologie (PMB), CEA Grenoble, University of Grenoble Alpes, Grenoble Cedex F-38054, France; ∥ 165047 SETI Institute, Mountain View, California 94043, United States; ⊥ Precision Spectroscopy Division, SPring-8/JASRI, Sayo 679-5198, Japan

**Keywords:** iron–sulfur cubanes, spectroscopy, electric fields, electronic structure, electron transfer

## Abstract

Cubane-type iron–sulfur clusters (Fe_4_S_4_) are some of the most versatile metallocofactors and, as such, among multiple functions, primarily responsible for mediating challenging electron transfers (ETs). Their efficient ET chemistry is enabled by a conflated interplay of cofactor–protein interactions, which can be categorized into the covalent first (1°) sphere ones and the noncovalent second (2°) sphere ones. The latter have remained particularly elusive, as they are difficult to observe and assess directly and independently. Accordingly, our understanding of these effects is hampered by their entangled nature. To address this, we herein leverage a systematic series of synthetic Fe_4_S_4_ complexes, which allows spectroscopically investigating 2° sphere electrostatic interactions and covalent 1° sphere interactions separately from one another. We expand the study of 1° sphere interactions with a histidine-type ligand in [Fe_4_S_4_]^1+^ complexes to the [Fe_4_S_4_]^2+^ and [Fe_4_S_4_]^3+^ oxidation states, supporting the notion that 1° sphere interactions “fine-tune” the electronic/magnetic structure of these systems in a manner that persists at ambient temperatures. In contrast, scrutinizing the 2° sphere electric dipolar interactions in [Fe_4_S_4_]^1+,2+,3+^ complexes revealed that although similar effects are observable at extremely low temperatures, no significant alteration of the clusters’ gross electronic/magnetic structure persists at the temperatures relevant to enzyme function. These results thus not only systematically catalogue the influence of 1° sphere covalent and 2° sphere electrostatic interactions on the observables and properties of Fe_4_S_4_ complexes, but also establish a clear energetic distinction between the two. As such, they will facilitate identifying the elusive 2° sphere interactions in biological systems, while also strengthening our biophysical understanding of structure–function relationships in Fe_4_S_4_ cofactors.

## Graphical abstract

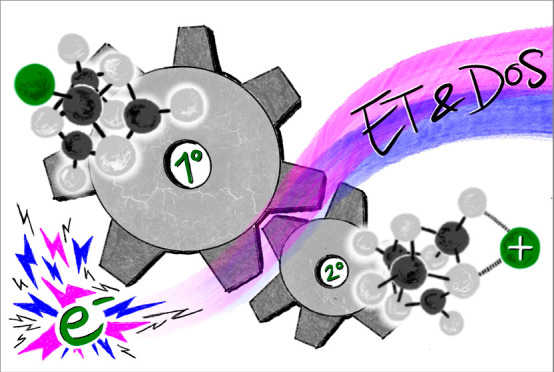

## 1 Introduction

Electron transfer (ET) forms the basis of many fundamental metabolic processes in biology and elementary steps in chemistry.^1,2^ However, despite its apparent simplicity, achieving controlled and selective ETs constitutes a significant challenge for ET proteins.^3,4^ Accordingly, biological systems have evolutionarily developed specific cofactors to facilitate redox processes. Among the most widespread are the “canonical” cubane-type iron–sulfur clusters (Fe_4_S_4_(^Cys^S)_4_), which mediate ET^5,6^ within two major classes of proteins: (i) high-potential iron–sulfur proteins (HiPIPs), which promote oxidative processes, and (ii) ferredoxins (Fds), which facilitate reductive processes.^7,8^ Beyond these systems, FeS cubanes (Fe_4_S_4_) are also often encountered arranged in chains in the protein scaffold of large metabolic enzymes, where they are an integral part of the tertiary structure and enable rapid *intra*-protein ET. Examples for this include respiratory complexes I and II, photosystem I or hydrogenases.^9–14^ Though ET is their main function, Fe_4_S_4_ clusters can adopt other roles.^15^ However, this is also owed to their uniquely efficient and highly adaptive redox chemistry:^7,8,15^ they can theoretically access up to 5 redox levels while maintaining individual Fe atoms in the high spin Fe^2+^/Fe^3+^ (3d^6/5^) valence range, thereby enabling reversible electron transfers with minimal reorganization energy.^16^

The remarkable versatility of biogenic FeS cubane clusters does not come at the expense of selectivity thanks to a complex array of cofactor–protein interactions, which adapt the cluster’s properties to its function. These interactions can be broadly classified into so-called first (1°) sphere and second (2°) sphere effects: the former typically involve covalent ligation of the cluster, while the latter encompass all noncovalent interactions from the surrounding protein matrix,^17^ including for example hydrogen bonding, and local electrostatic environments, as summarized in Figure 1B. Figure 1A provides two concrete examples of how such interactions are essential in modulating Fe_4_S_4_ cofactors’ properties: (i) in [FeFe] hydrogenase, the distal Fe_4_S_4_ cluster presents an unusual “non-canonical” ligand set, in which 1 of 4 cysteinates (Cys) is replaced by a histidine (His). This (covalent) effect in the 1° sphere of the cluster is crucial for maintaining efficient *intra*-protein ET and turnover.^18^ (ii) In CoA dehydratase, the Fe_4_S_4_ cofactor is located directly at the N-terminus of two α-helices, and accordingly experiences a strong local electrostatic effect.^19^ This 2° sphere interaction dynamically modulates its redox potential and enables the generation of high reducing power “on demand” upon ATP hydrolysis.^20–22^ However, despite significant progress regarding the understanding of Fe_4_S_4_ clusters’ functions in enzymatic systems, disentangling the respective contributions of 1° vs 2° sphere interactions remains an open challenge: it relies on being able to translate spectroscopic observables, which reflect the energy landscape of the cluster, into individual contributions of the 1° and 2° sphere perturbations. The latter are often intertwined in metalloenzymes, where particularly noncovalent (2° sphere) effects occur concomitant with changes in the (1°) coordination sphere of the metal center (Figure 1B), complicating the elucidation of structure–function relationships. While 1° sphere covalent interactions, i.e. ligand substitutions of the Fe-atoms, are typically straightforward to identify upon structural determination, 2° sphere effects are much more elusive, because they are generally not directly observable.^23^ This is owed to the fact that among all 2° sphere interactions, electrostatics are a pervasive component of all their types: besides “pure” dipolar effects, electric fields contribute significantly in the interaction of H-bonding networks with the cofactor,^24,25^ and may also describe part of its interaction with charged residues, for example arginine.^26^ In fact, electric fields are important determinants for the selectivity, efficiency and directionality of many fundamental metabolic processes, even beyond ET.^24,27–38^

**Figure 1:**
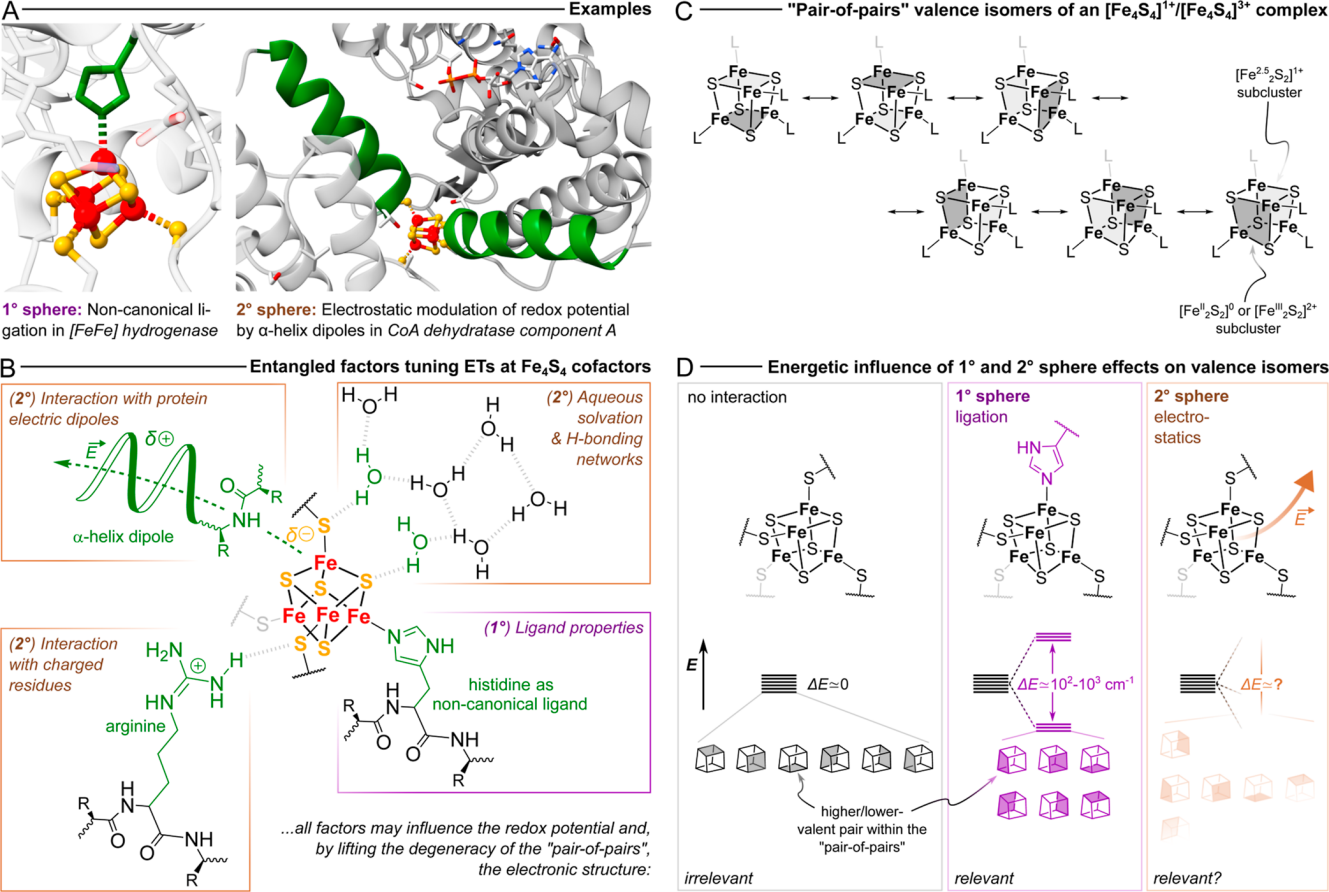
(A) Examples of first (1°) and second (2°) sphere mechanisms for the tuning of redox potential and the density of states in Fe_4_S_4_ cofactor containing enzymes: (*right*) electrostatic tuning by arrangement of the cofactor in the vicinity of α-helix (highlighted in *green*) dipoles in *Acidaminococcus fermentans* (*R*)-2-hydroxyglutaryl-CoA dehydratase component A (PDB: 1HUX);^19^ (*left*) substitution of a cysteine residue by histidine (highlighted in *green*) in [FeFe] hydrogenase complex I from *Clostridium pasteurianum* (PDB: 6GLY).^39^ Images were created using the ChimeraX program suite.^40^ (B) Summary of the most important determinants of ET at Fe_4_S_4_ cofactors established in the literature. The whole of these factors not only tailors the redox potential, but also the electronic structure and, accordingly, the excited state manifold and the composition of the RAMOs. 1° sphere ones are highlighted in *purple* boxes; 2° sphere ones in *brown* ones. (C) Scheme depicting the “pair-of-pairs” valence isomers of odd-electron [Fe_4_S_4_]^1+/3+^ complexes, whereby the two “pairs” of inequivalent valence are represented as differently *shaded planes*. (D) Schematic summary of how different interactions affect valence isomerism in (odd-electron) Fe_4_S_4_ cofactors: no interaction (*left*), ligation of the cofactor in the 1° sphere (*middle*) and 2° sphere electrostatics (*right*).

Here, the study of Fe_4_S_4_-containing enzymes, complemented by that of synthetically tunable Fe_4_S_4_ models, may offer a decisive advantage for elaborating structure–function relationships: they have been characterized by a wealth of spectroscopic techniques across all their redox levels^16^ and their electronic structure and the nature of their observables is well understood.^41–47^ More specifically, the magnetic and electronic properties of Fe_4_S_4_ complexes are related to the nature of their electronic delocalization, which results from the spin-dependent coupling schemes between the clusters’ 4 Fe ions. Though these are complex, they can be simplified by symmetry considerations.^48,49^ The most frequently occurring odd-electron oxidation states in biology are [Fe_4_S_4_]^1+^ and [Fe_4_S_4_]^3+^, each representing the “active” form of the cofactors of Fds and HiPIPs, respectively. In both, the electronic/magnetic structure adheres to the so-called “pair-of-pairs” architecture,^44,50–52^ within which high-spin [Fe_2_S_2_]^0^ or [Fe_2_S_2_]^2+^ rhombs with *S* = 8/2 or *S* = 10/2 total spin, respectively, are antiferromagnetically coupled to a class III^53^ mixed-valent *S* = 9/2 [Fe_2_S_2_]^1+^ cluster, yielding—despite certain exceptions^46,54–56^ —in most cases [Fe_4_S_4_]^1+^ and [Fe_4_S_4_]^3+^ cubanes with *S* = 1/2 total spin projections.^41^ Due to the valence-inequivalence of their two Fe_2_S_2_ subclusters, the odd-electron cubanes are associated with 6 distinct valence isomeric forms (Figure 1C). Accordingly, within the “pair-of-pairs” formalism, there are only 3 different arrangements of this type for the diamagnetic even-electron [Fe_4_S_4_]^2+^ complexes, because the two antiferromagnetically coupled [Fe_2_S_2_]^1+^ subclusters are equivalent. While these 3 arrangements are hence not valence isomers (class III mixed valences renders all Fe sites equal),^45^ they may be considered topological “spin isomers” of each other. In a perfectly symmetric cubane, all valence/spin isomers should have the same energy (Figure 1C,D). However, even small environmental effects, such as that imposed by a crystal lattice, can lead to trapping of energetically favored arrangements of the “pair-of-pairs”. This has been observed earlier on, for example as a “tetragonal compression” of the two coupled [Fe_2_S_2_]^1+/0^ rhombs of the [Fe_4_S_4_]^2+/1+^ cores in the seminal structural studies of [Fe_4_S_4_(BzS)_4_]^2–^ and [Fe_4_S_4_(^*t*^BuS/EtS)_4_]^3–^ by single-crystal X-ray diffraction.^57,58^

Recently, Suess and co-workers investigated the influence of 1° sphere interactions on ET chemistry, and on valence isomerism in 3:1 site substituted [Fe_4_S_4_]^1+^ clusters bearing carbene ligands^59,60^ within this framework.^48,49^ They concluded that the lowering of symmetry causes significant alterations to the size, localization and shape of the redox active molecular orbitals (RAMOs) by lifting the degeneracy of the Fe_4_S_4_ core’s valence isomers up to magnitudes of 10^2^ to 10^3^ cm^–1^ (Figure 1D, *middle*) and thereby substantially altering its density of states.

In contrast, the experimental rationalization of the influence of 2° sphere interactions remained more elusive: even though several theoretical and spectroscopic works on enzymatic active sites suggested that electric fields can alter the valence state ordering of Fe_4_S_4_(^Cys^S)_4_ cofactors,^61–66^ direct comparisons between 1° and 2° sphere effects have been hindered by the lack of synthetic systems in which electrostatics can be disentangled from other interactions.^67^ As such, our biophysical understanding of Fe_4_S_4_ cofactors’ functions would benefit significantly from distinguishing the extents to which the two spheres affect the clusters’ observables and how they accordingly influence the energetic structure of the Fe_4_S_4_ core on a more fundamental (i.e., quantum mechanical) level. While covalent 1° sphere interactions cause relatively large perturbations to the clusters’ geometric and electronic structure,^48,49^ we anticipate that 2° sphere electrostatic interactions induce much smaller perturbations and remain untraceable without well-defined molecular model systems in which only 2° sphere parameters are modified.

To experimentally assess the effect of electric fields on the ET chemistry of Fe_4_S_4_ cubanes, we recently prepared a complete series of [Fe_4_S_4_(RS)_4_]^*x*−^ (*x* = 0–4) model complexes with the ability to reversibly bind and release alkali-metal cations.^16^ This series is unique in the sense that, while encompassing all possible oxidation states of the Fe_4_S_4_ core, it allows us to experimentally disentangle the effect of an electric field acting on the cluster from all other factors,^22^ as exemplified in Figure 2A,B with two of the members of this family of clusters (*x* = 1, [Fe_4_S_4_]^3+^; with one bound/unbound K^+^ ion): the only difference between these two structures is the (2° sphere) directional electric field exerted on the Fe_4_-centroid, *C*(Fe_4_), by a K^+^ ion, highlighted as a *brown line* in Figure 2B. More specifically, we found that this local electric field causes a shift of the cluster’s redox potential (Δ*E*_redox_), which is predicted well by classical electrostatic theory^34,68^

1 $${\Delta E}_{redox}≅\sum_{i}\frac{q}{4\pi \varepsilon_{s}\varepsilon_{0}r_{i}}$$

with *q* standing for the elementary charge, ε_s_ for the solvent dielectric constant, which is multiplied by the vacuum permittivity, ε_0_, and *i* for the number of point charges situated at distances *r*_*i*_ from the redox active center, being, in this case, effectively *C*(Fe_4_). In turn, modulation of this electric field via the removal of the alkali cations enables a dynamic control of the cluster’s redox potential, providing a simple model that mimics an analogous behavior of the redox potential observed for FeS clusters involved in so-called “gated” biochemical ETs,^22^ such as the one shown in Figure 1A for CoA dehydratase enzymes.

**Figure 2:**
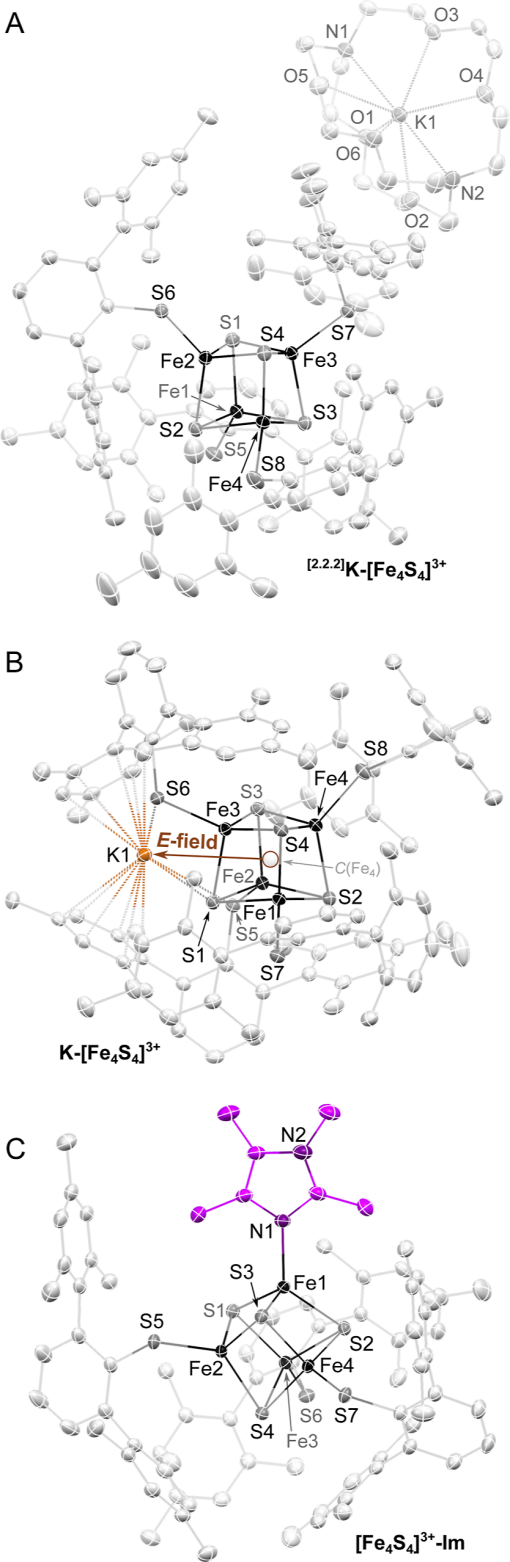
(A) Solid-state molecular structure of ^[2.2.2]^K­[Fe_4_S_4_(DmpS)_4_] in crystals of ^[2.2.2]^K­[Fe_4_S_4_(DmpS)_4_]·(C_7_H_8_).^22^ (B) Solid-state molecular structure of K­[Fe_4_S_4_(DmpS)_4_] in crystals of K­[Fe_4_S_4_(DmpS)_4_]·(C_7_H_8_),^16^ highlighting the electric field interaction between the encapsulated K^+^ ion and the iron–sulfur cluster: The Fe_4_ centroid, *C*(Fe_4_), is highlighted as a *brown circle*, and the K^+^ ion is likewise highlighted as a *brown ellipsoid*. (C) Solid-state molecular structure of [Fe_4_S_4_(DmpS)_3_(Im*)] in crystals of [Fe_4_S_4_(DmpS)_3_(Im*)]·(C_7_H_8_)_0.49_·(C_6_H_18_Si_2_O)_0.25_.^69,70^ In all panels thermal displacement ellipsoids are shown at 50% probability. Hydrogen atoms and cocrystallized solvent molecules were omitted for clarity.

In the present work, we use those cation-bound/-unbound clusters (specifically K_*x*_[Fe_4_S_4_(DmpS)_4_] and ^[2.2.2]^K_*m*_[Fe_4_S_4_(DmpS)_4_]; where *x* = 1–3, *m* = 1, 2, and DmpS^–^ = 2,6-dimesitylphenylthiolate^16,22^ ) as model compounds for Fe_4_S_4_ active sites influenced by, or devoid of, 2° sphere electrostatic effects. Leveraging the clusters’ spectroscopic signatures, we probe the electrostatic influence on electronic structure, noting that these (canonical) clusters are particularly well suited for this goal, because even small changes in their observables can be directly linked to a 2° sphere effect. To systematically track these changes, while ensuring compatibility to data collected on protein-bound clusters, we use a combination of spectroscopic methods commonly applied to FeS metalloenzymes: electron paramagnetic resonance (EPR) spectroscopy, nuclear magnetic resonance (NMR) spectroscopy, ^57^Fe Mössbauer spectroscopy, nuclear resonance vibrational spectroscopy (NRVS), and high energy resolution fluorescence-detected S K-edge X-ray absorption spectroscopy (HERFD-XAS). Furthermore, our spectroscopic investigations are complemented with comparisons to predictions based on density functional theory (DFT) calculations.

Altogether, by thoroughly evaluating the observables according to, (i) the presence or absence of a local electric field, (ii) the cluster oxidation state, and, when applicable, (iii) their temperature-dependence, we show that the 2° sphere electric field’s effect on the cluster’s electronic structure is exceedingly small, and effectively negligible at the temperatures relevant to enzyme function. Nonetheless, the effect clearly manifests at very low temperatures, and we propose an explanation for this phenomenon based on the fact that the degeneracy of the Fe_4_S_4_ complexes’ valence isomeric forms is lifted. These results are then compared to site-differentiated analogues using [Fe_4_S_4_(DmpS)_3_(Im*)] ([Fe_4_S_4_]^3+^, where Im* = 1,2,4,5-tetramethylimidazole; Figure 2C),^70^ [Fe_4_S_4_(DmpS)_3_(Im*)]^−^ ([Fe_4_S_4_]^2+^) and [Fe_4_S_4_(DmpS)_2_(Im*)_2_] ([Fe_4_S_4_]^2+^),^69^ as models for 1° sphere noncanonical (His-)­ligation, leading to a robust comparison between the 1° and 2° spheres’ effects. These results thereby well complement Suess’s work on carbene-substituted^48,49^ [Fe_4_S_4_]^1+^ clusters by expanding the evaluation of valence isomerism (or spin isomerism, respectively) to site-differentiated [Fe_4_S_4_]^3+^ and [Fe_4_S_4_]^2+^ clusters. By providing further data on the magnitude of three key descriptors, namely the redox potential difference, Δ*E*_redox_, the valence isomer energy difference, Δ*E*_VI_, and the vibrational energy differences, Δ*E*_vib_, we finally compile a comprehensive overview of the disentangled 1° vs 2° sphere influences on Fe_4_S_4_ complexes’ redox potentials, electronic structures and the relevant observables. Using the ET self-exchange (ETse) reaction as a model, we provide experimental evidence how these effects manifest in situ. Together, this could pave the way for more predictive structure–function relationship determinations in biological FeS clusters.

## 2 Results

The Results section is organized as follows: throughout Sections 2.1–2.3, we sequentially catalog and discuss the effect of 2° sphere interactions in our synthetic models on spectroscopic observables. These investigations are sorted by types of techniques and oxidation states, respectively, whereby 2.1 cumulatively covers EPR and ^57^Fe Mössbauer spectroscopy for the [Fe_4_S_4_]^3+^ (2.1.1), [Fe_4_S_4_]^2+^ (2.1.2), and [Fe_4_S_4_]^1+^ (2.1.3), redox levels, as applicable, 2.2 covers NRVS and 2.3 covers HERFD-XAS. Subsequently, Sections 2.4 and 2.5 contrast and complement our findings on the 2° sphere perturbations ([Sec sec2.1]–[Sec sec2.3]) with analogous investigations on synthetic models with 1° sphere perturbations. Toward illustrating the functional implications of these results, the final section (2.6) compares the estimated electron tunneling self-exchange rates of the respective systems to one another.

### 2.1 2° Sphere Electrostatic Effects on the EPR and ^57^Fe Mössbauer Spectra of [Fe_4_S_4_]^3+/2+/1+^ Complexes

#### 2.1.1 The Oxidized Cluster: [Fe_4_S_4_]^3+^

Due to their half-integer *S* = 1/2 spin ground state, [Fe_4_S_4_]^3+^ clusters (and oxidized HiPIPs) have intensively been studied by EPR spectroscopy. Characteristically, axial or rhombic signals are observed, with significant *g*-value distributions and average *g*-tensor values *g*_av_ > 2.^41^ Molecular model compounds in this state usually do not show a dependence of the EPR signal on solvent, but spin–spin relaxation, static dipolar, and exchange interactions generally cause the spectra of pure solid powders to differ strongly from the ones recorded in frozen solutions.^47^ In two instances, Na^+^-ion encapsulated [Fe_4_S_4_]^3+^ clusters were investigated in frozen toluene solution, namely [Na­(THF)]­[Fe_4_S_4_(DmpS)_4_] and [Na­(THF)]­[Fe_4_S_4_(TbtS)_4_].^71^ In both cases, however, a single-component rhombic, respectively axial spectrum was observed, with no evidence assigned to an electric field effect. During our investigations of K­[Fe_4_S_4_(DmpS)_4_] (**K-[Fe**_**4**_ **S**_**4**_ **]** ^**3+**^) in frozen toluene, we however unexpectedly observed a broad spectrum, evidencing at least three individual components both in X- and Q-band EPR spectra, namely an axial, a rhombic and a broader component. The spectrum collapses to a single rhombic component, if the sample is measured in frozen 2-Me-THF, or if the K^+^-ion is sequestered by [2.2.2]-cryptand to generate a well-separated ion pair. Spectra for all four scenarios of solvation/ionic separation are shown in Figure 3 (refer to the Supporting Information Figures S2–S4 for simulations of all spectra). Evidently, **K-[Fe**_**4**_ **S**_**4**_ **]** ^**3+**^ in frozen toluene exhibits a markedly distinct spectrum, whereas the three remaining cases present preserved *g*_*y*_ and similar *g*_*x*_ and *g*_*z*_ values (Table S1). All four spectra present *g*_av_ > 2, in fair agreement with existing literature.^41^ This leads us to infer that toluene is unable to dissociate the K^+^-ion from the anionic cubane, while solvation by 2-Me-THF suffices to generate a well-separated ion pair. Similar spectra with multiple components, as observed for **K-[Fe**_**4**_ **S**_**4**_ **]** ^**3+**^ in frozen toluene, have been documented for a variety of HiPIPs,^72,73^ as well as γ-irradiated single-crystalline [Fe_4_S_4_(RS)_4_]^2–^ model complexes^74–76^ and their origin was ascribed to the trapping of valence localized states. This prompted us to investigate the behavior of the EPR line at varying temperatures (Figure S3). As described before,^48,49^ if the ratios between the individual components exhibit Boltzmann-type behavior, this may allow to estimate the order of magnitude of their energetic separation, Δ*E*_VI_. For **K-[Fe**_**4**_ **S**_**4**_ **]** ^**3+**^, the transitions vary only marginally between 4.5 and 10 K, but the individual components appear to follow different thermal relaxation, as can be seen from a significant change of the line shape in the “central” *g* region at 20 and 30 K. This was also observed in some HiPIPs.^73^ The three components can be assigned in several ways, none of which produced a physically relevant Boltzmann-type relation between the individual population ratios with varying *T*. For example, two possible simulations at X- and Q-band as well as the corresponding putative temperature-dependent evolutions of the three components are shown in Figure S4C,F. We hypothesize that the three observed spectral components could be due to the existence of several conformers of **K-[Fe**_**4**_ **S**_**4**_ **]** ^**3+**^ in toluene solution, which itself is a consequence of the electrostatic interaction of the encapsulated K^+^-ion with the anionic [Fe_4_S_4_(DmpS)_4_]^−^ complex. In each of these conformers, a different valence isomer may populate the observed ground-state, giving rise to a marginally different EPR signal. Alternatively (i) the energetic separation between the valence isomers could lie within an order of magnitude that is smaller than *k*_B_ *T* (for *T* = 4.5–10 K; this corresponds to Δ*E*_VI_ ≤ 7 cm^–1^), and thus nonquantifiable in this manner, or, (ii) the different thermal relaxation behaviors of the three components could mask the effect. Regardless, the observation of multiple EPR lines in the spectrum of **K-[Fe**_**4**_ **S**_**4**_ **]** ^**3+**^ can be directly linked to the presence of a local electric field gradient close to the Fe_4_S_4_ core. Consequently, it leads us to reinforce the notion that the physical origin of this phenomenon in HiPIPs is similarly linked to an electric field gradient generated by the asymmetric (protein) environment, as proposed based on early theoretical investigations.^62^

**Figure 3:**
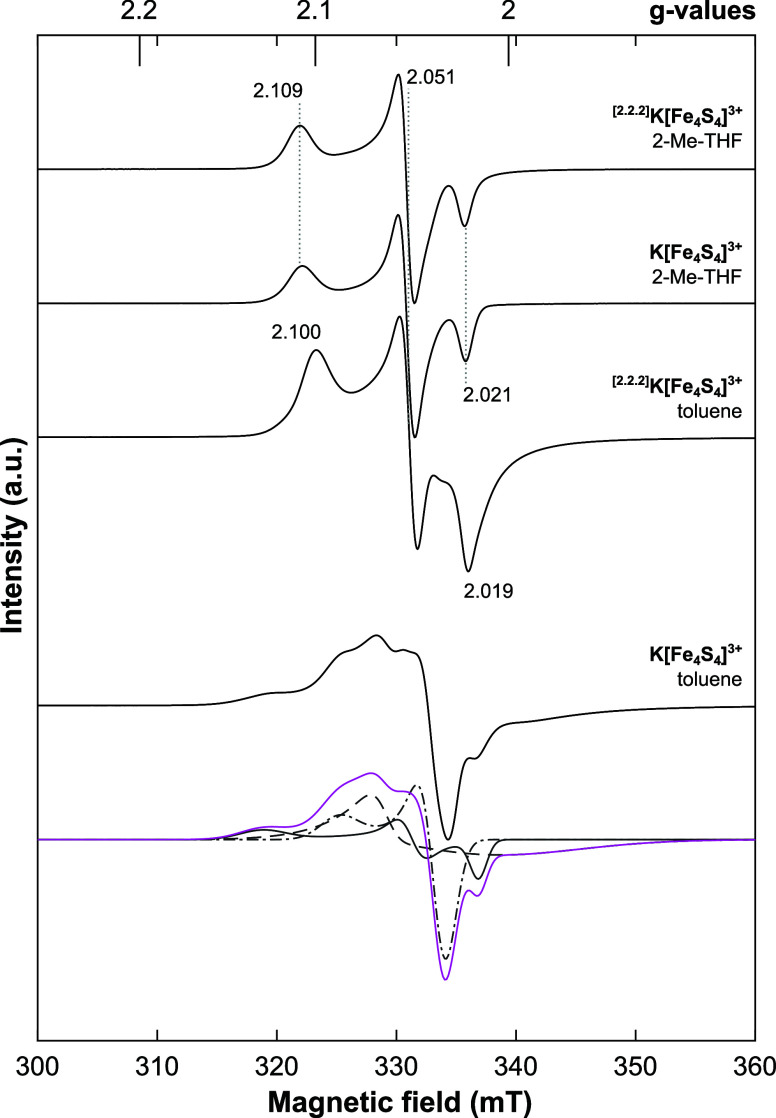
X-band continuous-wave (cw) EPR spectra (*black lines*) and a simulation (*magenta line*) deconvoluted into individual components (*gray lines*) of frozen toluene and 2-Me-THF solutions of **K-[Fe**_**4**_ **S**_**4**_ **]** ^**3+**^ and ^**[2.2.2]**^ **K-[Fe**_**4**_ **S**_**4**_ **]** ^**3+**^ recorded at 10 K (see Table S1 for simulation parameters).

Complementary Mössbauer studies were performed on a toluene solution of **K-[Fe**_**4**_ **S**_**4**_ **]** ^**3+**^ and on a THF solution of ^[2.2.2]^K­[Fe_4_S_4_(DmpS)_4_] (^**[2.2.2]**^ **K-[Fe**_**4**_ **S**_**4**_ **]** ^**3+**^). The low-field 80 K spectra present an asymmetric doublet, with the high-velocity line being the more broadened. They can be nicely reproduced by considering two Fe sites in a 1:1 ratio (Figure S19). The determined nuclear parameters are fairly consistent with those previously obtained for HiPIP-type clusters (Table S5), evidencing a *di*-ferric pair and a mixed-valent one.^77^ The two 6 K solution spectra recorded while applying a 7 T external magnetic field along the γ-ray’s direction are very similar (Figure S20E). However, whereas the spectrum of ^**[2.2.2]**^ **K-[Fe**_**4**_ **S**_**4**_ **]** ^**3+**^ is also very similar to that of the corresponding powder sample, that of the **K-[Fe**_**4**_ **S**_**4**_ **]** ^**3+**^ cluster significantly differs (Figure S20A,C, respectively). Satisfactory simulations of the high-field 6 K THF solution spectra of ^**[2.2.2]**^ **K-[Fe**_**4**_ **S**_**4**_ **]** ^**3+**^ and toluene solution spectra of **K-[Fe**_**4**_ **S**_**4**_ **]** ^**3+**^ were obtained assuming two equally contributing Fe sites with a *S* = 1/2 spin state in the slow relaxation regime (Figures S21–S24 and Tables S6–S9). As previously reported in the literature, the *di*-ferric pair presents positive hyperfine coupling constants while negative values are obtained for the Fe^2.5+^Fe^2.5+^ pair.^78^ The same sets of parameters do not allow to reproduce the low-field 6 K spectra. However, either adding a contribution of the powder spectrum for ^**[2.2.2]**^ **K-[Fe**_**4**_ **S**_**4**_ **]** ^**3+**^ or a fast-relaxation system for **K-[Fe**_**4**_ **S**_**4**_ **]** ^**3+**^ allows satisfying matches to the low-field experimental data. These combinations may reflect an intermediate electronic relaxation regime adopted at low-field for the clusters in solution, as previously evidenced on HiPIP models.^47^ It may be noticed that for the high-field data, a better fit of the absorptions at −1.1 and 1.8 mm s^–1^ and of the lines at −2.15 and 2.9 mm s^–1^, which are mainly due to the mixed-valent and *di*-ferric pairs, respectively, is obtained for ^**[2.2.2]**^ **K-[Fe**_**4**_ **S**_**4**_ **]** ^**3+**^. This evidences a different behavior for the two complexes in solution, in full agreement with the hypotheses drawn above, based on EPR spectroscopy.

For [Fe_4_S_4_]^3+^ in the *S* = 1/2 state, we furthermore evaluated spin-coupling patterns and energy splittings of valence isomers by means of broken-symmetry (BS) DFT calculations^50,79–81^ in the core cubane in the absence of K^+^, and for the overall uncharged assembly in the presence of one K^+^-ion. For both the native [Fe_4_S_4_]^3+^ core, and the K^+^-ion encapsulated variant, we find the respective valence isomer ground state to be best described by a β-(Fe^2.5+^Fe^2.5+^) pair antiferromagnetically coupled an α-(Fe^3+^Fe^3+^) pair, as expected. Both clusters adopt *g*_av_ > 2. Also, in agreement with experiment, broadening due to a distribution of *g*-tensor values is considerably more pronounced in the cation-encapsulating variant (Table S20). Furthermore, we find *g*_*y*_, *g*_*z*_ > 2 in both variants, but *g*_*x*_ < 2 in **K-[Fe**_**4**_ **S**_**4**_ **]** ^**3+**^, which is similar to the experimentally determined *g*-values (Table S1). In **K-[Fe**_**4**_ **S**_**4**_ **]** ^**3+**^, the lower-valent iron pair was calculated to preferably span the plane closest to the cation, as hypothesized by our symmetry considerations (Figure S1) and based on the simple electrostatic argument. In line with an electrostatic effect on valence isomerism, the energetic splitting between the BS-solutions is amplified when a K^+^ ion is introduced to the assembly, with a total energy span of approximately 12 kJ mol^–1^ (Figure S59). The calculated Mössbauer parameters are condensed in Table S24.

#### 2.1.2 The [Fe_4_S_4_]^2+^ Cluster

Because of their diamagnetic *S* = 0 spin ground state, the [Fe_4_S_4_(DmpS)_4_]^2–^ clusters are EPR silent. Accordingly, we focused on their Mössbauer spectra.^41^ Measurements were performed on K_2_[Fe_4_S_4_(DmpS)_4_] (**K**_**2**_ **-[Fe**_**4**_ **S**_**4**_ **]** ^**2+**^) dissolved in toluene and on ^[2.2.2]^K_2_[Fe_4_S_4_(DmpS)_4_] (^**[2.2.2]**^ **K**_**2**_ **-[Fe**_**4**_ **S**_**4**_ **]** ^**2+**^) dissolved in THF. The low-field spectra recorded at 6 and 80 K present doublets, as expected for diamagnetic [Fe_4_S_4_]^2+^ clusters. However, their comparison revealed different behaviors depending on the presence or absence of the K^+^ ions (Figure 4). Whereas the 6 and 80 K solution spectra of **K**_**2**_ **-[Fe**_**4**_ **S**_**4**_ **]** ^**2+**^ are almost superimposable, those of ^**[2.2.2]**^ **K**_**2**_ **-[Fe**_**4**_ **S**_**4**_ **]** ^**2+**^ are not (Figure 4A,B, respectively). These comparisons evidenced a very similar central position for the four spectra and a temperature dependence of the quadrupole splitting parameter, Δ*E*_Q_, for ^**[2.2.2]**^ **K**_**2**_ **-[Fe**_**4**_ **S**_**4**_ **]** ^**2+**^ in contrast to the constant value for **K**_**2**_ **-[Fe**_**4**_ **S**_**4**_ **]** ^**2+**^. The same behavior was observed on the powder spectra (see Supporting Information Figure S13), strongly suggesting that those features are intrinsic properties of the compounds.

**Figure 4:**
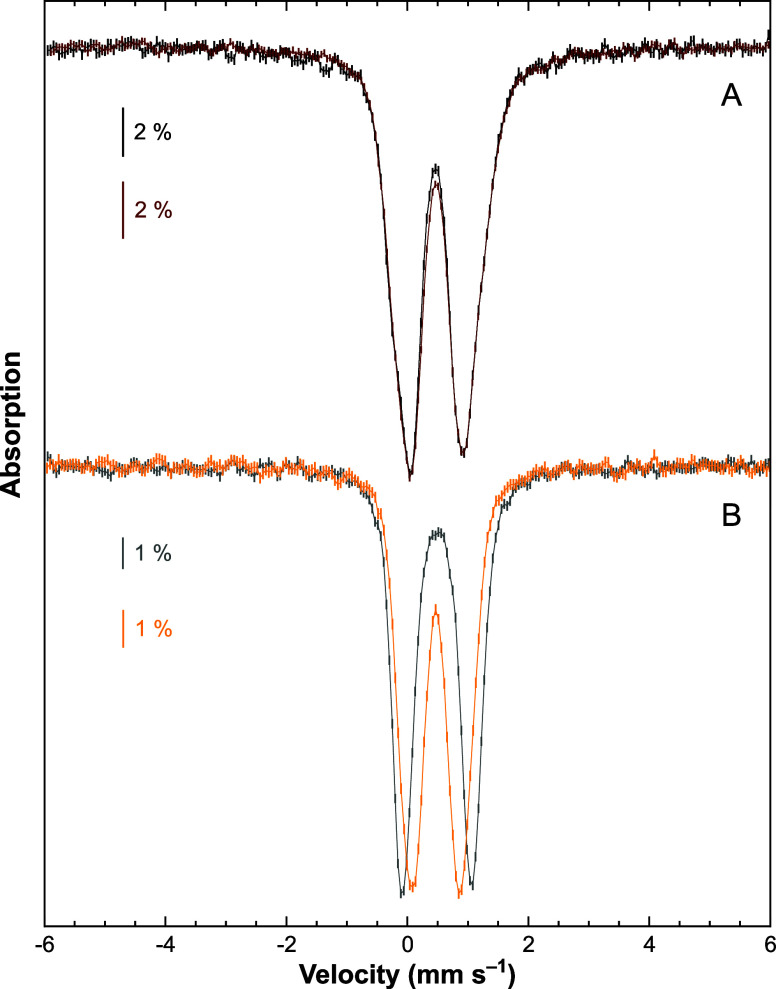
Mössbauer spectra (*vertical bars*) of the 1.5 mM toluene solution of ^57^Fe-enriched **K**_**2**_ **-[Fe**_**4**_ **S**_**4**_ **]** ^**2+**^ (A) and of the 1.2 mM THF solution of ^57^Fe-enriched ^**[2.2.2]**^ **K**_**2**_ **-[Fe**_**4**_ **S**_**4**_ **]** ^**2+**^ (B) recorded using a 0.06 T external magnetic field applied parallel to the γ-rays. Spectra recorded at 5.8 K are drawn in *black* and *gray* on A and B, respectively. Those recorded at 80 K are displayed in *brown* and *yellow* in A and B, respectively.

From a structural standpoint, the solid-state geometry of ^**[2.2.2]**^ **K**_**2**_ **-[Fe**_**4**_ **S**_**4**_ **]** ^**2+**^ ^22^ revealed very similar tetrahedral coordination environments for the four Fe centers. Accordingly, it was possible to reproduce the Mössbauer powder spectra with a single Fe site (Figure S16D,E). However, the environments of the Fe centers in **K**_**2**_ **-[Fe**_**4**_ **S**_**4**_ **]** ^**2+**^ are more distorted,^16,22^ differing more significantly from a regular tetrahedron and with respect to one another. In consequence, two Fe sites were considered to fit the corresponding Mössbauer powder spectra, but two line widths had to be implemented (Figure S16A–C). The determined parameters are fully consistent with those previously obtained for diamagnetic [Fe_4_S_4_]^2+^ clusters (Table S3), with isomer shift values characteristic of delocalized Fe^2.5+^Fe^2.5+^ pairs.

The best simulations of the solution spectra are displayed in Figure S15: two diamagnetic Fe sites were considered for both solutions (refer to the Supporting Information, Figure S17, for simulations of the THF solution spectra of ^**[2.2.2]**^ **K**_**2**_ **-[Fe**_**4**_ **S**_**4**_ **]** ^**2+**^ with a single Fe site), and the parameters are listed in Table 1. The quadrupole splitting of the two sites presents similar values at 6 and 80 K for **K**_**2**_ **-[Fe**_**4**_ **S**_**4**_ **]** ^**2+**^, whereas a reduction of 0.3–0.4 mm s^–1^ is observed upon the increase of the temperature for ^**[2.2.2]**^ **K**_**2**_ **-[Fe**_**4**_ **S**_**4**_ **]** ^**2+**^. Similar variations were determined for the powder spectra (Table S3). It should also be noticed that the line width of site 2 of **K**_**2**_ **-[Fe**_**4**_ **S**_**4**_ **]** ^**2+**^ is large (≈0.5 mm s^–1^) for both the powder and the solution samples and that of site 1 increases upon dissolution. An important variation of Δ*E*_Q_ of site 2 is detected upon dissolution in toluene (an increase of ≈0.2 mm s^–1^ at 6 K). This is in line with the equilibrium between the two forms of **K**_**2**_ **-[Fe**_**4**_ **S**_**4**_ **]** ^**2+**^, labeled **I** and **II**, in solution, which was evidenced in our previous work.^22^

**Table 1:** Parameters Determined for the Simulations of the Solution Spectra of the **K**_**2**_ **-[Fe**_**4**_ **S**_**4**_ **]** ^**2+**^ Cluster in Toluene and the ^**[2.2.2]**^ **K**_**2**_ **-[Fe**_**4**_ **S**_**4**_ **]** ^**2+**^ Cluster in THF Shown in Figure S15 ^a^

		**K**_**2**_ **-[Fe**_**4**_ **S**_**4**_ **]** ^**2+**^		^**[2.2.2]**^ **K**_**2**_ **-[Fe**_**4**_ **S**_**4**_ **]** ^**2+**^	
		site 1	site 2	site 1	site 2
experiment	δ (mm s^–1^)	0.47 (0.47)	0.48 (0.49)	0.49 (0.47)	0.49 (0.47)
	Δ*E*_Q_ (mm s^–1^)	0.78 (0.77)	1.30 (1.28)	0.98 (0.62)	1.29 (1.02)
	η	1.0 (−)	0.7 (−)	0.6 (−)	0.4 (−)
	Γ_fwhm_ (mm s^–1^)	0.33 (0.36)	0.47 (0.49)	0.29 (0.32)	0.29 (0.32)

		higher-lying	lower-lying	all spin isomers
theory	δ (mm s^–1^)	0.20	0.20	0.26
	Δ*E*_Q_ (mm s^–1^)	0.53–0.54	1.05–1.09	0.78–0.93
	η	0.58–0.60	0.61–0.92	0.30–0.69

a In the latter, site 1 appears in *dark grey* and site 2 in *light grey*, respectively. Diamagnetic sites were considered. Values for the 80 K spectra are indicated in parentheses. The bottom part of the table additionally summarizes the DFT calculated values for the truncated model compounds.

In general, the temperature dependence of Δ*E*_Q_ is related to nearly degenerate electronic states presenting different orbital compositions.^82^ This has been previously observed in high-spin ferrous porphyrin systems^83^ and in planar tricoordinate high-spin Fe^2+^ complexes.^84^ For [Fe_4_S_4_]^2+^, the electronic states that are close in energy are associated with different locations of the two delocalized pairs (different “spin isomers”). Three possibilities exist that are degenerate for a regular Fe_4_ tetrahedron with the four Fe^2.5+^ ions in identical environments. Indeed, the solid-state structure of ^**[2.2.2]**^ **K**_**2**_ **-[Fe**_**4**_ **S**_**4**_ **]** ^**2+**^ ^22^ features a quite regular Fe_4_-core, with similar Fe ions, whereas the Fe_4_ structure in **K**_**2**_ **-[Fe**_**4**_ **S**_**4**_ **]** ^**2+**^ ^16,22^ is more distorted, with more inequivalent Fe ions. These structural features, combined with the temperature-dependent behavior of Δ*E*_Q_, strongly suggest that the three electronic states are nearly degenerate in ^**[2.2.2]**^ **K**_**2**_ **-[Fe**_**4**_ **S**_**4**_ **]** ^**2+**^ whereas more pronounced separations are anticipated for **K**_**2**_ **-[Fe**_**4**_ **S**_**4**_ **]** ^**2+**^. Though we are not able to determine the exact magnitude of the splitting, Δ*E*_VI_, in this manner, our result demonstrates that the K^+^ ions are noninnocent when it comes to the energetic ordering of the isomeric states—even in the diamagnetic [Fe_4_S_4_]^2+^ complexes. Furthermore, the fact that the spectra of **K**_**2**_ **-[Fe**_**4**_ **S**_**4**_ **]** ^**2+**^ are nearly superimposable at 80 K and at 6 K implies that the energetic splitting between the isomeric forms may be either significantly larger than 80 *k*_B_ (56 cm^–1^), or smaller than 6 *k*_B_ (4 cm^–1^).

DFT calculations on smaller models of the resting oxidation state, [Fe_4_S_4_]^2+^, support the notion of a two-level system of spin isomers in **K**_**2**_ **-[Fe**_**4**_ **S**_**4**_ **]** ^**2+**^, which is in line with our conceptual symmetry considerations (Figure S1). Thereby, the isomer populating the ground state is that in which orientation of the antiferromagnetic coupling between the Fe_2_S_2_ subunits (i.e., the normal between the two delocalized *S* = 9/2 [Fe_2_S_2_]^1+^ rhombs; *shaded planes* in Figure S58) is perpendicular to the electric field gradient. For **[Fe**_**4**_ **S**_**4**_ **]** ^**2+**^, we emphasize that the DFT-predicted ordering of the spin isomeric forms is a result of the method and the cluster’s symmetry but cannot be ascribed to electric field effects due to the absence of alkali cations in the vicinity of the cluster. Conceptually, they should rather be viewed as degenerate. This highlights the limitations of the BS-DFT approach in describing the electronic structure of these systems. Nonetheless, the calculated Mössbauer parameters for all spin isomeric states of both systems are condensed in Tables S21–S23 of the Supporting Information. While the δ-values appear largely underestimated, the experimentally observed trends in Δ*E*_Q_ and η are excellently reproduced by our calculations, advocating similar Δ*E*_Q_-values for all isomers of **[Fe**_**4**_ **S**_**4**_ **]** ^**2+**^, but significantly different Δ*E*_Q_-values for the two isomeric forms of **K**_**2**_ **-[Fe**_**4**_ **S**_**4**_ **]** ^**2+**^. This particularly closely reflects the behavior of the solid-state Mössbauer spectra of our model [Fe_4_S_4_]^2+^ systems (Figure S16).

#### 2.1.3 The Reduced Cluster: [Fe_4_S_4_]^1+^

Like [Fe_4_S_4_]^3+^ clusters, due to their half-integer spin, [Fe_4_S_4_]^1+^ complexes (and reduced Fds) are also commonly studied by EPR spectroscopy. Most Fds, as well as most corresponding synthetic models exhibit an *S* = 1/2 ground state,^85^ although *S* = 3/2 has also been observed,^46,55,56^ including the series of clusters studies here.^16^ In contrast to the [Fe_4_S_4_]^3+^ complexes, however, [Fe_4_S_4_]^1+^ clusters characteristically present *g*_av_ < 2.^41^ As we described previously, K_3_[Fe_4_S_4_(DmpS)_4_] (**K**_**3**_ **-[Fe**_**4**_ **S**_**4**_ **]** ^**1+**^) exhibits *S* = 3/2 and *S* = 1/2 spin ground states in frozen toluene solution (Figure S9; 95% *S* = 3/2 and 5% *S* = 1/2, respectively), and a pure *S* = 3/2 ground state, in a powdered sample.^16^ Entirely different EPR spectra are observed in a frozen 2-Me-THF solution, where the solvent can facilitate separation of K^+^-ions from the cluster assembly: Here, the complex exhibits a signal evidencing at least three components with *S* = 1/2 spin state and distinct *g* values. A very broad *S* = 3/2 component may be hidden in the baseline but cannot be discerned clearly by EPR spectroscopy (Figure S6; refer to the complementary Mössbauer data discussed below). This spectrum remains unchanged, even if up to 40 equiv of additional free K^+^ ions are added (Figure 5A).

**Figure 5:**
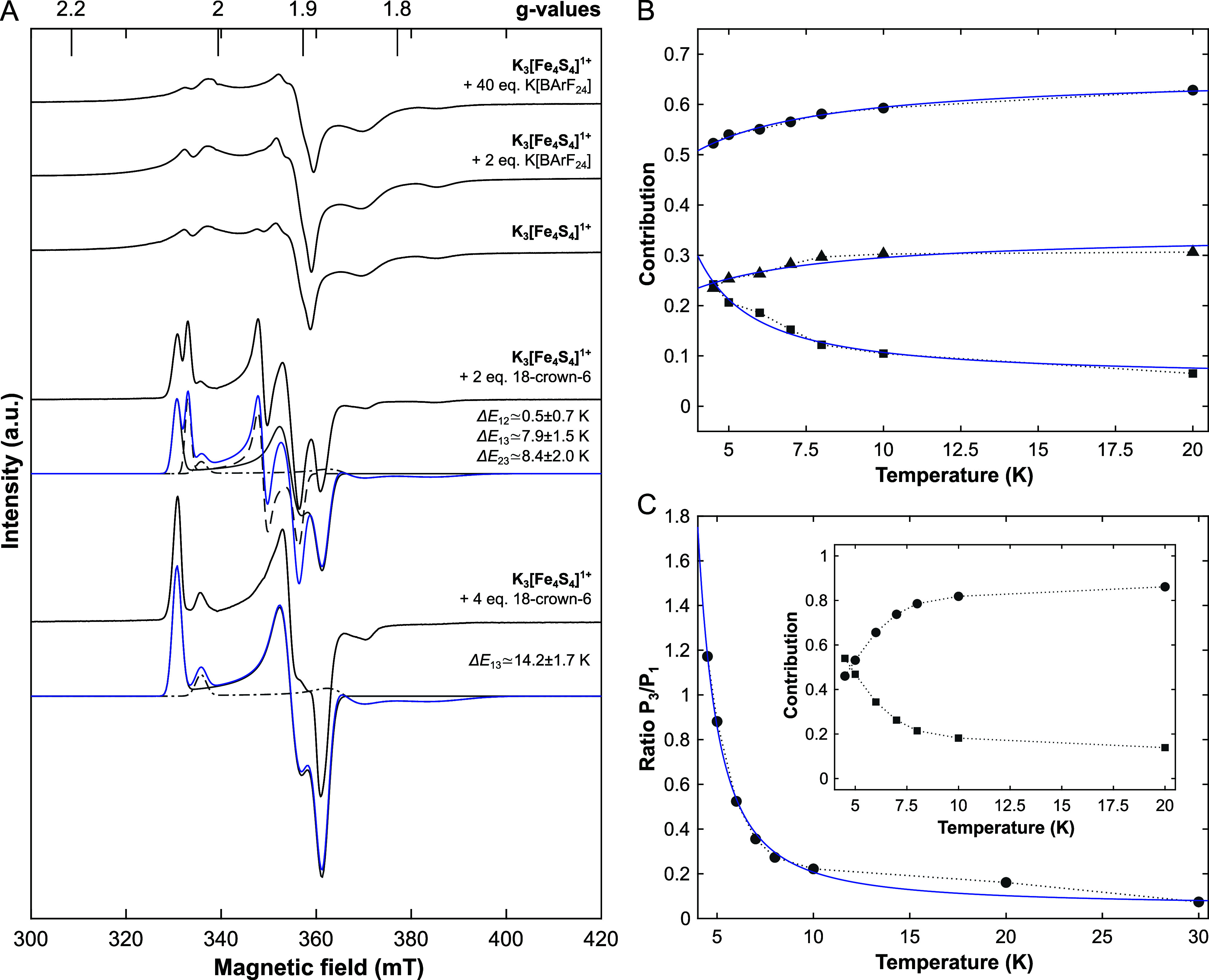
(A) X-band cw EPR spectra (*black lines*) and simulations (*blue lines*) deconvoluted into individual components (*gray lines*) of frozen 2-Me-THF solutions of **K**_**3**_ **-[Fe**_**4**_ **S**_**4**_ **]** ^**1+**^ in the presence of varying equivalents of 18-crown-6 or K­[BArF_24_], respectively, and recorded at 10 K (see Table S1 for simulation parameters). The relative energy differences between the components based on a Boltzmann-type analysis are indicated alongside the spectra. (B) Temperature evolution of the weights of the three individual components (1: *dots*; 2: *triangles*; 3: *squares*) according to the fitting model presented in Figure S5B,C and data in Figure S7. *Blue lines* represent fitting of the data according to Boltzmann-functions of the type: *P*_*i*_ = (1/*Q*) exp­(−*E*_*i*_/(*k*_B_ *T*)), where *P*_*i*_ denotes the (observed) Boltzmann population, *E*_*i*_ is the state’s energy, *k*_B_ is the Boltzmann constant, *T* is temperature and *Q* is the effective normalization denominator.^48^ Based on this, the relative energy differences between the three components are approximated as Δ*E*_12_ = 0.5 ± 0.7 *k*_B_ (0.3 cm^–1^), Δ*E*_13_ = 7.9 ± 1.5 *k*_B_ (5.5 cm^–1^) and Δ*E*_23_ = 8.4 ± 2.0 *k*_B_ (5.8 cm^–1^). Due to the different line widths in the spectrum recorded at 30 K, this spectrum was omitted from the Boltzmann-type analysis. (C) Temperature evolution of the population ratio between component 3 and component 1, *P*_3_/*P*_1_, based on the fitting model as in (B). Data are shown as *dots* and a *blue line* is a fit of the data according to a Boltzmann function of the type: (*P*_3_/*P*_1_) = *C* exp­(Δ*E*_13_/(*k*_B_ *T*)). Here, the factor *C* accounts for possible differences in signal responsivity.^48^ *Inset*: temperature evolution of the individual component populations (1: *dots*; 3: *squares*) for comparison with the data shown in panel (B). The relative energy difference is approximated as Δ*E*_13_ = 14.2 ± 1.7 *k*_B_ (9.9 cm^–1^).

A possible assignment of three components is proposed in the simulation shown in Figure S5A. While there is ambiguity in the arrangement of components 1 and 2, we are confident in our assignment of component 3 because it is also identifiable in the spectra of **K**_**3**_ **-[Fe**_**4**_ **S**_**4**_ **]** ^**1+**^ measured in frozen 2-Me-THF in the presence of 2 and 4 equiv of 18-crown-6, respectively (*dashed-dotted lines* in Figure 5A). Because sharp transitions appear, which are, in part, common to both spectra, similar components can be assigned clearly in these spectra. As reported previously,^22^ due to the high affinity of the cluster assembly toward K^+^, 18-crown-6 is not a strong enough chelating agent to completely remove K^+^ from **K**_**3**_ **-[Fe**_**4**_ **S**_**4**_ **]** ^**1+**^. Instead, the chemical equilibria summarized in Figure 6 are perturbed.

**Figure 6:**
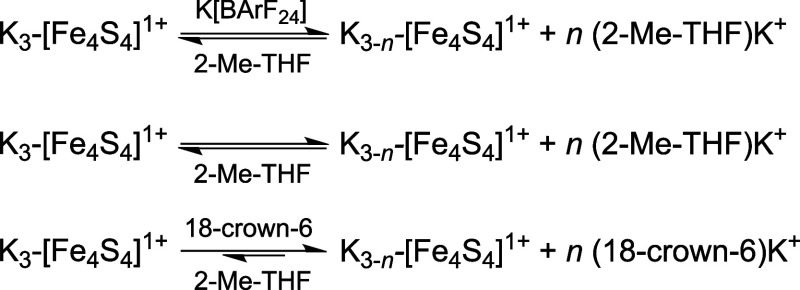
Perturbations of the chemical equilibria of K^+^-ion binding in **K**_**3**_ **-[Fe**_**4**_ **S**_**4**_ **]** ^**1+**^ in 2-Me-THF solution (*middle*) and in the presence of K­[BArF_24_] (*top*) and 18-crown-6 (*bottom*), respectively.

In line with existing literature on [Fe_4_S_4_]^1+^ complexes, all simulated parameters (Figure S5 and Table S1) show *g*_av_ < 2. To the best of our knowledge, similar multicomponent *S* = 1/2 EPR spectra of [Fe_4_S_4_]^1+^ clusters were previously only known to arise after γ-irradiation of single-crystalline [Fe_4_S_4_(RS)_4_]^2–^ model compounds,^74–76^ or more recently in 3:1 site-differentiated [Fe_4_S_4_(NHC)_3_(L/X)]^+/0^ complexes (where NHC refers to a bulky N-heterocyclic carbene ligand and L/X are arbitrary neutral/anionic ligands of varying field strength).^48^ The parameters of the simulated spectra of **K**_**3**_ **-[Fe**_**4**_ **S**_**4**_ **]** ^**1+**^ in the presence of 2 and 4 equiv of 18-crown-6, respectively, were fitted to data recorded at variable temperatures in the range of 4.5 to 30 K. This reveals that the three observed components vary in relative contribution at the expense of one another (Figure S7A,C) and that the temperature evolution itself can be fitted fairly with Boltzmann-type functions (Figure 5B,C). In contrast to our observation in **K-[Fe**_**4**_ **S**_**4**_ **]** ^**3+**^, this behavior indeed implies an interconversion of states, rather than the presence of multiple conformers, as the origin of this phenomenon. Notably, components 1 and 2 (*solid* and *dashed gray lines* in Figure 5) vary strongly with respect to component 3, but very little with respect to each other, and show almost identical energies (Δ*E*_12_ ≅ 0.5 ± 0.7 *k*_B_). We thus believe that they are the higher-lying states of the same valence isomer, but of the geometries (**I**) and (**II**) of the **K**_**2**_ **-[Fe**_**4**_ **S**_**4**_ **]** ^**1+**^ assembly, which differ in the distortion of the Fe atom’s first coordination tetrahedron and were described in our previous works.^16,22^ For this type of assembly (**K**_**2**_ **-[Fe**_**4**_ **S**_**4**_ **]** ^***n*+**^), judging by symmetry considerations and supported by DFT calculations (Figures S1 and S60), a two-level system should indeed be expected. Therefore, based on the two data sets (Figure S7), we estimate the energy separation of the two valence-arrangements, Δ*E*_VI_, (components [1, 2] and component 3) of the **K**_**2**_ **-[Fe**_**4**_ **S**_**4**_ **]** ^**1+**^ complex to be in the order of magnitude between 8 (Δ*E*_13_ & Δ*E*_23_) and 14 *k*_B_ (Δ*E*_13_), or 5 and 10 cm^–1^, depending on the data set (Figure 5B vs [Fig fig5]C). From the data it remains unclear how the signal of assemblies of the type **K-[Fe**_**4**_ **S**_**4**_ **]** ^**1+**^, **K**_**3**_ **-[Fe**_**4**_ **S**_**4**_ **]** ^**1+**^ and **K**_**4**_ **-[Fe**_**4**_ **S**_**4**_ **]** ^**1+**^ would appear and how their *g*-values and line shape parameters would compare. It is however entirely possible that the ground states of the lowest-lying valence isomers of **K-[Fe**_**4**_ **S**_**4**_ **]** ^**1+**^ and **K**_**2**_ **-[Fe**_**4**_ **S**_**4**_ **]** ^**1+**^, for example, display similar spectra, because the excess electron must have a similar local environment in both cases. Based on these considerations, regarding the equilibria outlined in Figure 6, we hypothesize that in the presence of 2 equiv of 18-crown-6, **K**_**3–*n***_ **-[Fe**_**4**_ **S**_**4**_ **]** ^**1+**^ assemblies are formed where *n* is approximately 1, and in the presence of 4 equiv, *n* is between 1 and 2. This would be in line with the order of magnitude of the binding constants of K^+^ ions to the [Fe_4_S_4_(DmpS)_4_]^*n*−^ inferred from our electrochemical investigations, compared to the affinity of 18-crown-6 to K^+^.^22^

To further corroborate the observed differences between the powder and solution samples of **K**_**3**_ **-[Fe**_**4**_ **S**_**4**_ **]** ^**1**^, the corresponding Mössbauer spectra were recorded and are displayed in Figure S25. The 6 K spectra clearly showed a contribution of the all-ferrous cluster, which was subtracted. Evidently, the solution spectra are broader than those recorded on a powder sample. At high-field (7 T), the powder spectrum expands on a narrow velocity window, as previously observed for *S* = 3/2 [Fe_4_S_4_]^1+^ clusters in proteins,^54^—fully consistent with our EPR spectroscopy studies.^16^ Hence, the main features of the low- and high-field 6 K powder spectra can be reproduced well, considering a *S* = 3/2 system in the fast and slow relaxation regime, respectively (Figure S26). Upon dissolution in THF, however, absorption is observed on both edges of the 6 K high-field spectrum, indicating the presence of *S* = 1/2 species.^55^ The high-field spectrum is strongly reminiscent of those observed for [Fe_4_S_4_(RS)_4_]^3–^ (R = Ph, CH_2_Ph) in solution^86^ or the reduced form of IspH.^87^ Due to the lack of an unambiguous *S* = 3/2 EPR signal for the THF solution of **K**_**3**_ **-[Fe**_**4**_ **S**_**4**_ **]** ^**1+**^, the 7 T Mössbauer spectrum was tentatively simulated considering a single *S* = 1/2 species, assuming two equally contributing iron sites, and the result is shown in Figure S27. Notably, the determined line width is large, suggesting a distribution of parameters, that supports the presence of several *S* = 1/2 species, as observed by EPR spectroscopy.

Our DFT calculations indicate that for the [Fe_4_S_4_]^1+^ oxidation state with *S* = 1/2, all **K**_***i***_ **-[Fe**_**4**_ **S**_**4**_ **]** ^**1+**^ (***i*** = 0, 1, 2, 3, 4) complexes exhibit *g*_av_ < 2 in agreement with the simulated experimental parameters. For **K-[Fe**_**4**_ **S**_**4**_ **]** ^**1+**^ and **K**_**2**_ **-[Fe**_**4**_ **S**_**4**_ **]** ^**1+**^, the valence isomers in which perpendicular orientation of the plane of Fe_2_S_2_-pairs accumulating β-spin excess to the cation are favored, while parallel alignment is favored for **K**_**3**_ **-[Fe**_**4**_ **S**_**4**_ **]** ^**1+**^ and **K**_**4**_ **-[Fe**_**4**_ **S**_**4**_ **]** ^**1+**^. Furthermore, the former two complexes exhibit similar *g*_*x*,*y*,*z*_ and *g*_av_ parameters, supporting the fact that the excess electron has an effectively equivalent local environment in both cases. While **[Fe**_**4**_ **S**_**4**_ **]** ^**1+**^ and **K-[Fe**_**4**_ **S**_**4**_ **]** ^**1+**^ do not exhibit valence isomer degeneracy, we find a two-level system for **K**_**2**_ **-[Fe**_**4**_ **S**_**4**_ **]** ^**1+**^ and **K**_**4**_ **-[Fe**_**4**_ **S**_**4**_ **]** ^**1+**^, with an energy separation of the two BS-solutions of approximately 5 and >25 kJ mol^–1^, respectively, and a three-level system for **K**_**3**_ **-[Fe**_**4**_ **S**_**4**_ **]** ^**1+**^ (see Figure S60), with an energy gap of approximately 15 kJ mol^–1^. Hence, in line with an electric field effect on the valence isomers, the overall energy gap between the different BS-configurations increases upon addition of K^+^ cations, due to amplification of the electric field gradient, and is most pronounced for **K**_**4**_ **-[Fe**_**4**_ **S**_**4**_ **]** ^**1+**^. DFT-EPR spectra and Mössbauer parameters of the lowest-energy isomers are condensed in Figure S61 and Table S26.

### 2.2 Evaluating the Nature of the 2° Sphere K^+^-Ion-Cluster-Interaction via HERFD-XAS

To investigate the physical origin of the K^+^-cluster interaction’s effect more fundamentally, we estimated the Fe–S bond covalencies in **K-[Fe**_**4**_ **S**_**4**_ **]** ^**3+**^ and **K**_**2**_ **-[Fe**_**4**_ **S**_**4**_ **]** ^**2+**^ as well as their cation-unbound congeners ^**[2.2.2]**^ **K-[Fe**_**4**_ **S**_**4**_ **]** ^**3+**^ and ^**[2.2.2]**^ **K**_**2**_ **-[Fe**_**4**_ **S**_**4**_ **]** ^**2+**^, respectively, via HERFD-XAS (Figure 7). The so-called pre-edge peak in these spectra is a superposition of transitions originating from Fe–S­(μ^3^) and Fe–S­(thiolate) bonds.^88^ They are S 1s to ψ* transitions, which are forbidden, but become observable if the vacant Fe 3d orbitals are covalently mixed with S 3p; the pure S 1s to S 3p transitions being electric dipole allowed.^24,88,89^ Accordingly, the difference between the intensities of the pre-edge peaks of K^+^-bound vs -unbound clusters provides direct information on the degree of covalency of the K^+^-cluster interaction.

**Figure 7:**
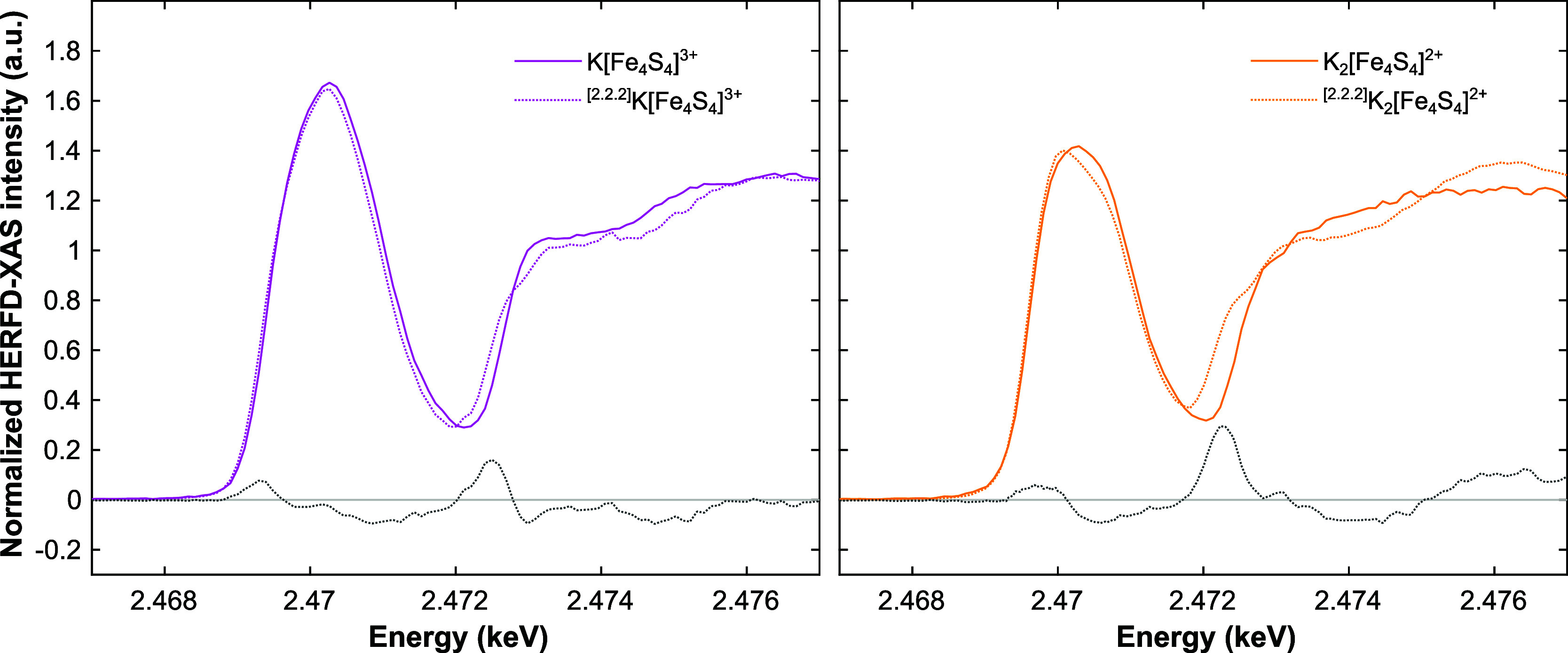
S K-edge high energy resolution fluorescence-detected X-ray absorption spectra (HERFD-XAS) of **K-[Fe**_**4**_ **S**_**4**_ **]** ^**3+**^ (*solid magenta line*, *left*) and **K**_**2**_ **-[Fe**_**4**_ **S**_**4**_ **]** ^**2+**^ (*solid yellow line*, *right*) as well as ^**[2.2.2]**^ **K-[Fe**_**4**_ **S**_**4**_ **]** ^**3+**^ (*dotted magenta line*, *left*) and ^**[2.2.2]**^ **K**_**2**_ **-[Fe**_**4**_ **S**_**4**_ **]** ^**2+**^ (*dotted yellow line*, *right*) recorded on powdered samples at room temperature in vacuo. The difference between the two respective spectra, arising after cation removal, is shown as a *dotted gray line*.

Relatedly, Solomon and co-workers previously proposed that a H-bonding-induced decrease in Fe–S covalency contributes to the modulation of the redox potential and spin topology of the [Fe_4_S_4_]^2+^ state in HiPIPs vs Fds as well as in a H-bond containing model complex.^24,88,90–92^ However, in the herein studied case, we did not observe any significant difference between the pre-edge peak intensities for both the [Fe_4_S_4_]^3+^ (Figure 7, *left*) and [Fe_4_S_4_]^2+^ complexes (Figure 7, *right*) in presence vs absence of bound K^+^-ions. Instead, the total Fe–S covalency (i.e., the total pre-edge peak intensity) appeared strictly invariable, regardless of the 2° sphere interaction to K^+^. This result is supported by fitting of the data (Table S2 and Figure S11),^93^ evidencing total and individual bond covalencies well within error of one another. Most importantly though, it demonstrates the primarily noncovalent electrostatic 2°-sphere-origin of the phenomenon studied here, in the K^+^-ion-containing compounds, as well as the electrochemical potential variation described in our previous publication.^22^

### 2.3 2° Sphere Effects on the Fe_4_S_4_ Cluster Vibrations Probed by ^57^Fe NRVS Spectroscopy

The valence- and spin-topology of Fe_4_S_4_ complexes is known to be coupled to their vibrational structure.^42,43,94^ In this regard, our group currently reported the oxidation-state-dependency of the Fe_4_S_4_ cluster vibrations in the redox series of **K**_***n***_ **-[Fe**_**4**_ **S**_**4**_ **]** ^**(4–*n*)+**^ complexes by a combined experimental and theoretical approach.^95–98^ Here, we focus on the key differences observable between the ^57^Fe nuclear resonance vibrational spectra (NRVS)^99,100^ of the K^+^-ion containing and K^+^-ion free congeners of the [Fe_4_S_4_]^2+^ and [Fe_4_S_4_]^3+^ cubanes, respectively (Figure 8A), to delineate the order of magnitude at which the cluster vibrations are perturbed by these 2° sphere electrostatic interactions. The difference spectra, summarized in Figure 8B, show that several vibrational bands are sensitive to the encapsulated ions, as they appear to split into multiplets upon incorporation of K^+^ into the assembly. Within a crude analysis, the bathochromic shifts of these features, taken as approximate bounds for Δ*E*_vib_, range from 9 to 23 cm^–1^ (Figure 8B). These energies are very small and are in fact in a similar order of magnitude as we estimate for the electrostatic influence on Δ*E*_VI_ (ca. ≤10 cm^–1^). As the basic electronic architectures of the two respective complexes are equivalent (i.e., having superimposable electronic absorption spectra; both being “pairs-of-pairs”),^22^ the perturbation of these vibrational levels by the 2° sphere interaction to the K^+^-ions and, in turn, their coupling with the electronic and magnetic levels of the valence and spin-topology could be a mechanism by which the isomerism is ultimately controlled in these types of structures.

**Figure 8:**
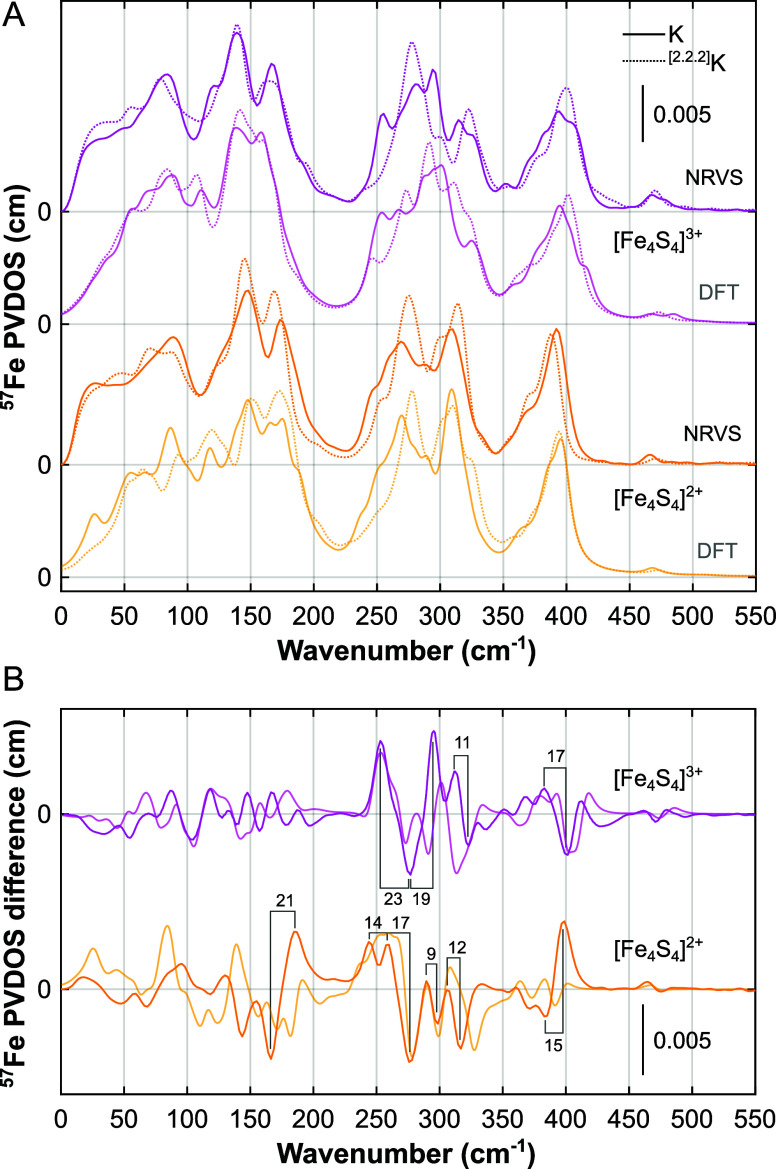
(A) ^57^Fe NRVS PVDOS spectra of **K-[Fe**_**4**_ **S**_**4**_ **]** ^**3+**^ (*solid magenta line*, *top*) and **K**_**2**_ **-[Fe**_**4**_ **S**_**4**_ **]** ^**2+**^ (*solid yellow line*, *bottom*) as well as ^**[2.2.2]**^ **K-[Fe**_**4**_ **S**_**4**_ **]** ^**3+**^ (*dotted magenta line*, *top*) and ^**[2.2.2]**^ **K**_**2**_ **-[Fe**_**4**_ **S**_**4**_ **]** ^**2+**^ (*dotted yellow line*, *bottom*) recorded on ^57^Fe-enriched (>95%), powdered samples at 30–40 K. (B) ^57^Fe NRVS PVDOS difference spectra upon addition of one, respectively two cations to the [Fe_4_S_4_]^3+^ (*magenta*), and [Fe_4_S_4_]^2+^ (*yellow*) cubanes. Selected energy differences are highlighted by *black lines*. In both panels, the DFT-PVDOS and DFT-derived difference spectra are shown as faint lines.

To confirm and rationalize this further, simulations of the ^57^Fe NRVS spectra for the complexes at the two redox levels, [Fe_4_S_4_]^2+^ and [Fe_4_S_4_]^3+^, with or without K^+^ ions encapsulation (“2 × 2 set”), were carried out using “extended” DFT modeling (as detailed in the Supporting Information), which retained the entire DmpS^–^-ligands. Our structural optimization largely preserved the positions of the K^+^ ions with respect to the FeS core (Figure S62). Furthermore, the relative trends observed using ^57^Fe NRVS for the 2 × 2 set are followed well in the DFT-based ^57^Fe PVDOS spectra (Figures 8A and S64): as typically found in the ^57^Fe NRVS spectra of FeS cubanes,^96–98^ the normal modes of Fe–S­(thiolate) and Fe–S­(μ^3^) characters in all four systems are clustered at vibrational energies (i) ∼350–430 cm^–1^ (mostly Fe–S­(thiolate) character; Fe–S_HIGH_) and (ii) ∼230–340 cm^–1^ (mostly Fe–S­(μ^3^) character; Fe–S_LOW_). The degree of degeneracy in these FeS modes becomes diminished upon either 1 e^–^ oxidation (Figure S64, *top*) or K^+^-ion encapsulation (Figure S64, *bottom*). This manifested as broadening of the corresponding ^57^Fe-PVDOS bands, e.g. when the spectra of either **[Fe**_**4**_ **S**_**4**_ **]** ^**3+**^ or **K**_**2**_ **-[Fe**_**4**_ **S**_**4**_ **]** ^**2+**^ are referenced against **[Fe**_**4**_ **S**_**4**_ **]** ^**2+**^.

The above-described behavior is explained by two alternative modes of Fe’s valence inhomogeneity enhancement, assisted by electron density maps in Figure S63. While the **[Fe**_**4**_ **S**_**4**_ **]** ^**2+**^ species contains four essentially equal mixed-valence Fe^2.5+^ sites, its oxidation to **[Fe**_**4**_ **S**_**4**_ **]** ^**3+**^ exchanges one (out of two) mixed-valence Fe^2.5+^Fe^2.5+^ pair to a *di*-ferric Fe^3+^Fe^3+^ pair. This leads to a 1° sphere effect of Fe^2.5+^–S­(thiolate) vs Fe^3+^–S­(thiolate) bonding variation, and hence a broader collective intensity in the Fe–S_HIGH_ region with a flattened peak at ∼400 cm^–1^ (Figure S64, *top left*). Similar redox-dependent dispersions are also produced in the Fe–S_HIGH_ modes of the K^+^-encapsulated systems, **K-[Fe**_**4**_ **S**_**4**_ **]** ^**3+**^ vs **K**_**2**_ **-[Fe**_**4**_ **S**_**4**_ **]** ^**2+**^ (Figure S64, *top right*). The high-end intensities of the Fe–S_HIGH_ region, known as FeS cluster redox state markers,^96,97^ upshift by at most 20 cm^–1^ upon the oxidation. In the Fe–S_LOW_ spectral region, the Fe^2.5+^/Fe^3+^ sites variation leads to a deterioration of the peaks around ∼310 cm^–1^. In contrast, the nonredox addition of the K^+^ ion(s) to the 2° sphere of the cubanes (**[Fe**_**4**_ **S**_**4**_ **]** ^**2+**^ → **K**_**2**_ **-[Fe**_**4**_ **S**_**4**_ **]** ^**2+**^, **[Fe**_**4**_ **S**_**4**_ **]** ^**3+**^ → **K-[Fe**_**4**_ **S**_**4**_ **]** ^**3+**^) earns only small modifications in the Fe–S_HIGH_ intensities (Figure S64, *bottom*). Instead, the cations contribute to the Fe sites dissimilarity via polarization of the electron density across the FeS core (Figure S63), which translates into Fe–S­(μ^3^) intensities adjustment at the low end of the Fe–S_LOW_ region. The latter effect is reflected in the decay of the most prominent peaks around ∼280 cm^–1^ upon K^+^ encapsulation, and redistribution of the intensities to lower vibrational energies around ∼250 cm^–1^ (Figure S64, *bottom*).

Notably, the relative energies of spin isomer (BS-)­states in the 2 × 2 set are only moderately altered by the K^+^ ions encapsulation, as predicted by the “extended” DFT modeling scheme (Table S30). At 50 K, the energies collected in Table S30 translate to at least 98% population of the ground states.

### 2.4 1° Sphere Effects on [Fe_4_S_4_]^3+/2+/1+^ Complexes

As highlighted in the introduction, the redox potential of a Fe_4_S_4_ cofactor and the energetic landscape of its valence isomers is also tuned by noncanonical amino acid ligation.^101^ Though several amino acids and/or other small ligands are known to bind to Fe_4_S_4_ cofactors (including in approximate order of decreasing occurrence histidine, asparagine, *S*-adenosylmethionine and citrate^101–103^ ), by far the most frequently encountered example for this is the replacement of a (anionic) cysteine ligand by a (neutral) histidine. This is, for instance, the case in nitrate reductase,^104^ NifB’s K1 cluster,^105^ some CoA dehydratases,^106^ [NiFe][Bibr ref107] and [FeFe] hydrogenases^108^ (Figure 1A, *left*) or even along the Fe_4_S_4_ chain in respiratory complex I.^109–111^ It has been emphasized that it is important to consider methods operating near ambient temperatures to probe the 1° sphere effect in such noncanonical site-differentiated Fe_4_S_4_ complexes,^112^ given the large expected effect of the 1° sphere on the energetics of valence isomerism when compared to the 2° sphere. Accordingly, in the following sections, we compare how the basic ^57^Fe Mössbauer, EPR, NRVS and UV–vis electronic absorption spectroscopic observables of the 1°-sphere-perturbed cubanes compare to those of their canonical “neat” and 2°-sphere-perturbed counterparts. Overall, we however focus less on the fingerprinting of oxidation-state- and technique-dependent observables; instead reporting the direct determination/estimation of the Δ*E*_VI_-, Δ*E*_redox_- and Δ*E*_vib_-values.

#### 2.4.1 1° Sphere Effects in Models for 3:1 (Cys/His) Ligated [Fe_4_S_4_]^1+^ and [Fe_4_S_4_]^3+^ Clusters

Using ΔΔ*E*_VI_ = Δ*E*_VI_(L = NMI) – Δ*E*_VI_(X = BnS^–^), Suess and colleagues estimated the ground state valence isomer splitting induced by Cys-to-His-substitution in [Fe_4_S_4_(NHC)_3_(L/X)]^+/0^ as ca. −210 cm^–1^ (Figure 9), whereby the more stable isomer favors localization of Fe^2+^ (majority oxidation state) at the unique site.^48,49^

**Figure 9:**
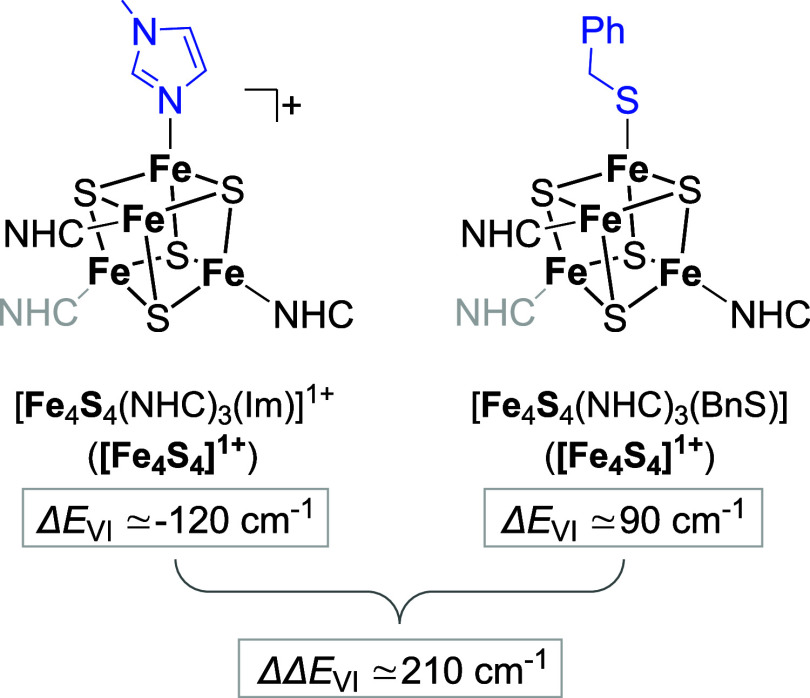
Estimated energetic splitting of the valence isomers in 3:1 (cysteine/histidine) ligated [Fe_4_S_4_]^1+^ clusters recently reported by Skeel and Suess based on ΔΔ*E*_VI_ between two NHC-supported site-differentiated clusters.^48,49^

By analogy, we estimate here Δ*E*_VI_ for [Fe_4_S_4_(DmpS)_3_(Im*)] (**[Fe**_**4**_ **S**_**4**_ **]** ^**3+**^ **-Im**; where Im* = 1,2,4,5-tetramethylimidazole), by evaluating its VT ^1^H NMR spectra and its VT solution-state magnetic moment: **[Fe**_**4**_ **S**_**4**_ **]** ^**3+**^ **-Im**’s mixed thiolate/imidazole ligation gives a direct estimate of the physiologically relevant Δ*E*_VI_ without the need of evaluating a ΔΔ*E*_VI_, and is also comparable to the Δ*E*_VI_ values estimated for K_*n*_[Fe_4_S_4_(DmpS)_4_] (vide supra). The ^1^H chemical shift values vary only marginally with *T* (maximally 0.27 ppm) in [Fe_4_S_4_(DmpS)_4_]^−^, and significantly more for **[Fe**_**4**_ **S**_**4**_ **]** ^**3+**^ **-Im** (up to 4.5 ppm over a range of 80 K) (Figures S37 and S38), mostly displaying Curie-type behavior (Figure S39). This illustrates how the considerations (and formalism) applicable to [Fe_4_S_4_]^1+^ complexes are also valid for the [Fe_4_S_4_]^3+^ oxidation state, because it is also an odd-electron “pair-of-pairs”.^77–79^ The simplified Hamiltonian^44,51,79^ for the two valence isomers (denoted “A” and “B”), used in Suess’s work to model the *T*-evolution of the magnetic parameters, is summarized in eq 2, namely^48,49^

2 $${Ĥ}_{{VI}_{A/B}}=\left[\sum_{1\leq p<q\leq 4}J_{A/B}{\mathrm{S⃗}}_{p}\cdot {\mathrm{S⃗}}_{q}\right]-BV_{34/12}T_{34/12}\pm \frac{J_{A/B}}{n}\left({\mathrm{S⃗}}_{1}\cdot {\mathrm{S⃗}}_{2}\right)\mp \frac{J_{A/B}}{n}\left({\mathrm{S⃗}}_{3}\cdot {\mathrm{S⃗}}_{4}\right)$$

Thereby, *J*_A/B_ denote the valence isomers’ global *J* values, and *B* is the double exchange value, which together parametrize the six Fe–Fe superexchange interactions and the one double exchange interaction. The operator *V* reproduces the correct spin dependencies for the energetic gain of double exchange, whereby *T*_*ij*_ represents the (symmetric) transfer operator for the electron hopping from one of the two mixed-valent sites to the other.^52,113^ It is further assumed that the exchange couplings of the reduced/oxidized Fe_2_S_2_ subunits have a fixed relationship to the global exchange coupling (*J*) via the parameter *n*.^48,49^ The corresponding results of our analysis on [Fe_4_S_4_(DmpS)_3_(Im*)] are compiled in Figure 10A,B, specifically amounting to *J*_A_ = 313 ± 2 cm^–1^, *J*_B_ = 654 ± 90 cm^–1^, *n* = −3.09 ± 0.04 and *B* = 204 ± 6 cm^–1^.

**Figure 10:**
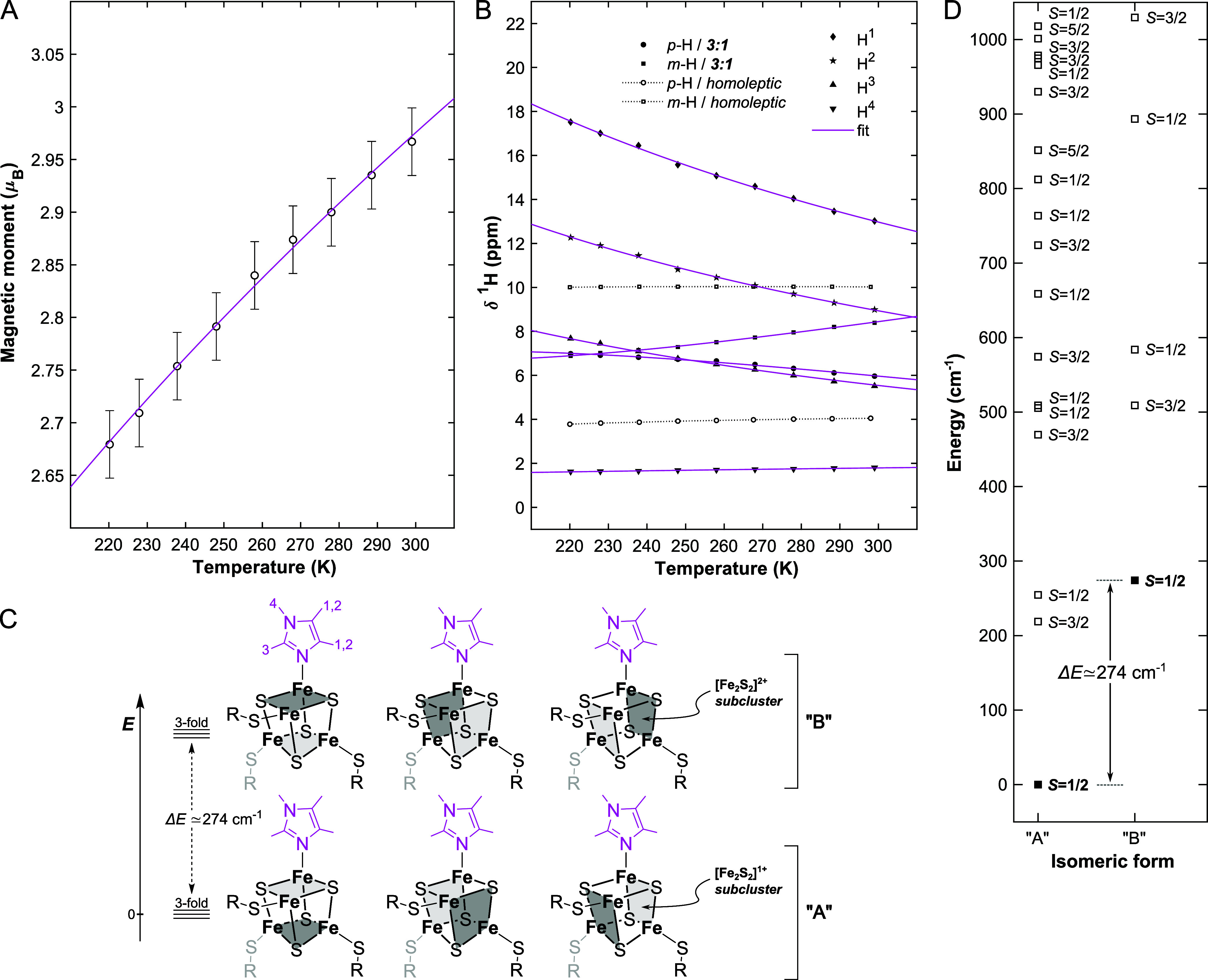
(A) Evans method magnetic moments for **[Fe**_**4**_ **S**_**4**_ **]** ^**3+**^ **-Im** (*dots*) vs temperature and best global fit of the data (*magenta trace*). (B) Plot of the DmpS^–^-ligand’s aryl *para*-H and *meta*-H chemical shifts as well as the Im* methyl group’s protons chemical shifts (*solid black markers*) against temperature. *Magenta traces* indicate the best global fit. For comparison, the variation of the homoleptic system’s DmpS^–^ ligand’s aryl *para*-H and *meta*-H are shown as *hollow black markers*. (C) Schematic depiction of the ground state valence isomeric forms “A” and “B” of **[Fe**_**4**_ **S**_**4**_ **]** ^**3+**^ **-Im** according to the model fitted to the VT ^1^H NMR data. (D) Spin-ladder plots (low-energy region) of the two valence isomers of **[Fe**_**4**_ **S**_**4**_ **]** ^**3+**^ **-Im** for the fitted parameters. The total spin of the system is given alongside each spin state (*squares*), and the ground states are highlighted in *bold*.

These values align well with the *J*-coupling constants proposed by Luchinat and co-workers for [Fe_4_S_4_]^3+^ clusters,^114^ but also with the notion that superexchange increases with oxidation state^81^

$$J\left({\left[{Fe}_{4}S_{4}\right]}^{3+}\right)>J\left({\left[{Fe}_{4}S_{4}\right]}^{2+}\right)>J\left({\left[{Fe}_{4}S_{4}\right]}^{1+}\right)$$ 3

and the fact that double exchange (*B*) decreases upon oxidation, owing to an increase in Fe–S bond covalency, resulting in stronger superexchange and valence localization compared to [Fe_4_S_4_]^2+^ or [Fe_4_S_4_]^1+^.^93^ The associated ground state Δ*E*_VI_ amounts to +274 ± 8 cm^–1^ (Figure 10C)—larger than the value determined by Suess for the [Fe_4_S_4_]^1+^ oxidation state (ca. −210 cm^–1^ based on ΔΔ*E*_VI_ = Δ*E*_VI_(L = NMI) – Δ*E*_VI_(X = BnS^–^)),^48,49^ but in the same order of magnitude. Note that the global simulation converged with an RMSE-value of 0.05, indicating an excellent fit of the model to the data. The corresponding spin-ladder plots of the two valence isomeric forms are summarized in Figure 10D.

Interestingly, the change in the structuring of the valence isomers in **[Fe**_**4**_ **S**_**4**_ **]** ^**3+**^ **-Im** goes in-hand with a change in the UV–vis electronic absorption spectrum of the cubane, if compared to its homoleptic congener (Figure S66B): the maximum of the extinction redshifts by ca. 20 nm, while the spectrum more strongly adopts the overall shape of the all-ferric, [Fe_4_S_4_]^4+^, complex,^16^ albeit at lower extinction.^69^ This observation is in line with the net increased localization of valences in the site-differentiated complex, where most of the Fe sites have higher Fe^3+^ character (average oxidation state effectively Fe^2.83^) than in its canonical congener. Its X-band perpendicular-mode EPR spectrum is distinct as well, exhibiting an axial *S* = 1/2 signal simulated with *g* = (2.107, 2.029, 2.026) and *g*-strains of (0.034, 0.028, 0.014) as its main component (Figure S10).

80 K Mössbauer spectra recorded on powder samples of ^**[2.2.2]**^ **K-[Fe**_**4**_ **S**_**4**_ **]** ^**3+**^ and **[Fe**_**4**_ **S**_**4**_ **]** ^**3+**^ **-Im** compounds are shown in Figure S28. The two doublets can be well simulated assuming two iron sites in a 1:1 ratio. No significant improvement was obtained upon considering a 3:1 ratio for the two iron sites in **[Fe**_**4**_ **S**_**4**_ **]** ^**3+**^ **-Im**. This is a good indication for the fact that the “pair-of-pairs” architecture of the [Fe_4_S_4_]^3+^ cubane is preserved without desymmetrization of the double-exchange interaction within the mixed-valent pair—a notion that is further supported by analysis of the 6 K variable-field spectra, which are very similar to those of the canonical complexes (Figure S34 and Table S15). Upon substitution of one DmpS^–^ ligand by Im*, a significant increase in line width and a slight decrease of the average isomer shift value is observed: 0.38 mm s^–1^ in ^**[2.2.2]**^ **K-[Fe**_**4**_ **S**_**4**_ **]** ^**3+**^ vs 0.37 mm s^–1^ in **[Fe**_**4**_ **S**_**4**_ **]** ^**3+**^ **-Im** (Table S12). Even if this difference is close to the uncertainty, it suggests a slightly increased ferric character of the iron sites upon introduction of the imidazole. This contrasts with the behavior observed for oxidized Fe_2_S_2_ clusters where the presence of one histidine results in a higher isomer shift value in comparison with that of the all-cysteine ferric site.^115^

Altogether, converting the ΔΔ*E*_VI_/Δ*E*_VI_-values to Boltzmann population differences, we report here that, as a consequence of lifting the degeneracy of the “pair-of-pairs”, the probability of localizing the lower valence (Fe^2.5+^) at the uniquely ligated Fe atom in a [Fe_4_S_4_(^Cys^S)_3_(His)]^0^ complex ([Fe_4_S_4_]^3+^) should amount to approximately 79% at ambient *T*. This is in excellent agreement with Suess’s corresponding result on the reduced, [Fe_4_S_4_(^Cys^S)_3_(His)]^2–^ ([Fe_4_S_4_]^1+^), congener, which was determined to be ca. 72%; the lower valence in their case being Fe^2+^.^48,49^ Therefore, the fact that the two methods, i.e. considering ΔΔ*E*_VI_ in the [Fe_4_S_4_(NHC)_3_(L/X)]^+/0^ system or Δ*E*_VI_ in **[Fe**_**4**_ **S**_**4**_ **]** ^**3+**^ **-Im**, give such similar results further highlights that this behavior is intrinsically associated with Cys-to-His ligand substitution.

#### 2.4.2 1° (and 2°) Sphere Effects in [Fe_4_S_4_]^2+^ Clusters in 3:1 and 2:2 (RS^–^/Im*) Symmetry

Because the two mixed-valent [Fe_2_S_2_]^1+^ subunits of [Fe_4_S_4_]^2+^ cubanes are equivalent, 3:1 symmetry should not cause an energetic separation of the valence isomeric states, unless the double-exchange is asymmetric. However, the situation should be different for a 2:2 site-substituted cluster. Although there are to date no reports of bis-histidine ligation on Fe_4_S_4_ cofactors in nature,^101^ we were yet fundamentally intrigued to investigate Δ*E*_redox_, cluster vibrations and potential isomerization patterns of a synthetic model for this scenario, namely [Fe_4_S_4_(DmpS)_2_(Im*)_2_] (**[Fe**_**4**_ **S**_**4**_ **]** ^**2+**^ **-Im**_**2**_).^69^ This approach would enable a systematic comparison to its mono-substituted (3:1) and canonical congeners, ^[2.2.2]/(18‑C‑6)^K­[Fe_4_S_4_(DmpS)_3_(Im*)] (^**[2.2.2]/(18‑C‑6)**^ **K-[Fe**_**4**_ **S**_**4**_ **]** ^**2+**^ **-Im**) and ^**[2.2.2]**^ **K**_**2**_ **-[Fe**_**4**_ **S**_**4**_ **]** ^**2+**^, respectively.^22,69^ In 2:2 symmetry, a two-level system of spin isomers—one nondegenerate level and another doubly degenerate level—could be expected, because the “[12], [34]” arrangement of pairs is inequivalent to the “[13], [24]” arrangement. Furthermore, the mixed-valent subclusters with mixed ligation may have the propensity for valence localization via the mechanism of asymmetric double exchange—a property which, although not described by the Hamiltonian, should manifest in the *T*-dependent behavior of the chemical shifts (refer to the Supporting Information for additional details). While the ground state’s diamagnetism of the [Fe_4_S_4_]^2+^ oxidation state poses a challenge for the evaluation of the magnetic parameters via VT-^1^H NMR/magnetic moment measurements, we did attempt to do so nonetheless, because the dense manifold of paramagnetic excited states^112^ may still be reflected in the room-temperature observable, δ_para_. In fact, we were able to fit models to the experimental data well, yielding similar *J*-values around 300 cm^–1^ (with *B* = 400 cm^–1^ and *n* = −1.25) for both, the 3:1 and the 2:2 (RS^–^/Im*) substituted [Fe_4_S_4_]^2+^ clusters, but we did not find any evidence for asymmetry in the double-exchange based on our modeling efforts (further details and a proposed extended interpretation of our efforts are summarized in the Supporting Information). Notably, however, these results are in fair agreement to those obtained in comparable VT-NMR spectroscopy studies.^113^

This picture is further confirmed by analysis of the [Fe_4_S_4_]^2+^ clusters’ Mössbauer spectra (vide infra), for which it was shown previously that the isomeric shifts are sensitive toward asymmetry in the exchange couplings:^116,117^ The 80 K spectra recorded on powder samples of **K-[Fe**_**4**_ **S**_**4**_ **]** ^**2+**^ **-Im**, ^**[2.2.2]**^ **K-[Fe**_**4**_ **S**_**4**_ **]** ^**2+**^ **-Im** and ^**(18‑C‑6)**^ **K-[Fe**_**4**_ **S**_**4**_ **]** ^**2+**^ **-Im** are displayed in Figure S29. The three doublets are asymmetric, with the broader line being the high-velocity one for **K-[Fe**_**4**_ **S**_**4**_ **]** ^**2+**^ **-Im** and ^**[2.2.2]**^ **K-[Fe**_**4**_ **S**_**4**_ **]** ^**2+**^ **-Im**, and the low-velocity one for ^**(18‑C‑6)**^ **K-[Fe**_**4**_ **S**_**4**_ **]** ^**2+**^ **-Im**. All spectra, including that of **[Fe**_**4**_ **S**_**4**_ **]** ^**2+**^ **-Im**_**2**_, can be fairly reproduced considering two iron sites in a 1:1 ratio (see the parameters listed in Tables S13 and 2). Considering two sites in a 3:1 ratio and three sites in a 2:1:1 ratio did not lead to better simulations. In line with our efforts in modeling the VT-NMR spectroscopy data (see Supporting Information), this supports the fact that the pairwise delocalized magnetic structure of the [Fe_4_S_4_]^2+^ core is preserved, even upon introduction of 1 or 2 imidazole-ligands—a notion also corroborated by the analysis of the respective low- and high-field spectra recorded at 6 K (Figures S31–S33 and Table S14). The 80 K spectrum of ^**[2.2.2]**^ **K-[Fe**_**4**_ **S**_**4**_ **]** ^**2+**^ **-Im** is compared to that of the all-thiolate cluster, namely ^**[2.2.2]**^ **K**_**2**_ **-[Fe**_**4**_ **S**_**4**_ **]** ^**2+**^, and to that of **[Fe**_**4**_ **S**_**4**_ **]** ^**2+**^ **-Im**_**2**_ in Figure 11.

**Figure 11:**
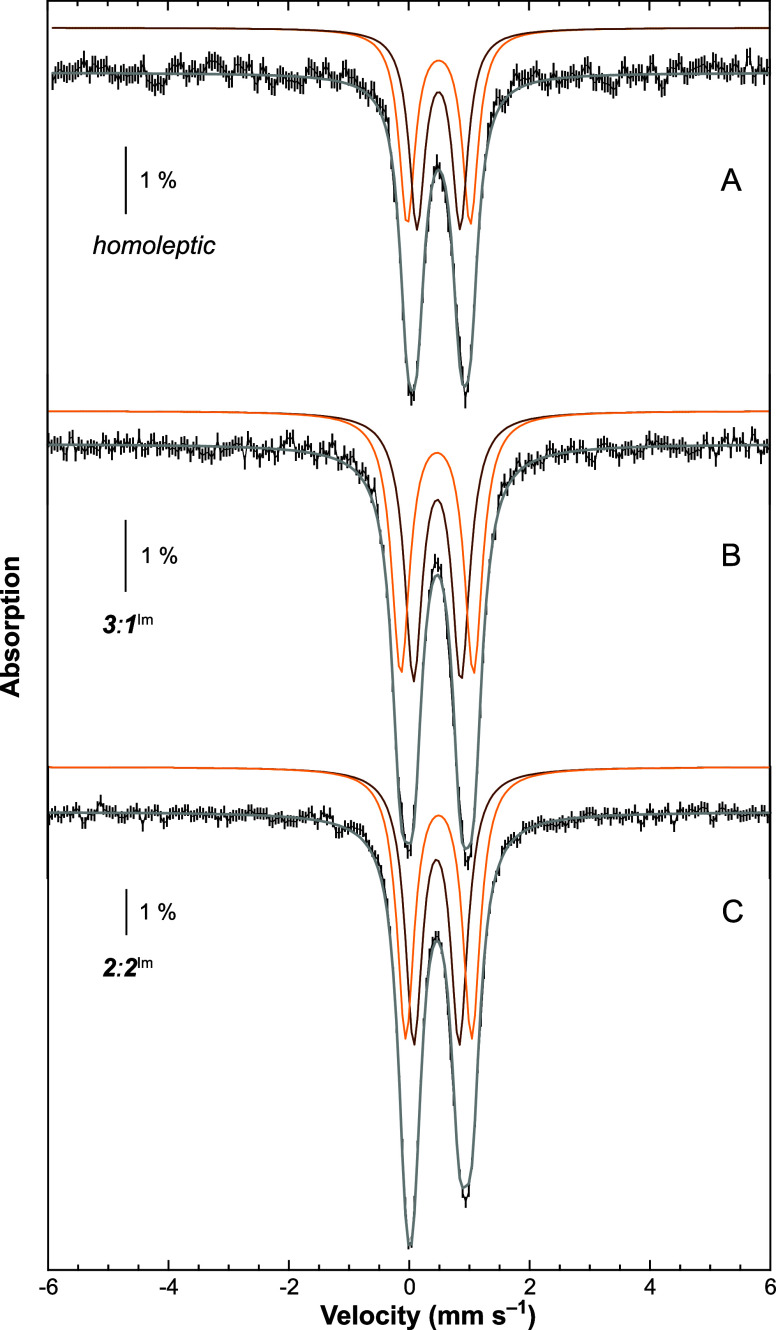
80 K experimental spectra (*black vertical bars*) recorded on a powder sample of ^**[2.2.2]**^ **K**_**2**_ **-[Fe**_**4**_ **S**_**4**_ **]** ^**2+**^ (A), ^**[2.2.2]**^ **K-[Fe**_**4**_ **S**_**4**_ **]** ^**2+**^ **-Im** (B) and **[Fe**_**4**_ **S**_**4**_ **]** ^**2+**^ **-Im**_**2**_ (C) at zero-field (B,C) and upon applying a 0.06 T external magnetic field along the γ-ray’s direction (A). Simulations considering two equally contributing doublets are overlaid as *thick solid gray lines*. Contributions are displayed above as *thin solid lines*. Parameters are listed in Table 2.

Our analysis indicates that substituting one thiolate by one imidazole does not lead to a significant change of the average or individual isomer shift values. At 6 K both species, ^**[2.2.2]**^ **K**_**2**_ **-[Fe**_**4**_ **S**_**4**_ **]** ^**2+**^ and ^**[2.2.2]**^ **K-[Fe**_**4**_ **S**_**4**_ **]** ^**2+**^ **-Im**, exhibit doublets centered at 0.49 mm s^–1^ (Table 2). This is in line with the previous lack of difference observed between the His-coordinated [Fe_4_S_4_]^2+^ cluster and the canonical [Fe_4_S_4_]^2+^ cluster of HydF,^118^ but it is worth to point out that the second substitution leads to a small change (average δ of 0.48 mm s^–1^ for **[Fe**_**4**_ **S**_**4**_ **]** ^**2+**^ **-Im**_**2**_; Table 2). At 80 K, however, all clusters present an average δ of 0.47 mm s^–1^. The tentative value of Δδ, i.e. the difference between the isomer shifts of the two sites, is zero in ^**[2.2.2]**^ **K**_**2**_ **-[Fe**_**4**_ **S**_**4**_ **]** ^**2+**^, and increases from 0.01 mm s^–1^ in ^**[2.2.2]**^ **K-[Fe**_**4**_ **S**_**4**_ **]** ^**2+**^ **-Im** to 0.04 mm s^–1^ in **[Fe**_**4**_ **S**_**4**_ **]** ^**2+**^ **-Im**_**2**_. As such, Δδ remains very small (a Δδ of 0.075 mm s^–1^ should be anticipated for a change in average Fe-oxidation state of 0.25),^16^ and we thus infer that there is negligible asymmetry of the double exchange in the ground states for both the 3:1 and the 2:2 site-differentiated clusters, and—if any at all—only negligible “pair-of-pairs” isomerization; the latter likely being in the same order of magnitude as that of **K**_**2**_ **-[Fe**_**4**_ **S**_**4**_ **]** ^**2+**^ (vide supra).

**Table 2:** Parameters Associated with the Simulations Displayed in Figures S15D–F and S28–S30, Which Correspond to the 6 K Variable-Field Spectra of the THF Solution of ^**[2.2.2]**^ **K**_**2**_ **-[Fe**_**4**_ **S**_**4**_ **]** ^**2+**^ and the Powders of ^**[2.2.2]**^ **K-[Fe**_**4**_ **S**_**4**_ **]** ^**2+**^ **-Im** and **[Fe**_**4**_ **S**_**4**_ **]** ^**2+**^ **-Im**_**2**_ ^a^

	^**[2.2.2]**^ **K**_**2**_ **-[Fe**_**4**_ **S**_**4**_ **]** ^**2+**^		^**[2.2.2]**^ **K-[Fe**_**4**_ **S**_**4**_ **]** ^**2+**^ **-Im**		**[Fe**_**4**_ **S**_**4**_ **]** ^**2+**^ **-Im**_**2**_	
	site 1 (yellow)	site 2 (brown)	site 1 (yellow)	site 2 (brown)	site 1 (yellow)	site 2 (brown)
δ (mm s^–1^)	0.49 (0.47)	0.49 (0.47)	0.48 (0.47)	0.49 (0.48)	0.50 (0.49)	0.45 (0.45)
Δ*E*_Q_ (mm s^–1^)	1.29 (1.02)	0.98 (0.62)	1.34 (1.21)	1.93 (0.79)	1.10 (1.10)	0.80 (0.76)
η	0.4 (−)	0.6 (−)	0.5 (−)	0.6 (−)	0.5 (−)	0.9 (−)
Γ_fwhm_ (mm s^–1^)	0.29 (0.32)	0.29 (0.32)	0.33 (0.36)	0.33 (0.36)	0.29 (0.35)	0.29 (0.35)
av. (δ) (mm s^–1^)	0.49 (0.47)		0.49 (0.47)		0.48 (0.47)	

a A single line width was considered for the two equally contributing doublets. The values given in parentheses are those simulated for the respective 80 K spectra (Figure 11).

From a more phenomenological perspective, this is also illustrated by the fact that very similar behaviors are evidenced in the *T*-dependent zero- or low-field ^57^Fe Mössbauer spectra of the Im*-substituted [Fe_4_S_4_]^2+^ clusters as those observed for their corresponding K^+^-containing and K^+^-free canonical congeners (Figure 4): The spectra of the two powders with chelated K^+^ (^**[2.2.2]**^ **K-[Fe**_**4**_ **S**_**4**_ **]** ^**2+**^ **-Im** and ^**[18‑C‑6]**^ **K-[Fe**_**4**_ **S**_**4**_ **]** ^**2+**^ **-Im**) behave the same. At 5.7 K and low-field, the doublets present low- and high-velocity lines of the same height (very symmetric doublets; Figure S30B,C). The doublet is however clearly not symmetric for the cation-encapsulating variant, **K-[Fe**_**4**_ **S**_**4**_ **]** ^**2+**^ **-Im** (Figure S30A), and for the 2:2 substituted complex (Figure S30D). Moreover, the low-field spectra at 6 and 80 K are similar for the K^+^-containing cluster and **[Fe**_**4**_ **S**_**4**_ **]** ^**2+**^ **-Im**_**2**_ (Figure S30A,D), whereas shifts are detected at 80 K (versus 5.7 K) when K^+^ is chelated (Figure S30B,C).

These patterns are identical to those observed for the powders and solutions of the ^**[2.2.2]**^ **K**_**2**_ **-[Fe**_**4**_ **S**_**4**_ **]** ^**2+**^/**K**_**2**_ **-[Fe**_**4**_ **S**_**4**_ **]** ^**2+**^ clusters described above, and support the notion that similar factors are at play here. In fact, while there is no conceptual possibility of symmetrically distinct spin isomerization in ^**[2.2.2]**^ **K-[Fe**_**4**_ **S**_**4**_ **]** ^**2+**^ **-Im** and ^**[18‑C‑6]**^ **K-[Fe**_**4**_ **S**_**4**_ **]** ^**2+**^ **-Im** (Figure 12A), inequivalent isomers potentially arise in **K-[Fe**_**4**_ **S**_**4**_ **]** ^**2+**^ **-Im** and **[Fe**_**4**_ **S**_**4**_ **]** ^**2+**^ **-Im**_**2**_, upon coupling the “pair-of-pairs” symmetry to the (electrostatic and structural) symmetry of the complex: 2:2 ligands in the latter and 3:1 ligands plus a positive point charge in the former (Figure 12B).

**Figure 12:**
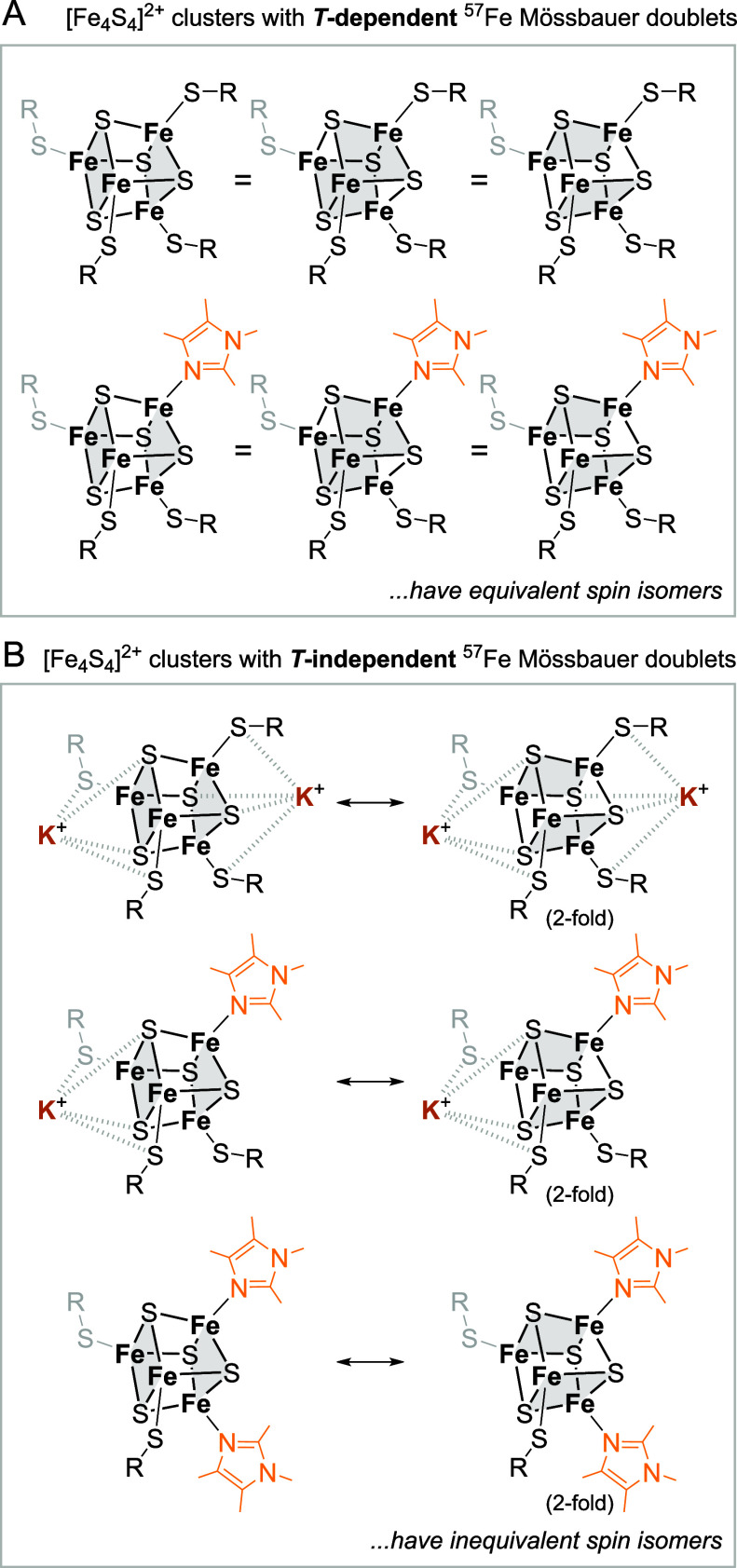
Schematic overview of the [Fe_4_S_4_]^2+^ complexes’ structures and their spin isomeric forms, classified by displaying *T*-dependent (A) and *T*-independent (B) ^57^Fe Mössbauer doublets in their zero-/low-field spectra. For clarity, the cluster charges were omitted.

This observation allows to invoke the fact that all the herein investigated [Fe_4_S_4_]^2+^ complexes, which have symmetry-inequivalent arrangements of the “pairs-of-pairs” displayed *T*-independent ^57^Fe Mössbauer doublets in their zero- or low-field spectra, whereas those with degenerate spin isomers displayed doublets that varied with *T* (Figure 12). Because all of these [Fe_4_S_4_]^2+^ complexes showed very similar spectra overall, the Mössbauer spectra of [Fe_4_S_4_]^2+^ clusters are clearly not useful to identify noncanonical (weak-field) ligands. On the contrary, however, the fact that these spectra do not appear particularly sensitive to the 1° sphere also leads us to suggest that the *T*-dependence of the doublet signal could be a viable probe for the assessment of the 2° sphere’s asymmetry. This should hold true independent of a cluster’s (canonical or noncanonical) 1° sphere as long as its pairwise delocalized electronic structure is maintained, and it possesses no more than 1 noncanonical ligand, which is the case for most native Fe_4_S_4_ cofactors.^101^

As for the [Fe_4_S_4_]^3+^ system, site-differentiation induces significant changes in the UV–vis electronic absorption spectra of the [Fe_4_S_4_]^2+^ cubanes:^69^ upon sequential substitution, the net extinction of the spectrum decreases as it concomitantly becomes broader and absorbs over a wider energy range (Figure S66A). In contrast to the oxidized cluster, however, the extinction maxima of the site-differentiated [Fe_4_S_4_]^2+^ complexes are not energetically shifted. This further underscores that the electronic structure of [Fe_4_S_4_]^2+^ is not influenced in the sense that the effective oxidation states of the individual Fe atoms are affected—as it occurs for the odd-electron [Fe_4_S_4_]^1+/3+^ complexes. Instead, if anything, only the spin isomerism between the different “pairs-of-pairs” is perturbed.

Beyond electronic/magnetic structure, Δ*E*_redox_ is readily accessible from cyclic voltammetry experiments. As reported previously, compared to the homoleptic complex, site-substitution causes drastic changes in the voltammogram,^69^ suggesting that complex chemical processes ensue upon redox reactions for both for the 3:1, as well as the 2:2 site-differentiated cubanes, but those were the topic of another work.^69^ The positions of the main redox events can be used to determine Δ*E*_redox_ as a function of the number of unique ligands bound to the cubane. We observed that the relation between the number of Im* ligands and the redox potential is linear (Figure 13), and nearly identical for both redox couples ([Fe_4_S_4_]^1+/2+^ and [Fe_4_S_4_]^2+/3+^), resulting in an increase of ca. 450 mV for each additional Im*. This value is surprisingly congruent with the Δ*E*_redox_ we inferred for encapsulation of a single cation into the [Fe_4_S_4_(DmpS)_4_]^*n*−^ assembly, being also ca. 450 mV.^22^

**Figure 13:**
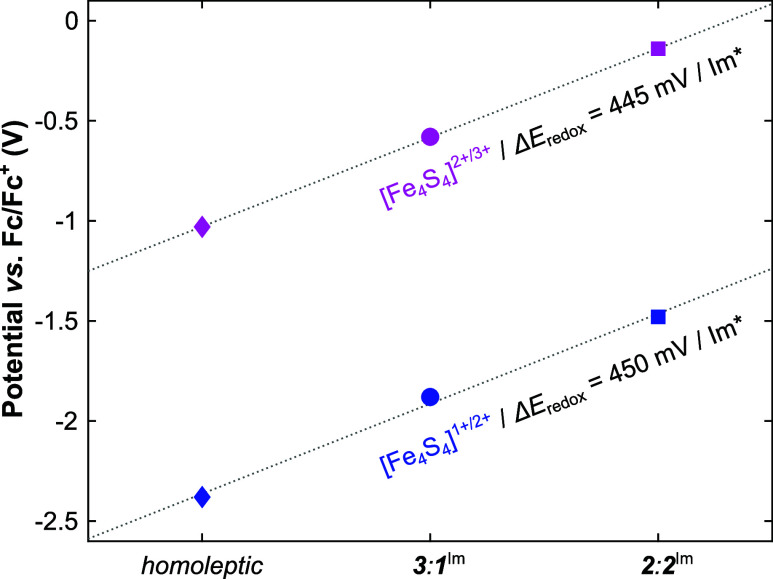
Correlation of the number of Im* ligands on the Fe_4_S_4_ cubane with the complex’s redox potential in the [Fe_4_S_4_]^1+/2+^ (*blue*) and [Fe_4_S_4_]^2+/3+^ (*magenta*) oxidation states vs Fc/Fc^+^: [Fe_4_S_4_(DmpS)_4_] (*homoleptic*; 0 Im*; *diamonds*), **[Fe**_**4**_ **S**_**4**_ **]** ^**3+**^ **-Im** (***3:1*** ^Im^; 1 Im*; *dots*) and **[Fe**_**4**_ **S**_**4**_ **]** ^**2+**^ **-Im**_**2**_ (***2:2*** ^Im^; 2 Im*; *squares*).

### 2.5 1° Sphere Imidazole Ligation and Cluster Vibrations

We suspected that, similar to the interaction with K^+^-ions, site-differentiation would also cause subtle changes to the Fe_4_S_4_ cluster vibrations. To this end, the family of 2:2 and 3:1 site-differentiated [Fe_4_S_4_]^2+^ and [Fe_4_S_4_]^3+^ clusters was analogously analyzed by ^57^Fe NRVS spectroscopy. As for the canonical cubanes, the ^57^Fe PVDOS spectra of the site-differentiated ones (Figure S65, *left*) exhibit the typical vibrational structure of Fe_4_S_4_ complexes,^96–98^ whereby the main difference upon Im* ligation is the emergence of an additional band around 240 cm^–1^. This band is characteristic for Fe–N­(imidazole) stretching, and has been observed in enzymatic systems containing [Fe_2_S_2_(^Cys^S)_3_(His)]-type cofactors.^119^ Parallel to our analysis of the K^+^/^[2.2.2]^K^+^-salts of the cubanes (vide supra), inspection of the spectra and the difference spectra reveals that several of the peaks split or shift upon Im* substitution. Multiple features are reminiscent of bathochromic shifts (Figure S65, *right*), which are marginally larger than those observed for K^+^-ion encapsulation, Δ*E*_vib_ lying between 13 and 26 cm^–1^, but they are in the same order of magnitude.

In analogy to our observations in the spectra of the canonical cubanes, the most changes to the Fe–S modes’ degeneracy appear in the Fe–S_LOW_ region of the spectra (∼230–340 cm^–1^). However, instead of the band at 280 cm^–1^, as it was the case for K^+^-ion encapsulation, here, substitution causes the higher-energy band of the two peaks in the Fe–S_LOW_ region (around 310–320 cm^–1^) to deteriorate, redistributing intensity to higher and lower wavenumbers, and visibly forming a shoulder at about 330 cm^–1^. Furthermore, 1° sphere ligation causes no broadening of the vibrations in the Fe–S_HIGH_ region of the spectra. Still, a common trend for all substituted clusters is a net blueshift of these modes (∼380–410 cm^–1^), namely by up to 10 cm^–1^ for 2:2 substitution, when they are compared to their canonical counterparts.

### 2.6 Beyond Redox Potential and Electronic Structure: Estimating 1° vs 2° Sphere Effects on Electron Self-Exchange Rates

Redox potential is central, but not sufficient, to discuss the ET properties of the clusters. According to Marcus theory, ET rates are also influenced by the reorganization energy, λ, and the electronic coupling between the initial (A) and final (B) states, *H*_AB_.^120^ These parameters can be probed from ETse rates, where ET should occur with no redox driving force (i.e. Δ*G* ^0^ = 0), as schematically illustrated in Figure 14. The associated ETse rates, *k*_se_, are provided by eq 4 ^1,2^

4 $$k_{se}=\frac{2\pi }{\hbar }\frac{{\left|H_{AB}\right|}^{2}}{\sqrt{4\pi \lambda k_{B}T}}\;e^{\frac{-\lambda^{2}}{4\lambda k_{B}T}}$$

whereby *k*_B_ is the Boltzmann constant, *ℏ* is the reduced Planck constant, and *k*_se_ thus depends only on *T*, λ and *H*_AB_.

**Figure 14:**
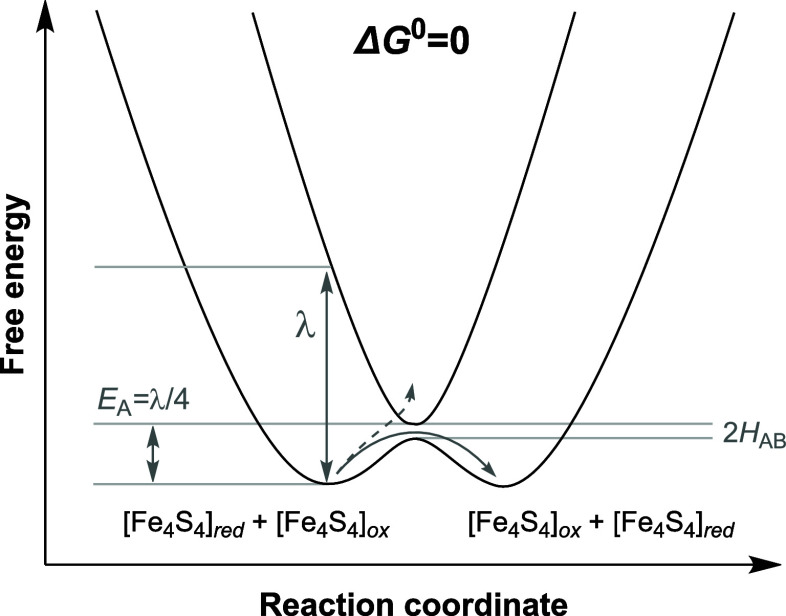
Marcus theory description of a nonadiabatic ET with Δ*G* ^0^ = 0 between two isosteric iron–sulfur clusters of differing oxidation states.

ETse kinetics are commonly studied by NMR spectroscopy line width methods.^121–124^ However, while a significant quantitative analysis of the *k*_se_-values of our systems proved unfeasible, the phenomenological relationship between *k*_se_, and the frequency difference between the two exchanging species’ NMR signals, Δν,^123,124^ nevertheless allowed us to delineate qualitative differences and estimates for the ETse kinetics of our model systems, encompassing ^**[2.2.2]**^ **K**_***i***_ **-[Fe**_**4**_ **S**_**4**_ **]** ^**2+/3+**^, **K**_***i***_ **-[Fe**_**4**_ **S**_**4**_ **]** ^**2+/3+**^ (where ***i*** = 2, 1) and ^**[2.2.2]**^ **K**_***j***_ **-[Fe**_**4**_ **S**_**4**_ **]** ^**2+/3+**^ **-Im** (where ***j*** = 1, 0) (refer to the Supporting Information for further details):

For **K**_***i***_ **-[Fe**_**4**_ **S**_**4**_ **]** ^**2+/3+**^ (where ***i*** = 2, 1), the line shapes exhibit no evidence for exchange phenomena: all NMR signals of the redox mixture bear their native positions and widths (Figure S50). This suggests that *k*_se_(**K**_**2**_ **-[Fe**_**4**_ **S**_**4**_ **]** ^**2+**^/**K-[Fe**_**4**_ **S**_**4**_ **]** ^**3+**^) is either very small (*k*_se_ ≪ Δν; here *k*_se_ ≪ 10^2^ s^–1^ for the smallest Δν), or ETse is entirely inhibited, because it must be coupled with the transfer of a K^+^ ion, which, in turn, renders Δ*G* ^0^ ≠ 0.

In contrast, for ^**[2.2.2]**^ **K**_***i***_ **-[Fe**_**4**_ **S**_**4**_ **]** ^**2+/3+**^ (where ***i*** = 2, 1), all resonances observed for the redox mixture are exchange broadened (Figure S51) and the *m*-H­(Mes) resonances (Δν = 40 Hz) coalesce around 290–300 K (Figure S52). We thus infer that *k*_se_ lies somewhere between 40 and 1000 s^–1^ (for the smallest and largest Δν’s, respectively), though the observed coalescence of the signals separated by 40 Hz supports the notion that the true *k*_se_ is found at the lower end of this range.

Similarly, for ^**[2.2.2]**^ **K**_***j***_ **-[Fe**_**4**_ **S**_**4**_ **]** ^**2+/3+**^ **-Im** (where ***j*** = 1, 0), all peaks of the redox mixture are broadened by exchange—some of them even to the degree of being undetectable (Figure S53). Based on the Δν’s, *k*_se_ should be ≫300 s^–1^, but ≤5000 s^–1^, which is slightly faster than the *k*_se_ for the homoleptic system. These proposed ranges of *k*_se_ are summarized in Figure 15. Note that all of these estimated rates are somewhat slower than those reported for [Fe_4_S_4_(TolS)_4_]^2–/3–^ ^125^ and HiPIPs^126^—a possible consequence of the large organic DmpS^–^-ligands shielding the cluster core.

**Figure 15:**
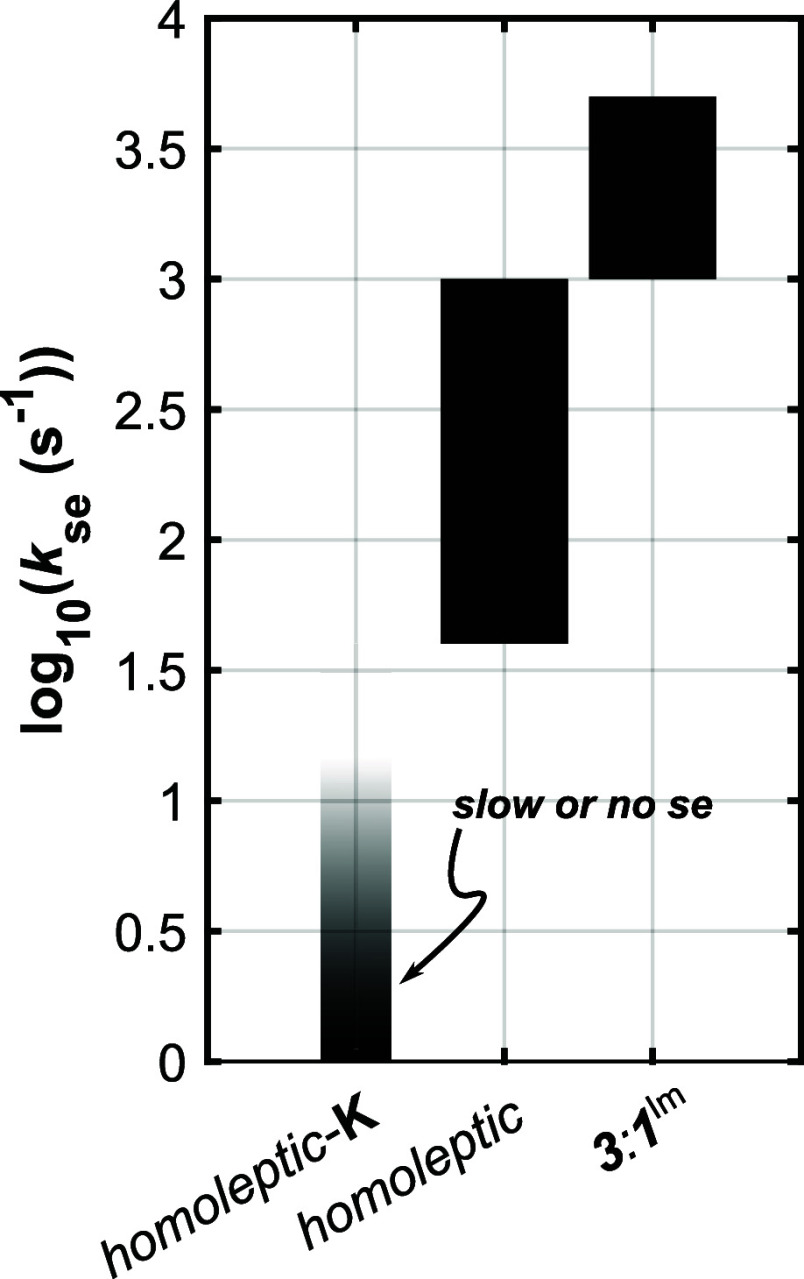
Summary of the estimated ETse rates (*black bars*) based on the phenomenological appearance of the NMR spectra of the redox mixtures (*left*) ^**[2.2.2]**^ **K**_***i***_ **-[Fe**_**4**_ **S**_**4**_ **]** ^**2+/3+**^, (*middle*) **K**_***i***_ **-[Fe**_**4**_ **S**_**4**_ **]** ^**2+/3+**^ (where ***i*** = 2, 1) and (*right*) ^**[2.2.2]**^ **K**_***j***_ **-[Fe**_**4**_ **S**_**4**_ **]** ^**2+/3+**^ **-Im** (where ***j*** = 1, 0; see Figures S50–S53).

While the kinetics of encapsulating (a different number of) K^+^ ions into the structure therefore appears to interfere with (and shut-down) ETse, a significant difference between the exchange rates of the homoleptic (or canonical) [Fe_4_S_4_]^2+/3+^ complex and its 3:1 site-differentiated congener is observed. As Δ*G* ^0^ = 0 in both cases, this difference should arise due to changes in λ and *H*_AB_, constituting initial evidence for a possible connection between the cluster’s structure and charge, its excited states and its Marcus parameters.^120^ Efforts to better understand this complex relationship are ongoing in our group.

## 3 Discussion

Disentangling the different effects that can alter the spectroscopic signatures and the energetic landscape of Fe_4_S_4_ cofactors is challenging, particularly due to the variety of mutually interfering 1° and 2° sphere effects (Figure 1B), the elusive nature of electric field interactions,^32^ but also the clusters’ inherently complex electronic and magnetic structures. However, the rigorous characterization of our well-defined molecular models, encompassing “neat” ^[2.2.2]^K_*m*_[Fe_4_S_4_(DmpS)_4_] (*m* = 1–2), electric field containing K_*x*_[Fe_4_S_4_(DmpS)_4_] (*x* = 1–3), and site-differentiated [Fe_4_S_4_(DmpS)_4–*y*_(Im*)_*y*_] (*y* = 1, 2), should contribute to a robust understanding of the energetics of two of the tuning mechanisms for Fe_4_S_4_ cofactors—one being the electrostatics of the 2° sphere, and the other being the ligation in the 1° sphere—and to our ability to better identify them in the first place.

Here, the spectroscopic investigations on the K_*x*_[Fe_4_S_4_(DmpS)_4_] (*x* = 1–3) complexes enabled estimating the absolute order of magnitude by which valence isomers are energetically separated by electrostatics. While these values are associated with uncertainties for which we cannot sufficiently account for based on our methodology,—including electron spin relaxation behavior, thermal EPR signal responsivity, cryostat inaccuracy, etc.—the qualitative attributes of the experimental data and our analyses allow us to constrain the extent of the electric field effect to approximately Δ*E*_VI_ = 0–10 cm^–1^ (Figure 16B, *right*). We propose the upper bound as the highest energy difference, for which we have experimental evidence, namely the signal interconversion in the 4.5–30 K VT X-band EPR spectra of **K**_**3**_ **-[Fe**_**4**_ **S**_**4**_ **]** ^**1+**^ in the presence of 4 equiv of 18-crown-6. However, it should be noted that, despite the limited magnitude of this energy difference, we observed a 2° sphere electric field effect on the spectroscopic properties of the Fe_4_S_4_ clusters in all three investigated oxidation states of the cubane. In fact, the VT EPR and ^57^Fe Mössbauer experiments on **K-[Fe**_**4**_ **S**_**4**_ **]** ^**3+**^ and **K**_**2**_ **-[Fe**_**4**_ **S**_**4**_ **]** ^**2+**^, respectively, hint toward the fact that Δ*E*_VI_ may also be even smaller, in an order of magnitude ≤6 *k*_B_ (≤4 cm^–1^). In addition, VT-NMR spectroscopy allowed us to measure Δ*E*_VI_ of **[Fe**_**4**_ **S**_**4**_ **]** ^**3+**^ **-Im** in more detail, including its sign, and to recognize that it is directly comparable to Suess’ results on reduced,^48,49^ [Fe_4_S_4_]^1+^, complexes. However, this is because, compared to the 2° sphere, Δ*E*_VI_-values of the odd-electron systems perturbed in the 1° sphere can be assumed to be much larger, as the magnetic “pair-of-pairs” symmetry of the cubane couples to the molecular *C*_3*v*_ symmetry through covalent interactions.

**Figure 16:**
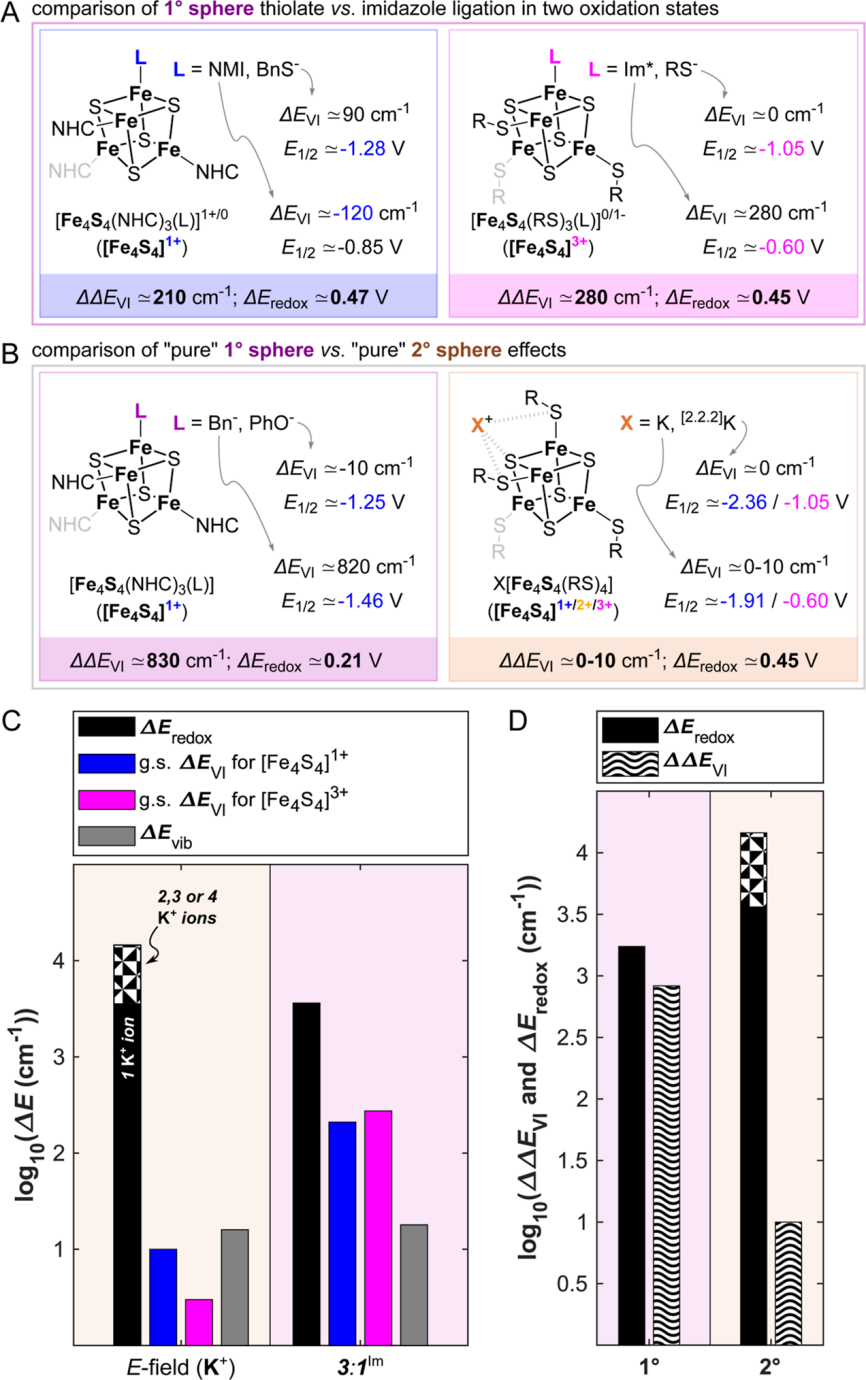
(A) Schematic representation of the molecular structures and associated Δ*E*_VI_ and *E*_1/2_-values for the clusters considered for the evaluation of the effect of noncanonical ligation by a histidine- or imidazole-type ligand. They include those reported by Suess and Skeel for the [Fe_4_S_4_]^1+^ oxidation state,^48,49^ and the systems discussed in this work, for the [Fe_4_S_4_]^3+^ oxidation state. For clarity, cluster charges were omitted in the structures but are indicated in the sum-formulas, which are shown alongside. (B) Schematic representations of the molecular structures and their associated Δ*E*_VI_ and Δ*E*_redox_-values for the clusters considered for the evaluation of the “pure” 1° and 2° sphere effects; i.e. without considering a change in the net charge of the cluster by a 1° sphere manipulation. These include the two neutral clusters with most different *E*_1/2_ from Suess’ work,^48,49^ and the cation-encapsulated/cation-sequestered clusters analyzed here. In both panels (A,B), relevant ΔΔ*E*_VI_- and Δ*E*_redox_-values are indicated alongside in bold font. (C) Bar-plot for the absolute magnitudes of various log_10_(Δ*E*)-values for the 2° sphere electrostatics of a single encapsulated K^+^ ion (*left*) and 1° sphere 3:1 (thiolate/imidazole) site-differentiation (*right*) in the [Fe_4_S_4_]^1+^ (*blue*) and [Fe_4_S_4_]^3+^ (*magenta*) oxidation states.^48,49^ The *mosaic area* of the bar includes the range relevant for encapsulation of a 2nd, 3rd and 4th K^+^ ion. (D) Bar-plot for the absolute magnitudes of ΔΔ*E*_VI_- and Δ*E*_redox_-values for the 1° and 2° sphere perturbed complexes summarized in panel (B).

Figure 16A,C summarize the model complexes’ structures and associated magnitudes of Δ*E*_redox_ for encapsulation of a single K^+^ ion,^22^ the (absolute) Δ*E*_VI_-values for 3:1 site substitution by imidazole derivatives (NMI for [Fe_4_S_4_]^1+^ and Im* for [Fe_4_S_4_]^3+^), for the two “active” redox states of Fe_4_S_4_ cofactors,^48,49,70^ and the respective largest observed Δ*E*_vib_ (Figure 16C).

A single K^+^ cation’s electric field or thiolate-to-imidazol substitution both cause a Δ*E*_redox_ of approximately +400–450 mV (ca. +3200–3600 cm^–1^ for one-electron transfer).^22,70^ If more than one K^+^ ion—or more than one charge, respectively—interacts with the cluster, the electrostatically caused Δ*E*_redox_ can even be much larger (*checkered bar* in Figure 16C; up to ca. +1.8 V or +14,500 cm^–1^ for four K^+^ ions^22^). Together, however, the similarity of Δ*E*_redox_^(1°)^ and Δ*E*_redox_^(2°)^ underlines the fact that the net charge of the molecular cofactor assembly is the dominant factor influencing its redox potential, and not so much the exact identity of the cluster’s (weak field) ligands. In contrast, VT-NMR spectroscopy studies corroborated the fact that 3:1 thiolate-to-imidazole ligation in the 1° sphere splits the energetic landscape of the valence isomers one to 2 orders of magnitude more strongly than the 2° sphere electrostatics. Our investigations on the corresponding “resting” oxidation state of the cofactors, namely [Fe_4_S_4_]^2+^, lead us to suggest that such valence isomerism is restricted to the “active” oxidation states of the cubane, [Fe_4_S_4_]^1+^ and [Fe_4_S_4_]^3+^, respectively. After all, the absence of significant variations in the ^57^Fe Mössbauer isomeric shifts of the Fe-sites in the canonical, 3:1 and 2:2 site-differentiated [Fe_4_S_4_]^2+^ cubanes implies that there is likely no asymmetry of the double exchange within their heteroligated Fe_2_S_2_ subunits. Nevertheless, even noncanonical [Fe_4_S_4_(^Cys^S)_3_(His)]^−^ cofactors may demonstrate manifestations of “spin isomerism” in their *T*-dependent ^57^Fe Mössbauer spectra, as a consequence of (electrostatic) desymmetrization of their 1° or 2° spheres, when carefully analyzed (Figures 4 and S27).

In this context, the ^57^Fe PVDOS (NRVS) vibrational investigations revealed that both, 1° and 2° sphere effects, have an influence on the cluster vibrations (Δ*E*_vib_). However, the differences between them are small: ca. 13–26 cm^–1^ for the 1° sphere effects and ca. 9–23 cm^–1^ for the 2° sphere effects. While Δ*E*_vib_^(1°)^ is thus an order of magnitude smaller than Δ*E*_VI_^(1°)^, it is in the same order of magnitude as Δ*E*_VI_^(2°)^ (Δ*E*_VI_^(2°)^ ≃ Δ*E*_vib_^(2°)^). It could therefore be possible that the encapsulation of K^+^ ions, much like the interaction of a cofactor-bound ^Cys^S residue with a N–H moiety of the backbone or H_2_O,^127,128^ leads to desymmetrization of the cluster vibrations. In the present case, this manifested specifically as a red-shift of the low-end Fe–S bands around ∼250 cm^–1^ (Figure S64, *bottom*). In turn, and in addition to the purely electrostatic arguments, these perturbed Fe–S vibrations may promote the trapping of valence isomers on the potential energy surface at cryogenic temperatures. However, the exact origin of this valence isomer trapping in the K^+^-ion-encapsulating Fe_4_S_4_ complexes, be it vibrational or electrostatic, at this point appears to be a “chicken-and-egg” dilemma.

A limit of our approach is reached, when attempting to evaluate the 1° sphere covalent effect in a more “pure” form, i.e. without being affected by an alteration of the charge-state of the complex, as it is the case for thiolate-to-imidazole substitution. However, further contextualizing our work with that of Suess allows^48,49^ to do so: benzyl-to-phenoxide substitution in the [Fe_4_S_4_(NHC)_3_(X)]-system (X = Bz^–^, PhO^–^) tunes the electronic structure of the cubane strongly, but rather minorly affects the *E*_1/2_-values (Figure 16B, *left*). Specifically, the ground-state valence isomeric forms are split by as much as 830 cm^–1^, while the redox potential of the cluster is altered by a mere 210 mV (1690 cm^–1^). These ΔΔ*E*_VI_- and Δ*E*_redox_-values are thus approaching the same order of magnitude (10^3^ cm^–1^; Figure 16B,D). Compared to the 2° sphere electrostatics, however, ΔΔ*E*_VI_^(1°)^ ≫ ΔΔ*E*_VI_^(2°)^ and Δ*E*_redox_^(1°)^ < Δ*E*_redox_^(2°)^, illustrating how local 2° sphere electric dipolar interactions can tune the redox potentials of the clusters much more efficiently and delicately than the 1° sphere, because they enable significant *E*_1/2_-variations without electronic restructuring of the “active” (odd-electron) forms of the clusters (ΔΔ*E*_VI_^(2°)^ ≪ Δ*E*_redox_^(2°)^ but ΔΔ*E*_VI_^(1°)^ ≤ Δ*E*_redox_^(1°)^).

Altogether, these results lead us to hypothesize that the main distinction between the 1° and 2° sphere mechanisms of tuning the cofactor lies in their relevance at ambient temperature, as well as the extent to which they influence Δ*E*_redox_-, Δ*E*_vib_- and Δ*E*_VI_-values differently, particularly for the cofactors in their odd-electron oxidation states. Among the three investigated Δ*E*-values, and for the most common type of noncanonical ligation (imidazole-type; Figure 16A,C) only Δ*E*_VI_ is significantly different for the 1° (imidazole) and 2° sphere effects, whereby the difference appears larger for the [Fe_4_S_4_]^3+^ than for the [Fe_4_S_4_]^1+^ complex. Regarding electron transfer, Δ*E*_VI_ is equivalent to the difference between the reduction potentials of the individual valence isomeric forms of the cubane. Thus, while 2° sphere interactions do not create a redox potential gradient within the Fe_4_S_4_ clusters core, altering the relative electrochemical potentials of the isomers by a mere 0.5–1.5 mV, 1° ligation causes them to be split by ca. 25–40 mV; an amount we think is large enough so that it may influence at which spatial orientation of the Fe_4_S_4_ complex the electron is preferably released or taken-up, respectively. Notably, this effect is also often described as “control over the size, shape and localization of electrons/holes or the redox-active molecular orbital (RAMO)”.^48,49,129–131^ Such a delicate regulation of ETs, combined with tuning of the excited states, is likely to influence electron tunneling rates, or the interaction between cofactor and substrate. After all, the magnetic and electronic properties of a specific valence isomer may be altered by a noncanonical ligand such that the electronic coupling between the wave functions of the cofactor and the substrate is maximized or diminished, respectively. In contrast, the electrostatic component of 2° sphere interactions falls short of such delicate tuning. They affect the net redox potential of the cofactor in a similar manner (i.e. Δ*E*_redox_^(1°)^ ≃ Δ*E*_redox_^(2°)^, namely 10^3^–10^4^ cm^–1^), but they do not tune the electronic/magnetic properties of the Fe_4_S_4_ complex to the same degree, as they do not create a significant redox potential gradient between the cluster’s valence isomers. Instead, the fact that Δ*E*_VI_^(2°)^ ≃ Δ*E*_vib_^(2°)^ suggests that perturbation and desymmetrization of the cluster vibrations by those interactions may promote the trapping of energetically similar valence localized states on the potential energy surface, which, in turn, are split according to electrostatic arguments. However, the thermal motion of the cluster at ambient *T* must be assumed to completely average-out this effect.

The estimated ETse rates for ^**[2.2.2]**^ **K**_***i***_ **-[Fe**_**4**_ **S**_**4**_ **]** ^**2+/3+**^, **K**_***i***_ **-[Fe**_**4**_ **S**_**4**_ **]** ^**2+/3+**^ (where ***i*** = 2, 1) and ^**[2.2.2]**^ **K**_***j***_ **-[Fe**_**4**_ **S**_**4**_ **]** ^**2+/3+**^ **-Im** (where ***j*** = 1, 0) allow to illustrate this notion about 1° and 2° sphere effects: Though the interaction between K^+^ ions and the Fe_4_S_4_ cluster is noncovalent, the different 2° spheres of **K**_**2**_ **-[Fe**_**4**_ **S**_**4**_ **]** ^**2+**^ and **K-[Fe**_**4**_ **S**_**4**_ **]** ^**3+**^ cause a significant difference between the two redox congener’s *E*_1/2_-values, rendering Δ*G* ^0^ ≠ 0. In other words, the 2° sphere can dictate the distribution of electrons between two clusters, which are isosteric in their 1° coordination spheres, thereby inhibiting ETse. Based on this, we propose that the central influence of 2° sphere interactions is on the “extrinsic” control of an ET’s directionality, while not affecting the clusters’ (“intrinsic”) quantum mechanical properties or inner-sphere reorganization, λ^inner^. This makes the 2° sphere well-suited for the dynamic modulation of ET mechanisms, such as gated ones,^22^ via dynamic adaptation of Δ*G* ^0^. In contrast, based on the comparison of ^**[2.2.2]**^ **K**_***i***_ **-[Fe**_**4**_ **S**_**4**_ **]** ^**2+/3+**^ (where ***i*** = 2, 1) and ^**[2.2.2]**^ **K**_***j***_ **-[Fe**_**4**_ **S**_**4**_ **]** ^**2+/3+**^ **-Im** (where ***j*** = 1, 0), clusters perturbed in their 1° sphere exhibit rapid ETse. Thereby, our investigations revealed initial evidence for a potential link between the Fe_4_S_4_ complexes’ electronic structure and its “intrinsic” ET properties (λ and *H*_AB_), underscoring the notion that the key effect of the 1° sphere is to statically fine-tune the cluster for ET chemistry (Figure 17) or other types of chemical reactivity.

**Figure 17:**
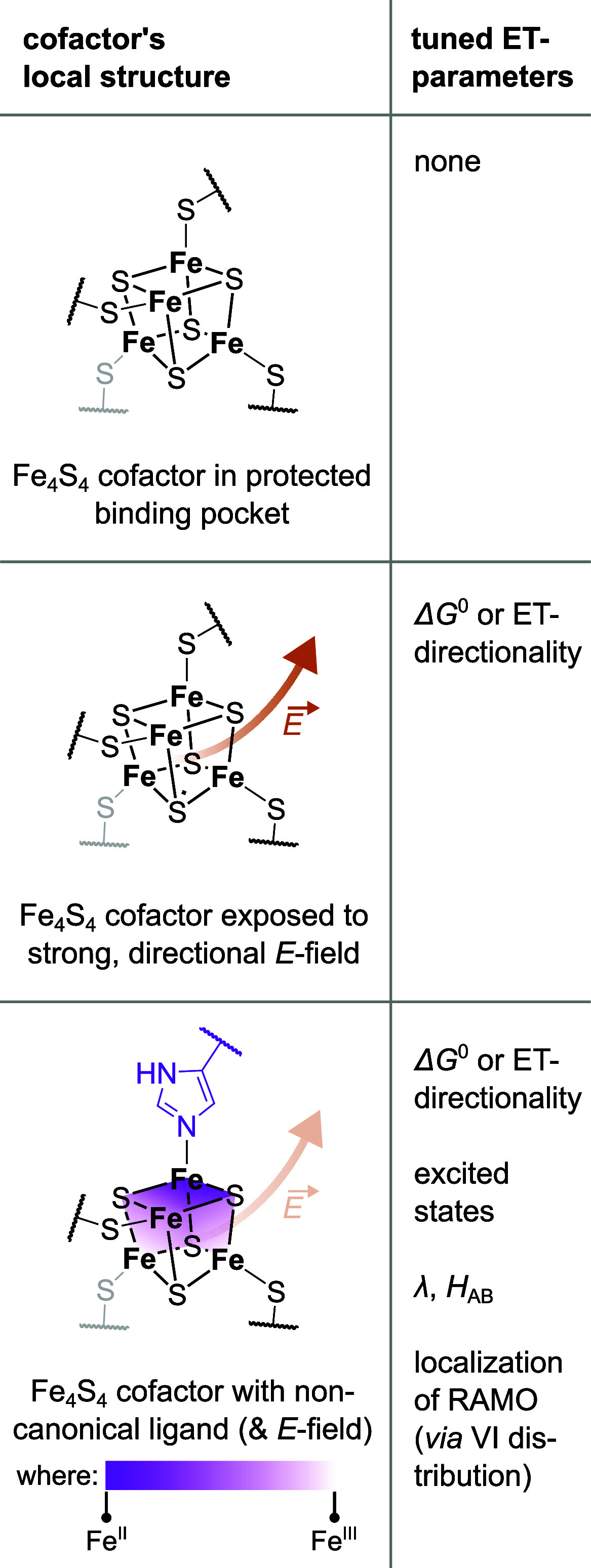
Summary of the tuned ET parameters in Fe_4_S_4_ cofactors with no interactions (*top*), 2° sphere electrostatic interactions (*middle*) and noncanonical ligation of the 1° sphere (*bottom*).

Much of this picture is also supported by our theoretical calculations at the DFT level, which were used to rationalize the effect of the 2° sphere electrostatic interactions on the cluster’s spectroscopic properties. A limitation not addressed in this work is the explicit treatment of conformers, as it has been demonstrated that structure and properties are tightly coupled for FeS cubanes,^132^ for instance ligand distortions. However, we hypothesize that the investigated DmpS^–^-supported complexes remain rather rigid, both the core Fe_4_S_4_ cubanes and the encapsulated cation(s) due to steric constraints imposed by four bulky ligands—a property that we cannot directly describe due to truncating these ligands with MeS^–^ moieties in most of our calculations. Nevertheless, and even though DFT appears to overestimate the Δ*E*_VI_^(2°)^ greatly,—this has been observed in Suess’s work on Δ*E*_VI_^(1°)^ as well^48^—the density of the experimental and computational data, and its qualitative attributes can nonetheless support a rigid assessment of the calculated parameters, and their implications.

Another limitation of the approach presented in this work is that the model systems investigated here are not suitable to probe 2° sphere interactions with partially covalent character. Particularly the interaction of Fe_4_S_4_ cofactors with H-bonding networks (“2°-H”-type interactions) are expected to fall in this category,^133^ because the proton is thought to form a partially covalent interaction with the H-bond acceptor atom.^134,135^ Nonetheless, the electrostatic dipolar contribution to 2° sphere interactions is significant and pervasive to all their kinds (Figure 1B). The present work allows suggesting that if some 2° sphere interactions possess weakly covalent character, the electronic/magnetic structure of the [Fe_4_S_4_]^2+^ oxidation state should not be strongly affected by them. In contrast, the active, [Fe_4_S_4_]^1+^ and [Fe_4_S_4_]^3+^, oxidation states may exhibit valence isomer splitting patterns in an order of magnitude that is larger than that estimated here (for “pure” electrostatics), but still significantly smaller than that associated with the 1° sphere ligation (i.e., 10 < Δ*E*_VI_^(2°‑H)^ ≪ 100 cm^–1^). Accordingly, our results provide robust “upper” and “lower” bounds for the real-world 1° and 2° sphere’s effects, which are at play in biological systems.

## 4 Conclusions

In summary, we undertook a comprehensive spectroscopic study of a systematic family of synthetic Fe_4_S_4_ clusters, encompassing canonical, Fe_4_S_4_(RS)_4_-type structures with and without 2° sphere electrostatic interactions, and 1°-sphere-perturbed, noncanonical Fe_4_S_4_(RS)_3_(Im*)-type structures. Focusing on three key attributes (Δ*E*_VI_, Δ*E*_redox_ and Δ*E*_vib_) related to ET chemistry and electronic structure, we experimentally established the energetic magnitudes of 1° vs 2° sphere perturbations for the three most biorelevant oxidation states of Fe_4_S_4_ cofactors ([Fe_4_S_4_]^1+,2+,3+^). It was found that both types of interactions affect the vibrational modes of the cubanes within 5–20 cm^–1^. However, whereas 2° sphere electrostatic interactions are very efficient at altering redox potential (or Δ*G* ^0^), equivalent Δ*G* ^0^-adjustments by 1° sphere interactions come at the cost of significant perturbations to the clusters’ electronic states (10^2^ to 10^3^ cm^–1^). These by far exceed the electronic perturbations caused by the 2° sphere (0–10 cm^–1^), whose biological relevance is either negligible or remains unproven based on the current results. 1° sphere perturbations are hereby easier recognized than the 2° sphere ones, not only due to their magnitude, but also due to their often irreversible and static nature. The much smaller 2° sphere perturbations likely proceed without making/breaking of chemical bonds, changing λ^inner^, or reshuffling electronic states, which makes them harder to detect spectroscopically, but well-suited to dynamically modulate a cluster’s Δ*G* ^0^ for ET. Together, this showcases the “intrinsic” (but static) vs “extrinsic” (and potentially dynamic) influences of the 1° vs 2° sphere interactions on the properties of these systems. More broadly, the individual Δ*E*-values reported here are reliable energetic bounds for the magnitude of such effects in real biological systems, in which those interactions are heavily conflated with one another (Figure 1B).^18,48,67,111,136^ We thus anticipate that these results could improve our ability to recognize and dissect the relevance of such interactions—particularly that of the elusive 2° sphere ones—in enzymes, while also contributing to our fundamental understanding of the mechanisms adapting Fe_4_S_4_ cofactors’ properties to their diverse functions.

## Supplementary Material







## Notes

Synthetic procedures, experimental details, and characterization data relevant to this work. This includes UV–vis electronic absorption spectra, (VT) ^1^H and ^13^C NMR spectra, cyclic voltammograms, elemental analyses, EPR data, ^57^Fe Mössbauer spectra, DFT computational details as well as further discussions on some of the spectroscopic and synthetic results (PDF)
The XYZ coordinates of DFT-optimized structures for the extended model compounds (ZIP)
The XYZ coordinates of DFT-optimized structures for the truncated model compounds (ZIP)
