# Supplementary material for: Disentangling First and Second Sphere Effects in Iron–Sulfur Cubanes
Source: JACS Au. 2025 Dec 18;6(1):209–32. doi: 10.1021/jacsau.5c01171 (PMC12848739; doi:10.1021/jacsau.5c01171)
Supplement: Supplementary file 1 [file au5c01171_si_001.pdf]

Supporting Information for

# Disentangling First and Second Sphere Effects in Iron-Sulfur Cubanes

Liam Grunwald,[§,*] Katja-Sophia Csizi,[§] Daniel Klose,[§] Vladimir Pelmenschikov,[%] Martin Clémancey,[$] Hongxin Wang,[#] Micha L. Weber,[§] Henrik Seng,[§] Yoshitaka Yoda,[¿] Daniel F. Abbott,[§] Patrick Dubourdeaux,[$] Stephen P. Cramer,[#] Markus Reiher,[§] Geneviève Blondin[$,*] and Victor Mougel[§,*]

[§] Department of Chemistry and Applied Biosciences (D-CHAB), Swiss Federal Institute of Technology Zürich (ETHZ), CH-8093 Zürich, Switzerland

[%] Institute of Chemistry, Technical University of Berlin (TUB), DE-10623, Berlin, Germany

[$] University of Grenoble Alpes, CNRS, Commissariat a l'energie atomique et aux energies alternatives (CEA), Institut de Recherche Interdisciplinaire de Grenoble (IRIG), Laboratoire de Chimie et Biologie des Metaux, Physicochimie des Metaux en Biologie (PMB), CEA Grenoble, F-38054 Grenoble Cedex, France

[#] SETI Institute, Mountain View, California 94043, United States of America

[¿] Precision Spectroscopy Division, SPring-8/JASRI, Sayo 679-5198, Japan

* Corresponding authors: Liam Grunwald, Geneviève Blondin and Victor Mougel

Email: gliam@ethz.ch, genevieve.blondin@cea.fr, mougel@inorg.chem.ethz.ch

**General Considerations** .......... 3
**Symmetry Considerations** .......... 6
**Supplementary EPR Spectra and Simulations** .......... 7
**Summary of All EPR Simulation Parameters** .......... 16
**Fitting of the HERFD-XAS Spectra** .......... 17
**Supplementary Mössbauer Spectra and Simulations** .......... 19
**$^{1}H$,$^{13}C$ NMR and VT $^{1}H$ NMR spectra** .......... 49
**Summary of the Hamiltonian Description of the Odd-electron "Pair-of-Pairs" by Suess and co-workers** .......... 67
**Modelling Valence Isomerism in the Non-Canonical $[Fe_4S_4]^{2+}$ Cubanes** .......... 69
**Further Details and Results of the VT $^{1}H$ NMR Fits** .......... 74
**Computational Details for Simulation of $^{57}Fe$ Mössbauer and EPR Parameters** .......... 75
**DFT Simulations of $^{57}Fe$ NRVS** .......... 86
**$^{57}Fe$ PVDOS Spectra of Canonical *versus* Site-Differentiated Cubanes based on NRVS** .......... 90
**UV-vis Electronic Absorption Spectra of Canonical versus Site-Differentiated Cubanes** .......... 91
**Adapted MatLab Scripts for Fitting the VT NMR Data** .......... 92
**SI References** .......... 108

## General Considerations

All canonical iron-sulfur cubane complexes studied in this work, including the $^{57}Fe$ labelled derivatives, were synthesized according to our reported procedures;[1-3] 2,6-dimesitylphenylthiol (DmpSH) and its derivatives were prepared according to our previously reported procedures as well.[1] Commercially acquired 18-crown-6 (Sigma-Aldrich) was sublimed prior to use. Toluene was purified using a Vigor® Solvent purification system and degassed by 4 freeze-pump-thaw cycles prior to use. 2-Me-THF was purchased from Sigma-Aldrich, dried over K/benzophenone, distilled, and degassed by 3 freeze-pump thaw cycles. All spectroscopic sample preparations and syntheses were carried out inside Ar-filled gloveboxes (Vigor®), where oxygen and moisture levels were maintained below 1 ppm—unless otherwise stated.

All EPR spectroscopy samples were dissolved in the appropriate solvent to yield 2 mM solutions of complex. Of these, 60 μL were transferred into a 3.0 mm quartz tube (or 4.0 mm in case of 2-Me-THF) with female NS14 adapter and capped with a well-greased male NS14 valve. The tube containing the solution was connected to a Schlenk line, frozen by immersion into liquid nitrogen, and the quartz tube was flame-sealed under dynamic vacuum.
Continuous-wave (cw) EPR spectra in X-band were recorded on an Elexsys E500 cw EPR spectrometer (Bruker Biospin, Ettlingen, Germany) equipped with a Super-High-Q resonator (Bruker Biospin) and an ESR-900 helium flow cryostat (Oxford Instruments, Oxfordshire, UK) for temperature stabilization. A gentle gas stream of dry nitrogen through the resonator was used to avoid condensation. Q-band cw EPR spectra were recorded on an Elexsys E580 EPR spectrometer (Bruker Biospin) equipped with a cryogen-free variable temperature EPR cryostat (Cryogenic Ltd., London, UK) and a home-built cw EPR resonator with 3 mm sample access.[4]
In both frequency bands, a magnetic field modulation with 100 kHz and amplitudes of 0.1 to 0.3 mT were applied for lock-in detection with a conversion time and time constant of 163.84 ms and 40.96 ms, respectively. Spectra were acquired with steps of <0.1 mT and at a microwave frequency of either ca. 9.4 GHz or 33.8 GHz at non-saturating microwave power levels. For temperature-dependent experiments, a non-saturating microwave power was determined at the lowest temperature and maintained throughout the series. In post-processing using MATLAB (The Mathworks, Massachusetts, USA), spectra were baseline-corrected using a first-order polynomial function, the magnetic field offset was corrected using a reference measurement of the radical DPPH (Sigma Aldrich, Buchs, Switzerland), and $S$=1/2 spectra were scaled to 9.5 GHz for unified presentation. Spectral simulations were carried out with the MATLAB package EasySpin.[5]
To record spectra of the mixtures with 18-crown-6 or K[BArF$_{24}$], the cluster **$K_3$-[$Fe_4S_4$]$^{1+}$** was stoichiometrically mixed with the reagent on a larger scale (10 mg of cluster minimum), and a 60 μL aliquot was taken for EPR measurement from the stirred reaction mixture after 1 minute.

$^{57}Fe$ Mössbauer spectra were recorded on powder and solution samples contained in Delrin cups at 5-6 K and 80 K on either a low-field Mössbauer spectrometer equipped with a Janis SVT-400 cryostat, a low-field Mössbauer spectrometer equipped with a closed-cycle SHI-850-5 cryostat from 57 Janis and SHI, or on a strong-field Mössbauer spectrometer equipped with an Oxford Instruments Spectromag 4000 cryostat containing an 8 T split-pair superconducting magnet. The three spectrometers were operated in a constant acceleration mode in transmission geometry. The isomer shifts were referenced against that of a room-temperature metallic iron foil. Analysis of the data was performed with a home-made program (SimuMoss software. C. Charavay, S. Segard, F. Edon, M. Clémancey, G. Blondin. CEA/iRTSV, CNRS, Univ. Grenoble Alpes).[6]

Powder spectra were recorded on complexes with natural iron isotope distribution. Solution spectra were recorded on complexes synthetized with a 95%-enriched $^{57}Fe$ source.
Spectra recorded at 5-6 K on $K_2[^{57}Fe_4S_4(DmpS)_4]\cdot2(HMDSO)$ dissolved in toluene were corrected by a parabola evaluated on the data points with velocities below –1.5 mm $s^{-1}$ and above +2.4 mm $s^{-1}$. The crude data with those after correction are given in Figure S18.

X-ray absorption spectroscopy (XAS) measurements were performed at the S K-edge (2.472 keV) at the ID26 beamline of the European Synchrotron Radiation Facility (ESRF) (Grenoble, France).[7] The first harmonic of the undulator source was used, and higher harmonics were rejected using Si-coated mirrors. The incident beam was monochromatized using a cryogenically cooled Si(111) double-crystal monochromator and Si-coated focusing mirrors were used to achieve a spot size of 200 x 100 $\mu m^2$ at the sample position. The dispersive optics of the spectrometer and the sample analysis chamber are always maintained under vacuum.[8] High energy resolution fluorescence detected (HERFD) XAS were measured at the maximum of the $K_\alpha$ emission mainline. The measurements utilized a crystal array spectrometer with six cylindrically bent $LiNbO_3$ (10-4) crystals with a 1 m radius of curvature. For the XAS measurements, the incident energy wasn't specifically calibrated since there was no suitable reference foil at low energies. The calibration was instead based on the previous calibration done at Mn K-edge (6.539 keV). The measurements were performed using a multi-wire gas detector.[8]
All samples were finely ground into powders using a mortar and pestle and then loaded into a custom sample plate, where the sample powders were protected from the atmosphere by a thin, sulfur-free Kapton foil—both of these were generously provided by the beamline staff. Sample loading was performed inside an Ar-filled glovebox and the sample holders were individually sealed airtight in multiple layers, allowing inert transport to the beamline. The sample holders were transferred into the vacuum loading chamber of the spectrometer quickly. To assess beam-damage effects, quick near-edge XAS spectra were consecutively recorded on the same position in the sample until differences in the consecutive spectra were noticed. During a 10 s exposure of the sample, we were unable to detect beam-damage induced spectral distortions and, therefore, 10 s scans were performed in 150 individual positions throughout the sample, respectively, and their average was used to construct the final spectrum. For some samples, a small number of individual spectra were manually excluded as outliers, because of the low signal-to-noise ratio; This was likely due to minor sample inhomogeneities (*i.e.* crystallite/particle sizes). Furthermore, because self-absorption is a known problem for S K-edge XAS, we tested the dilution of our samples with boron-nitride, as it is commonly done. However, we were unable to observe significant qualitative differences in the spectral attributes between the diluted and undiluted samples, besides an improved signal-to-noise ratio of the undiluted powders. Therefore, in this work, we will present the data recorded on the undiluted samples. HERFD-XAS data were initially analyzed using the PyMCA software package.[9] This included background subtraction, normalization, and energy-calibration *vs.* the literature-known compounds, $(Et_4N)_2[Fe_4S_4(PhS)_4]$, $(Et_4N)_2[Fe_4S_4(PhSe)_4]$ and $(Et_4N)_2[Fe_4Se_4(PhS)_4]$.
Fitting of the pre-edge region was carried out according to the procedure described by us previously.[10]

$^{57}Fe$ nuclear resonance spectroscopy was measured with a published method[11-14] at SPring-8 BL19LXU. A beamtime with an operational C mode is needed which has a 145.5 ns interval between X-ray pulses to match the 1/e nuclear decay period (143 ns) for $^{57}Fe$. The 100 eV in bandwidth X-ray beam from the planar undulator was first monochromatized to ~1 eV bandwidth by a high heat load monochromator, and then further monochromatized to 0.8 meV in linewidth *via* a high-resolution monochromator (HRM). The energy exited from HRM is centered at the $^{57}Fe$ nuclear resonant energy of 14.4 keV, and scans across the vibrational energy region (*e.g.* 80 meV). Following the nuclear resonance and vibrational excitation at energies around 14.4 keV, at the perpendicular position to the

incident beam, a 2×2 avalanche photodiodes array (APDs) was used to detect the $^{57}Fe$ nuclear fluorescence ($h\nu_1$=14.4 keV) and the converted Fe $K_\alpha$ fluorescence ($h\nu_2$=6.4 keV). Since APDs have fast response and high time resolution, the weak nuclear scattering signal ($\tau$=143 ns for $^{57}Fe$) can be well separated from the huge electronic scattering background (often in fs) in the time domain, avoiding a low throughput diffraction spectrometer.[11-14] Then the pure nuclear scattering signal *vs*. the vibrational energies forms raw NRVS spectra. For most of the iron-sulfur complexes, an even time of 3 seconds per point (3 s/p) was used to scan the whole spectrum from –240 to 540 $cm^{-1}$. For the complexes with possible high energy features, the scan range was extended to 650 $cm^{-1}$ and sectional scans were used to emphasize the possible high energy region. For example, 6 s/p was used for the 400-650 $cm^{-1}$ region while 2 s/p was used for the region from –240 $cm^{-1}$ to 400 $cm^{-1}$. Each final spectrum in this publication is the average of 6-10 such scans.
The collected raw NRVS scans were processed *via* spectra.tools[14, 15] which combines the data format transformation with PHOENIX software package[14, 16] to yield $^{57}Fe$ partial vibrational density of states (PVDOS). In principle, the calculation of element specific PVDOS involves sum of the raw NRVS, Fourier-log calculation and spectral normalization through Lipkin's sum rule.[15, 17]
To maintain samples' integrity during the measurement, to increase the scattering intensity in the Stoke branch and to increase samples' Lambda-Mössbauer factor, the NRVS samples were cooled with a helium flow cold finger cryostat or an enclosed helium circulator maintained at 10K. Nevertheless, the real sample temperatures determined from NRVS analysis *via* spectra.tools were found from 40 to 65 K due to the arrangement that NRVS has an extremely close sample-detector distance to increase signal.[14, 18]

$^{1}H$ and $^{13}C$ NMR spectra were recorded on a 300 MHz Bruker AVII spectrometer at room temperature. $^{1}H$ and $^{13}C$ spectra are reported in parts per million (ppm) and are calibrated with respect to the corresponding solvent residual peak. $^{13}C$ NMR spectra were recorded with complete proton decoupling and the spectra are likewise calibrated with respect to the corresponding solvent residual peak. Multiplet signals are reported as follows: s=singlet, d=doublet, t=triplet, q=quartet, quint=quintet, sept=septet, m=multiplet, b=broad, or combinations thereof.

VT $^{1}H$ NMR spectra were recorded on a 500 MHz Bruker AVANCE DRX spectrometer equipped with a cryostat. The sample temperature was allowed to equilibrate for 5-10 minutes before each VT spectrum was acquired. The temperature evolution of the shifts were simulated using the adaptations of the MatLab code provided by Suess and co-workers in their 2023 publication.[19] For the diamagnetic contribution to the chemical shift, the values measured on DmpSH, $(DmpS)_2$ DmpSK or $DmpS^{(18\text{-}C\text{-}6)}K$ (*vide infra*) were all considered, yielding negligible differences to the fitted values in the spectra of $[Fe_4S_4(DmpS)_3(Im^*)]$. For Im*, we considered only the values of the neutral ligand molecule.

## Symmetry Considerations

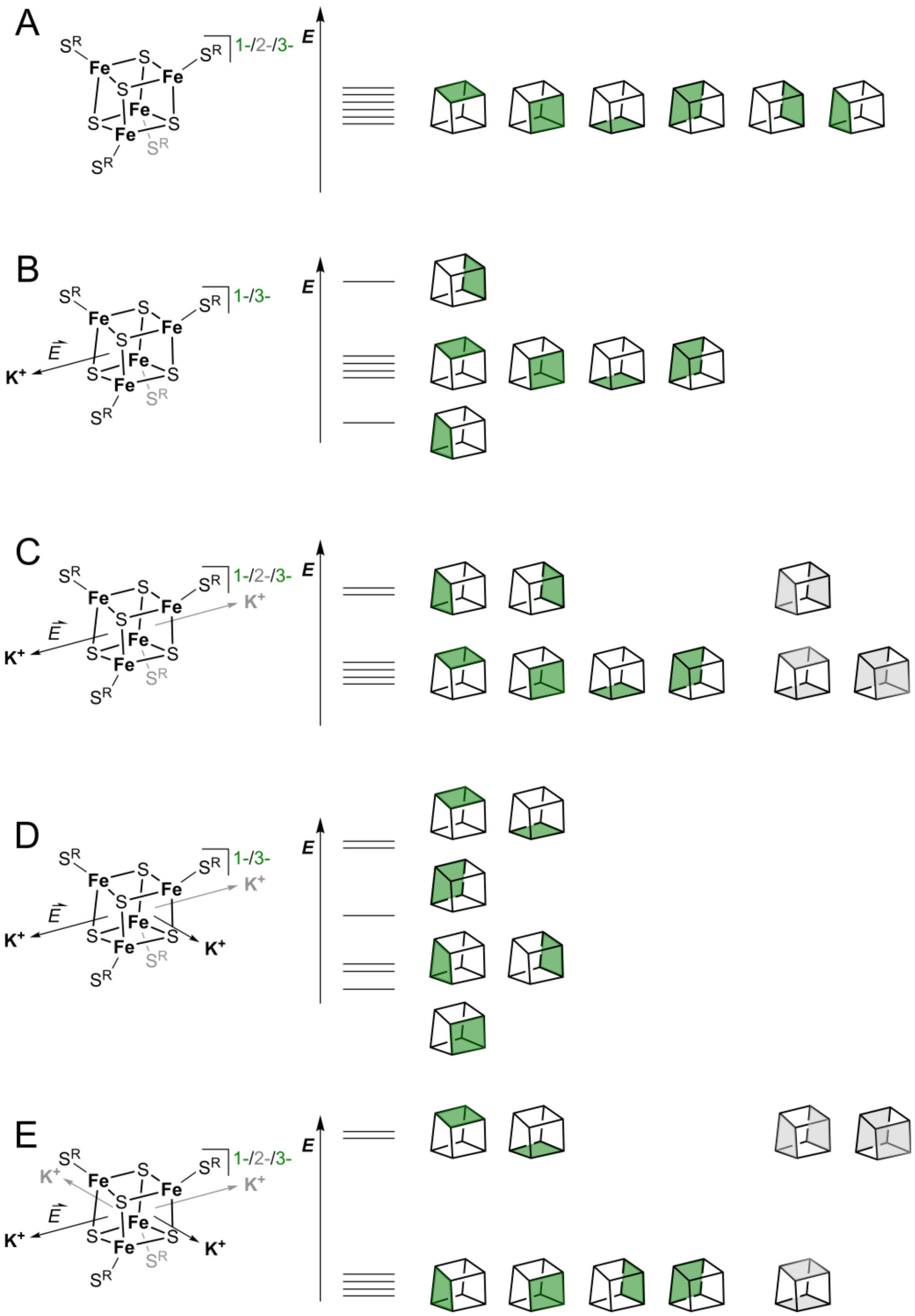


**Figure S1.** Symmetry considerations for the energy splitting schemes of valence isomers/topological coupling isomers of $[Fe_4S_4]^{3+/2+/1+}$ complexes with varying number of $K^+$ cations. The direction of the electric field gradient, $\vec{E}$, resulting from the cation/anion pair is indicated as an arrow. In all cases of electron-/hole- bearing oxidation states ($[Fe_4S_4]^{1+}$/$[Fe_4S_4]^{3+}$; *green*), we suggest that the energetically lowest lying state is oriented such that the lower-valent $Fe_2S_2$ unit (*green*) is in an electrostatically favorable position, close to the cation. For $[Fe_4S_4]^{2+}$ (*gray*), we suggest that the orientation of the antiferromagnetic coupling between the $Fe_2S_2$ subunits (*gray*) perpendicular to the electric field gradients is favored over a parallel alignment.

## Supplementary EPR Spectra and Simulations

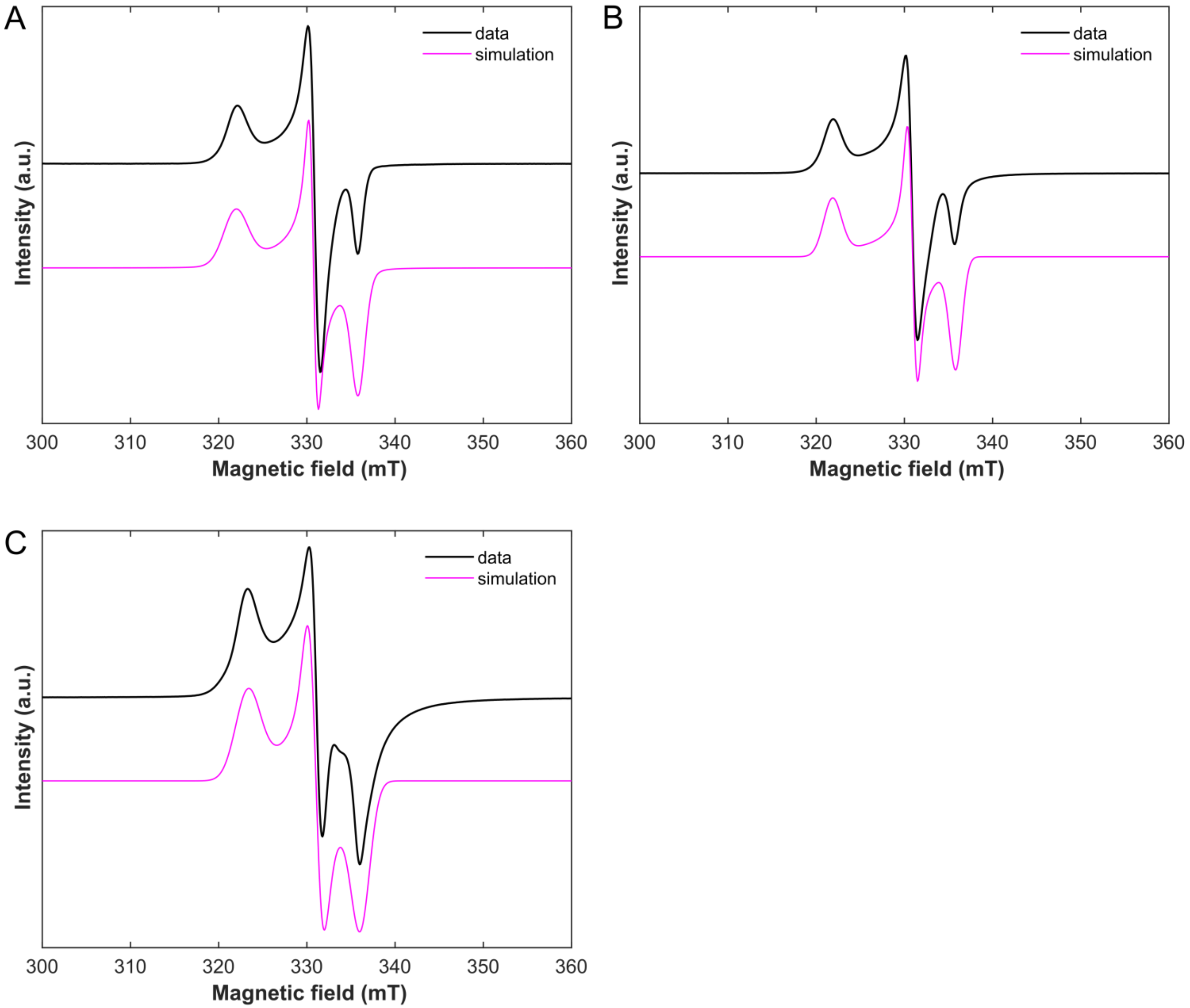


**Figure S2.** X-band perpendicular mode EPR spectra of: (A) **K-[$Fe_4S_4$]$^{3+}$** recorded at 10 K in frozen 2-Me-THF solution. Data are represented as a *bold black line* and a simulation as a *magenta line*. The simulation parameters are: *S*=1/2, *g*=(2.109, 2.052, 2.021) with *g*-strains of (0.020, 0.007, 0.010); (B) **$^{[2.2.2]}$K-[$Fe_4S_4$]$^{3+}$** recorded at 10 K in frozen 2-Me-THF solution. Data are represented as a *bold black line* and a simulation as a *magenta line*. The simulation parameters are: *g*=(2.109, 2.051, 2.021) with *g*-strains of (0.016, 0.006, 0.010); and (C) **$^{[2.2.2]}$K-[$Fe_4S_4$]$^{3+}$** recorded at 10 K in frozen toluene solution. Data are represented as a *bold black line* and a simulation as a *magenta line*. The simulation parameters are: *g*=(2.100, 2.051, 2.019) with *g*-strains of (0.022, 0.011, 0.015).

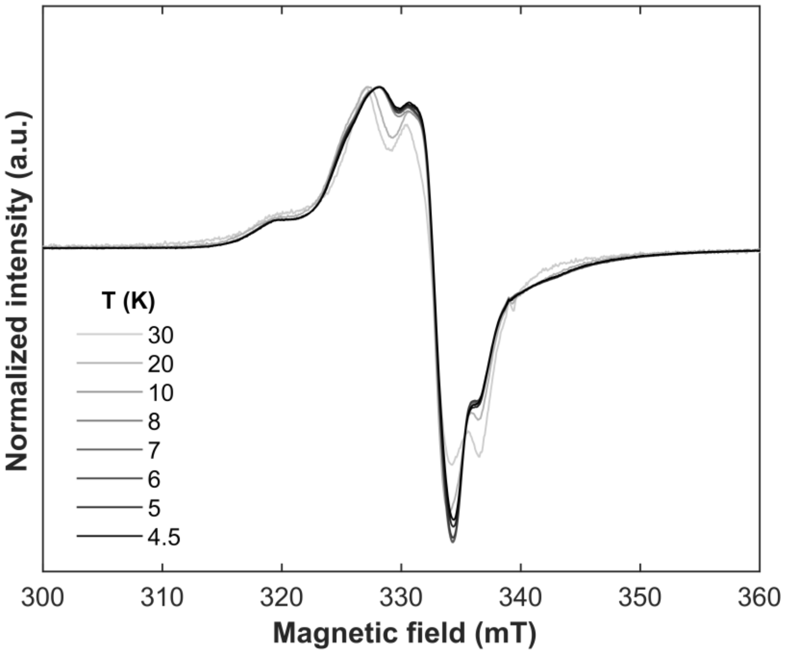


**Figure S3.** Normalized temperature-dependent X-band perpendicular mode EPR spectra of **K-[$Fe_4S_4$]$^{3+}$** recorded at varying temperatures between 4.5 and 30 K in frozen toluene solution.

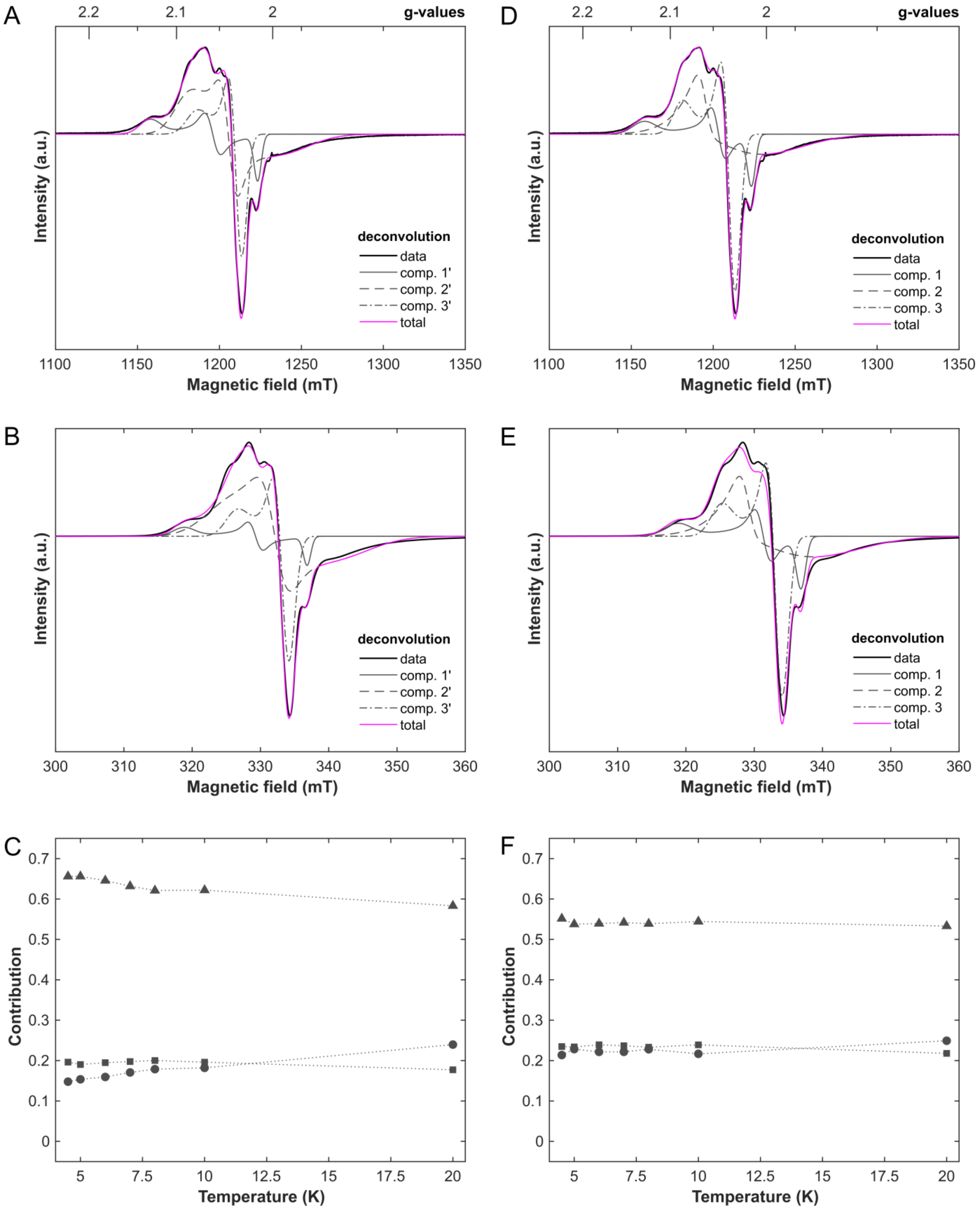


**Figure S4.** Continued on the following page.

**Figure S4.** (A,D) Q-band perpendicular mode EPR spectrum of **K-[$Fe_4S_4$]$^{3+}$** recorded at 10 K in frozen toluene solution and simulations presenting two different arrangements of the lines: (A) All components *S*=1/2; component 1' (*solid grey line*) *g*=(2.130, 2.062, 2.015) with *g*-strains of (0.027, 0.015, 0.008) and a weight of 21%; component 2' (*dashed grey line*) *g*=(2.086, 2.043, 1.984) with *g*-strains of (0.037, 0.015, 0.064) and a weight of 62%; component 3' (*dotted-dashed grey line*) *g*=(2.080, 2.036, 2.033) with *g*-strains of (0.025, 0.014, 0.014) and a weight of 17%. (D) All components *S*=1/2; component 1 (*solid grey line*) *g*=(2.130, 2.049, 2.015) with *g*-strains of (0.030, 0.014, 0.009) and a weight of 21%; component 2 (*dashed grey line*) *g*=(2.063, 2.040, 1.993) with *g*-strains of (0.012, 0.095, 0.099) and a weight of 56%; component 3 (*dotted-dashed grey line*) *g*=(2.088, 2.035, 2.036) with *g*-strains of (0.022, 0.015, 0.015) and a weight of 23%. (B,E) X-band perpendicular mode EPR spectrum of **K-[$Fe_4S_4$]$^{3+}$** recorded at 10 K in frozen toluene solution and corresponding simulations presenting two different arrangements of the lines: (B) All components *S*=1/2; component 1 (*solid grey line*) *g*=(2.130, 2.062, 2.015) with *g*-strains of (0.023, 0.013, 0.007) and a weight of 11%; component 2 (*dashed grey line*) *g*=(2.086, 2.043, 1.984) with *g*-strains of (0.057, 0.021, 0.070) and a weight of 72%; component 3 (*dotted-dashed grey line*) *g*=(2.080, 2.036, 2.033) with *g*-strains of (0.025, 0.014, 0.015) and a weight of 17%. (E) All components *S*=1/2; component 1 (*solid grey line*) *g*=(2.130, 2.049, 2.015) with *g*-strains of (0.030, 0.014, 0.009) and a weight of 21%; component 2 (*dashed grey line*) *g*=(2.063, 2.040, 1.993) with *g*-strains of (0.012, 0.095, 0.099) and a weight of 56%; component 3 (*dotted-dashed grey line*) *g*=(2.088, 2.035, 2.036) with *g*-strains of (0.023, 0.015, 0.015) and a weight of 23%. (C,F) Temperature-evolution of the weights of the three fitting components for the two arrangements of lines based on the simulations in Figure S4A,B,D,E and data in Figure S3. For all spectra, data are represented as a *bold black line* and a deconvoluted simulation as a *magenta line*.

**Note S1.** Due to the good fit of the lineshape and intensity of the arrangement of lines as presented in Figure S4 panels D and E, and in particular due to the superior capability of this arrangement to reproduce the lineshape at varying *T* (if *g*-strains are optimized), we believe that the corresponding simulation parameters likely describe the physical reality better than the assignment shown in panels A and B.
Furthermore, using the above-described simulation parameters, we simulated the spectra at W-band (94.2 GHz), in order to probe whether it is feasible to obtain higher-resolution data at said field strength. However, we did not predict a significant improvement over the X- and Q-band spectra, which is why we chose not to pursue such high-field EPR experiments.

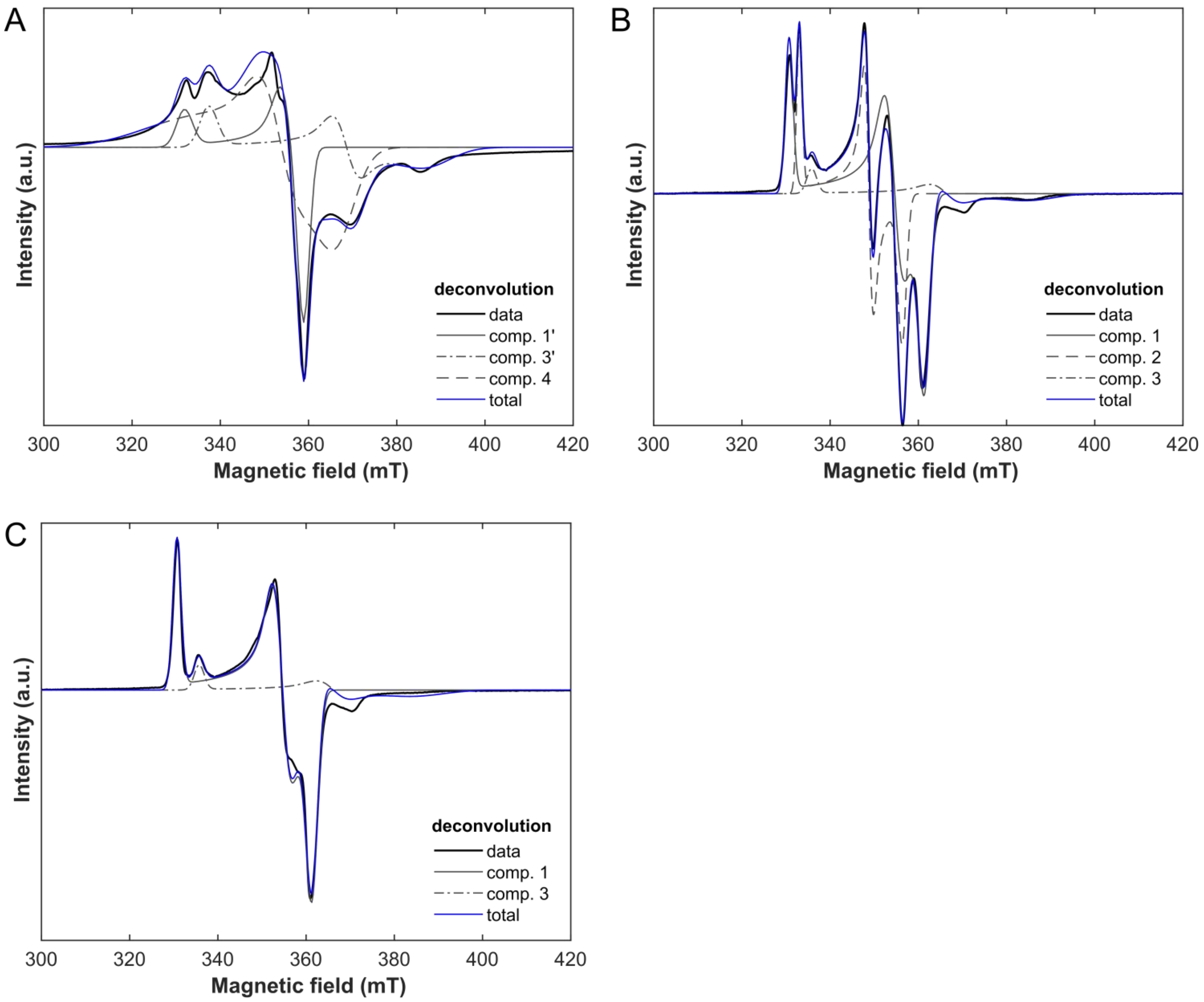


**Figure S5.** X-band perpendicular mode EPR spectra and simulations of **$K_3$-[$Fe_4S_4$]$^{1+}$** recorded at 10 K in frozen 2-Me-THF solution in presence of (A) 2 equiv. of K[BArF$_{24}$], (B) 2 equiv. 18-crown-6 and (C) 4 equiv. 18-crown-6. Data are represented as a *bold black line* and simulation as a *blue line*, deconvoluted into spectral components shown as *gray lines*. Simulation parameters are: (A) All components *S*=1/2; component 1' (*solid grey line*) *g*=(2.046, 1.908, 1.892) with *g*-strains of (0.025, 0.024, 0.015) and a weight of 14%; component 3' (*dashed dotted grey line*) *g*=(2.011, 1.840, 1.753) with *g*-strains of (0.027, 0.029, 0.060) and a weight of 27%; component 4 (*dashed grey line*) *g*=(2.054, 1.926, 1.856) with *g*-strains of (0.167, 0.048, 0.046) and a weight of 59%. (B) All components *S*=1/2; component 1 (*solid grey line*) *g*=(2.052, 1.915, 1.879) with *g*-strains of (0.013, 0.023, 0.016) and a weight of 55%; component 2 (*dashed grey line*) *g*=(2.038, 1.946, 1.905) with *g*-strains of (0.009, 0.011 0.012) and a weight of 28%; component 3 (*dashed-dotted grey line*) *g*=(2.020, 1.851, 1.760) with *g*-strains of (0.015, 0.034, 0.061) and a weight of 17%. (C) All components *S*=1/2; component 1 (*solid grey line*) *g*=(2.052, 1.915, 1.879) with *g*-strains of (0.013, 0.022, 0.016) and a weight of 77%; component 3 (*dashed-dotted grey line*) *g*=(2.020, 1.851, 1.760) with *g*-strains of (0.015, 0.035, 0.070) and a weight of 23%.

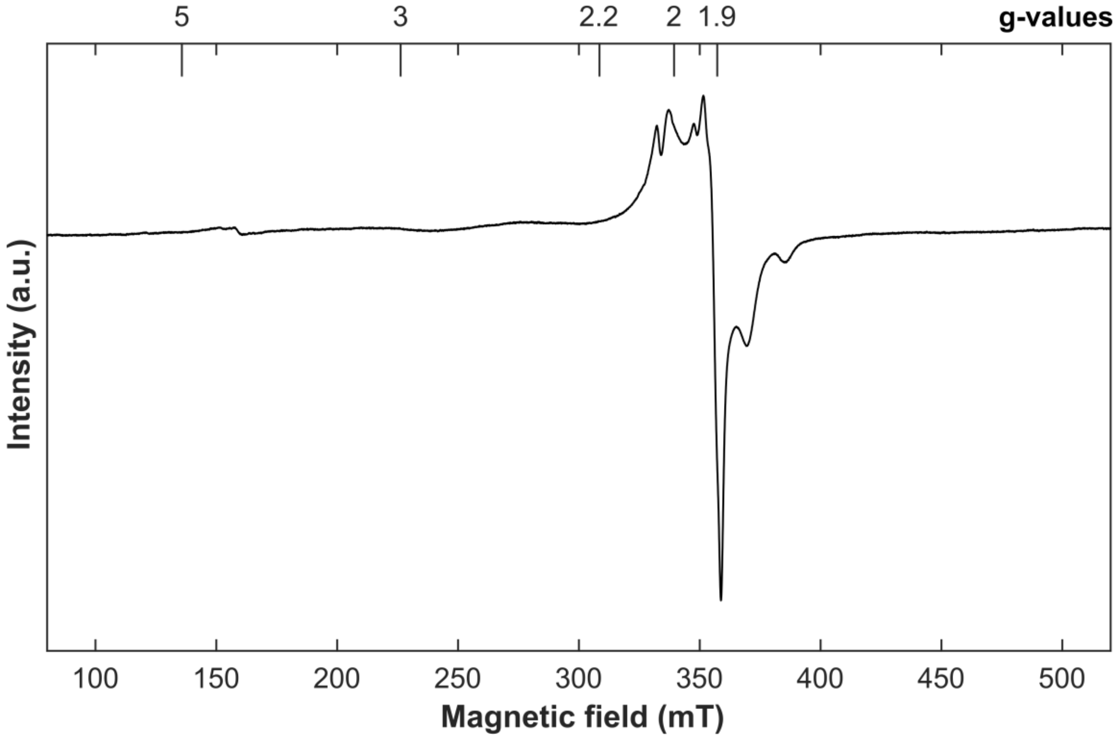


**Figure S6.** X-band perpendicular mode EPR spectrum of a frozen 2-Me-THF solution of **$K_3$-[$Fe_4S_4$]$^{1+}$** measured at 10 K shown over a wider field range.

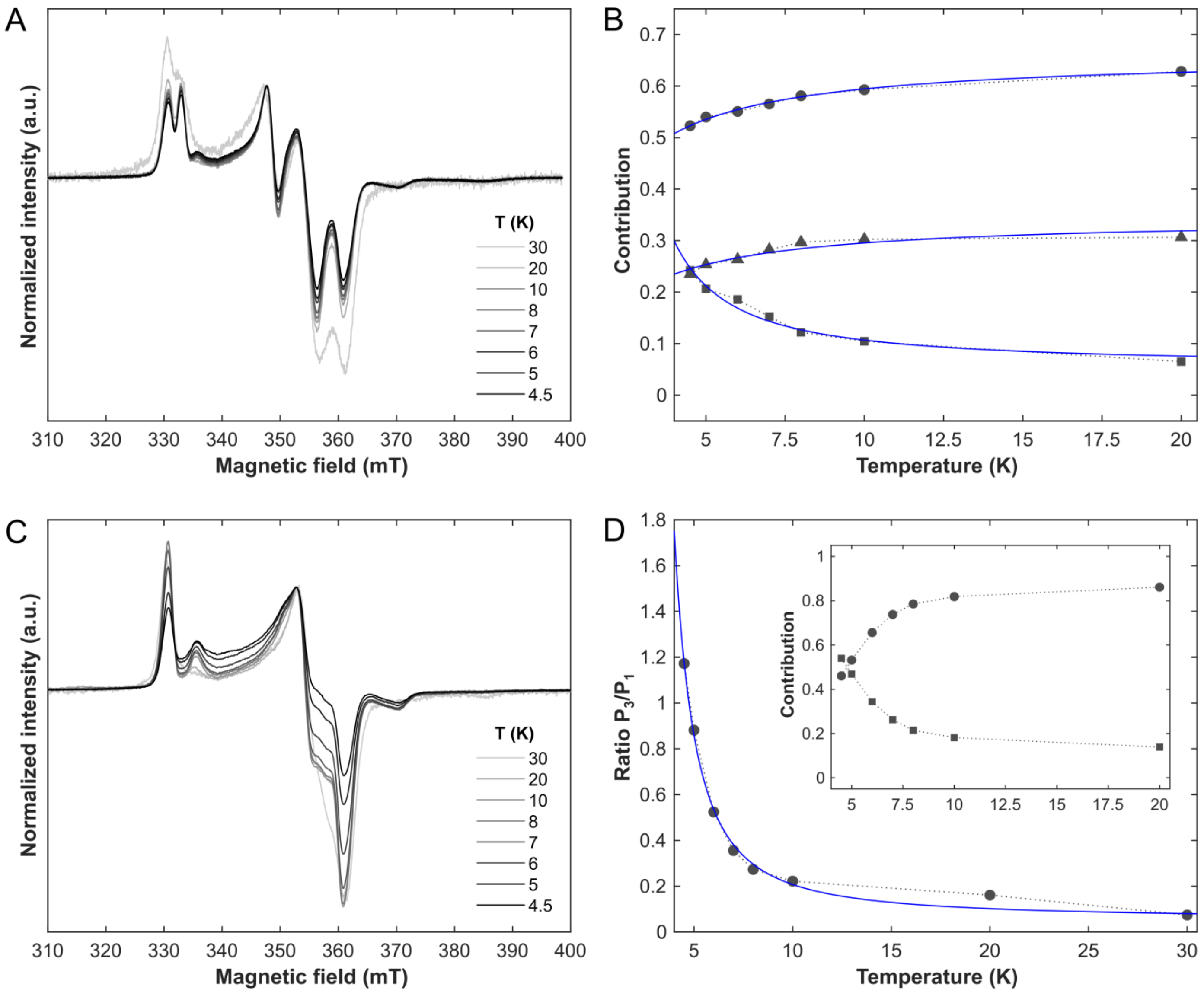


**Figure S7.** (A) Normalized temperature-dependent X-band perpendicular mode EPR spectra of a frozen 2-Me-THF solution of $\mathbf{K_3\text{-}[Fe_4S_4]^{1+}}$ in presence of 2 equiv. 18-crown-6 at varying temperatures between 4.5 and 30 K. (B) Temperature evolution of the weights of the three individual components (1: *dots*; 2: *triangles*; 3: *squares*) according to the fitting model presented in Figure S5. *Blue lines* represent fitting of the data according to Boltzmann-functions of the type: $P_i = (1/Q)exp\,(-E_i/(k_BT))$, where $P_i$ denotes the relative population, $E_i$ is the state's energy, $k_B$ is the Boltzmann constant, $T$ is temperature and $Q$ is the effective normalization denominator.[19] Based on this, the relative energy differences between the three components are approximated as $\Delta E_{12}$=0.5±0.7$k_B$, $\Delta E_{13}$=7.9±1.5$k_B$ and $\Delta E_{23}$=8.4±2.0$k_B$. Due to the different linewidths in the spectrum recorded at 30 K, this spectrum was omitted from the Boltzmann-type analysis. (C) Normalized temperature-dependent X-band perpendicular mode EPR spectra of a frozen 2-Me-THF solution of $\mathbf{K_3\text{-}[Fe_4S_4]^{1+}}$ in presence of 4 equiv. 18-crown-6 at varying temperatures between 4.5 and 30 K. (D) Temperature evolution of the population ratio between component 3 and component 1, $P_3/P_1$, based on the fitting model as in B. Data are shown as *dots* and a *blue line* is a fit of the data according to a Boltzmann function of the type: $(P_3/P_1) = C\,exp\,(\Delta E_{13}/(k_BT))$. Here, the factor $C$ accounts for possible differences in signal intensity.[19] *Inset*: Temperature evolution of the individual component populations (1: *dots*; 3: *squares*) for comparison with the data shown in panel (B). The relative energy difference is approximated as $\Delta E_{13}$=14.2±1.7$k_B$.

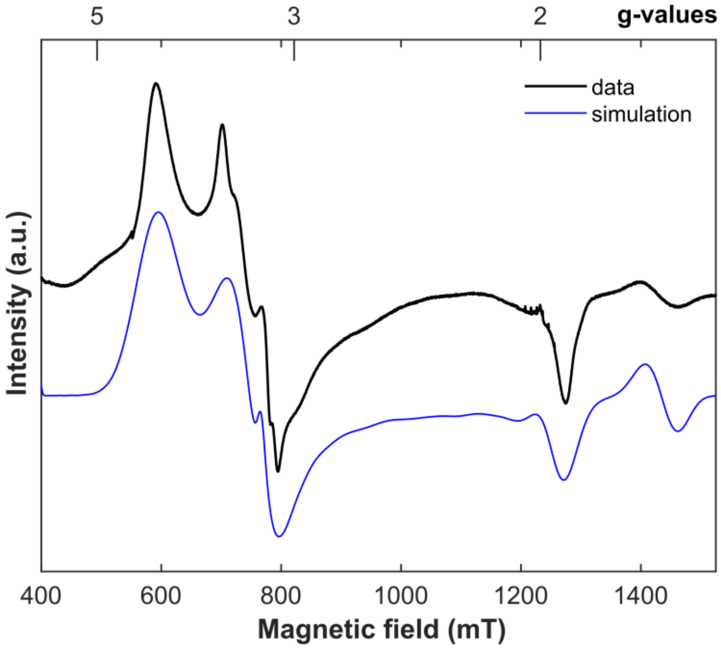


**Figure S8.** Q-band perpendicular mode EPR spectrum of powdered **$K_3$-[$Fe_4S_4$]$^{1+}$** immobilized in eicosane, recorded at 10 K. Data is presented as a *bold black line* and a simulation as *blue line*. The Simulated parameters are: *S*=3/2; *g*=(1.907, 1.964, 2.082), *D*=–1.30 cm$^{-1}$, *E*/*D*=0.07 and *g*-strains of (0.170, 0.259, 0.034). The zero-field splitting and rhombicity were used later-on as fixed values to simulate the data shown in Figure S9.

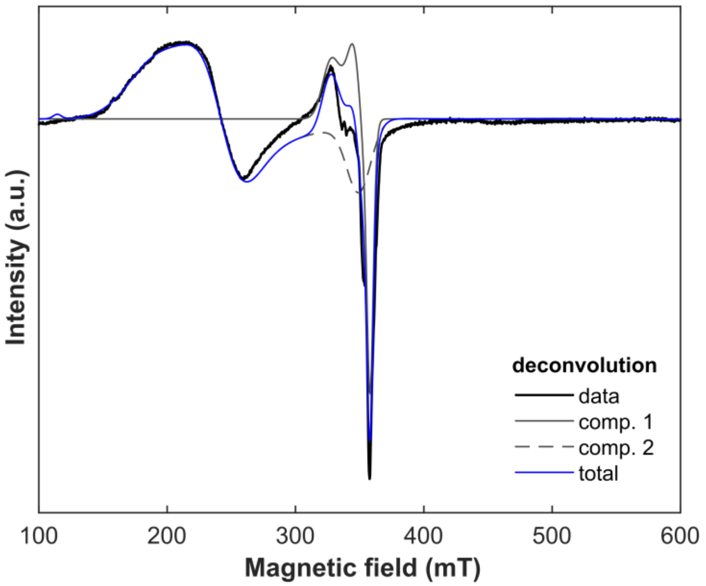


**Figure S9.** Fit of the X-band perpendicular mode EPR spectrum of **$K_3$-[$Fe_4S_4$]$^{1+}$** recorded at 10 K in frozen toluene solution. Data is presented as a *bold black line* and a deconvoluted simulation as *blue line*. The zero-field splitting parameters, *D* and *E*/*D* for the *S*=3/2 contribution were adapted from the fit of the Q-band spectrum of the powdered sample, shown above. The simulated parameters are: component 1 (*solid grey line*) *S*=1/2, *g*=(1.899, 1.941, 2.078), *g*-strains of (0.027, 0.066, 0.097) and a weight of 5%; component 2 (*dashed grey line*) *S*=3/2, *g*=(1.569, 1.636, 1.972), *D*=–1.30 cm$^{-1}$, *E*/*D*=0.07, *g*-strains of (0.234, 0.458, 0.120) and a weight of 95%.

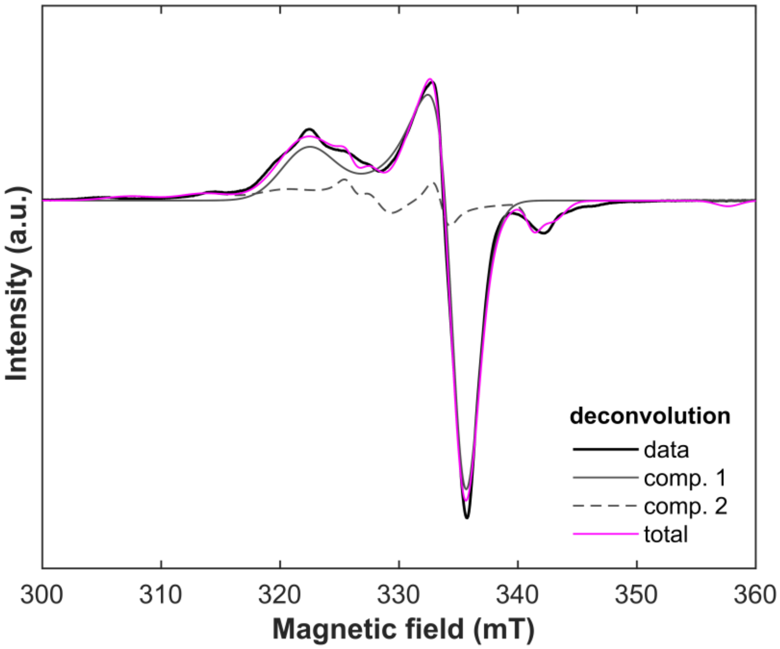


**Figure S10.** X-band perpendicular mode EPR spectrum of [$Fe_4S_4(DmpS)_3(Im^*)$] recorded at 10 K in frozen toluene solution. Data is presented as a *bold black line* and a deconvoluted simulation as *magenta line*. The simulated parameters are: component 1 (*solid grey line*) *S*=1/2, *g*=(2.107, 2.029, 2.026), *g*-strains of (0.034, 0.028, 0.014) and a weight of 89%; component 2 (*dashed grey line*) *S*=3/2, *g*=(2.164, 1.980, 2.034), *D*=0.004 $cm^{-1}$, *E*/*D*=0.94, *g*-strains of (0.033, 0.014, 0.008) and a weight of 11%.

## Summary of All EPR Simulation Parameters

**Table S1.** Summary of all simulation parameters of the EPR spectra presented in this work.

| sample | solvent | comp. | *S* | ($g_x$, $g_y$, $g_z$) | $g_{av}$ | *g*-strains | *D* ($cm^{-1}$) | *E/D* | weight |
|---|---|---|---|---|---|---|---|---|---|
| **K-[$Fe_4S_4$]$^{3+}$** | 2-Me-THF | 1 | 1/2 | (2.109, 2.052, 2.021) | **2.061** | (0.020, 0.007, 0.010) | - | - | 100% |
| **K-[$Fe_4S_4$]$^{3+}$** | toluene | 1 | 1/2 | (2.130, 2.049, 2.015) | **2.065** | (0.030, 0.014, 0.009) | - | - | 21% |
| | | 2 | 1/2 | (2.063, 2.040, 1.993) | **2.032** | (0.012, 0.095, 0.099) | - | - | 56% |
| | | 3 | 1/2 | (2.088, 2.035, 2.036) | **2.053** | (0.022, 0.015, 0.015) | - | - | 23% |
| | | 1' | 1/2 | (2.130, 2.062, 2.015) | **2.069** | (0.027, 0.015, 0.008) | - | - | 21% |
| | | 2' | 1/2 | (2.086, 2.043, 1.984) | **2.038** | (0.037, 0.015, 0.064) | - | - | 62% |
| | | 3' | 1/2 | (2.080, 2.036, 2.033) | **2.050** | (0.025, 0.014, 0.014) | - | - | 17% |
| **[2.2.2]K-[$Fe_4S_4$]$^{3+}$** | 2-Me-THF | 1 | 1/2 | (2.109, 2.051, 2.021) | **2.060** | (0.016, 0.006, 0.010) | - | - | 100% |
| **[2.2.2]K-[$Fe_4S_4$]$^{3+}$** | toluene | 1 | 1/2 | (2.100, 2.051, 2.019) | **2.057** | (0.022, 0.011, 0.015) | - | - | 100% |
| **$K_3$-[$Fe_4S_4$]$^{1+}$** | toluene | 1 | 1/2 | (1.899, 1.941, 2.078) | **1.973** | (0.027, 0.066, 0.097) | - | - | 5% |
| | | 2 | 3/2 | (1.569, 1.636, 1.972) | **1.726** | (0.234, 0.458, 0.120) | -1.30 | 0.07 | 95% |
| **$K_3$-[$Fe_4S_4$]$^{1+}$** | 2-Me-THF | 1' | 1/2 | (2.046, 1.908, 1.892) | **1.948** | (0.026, 0.024, 0.015) | - | - | 14% |
| | | 3' | 1/2 | (2.011, 1.840, 1.753) | **1.868** | (0.027, 0.029, 0.060) | - | - | 27% |
| | | 4 | 1/2 | (2.054, 1.926, 1.856) | **1.945** | (0.167, 0.048, 0.046) | - | - | 59% |
| **$K_3$-[$Fe_4S_4$]$^{1+}$** | none | 1 | 3/2 | (1.907, 1.964, 2.082) | **1.984** | (0.170,0.259, 0.034) | -1.30 | 0.07 | 100% |
| **$K_3$-[$Fe_4S_4$]$^{1+}$ +2 eq. K[$BArF_{24}$]** | 2-Me-THF | 1' | 1/2 | (2.045, 1.941, 1.893) | **1.960** | (0.030, 0.048, 0.012) | - | - | 17% |
| | | 2' | 1/2 | (2.052, 1.910, 1.855) | **1.939** | (0.136, 0.028, 0.060) | - | - | 59% |
| | | 3 | 1/2 | (2.011, 1.845, 1.753) | **1.870** | (0.024, 0.025, 0.053) | - | - | 24% |
| **$K_3$-[$Fe_4S_4$]$^{1+}$ +40 eq. K[$BArF_{24}$]** | 2-Me-THF | 1' | 1/2 | (2.045, 1.941, 1.893) | **1.960** | (0.030, 0.048, 0.012) | - | - | 17% |
| | | 2' | 1/2 | (2.052, 1.910, 1.855) | **1.939** | (0.136, 0.028, 0.060) | - | - | 59% |
| | | 3 | 1/2 | (2.011, 1.845, 1.753) | **1.870** | (0.024, 0.025, 0.053) | - | - | 24% |
| **$K_3$-[$Fe_4S_4$]$^{1+}$ +2 eq. 18-crown-6** | 2-Me-THF | 1 | 1/2 | (2.052, 1.915, 1.879) | **1.949** | (0.013, 0.023, 0.016) | - | - | 55% |
| | | 2 | 1/2 | (2.038, 1.946, 1.905) | **1.936** | (0.009, 0.011, 0.012) | - | - | 28% |
| | | 3 | 1/2 | (2.020, 1.851, 1.760) | **1.877** | (0.015, 0.034, 0.061) | - | - | 17% |
| **$K_3$-[$Fe_4S_4$]$^{1+}$ +4 eq. 18-crown-6** | 2-Me-THF | 1 | 1/2 | (2.052, 1.915, 1.879) | **1.949** | (0.013, 0.022, 0.016) | - | - | 77% |
| | | 3 | 1/2 | (2.020, 1.851, 1.760) | **1.877** | (0.015, 0.035, 0.070) | - | - | 23% |
| **[$Fe_4S_4$]$^{3+}$-Im** | toluene | 1 | 1/2 | (2.107, 2.029, 2.026) | **2.054** | (0.034, 0.028, 0.014) | | | 89% |
| | | 2 | 3/2 | (2.164, 1.980, 2.034) | **2.059** | (0.033, 0.014, 0.008) | 0.004 | 0.94 | 11% |

## Fitting of the HERFD-XAS Spectra

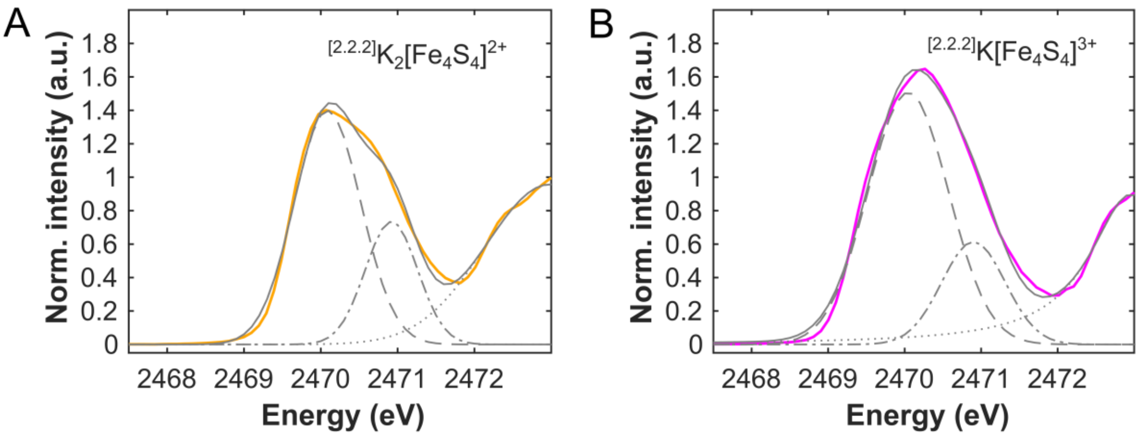


**Figure S11.** Normalized and fitted S K-edge HERFD-XAS spectra of $^{[2.2.2]}K_n[Fe_4S_4(DmpS)_4]$ ($n$=2,1; panels A and B, respectively) recorded on powdered samples at room-temperature *in vacuo*. The fitting contributions are shown as *grey lines*; the rising edge as a *dotted* one, the thiolate's contribution as a *dashed-dotted* one and the sulfide's contribution as a *dashed* one. The total fit is represented by a *solid grey line*. Refer to Table S2 for a compilation of selected parameters of the fits.

**Table S2.** Comparison of the fitted parameters for the covalencies of $^{[2.2.2]}K_n[Fe_4S_4(DmpS)_4]$ ($n$=1,2) with their K-ion encapsulated congeners, $K_n[Fe_4S_4(DmpS)_4$ ($n$=1,2). Refer to Figure S12 for a graphical comparison of the covalencies. Values for the $K^+$ ion encapsulated congeners were adapted from ref.[10].

| | sulfide[a] | | | | thiolate[b] | | | |
|---|---|---|---|---|---|---|---|---|
| **complex** | peak energy (eV) | intensity (a.u.) | dipole strength per bond ($D_0$)[c] | Cova-lency per bond ($\alpha^2$ in %)[a] | peak energy (eV) | intensity (a.u.) | dipole strength per bond ($D_0$)[d] | Cova-lency per bond ($\alpha^2$ in %)[b] |
| **K-[Fe$_4$S$_4$]$^{3+}$** | 2470.0±0.1 | 1.95±0.11 | 1.30±0.07 | **60±3** | 2470.8±0.1 | 0.75±0.03 | 1.50±0.06 | **56±3** |
| **$^{[2.2.2]}$K-[Fe$_4$S$_4$]$^{3+}$** | 2470.0±0.1 | 1.99±0.11 | 1.33±0.07 | **61±3** | 2470.8±0.1 | 0.65±0.03 | 1.29±0.06 | **49±3** |
| **K$_2$-[Fe$_4$S$_4$]$^{2+}$** | 2470.1±0.1 | 1.52±0.09 | 1.01±0.06 | **46±3** | 2470.9±0.1 | 0.59±0.03 | 1.19±0.06 | **44±3** |
| **$^{[2.2.2]}$K$_2$-[Fe$_4$S$_4$]$^{2+}$** | 2470.4±0.1 | 1.52±0.07 | 1.02±0.05 | **47±2** | 2470.9±0.1 | 0.63±0.02 | 1.26±0.04 | **47±2** |

[a] For the sulfide contribution, a transition dipole moment of 6.54 was used to calculate covalency, as determined by independent spectroscopic techniques in the literature on the infinite chain compounds $KFeS_2$ and $CsFeS_2$.[20] [b] For the thiolate contribution, a transition dipole moment of 8.05 was used to calculate covalency, as determined by independent spectroscopic techniques in the literature on the blue copper protein plastocyanine.[20, 21] [c] The renormalized dipole strength of the sulfide contribution (per bond) is obtained by multiplication of the intensity by a factor of 2/3. [d] The renormalized dipole strength of the thiolate contribution (per bond) is obtained by multiplication of the intensity by a factor of 2.

**Note S2.** The major distinction between the difference spectra of the $K^+$-containing and $K^+$-free variants of $[Fe_4S_4]^{2+}$ and $[Fe_4S_4]^{3+}$, respectively, is evidently in the region of *ca.* 2472.5 eV. This region harbours the S 1*s* → $\sigma$*(C-S) transition [22].

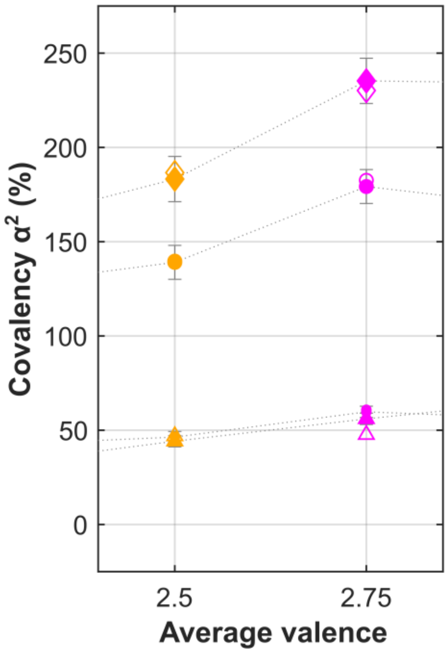


**Figure S12.** Variation of covalency, $\alpha^2$, and its individual contributions with the average valence of the Fe-atom in the $[Fe_4S_4]^{2+}$ and $[Fe_4S_4]^{3+}$ oxidation states in presence (*solid black markers*) and absence (*hollow markers*) of encapsulated $K^+$ ions. While the total $\alpha^2$ is shown as *diamonds*, the contributions arising from sulfide- and thiolate-bonding, are presented as *dot* and *triangle* shaped markers, respectively. *Dotted gray lines* are intended to guide the eye. Approximate error bars are shown in *grey* colour.

**Supplementary Mössbauer Spectra and Simulations**

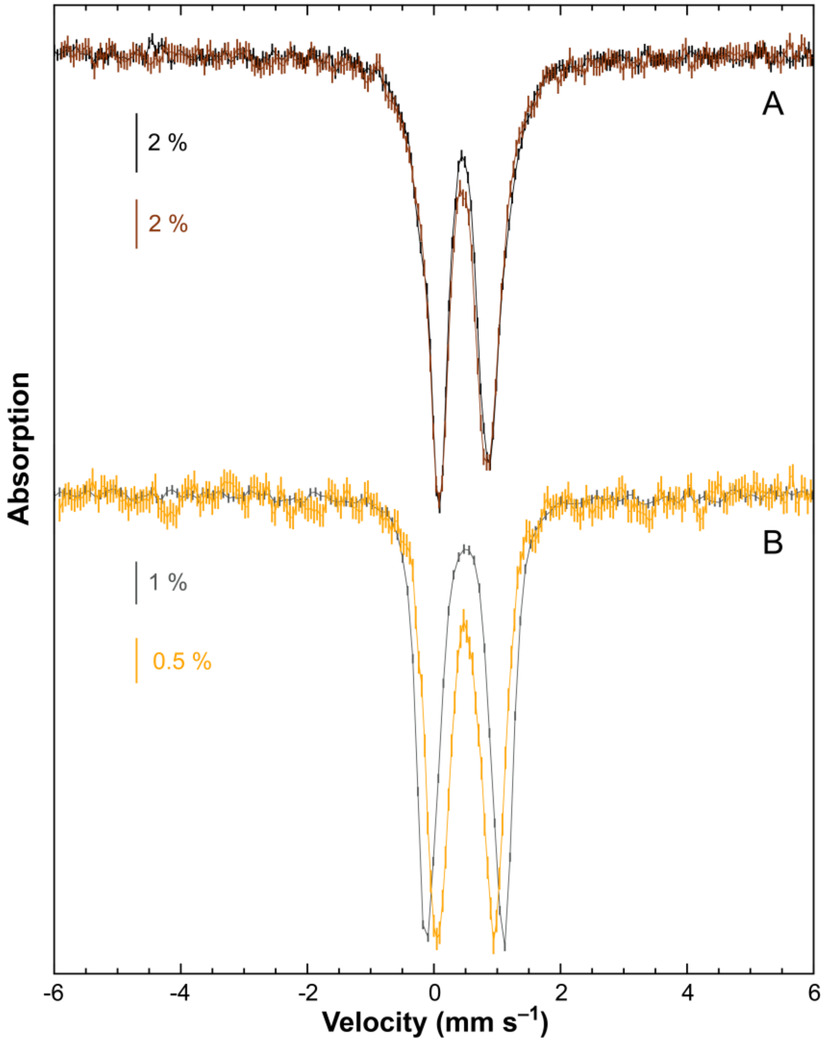


**Figure S13.** Mössbauer spectra (*vertical bars*) of the **$K_2$-[$Fe_4S_4$]$^{2+}$** (A) and **$^{[2.2.2]}K_2$-[$Fe_4S_4$]$^{2+}$** (B) clusters in powder recorded using a 0.06 T external magnetic field applied parallel to the *γ*-rays. No $^{57}$Fe-enrichment was performed. Spectra recorded at 5.8 K are drawn in *black* and *grey* on A and B, respectively. Those recorded at 80 K are displayed in *brown* and *yellow* on A and B, respectively.

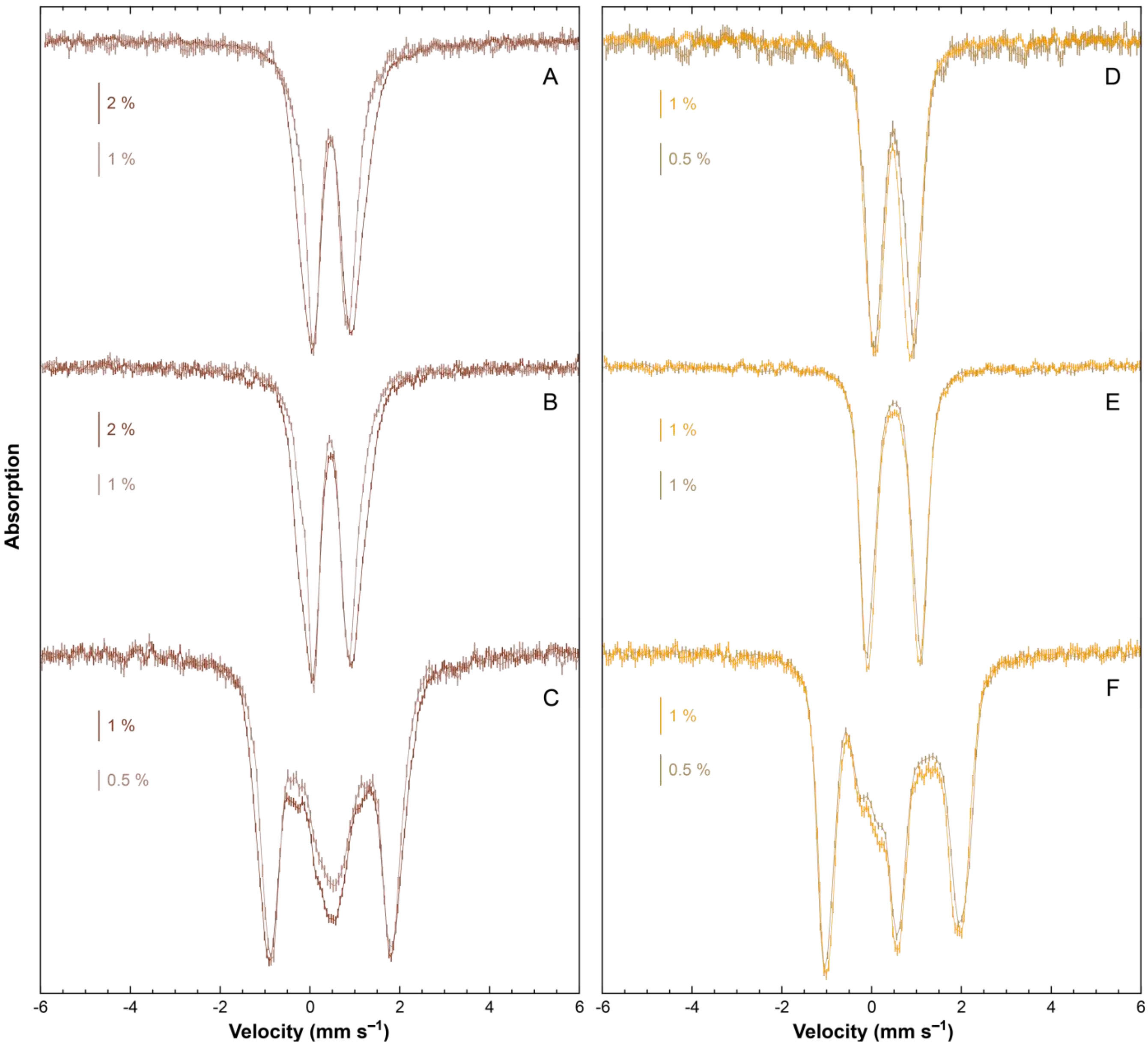


**Figure S14.** Mössbauer spectra (*vertical bars*) of the **$K_2$-[$Fe_4S_4$]$^{2+}$** (*left column*) and **[2.2.2]$K_2$-[$Fe_4S_4$]$^{2+}$** (*right column*) clusters. Powder spectra are displayed in ochre-brown (*left*) and ochre-yellow (*right*) whereas those for solutions are drawn in brown (*left*, solvent: toluene) and yellow (*right*, solvent: THF). Full $^{57}$Fe-enrichment was performed for solution samples. Recording conditions: Panels A and D: $T$=80 K and $B$=0.06 T; Panels B and E: $T$=5.8 K and $B$=0.06 T; Panels C and F: $T$=5.8 K and $B$=7 T. The external magnetic field $B$ was applied parallel to the $\gamma$-rays.

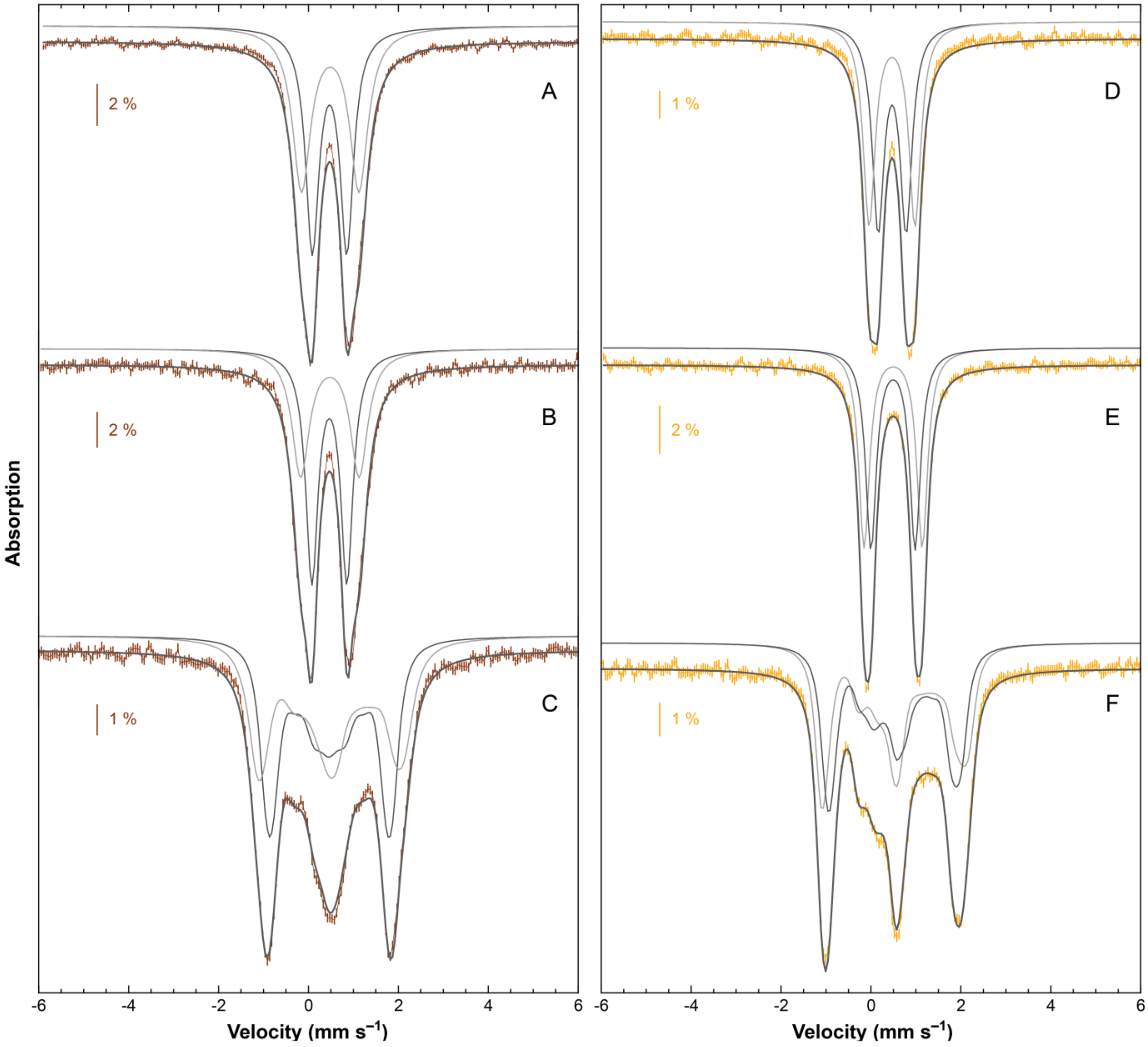


**Figure S15.** Mössbauer solution spectra (*vertical bars*) of the **$K_2$-[$Fe_4S_4$]$^{2+}$** in toluene (*left column*) and **$^{[2.2.2]}K_2$-[$Fe_4S_4$]$^{2+}$** in THF (*right column*) clusters. Recording conditions: panels A and D: *T*=80 K and *B*=0.06 T; panels B and E: *T*=5.8 K and *B*=0.06 T; panels C and F: *T*=5.8 K and *B*=7 T. The external magnetic field *B* was applied parallel to the *γ*-rays. Simulations are overlaid as brown solid thick lines. Two diamagnetic Fe sites in a 1:1 ratio were considered with the same linewidth for **$^{[2.2.2]}K_2$-[$Fe_4S_4$]$^{2+}$** and two different ones for **$K_2$-[$Fe_4S_4$]$^{2+}$**. Contributions are displayed above the data as thin *dark grey* and *light grey* lines. See Table 1 for the corresponding parameters. Fitting parameters are summarized in Table 1.

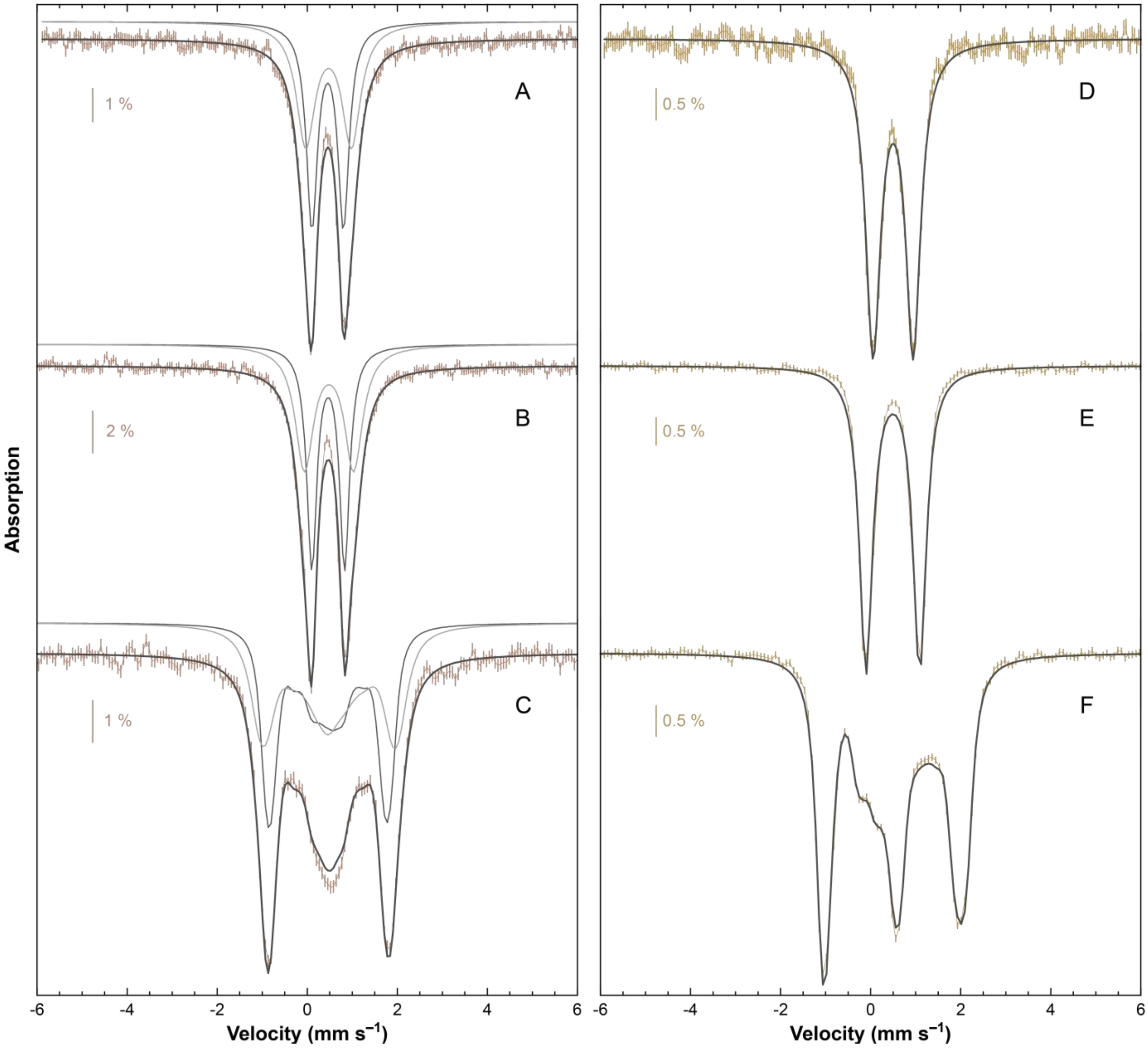


**Figure S16.** Mössbauer powder spectra (*vertical bars*) of the **$K_2$-[$Fe_4S_4$]$^{2+}$** (*left column*) and **$^{[2.2.2]}K_2$-[$Fe_4S_4$]$^{2+}$** (*right column*) clusters. Recording conditions: Panels A and D: *T*=80 K and *B*=0.06 T; Panels B and E: *T*=5.8 K and *B*=0.06 T; Panels C and F: *T*=5.8 K and *B*=7 T. The external magnetic field B was applied parallel to the γ-rays. Simulations are overlaid as brown solid thick lines. Two diamagnetic Fe sites in a 1:1 ratio were considered for **$K_2$-[$Fe_4S_4$]$^{2+}$** whereas a single one was assumed for **$^{[2.2.2]}K_2$-[$Fe_4S_4$]$^{2+}$**. Contributions for **$K_2$-[$Fe_4S_4$]$^{2+}$** are displayed as thin *grey* and *light grey*. See Table S3 for parameters.

**Table S3.** Parameters determined for the simulations of the powder spectra of the **$K_2$-[$Fe_4S_4$]$^{2+}$** and **$^{[2.2,2]}K_2$-[$Fe_4S_4$]$^{2+}$** clusters shown in Figure S16. Diamagnetic sites were considered. Values for the 80 K spectra are indicated in parentheses.

| parameter | **$K_2$-[$Fe_4S_4$]$^{2+}$** | | **$^{[2.2.2]}K_2$-[$Fe_4S_4$]$^{2+}$** |
|---|---|---|---|
| | Site 1 (*dark grey*) | Site 2 (*light grey*) | Site 1 |
| $\delta$ (mm s$^{-1}$) | 0.46 (0.45) | 0.48 (0.47) | 0.49 (0.50) |
| $\Delta E_Q$ (mm s$^{-1}$) | 0.73 (0.70) | 1.09 (1.01) | 1.19 (0.89) |
| $\eta$ | 0.9 (-) | 1.1 (-) | 0.5 (-) |
| $\Gamma_{fwhm}$ (mm s$^{-1}$) | 0.27 (0.30) | 0.49 (0.50) | 0.35/0.30[a] (0.40) |

[a] The first and second values are for the 0.06 and 7 T spectra, respectively.

**Table S4.** Parameters determined for the simulations of the solution spectra of the **$^{[2.2.2]}K_2$-[$Fe_4S_4$]$^{2+}$** cluster in THF shown in Figure S17. A single diamagnetic site was considered. Values for the 80 K spectra are indicated in parentheses.

| parameter | **$^{[2.2.2]}K_2$-[$Fe_4S_4$]$^{2+}$** |
|---|---|
| | Site 1 |
| $\delta$ (mm s$^{-1}$) | 0.49 (0.47) |
| $\Delta E_Q$ (mm s$^{-1}$) | 1.14 (0.82) |
| $\eta$ | 0.5 (-) |
| $\Gamma_{fwhm}$ (mm s$^{-1}$) | 0.36/0.32[a] (0.42) |

[a] The first and second values are associated to the low- and high-field spectra, respectively.

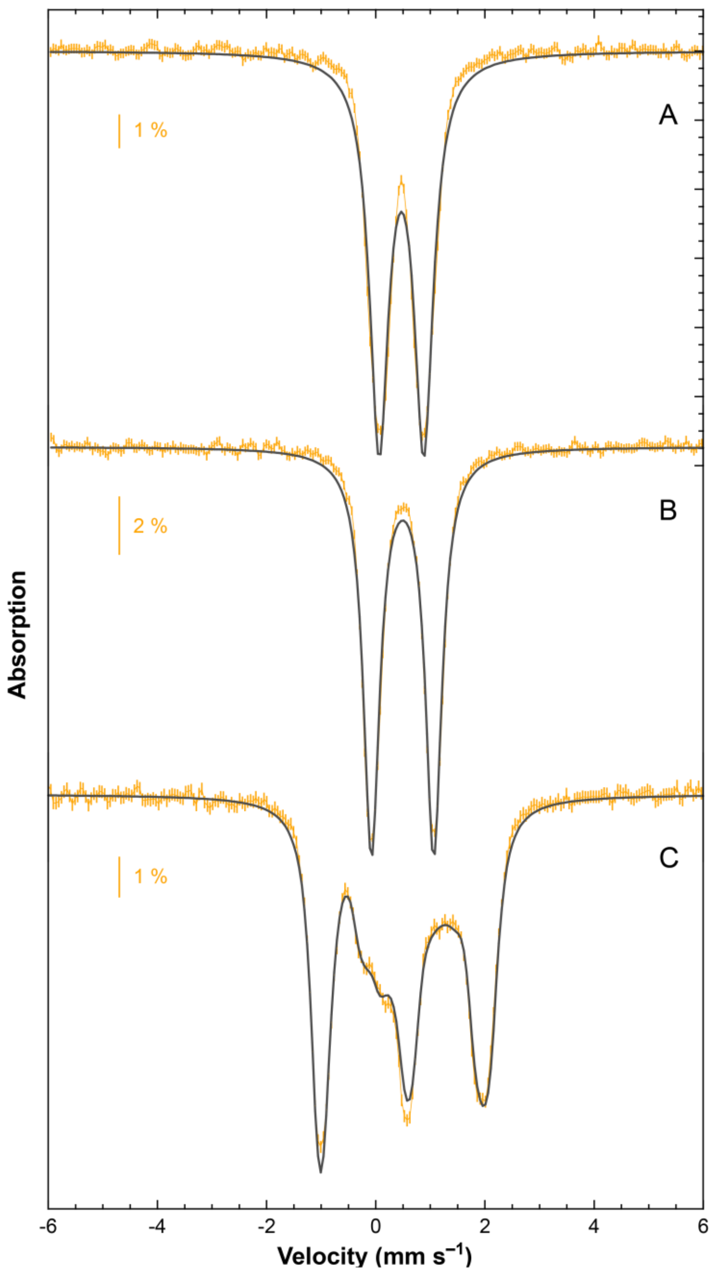


**Figure S17.** Mössbauer solution spectra (*vertical bars*) of the **[2.2.2]$K_2$-[$Fe_4S_4$]$^{2+}$** cluster in THF. Recording conditions: Panel A: *T*=80 K and *B*=0.06 T; Panel B: *T*=5.8 K and *B*=0.06 T; Panel C: *T*=5.8 K and *B*=7 T. The external magnetic field *B* was applied parallel to the γ-rays. Simulations assuming a single Fe site are overlaid as *grey solid thick lines*. See Table S4 for parameters.

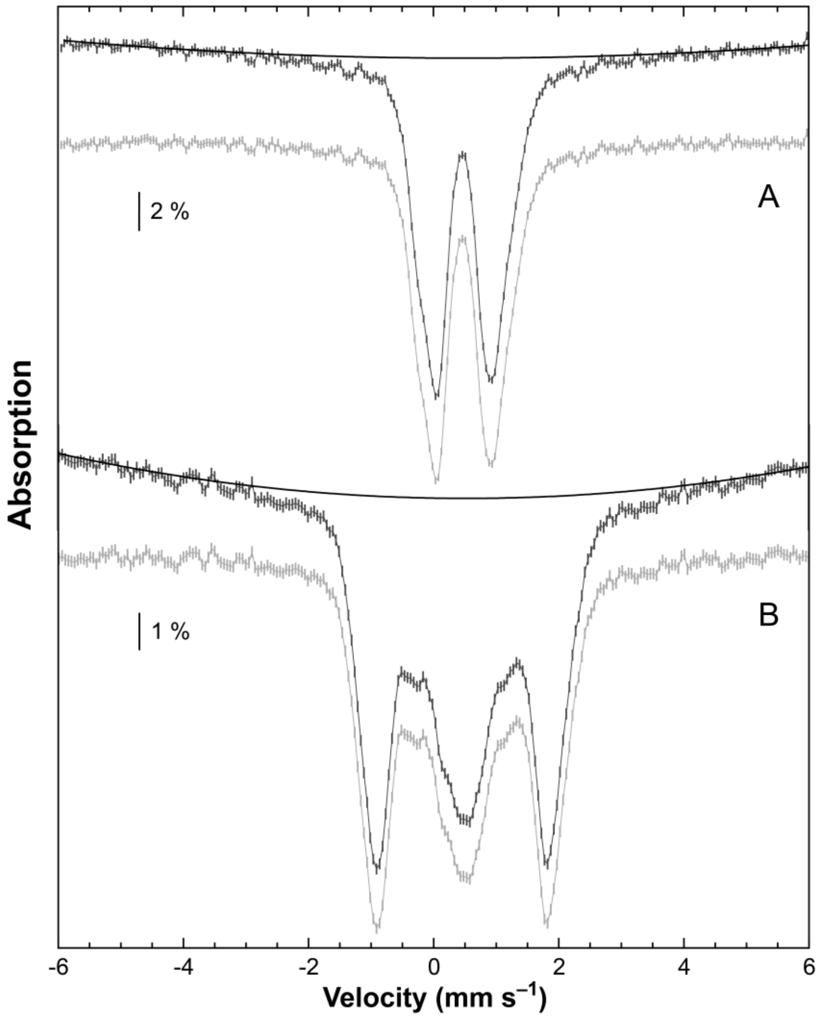


**Figure S18.** 6 K Mössbauer solution spectra (*black vertical bars*) of the $\mathbf{K_2\text{-}[Fe_4S_4]^{2+}}$ cluster dissolved in toluene. The external magnetic field of 0.06 T (A) and 7 T (B) was applied parallel to the *γ*-rays. The parabola correction was calculated on points corresponding to velocities below –1.5 mm s$^{-1}$ and above +2.4 mm s$^{-1}$ and is indicated in black. The resulting spectra are displayed in *grey*.

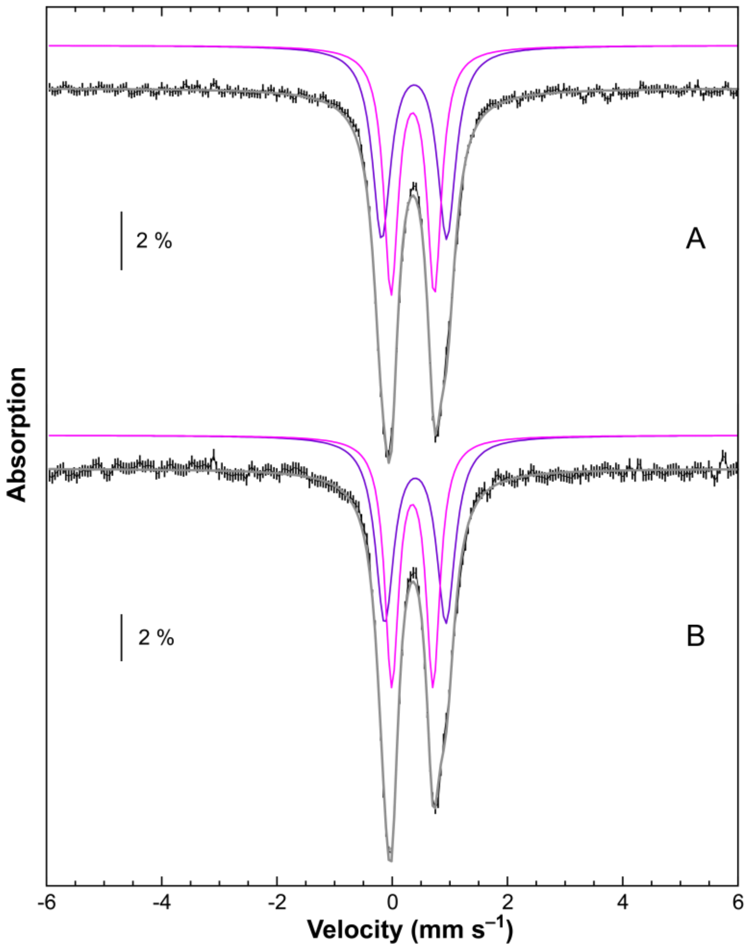


**Figure S19.** 80 K Mössbauer spectra (vertical bars) of the 1.8 mM toluene solution of $^{57}$Fe-enriched **K-[Fe$_4$S$_4$]$^{3+}$** (A) and of the 1.6 mM THF solution of $^{57}$Fe-enriched **$^{[2.2.2]}$K-[Fe$_4$S$_4$]$^{3+}$** (B) recorded using a 0.06 T external magnetic field applied parallel to the *γ*-rays. Simulations are overlaid as *grey* thick solid lines. Two Fe sites in a 1:1 ratio were considered. Contributions are displayed above the data as thin *magenta* and *purple* lines. See Table S5 for the corresponding parameters.

**Table S5.** Sets of parameters used to simulate the 80 K Mössbauer spectra displayed in Figure S19. Two equally contributing Fe sites were considered, and two different linewidths were considered for the two doublets.

| | parameter | **K-[Fe$_4$S$_4$]$^{3+}$** | **$^{[2.2.2]}$K-[Fe$_4$S$_4$]$^{3+}$** |
|---|---|---|---|
| Site 1 (*magenta*) | $\delta$ (mm s$^{-1}$) | 0.35 | 0.35 |
| | $\Delta E_Q$ (mms$^{-1}$) | 0.75 | 0.72 |
| | $\Gamma_{fwhm}$ (mm s$^{-1}$) | 0.30 | 0.29 |
| Site 2 (*purple*) | $\delta$ (mm s$^{-1}$) | 0.38 | 0.40 |
| | $\Delta E_Q$ (mms$^{-1}$) | 1.13 | 1.06 |
| | $\Gamma_{fwhm}$ (mm s$^{-1}$) | 0.39 | 0.39 |

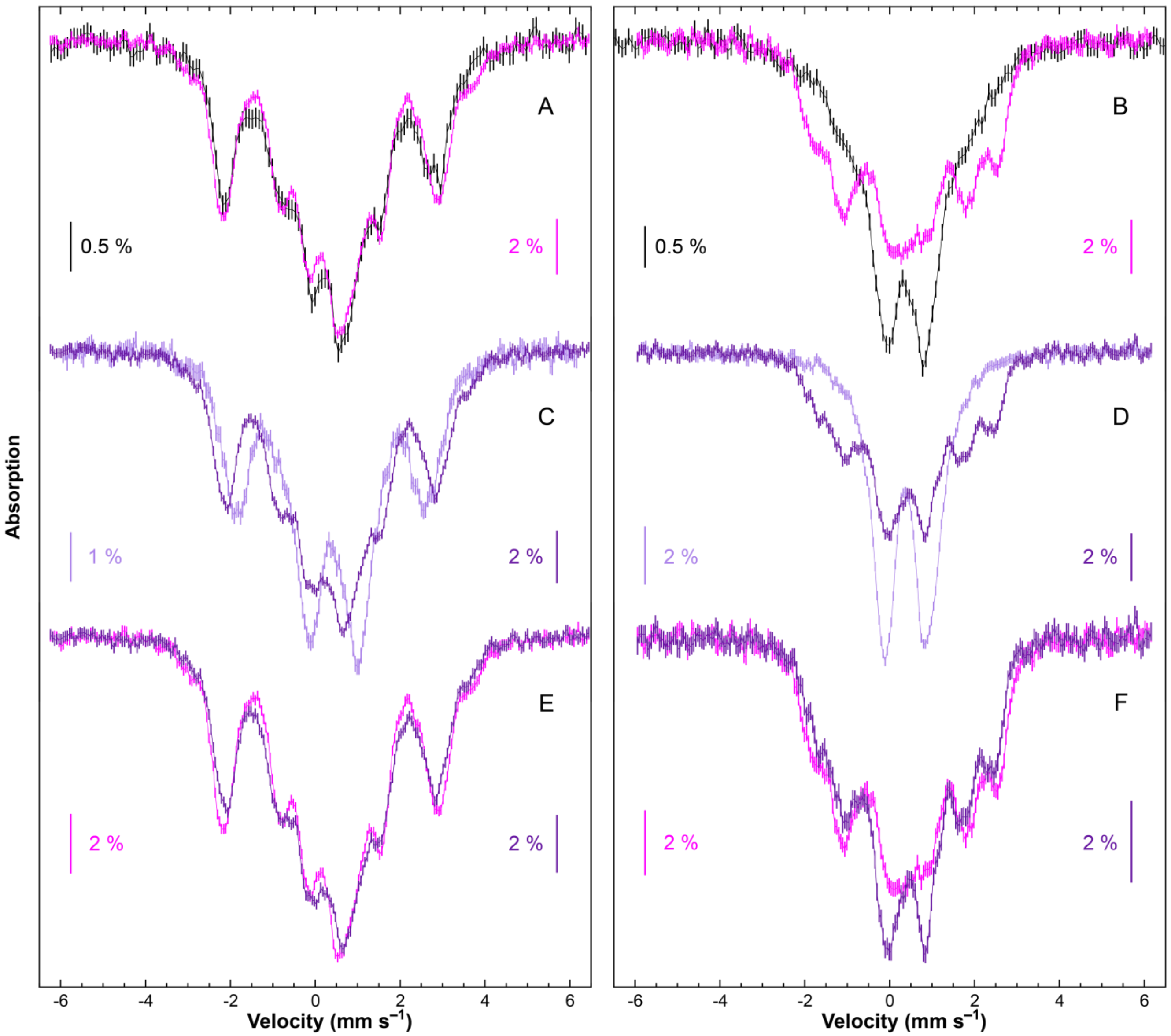


**Figure S20.** Comparisons of experimental 6 K Mössbauer spectra (*vertical bars*) using a 7 T (*left* column, panels A, C and E) and 0.06 T (*right* column, panels B, D and F) external magnetic field applied parallel to the γ-rays. The two spectra on each panel were scaled to the same area. (A-B) Spectra recorded on a powder (*black*) and 1.6 mM THF solution (*magenta*) samples of $^{[2.2.2]}$**K-[Fe$_4$S$_4$]$^{3+}$**. (C-D) Spectra recorded on a powder (*light purple*) and 1.8 mM toluene solution (*dark purple*) samples of **K-[Fe$_4$S$_4$]$^{3+}$**. (E-F) Comparison of the two solution-sample spectra displayed in panels A and C (resp. B and D) for panel E (resp. F).

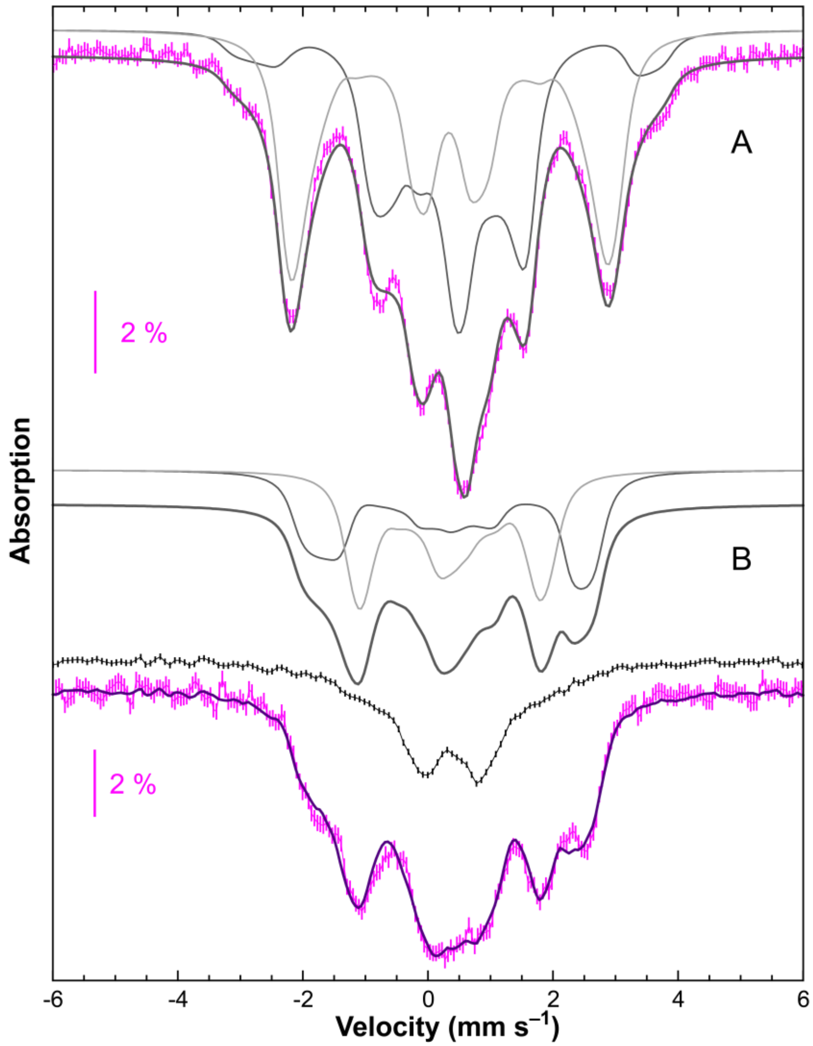


**Figure S21.** (A) Experimental (*magenta vertical bars*) 6 K high-field Mössbauer spectrum of the 1.6 mM THF solution of **$^{[2.2.2]}$K-[$Fe_4S_4$]$^{3+}$** displayed in panel A of Figure S20. The simulation is overlaid as a *grey* solid thick line and parameters are listed in Table S6. The two contributions are displayed above the data as *grey* and *dark grey* thin solid traces. (B) Experimental (*magenta vertical bars*) 6 K low-field Mössbauer spectrum of the 1.6 mM THF solution of **$^{[2.2.2]}$K-[$Fe_4S_4$]$^{3+}$** displayed in panel B of Figure S20. The theoretical spectrum, calculated using the parameters determined from the simulation at 7 T and scaled at 70 % of the area of the experimental low-field spectrum, is displayed as a *grey* solid thick line with contributions displayed above the data (same color code as in panel A). To reproduce the experimental low-field spectrum, the powder 6 K low-field spectrum shown in panel B of Figure S20 (*black vertical bars*) and scaled to 30 % of the area of the solution experimental spectrum was added. The sum is displayed as a *purple* solid thick line overlaid on the experimental spectrum.

**Table S6.** List of parameters determined upon simulating the 6 K high-field spectrum of the THF solution of the **$^{[2.2.2]}$K-[Fe$_4$S$_4$]$^{3+}$** cluster. Two Fe sites in a 1:1 ratio were considered with a $S$ = 1/2 spin state in the slow relaxation regime. A unique linewidth was assumed.

| parameter | Site 1 (*light grey*) | Site 2 (*dark grey*) |
|---|---|---|
| $\delta$ (mm s$^{-1}$) | 0.35 | 0.43 |
| $\Delta E_Q$ (mm s$^{-1}$) | –0.93 | –1.12 |
| $\eta$ | 0.8 | 1.0 |
| $A_k$ / ($g_n$ $\mu_n$) ($k$ = $x$, $y$, $z$) (T) | 19 | –20 |
| | 12 | –23 |
| | 12 | –26 |
| $\Gamma_{fwhm}$ (mm s$^{-1}$) | 0.36 | 0.36 |

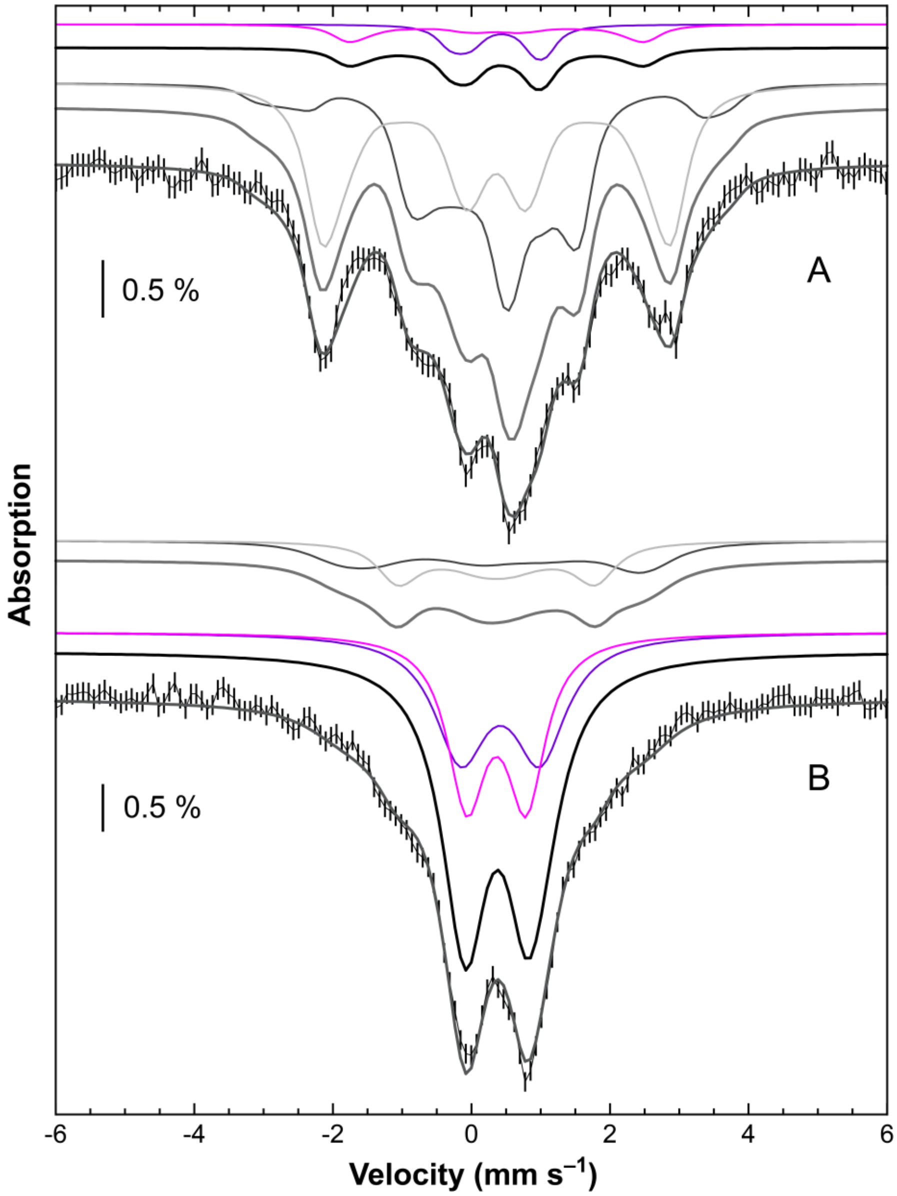


**Figure S22.** Experimental (*black vertical bars*) 5-6 K Mössbauer powder spectrum of $^{[2.2.2]}$**K-[Fe$_4$S$_4$]$^{3+}$** recorded using a 7 T (A) and 0.06 T (B) magnetic field applied along the $\gamma$-ray's direction. The simulations are overlaid as a *grey solid thick line* and parameters are listed in Table S7. A *S*=1/2 spin was considered. Two 1:1 slow relaxing and two 1:1 fast relaxing sites were considered. The same parameter set was considered for the first (respectively second) site of each system. The slow relaxing contributions are shown in *grey* (*medium grey thick line* for the sum, *dark* and *light grey thin lines* for each site) above the spectra whereas the fast ones are reproduced in *color* (*black thick line* for the sum, *magenta* and *purple thin lines* for each site).

**Table S7.** List of parameters determined upon simulating the 6 K powder spectra of **$^{[2.2.2]}$K-[$Fe_4S_4$]$^{3+}$** when considering a $S$=1/2 ground state.

| parameter | Site 1 | | Site 2 | |
|---|---|---|---|---|
| $\delta$ (mm s$^{-1}$) | 0.36 | | 0.42 | |
| $\Delta E_Q$ (mm s$^{-1}$) | –0.85 | | –1.14 | |
| $\eta$ | 1.0 | | 0.5 | |
| $A_k$ / ($g_n$ $\mu_n$) ($k$ = $x$, $y$, $z$) (T) | 17 | | –18 | |
| | 13 | | –23 | |
| | 13 | | –25 | |
| $\Gamma_{fwhm}$ (mm s$^{-1}$) | 0.92/0.52[a] | | 0.67/0.40[a] | |
| relaxation | slow | fast | slow | fast |
| color | *light grey* | *magenta* | *dark grey* | *purple* |
| contribution (%) | 17/48[a] | 37/4[a] | 17/48[a] | 37/4[a] |

[a] The two values are for the spectra recorded at 0.06 and 7 T external magnetic field, respectively.

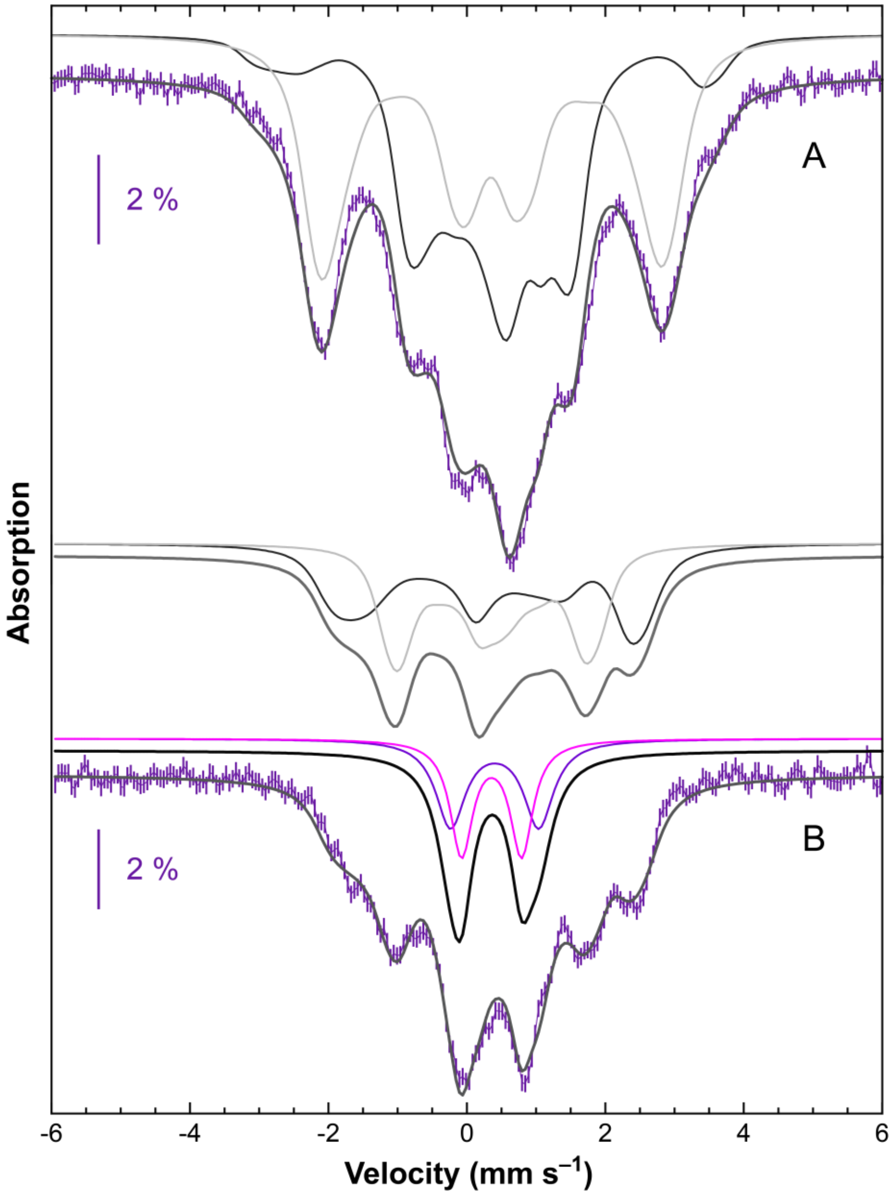


**Figure S23.** Experimental (*dark purple vertical bars*) 5-6 K Mössbauer toluene solution spectrum of **K-[$Fe_4S_4$]$^{3+}$** recorded using a 7 T (A) and 0.06 T (B) magnetic field applied along the *γ*-rays' direction. The simulations are overlaid as a *grey solid thick line* and parameters are listed in Table S8. A *S*=1/2 spin was considered. Two 1:1 slow relaxing and two 1:1 fast relaxing sites were considered at 0.06 T whereas only the former system was considered at 7 T. The same parameter set was considered for the first (respectively second) site of each system. The slow relaxing contributions are shown in *grey* (*medium grey thick line* for the sum, *dark* and *light grey thin lines* for each site) above the spectra whereas the fast ones are reproduced in *color* (*black thick line* for the sum, *magenta* and *purple thin lines* for each site).

**Table S8.** List of parameters determined upon simulating the 6 K toluene solution spectra of **K-[$Fe_4S_4$]$^{3+}$** when considering a $S$=1/2 ground state.

| parameter | Site 1 | | Site 2 | |
|---|---|---|---|---|
| $\delta$ (mm s$^{-1}$) | 0.36 | | 0.40 | |
| $\Delta E_Q$ (mm s$^{-1}$) | –0.86 | | –1.27 | |
| $\eta$ | 0.8 | | 0.0 | |
| $A_k$ / ($g_n$ $\mu_n$) ($k$ = $x$, $y$, $z$) (T) | 19 | | –17 | |
| | 12 | | –23 | |
| | 11 | | –23 | |
| $\Gamma_{fwhm}$ (mm s$^{-1}$) | 0.52/0.50[a] | | 0.39/0.42[a] | |
| relaxation | slow | fast | slow | fast |
| color | *light grey* | *magenta* | *dark grey* | *purple* |
| contribution (%) | 37/52[a] | 15/0[a] | 37/52[a] | 15/0[a] |

[a] The two values are for the spectra recorded at 0.06 and 7 T external magnetic field, respectively.

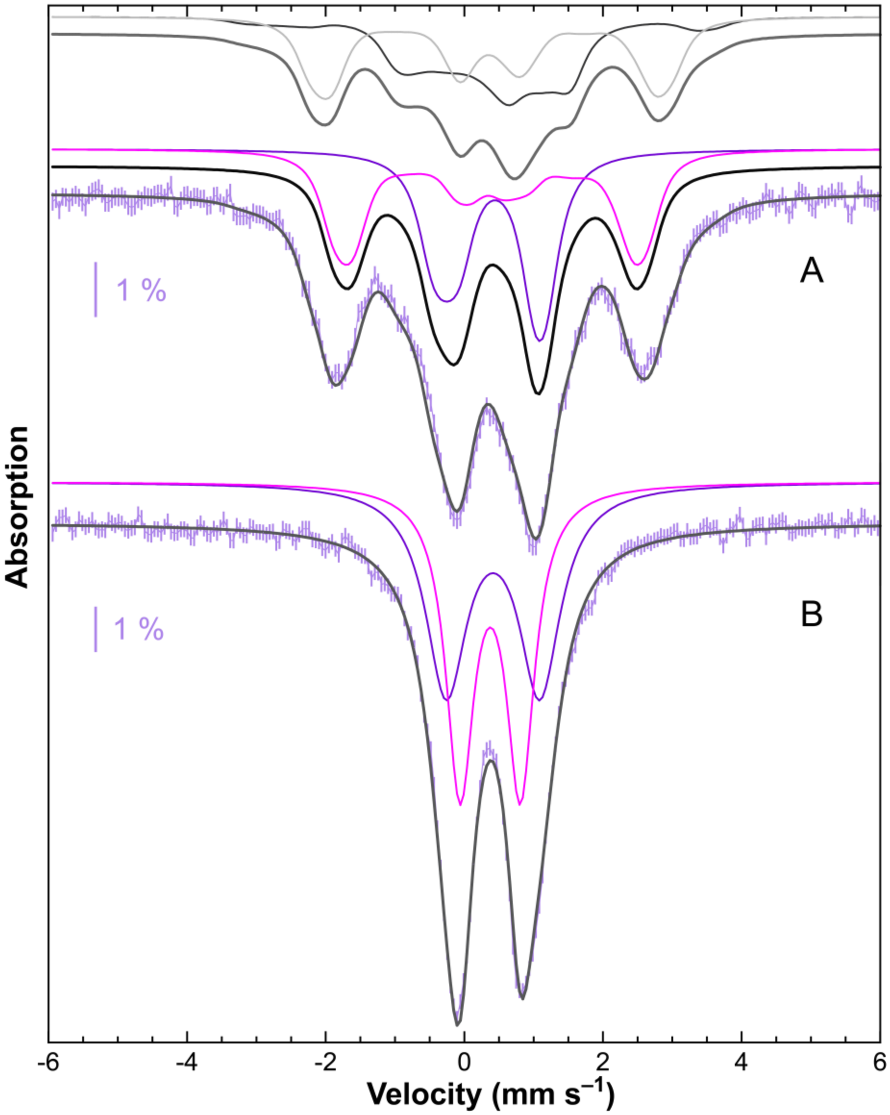


**Figure S24.** Experimental (*light purple vertical bars*) 5-6 K Mössbauer powder spectrum of **K-[$Fe_4S_4$]$^{3+}$** recorded using a 7 T (A) and 0.06 T (B) magnetic field applied along the $\gamma$-rays' direction. The simulations are overlaid as a *grey solid thick line* and parameters are listed in Table S9. A $S$=1/2 spin was considered. Two 1:1 slow relaxing and two 1:1 fast relaxing sites were considered at 0.06 T whereas only the latter system was considered at 7 T. The same parameter set was considered for the first (respectively second) site of each system. The slow relaxing contributions are shown in *grey* (*medium grey thick line* for the sum, *dark* and *light grey thin lines* for each site) above the spectra whereas the fast ones are reproduced in *color* (*black thick line* for the sum, *magenta* and *purple thin lines* for each site).

**Table S9.** List of parameters determined upon simulating the 6 K powder spectra of **K-[$Fe_4S_4$]$^{3+}$** when considering a $S$=1/2 ground state.

| parameter | Site 1 | | Site 2 | |
|---|---|---|---|---|
| $\delta$ (mm s$^{-1}$) | 0.37 | | 0.41 | |
| $\Delta E_Q$ (mm s$^{-1}$) | –0.87 | | –1.35 | |
| $\eta$ | 0.7 | | 0.4 | |
| $A_k$ / ($g_n$ $\mu_n$) ($k$ = $x$, $y$, $z$) (T) | 14 | | –17 | |
| | 16 | | –21 | |
| | 15 | | –25 | |
| $\Gamma_{fwhm}$ (mm s$^{-1}$) | 0.49/0.41[a] | | 0.72/0.47[a] | |
| relaxation | slow | fast | slow | fast |
| color | *light grey* | *magenta* | *dark grey* | *purple* |
| contribution (%) | 0/23[a] | 52/28[a] | 0/23[a] | 52/28[a] |

[a] The two values are for the spectra recorded at 0.06 and 7 T external magnetic field, respectively.

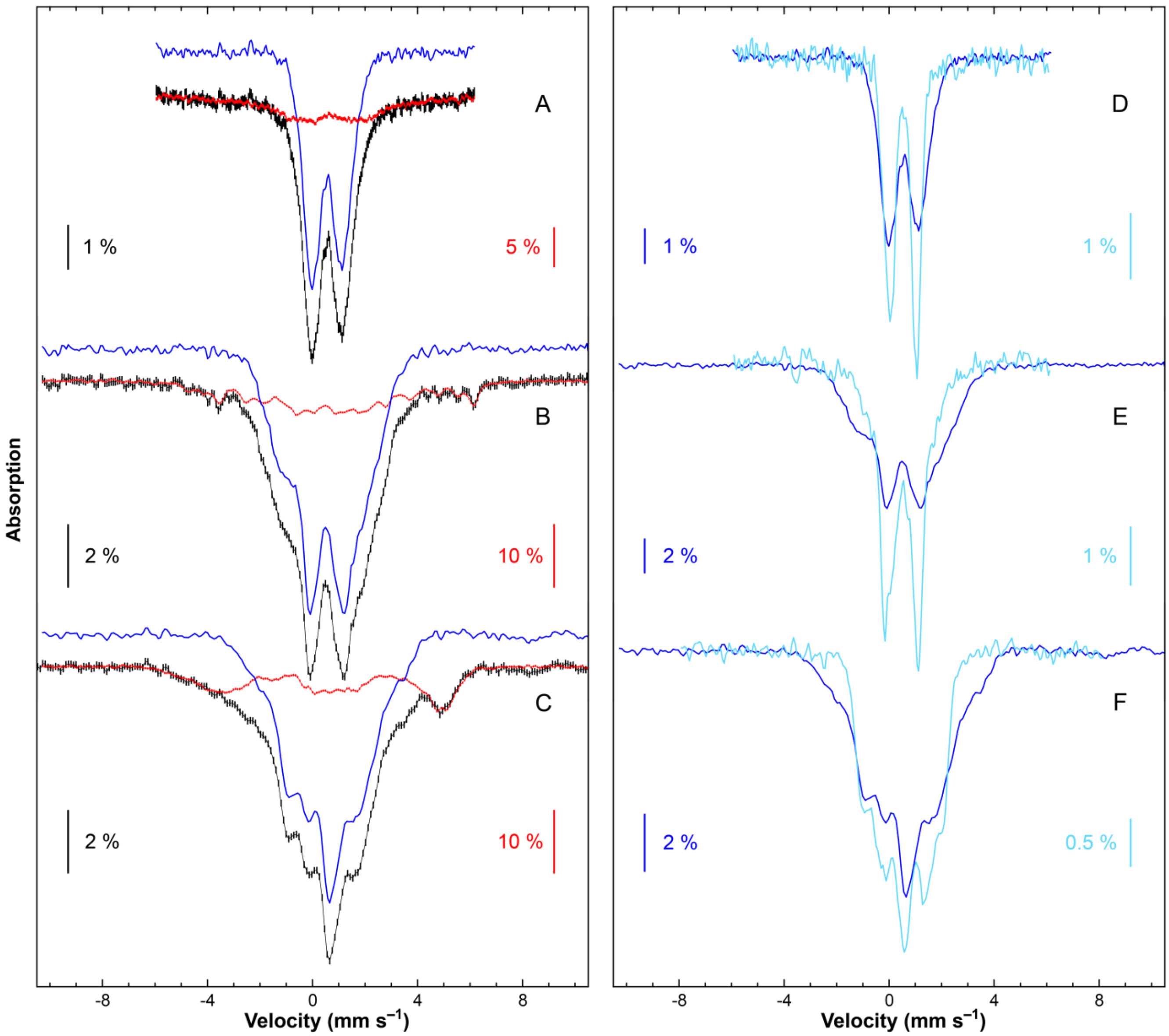


**Figure S25.** (*Left*) Experimental spectra recorded on a 1.4 mM THF solution of **$K_3$-[$Fe_4S_4$]$^{1+}$** (*black vertical bars*) at 80 (panel A) and 4-6 K (panels B and C) using a 0.06 (panels A and B) and 7 T (panel C) external magnetic field applied along the *γ*-ray's direction. Spectra of the 1.4 mM THF solution of the $K_4[Fe_4S_4(DmpS)_4]$ compound were recorded on similar conditions (*red vertical bars*). The $[Fe_4S_4]^0$ spectra are displayed scaled to the contribution observed in those of $[Fe_4S_4]^{1+}$. The differences are displayed above as *blue* traces. (*Right*) The spectra of the 1.4 mM THF solution of **$K_3$-[$Fe_4S_4$]$^{1+}$** (*blue* traces, same as in the *left* column) are compared to those recorded on a powder sample (*light blue* traces). The contributions of the $[Fe_4S_4]^0$ cluster were removed here as well. Recording conditions in panels D, E and F are identical to those of panels A, B, and C, respectively.

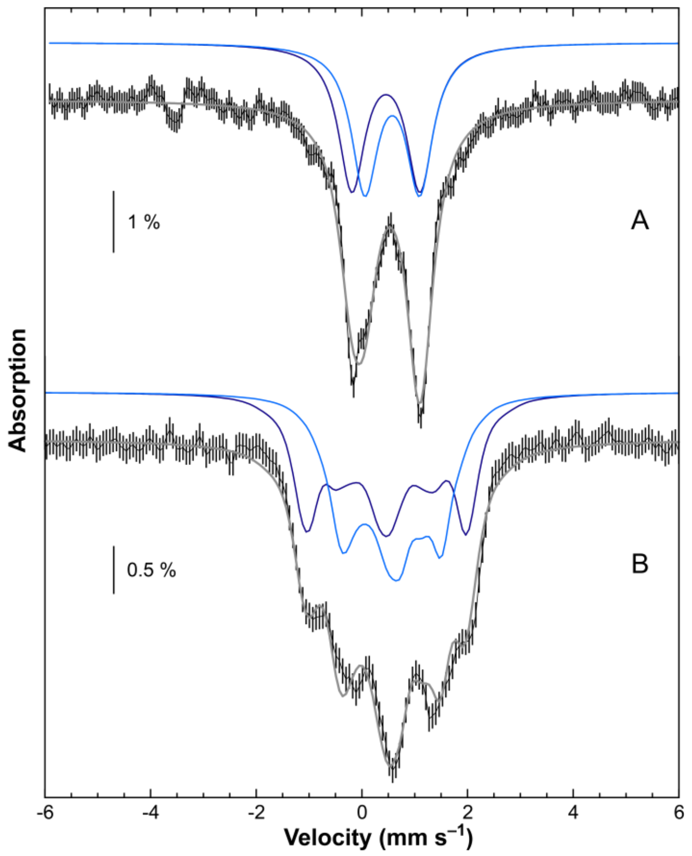


**Figure S26.** Experimental (*vertical bars*) spectra recorded on a powder sample **$K_3$-[$Fe_4S_4$]$^{1+}$** at 4.2 K (A) and 5.8 K (B) upon applying a 0.06 (A) and 7 T (B) external magnetic field parallel to the *γ*-beam. Spectra were corrected from the contribution of the all-ferrous cluster (see panels E and F in Figure S25). Tentative simulations are overlaid as thick solid *grey* lines. They were obtained considering two iron sites in a 1:1 ratio (same linewidth) with a *S*=3/2 state. Zero-field parameters issued from EPR studies were considered (*vide supra*). Isotropic hyperfine interactions were assumed. Contributions are displayed above the spectra and parameters listed in Table S10.

**Table S10.** Parameters used to reproduce the Mössbauer spectra shown in Figure S26. The two iron sites present a *S*=3/2 state with ZFS parameters fixed to those determined by EPR (Figure S8): $D$ = –1.3 cm$^{-1}$ and $E/D$ = 0.07. The *g*-values were fixed to 2.0. The relaxation regime is slow at high-field and fast at low-field.

| parameter | Site 1 (*dark blue*) | Site 2 (*light blue*) |
|---|---:|---:|
| $\delta$ (mm s$^{-1}$) | 0.46 | 0.58 |
| $\Delta E_Q$ (mm s$^{-1}$) / $\eta$ | –1.29 / 0.8 | –1.04 / 0.3 |
| Euler angles ZFS *vs*. EFG (°) | –180 / 90 / 0 | –90 / –90 / 0 |
| $A_{iso}$ / ($g_n$ $\mu_n$) (T) | –9.3 | –2.6 |
| $\Gamma_{fwhm}$ (mm s$^{-1}$)[a] | 0.61 / 0.46[a] | |

[a] The first and second values are associated to the low- and high-field spectra, respectively. The same linewidth was considered for the two sites.

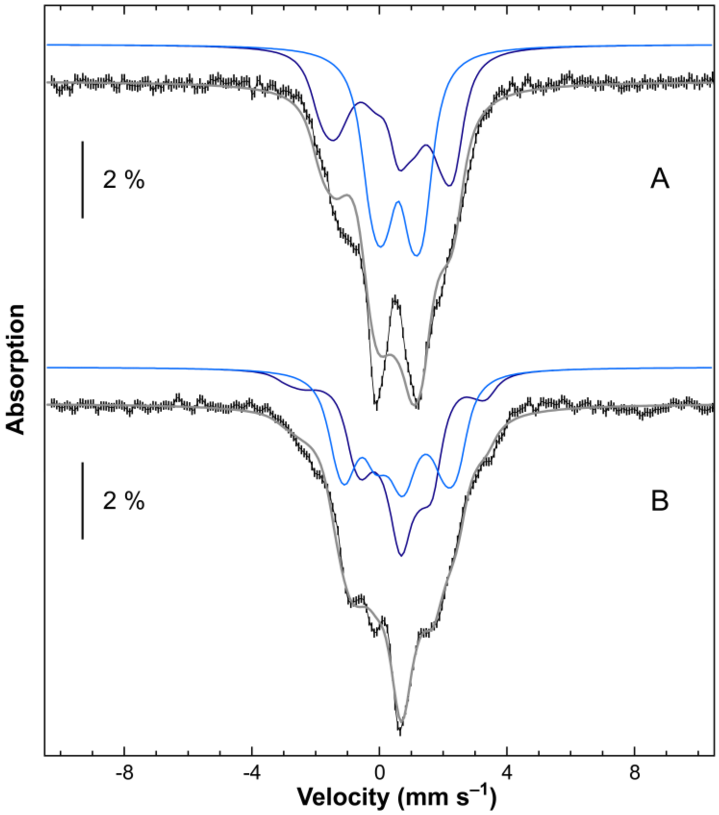


**Figure S27.** Experimental (vertical bars) spectra recorded on the 1.4 mM THF solution sample of **$K_3$-[$Fe_4S_4$]$^{1+}$** at 5.8 K upon applying a 0.06 (A) and 7 T (B) external magnetic field parallel to the *γ*-beam. Spectra were corrected from the contribution of the all-ferrous cluster (see panels E and F in Figure S25). A tentative simulation of the high-field spectrum is overlaid as thick solid *grey* line. It was obtained considering two iron sites in a 1:1 ratio (same linewidth) with a *S*=1/2 state in the slow relaxation regime. Contributions are displayed above the spectrum and parameters listed in Table S11. The theoretical low-field spectrum (thick *grey* solid trace) was calculated using the same parameters.

**Table S11.** Parameters used to reproduce the Mössbauer spectra shown in Figure S27. The two equally contributing iron sites present a *S*=1/2 state in the slow relaxation regime with *g*-values fixed to 2.0.

| parameter | Site 1 (*dark blue*) | Site 2 (*light blue*) |
|---|---|---|
| $\delta$ (mm s$^{-1}$) | 0.58 | 0.59 |
| $\Delta E_Q$ (mm s$^{-1}$) / $\eta$ | 1.17 / 1.0 | –1.04 / 0.3 |
| $A_k$ / ($g_n$ $\mu_n$) ($k$ = $x$, $y$, $z$) (T) | –20.8 / – 25.9 / –6.5 | 0.6 / 6.1 / 3.9 |
| $\Gamma_{fwhm}$ (mm s$^{-1}$) | 0.71 | |

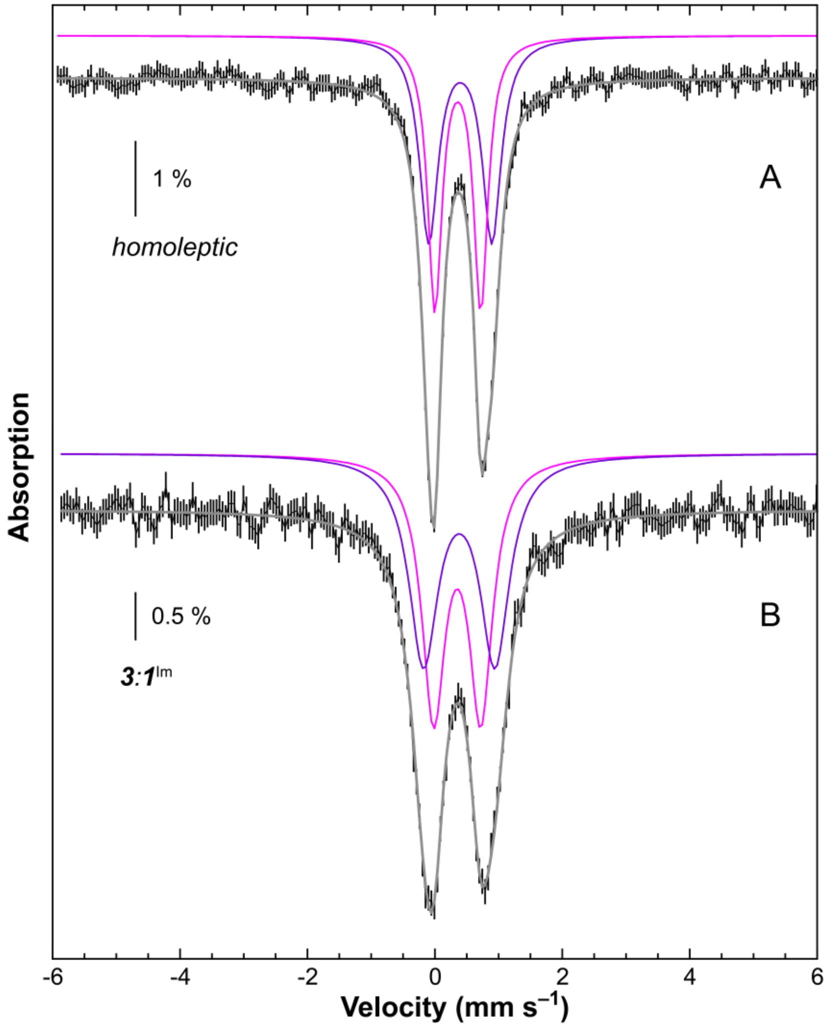


**Figure S28.** 80 K experimental spectra (*black* vertical bars) recorded on a powder sample of $^{[2.2.2]}$**K-[Fe$_4$S$_4$]$^{3+}$** (A) and **[Fe$_4$S$_4$]$^{3+}$-Im** (B) upon applying a 0.06 T external magnetic field along the $\gamma$-ray's direction. Simulations considering two equally contributing doublets are overlaid as thick solid *grey* lines. Contributions are displayed above as thin solid lines. Parameters are listed in Table S12.

**Table S12.** Parameters associated to the simulations displayed in Figure S28.

| | parameter | $^{[2.2.2]}$**K-[Fe$_4$S$_4$]$^{3+}$** | **[Fe$_4$S$_4$]$^{3+}$-Im** |
|---|---|---|---|
| Site 1 (*magenta*) | $\delta$ (mm s$^{-1}$) | 0.36 | 0.35 |
| | $\Delta E_Q$ (mms$^{-1}$) | 0.72 | 0.74 |
| | $\Gamma_{fwhm}$ (mm s$^{-1}$) | 0.27 | 0.45 |
| Site 2 (*purple*) | $\delta$ (mm s$^{-1}$) | 0.40 | 0.38 |
| | $\Delta E_Q$ (mms$^{-1}$) | 0.99 | 1.12 |
| | $\Gamma_{fwhm}$ (mm s$^{-1}$) | 0.36 | 0.56 |
| | av. $\delta$ (mm s$^{-1}$) | 0.38 | 0.37 |

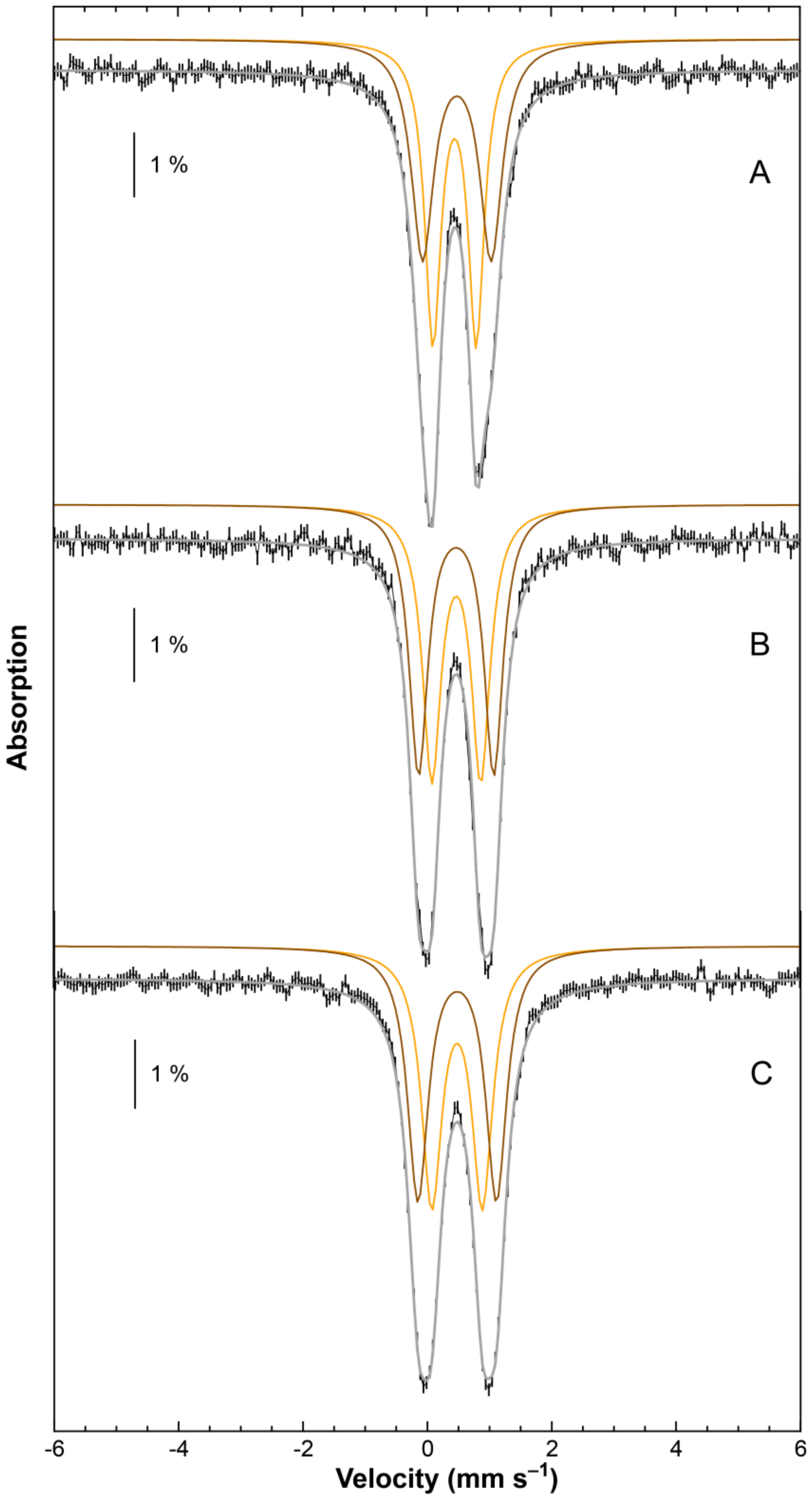


**Figure S29.** Experimental zero-field 80 K Mössbauer powder spectra (*black vertical bars*) recorded on powder samples of K[$Fe_4S_4$(DmpS)$_3$(Im*)] (**K-[$Fe_4S_4$]$^{2+}$-Im**) (A), $^{[2.2.2]}$K[$Fe_4S_4$(DmpS)$_3$(Im*)] (**$^{[2.2.2]}$K-[$Fe_4S_4$]$^{2+}$-Im**) (B) and $^{(18\text{-}C\text{-}6)}$K[$Fe_4S_4$(DmpS)$_3$(Im*)] (**$^{(18\text{-}C\text{-}6)}$K-[$Fe_4S_4$]$^{2+}$-Im**) (C). Simulations assuming two diamagnetic Fe sites in a 1:1 ratio are overlaid as *grey* thick solid lines. The two contributions are displayed above the data as *yellow* and *brown* thin traces. Parameters are listed in Table S13.

**Table S13.** Parameters of the two equally contributing diamagnetic Fe sites considered to reproduce the experimental 80 K spectra of powder samples of the *X*[$Fe_4S_4$(DmpS)$_3$(Im*)] clusters (Figure S29). A common linewidth was assumed, except for **K-[$Fe_4S_4$]$^{2+}$-Im** ([a]).

| *X* | | $K^+$ | $^{[2.2.2]}K^+$ | $^{(18\text{-}C\text{-}6)}K^+$ |
|---|---|---|---|---|
| Site 1 (*yellow*) | $\delta$ (mm s$^{-1}$) | 0.45 | 0.47 | 0.48 |
| | $\Delta E_Q$ (mms$^{-1}$) | 0.70 | 0.79 | 0.81 |
| Site 2 (*brown*) | $\delta$ (mm s$^{-1}$) | 0.48 | 0.47 | 0.48 |
| | $\Delta E_Q$ (mms$^{-1}$) | 1.10 | 1.21 | 1.26 |
| | $\Gamma_{fwhm}$ (mm s$^{-1}$) | 0.32/0.43[a] | 0.36 | 0.41 |
| | av. $\delta$ (mm s$^{-1}$) | 0.47 | 0.47 | 0.48 |

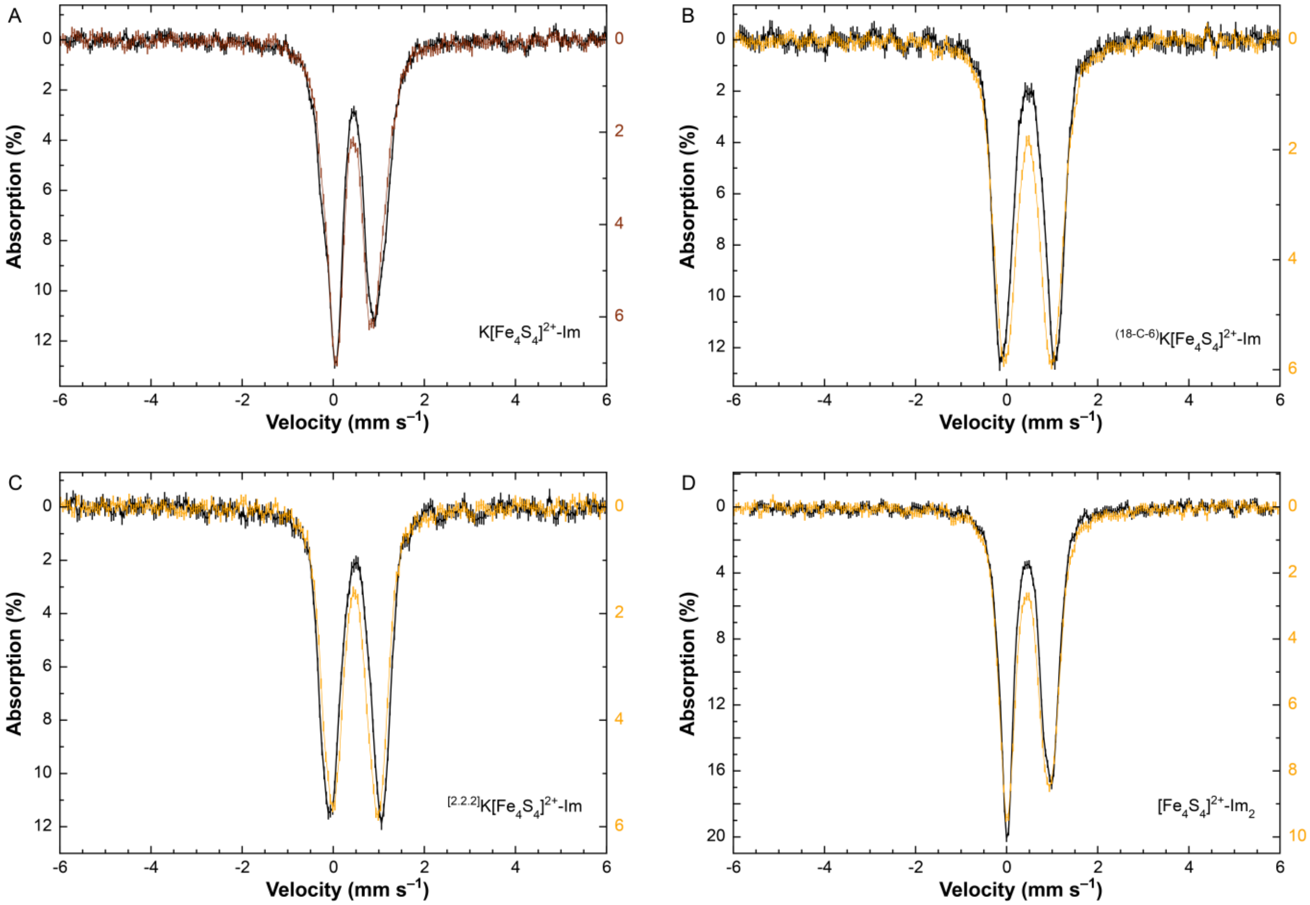


**Figure S30.** Mössbauer powder spectra (*vertical bars*) of K[$Fe_4S_4$(DmpS)$_3$(Im*)] (A), [18-C-6]K[$Fe_4S_4$(DmpS)$_3$(Im*)] (B), [2.2.2]K[$Fe_4S_4$(DmpS)$_3$(Im*)] (C) and [$Fe_4S_4$(DmpS)$_2$(Im*)$_2$] (D) recorded at 80 K and zero-field (*colored*) and at 5.7 K and 0.06 T magnetic field applied parallel to the γ-rays (*black*).

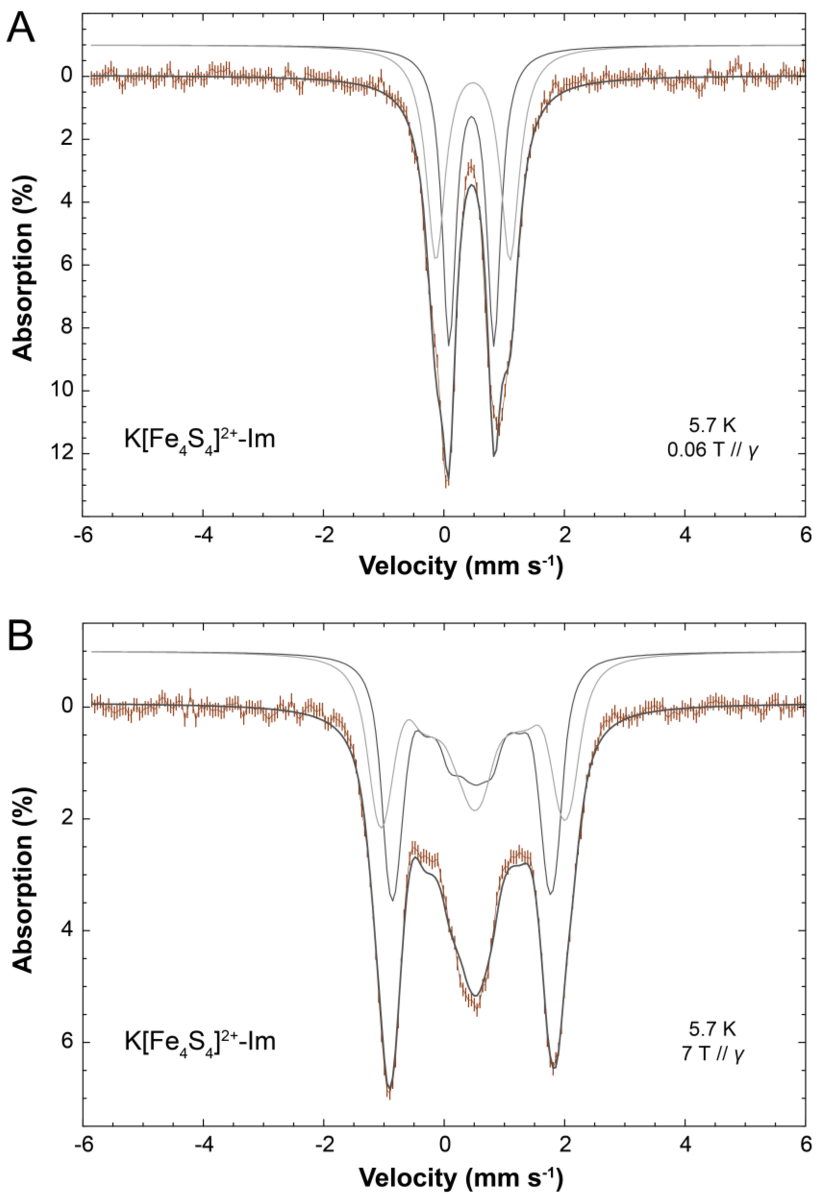


**Figure S31.** 6 K variable-field $^{57}Fe$ Mössbauer spectra of $K[Fe_4S_4(DmpS)_3(Im^*)]$ (*vertical bars*) recorded on a powder sample at 0.06 (A) and 7 T (B) external magnetic fields applied parallel to the *γ*-rays. In both panels, simulations are overlaid as *bold dark grey lines*. Two diamagnetic sites in a 1:1 ratio were considered, each bearing a unique linewidth. The individual sites' contributions are displayed above as *thin light grey* and *dark grey traces*, respectively. Simulation parameters are condensed in Table S14.

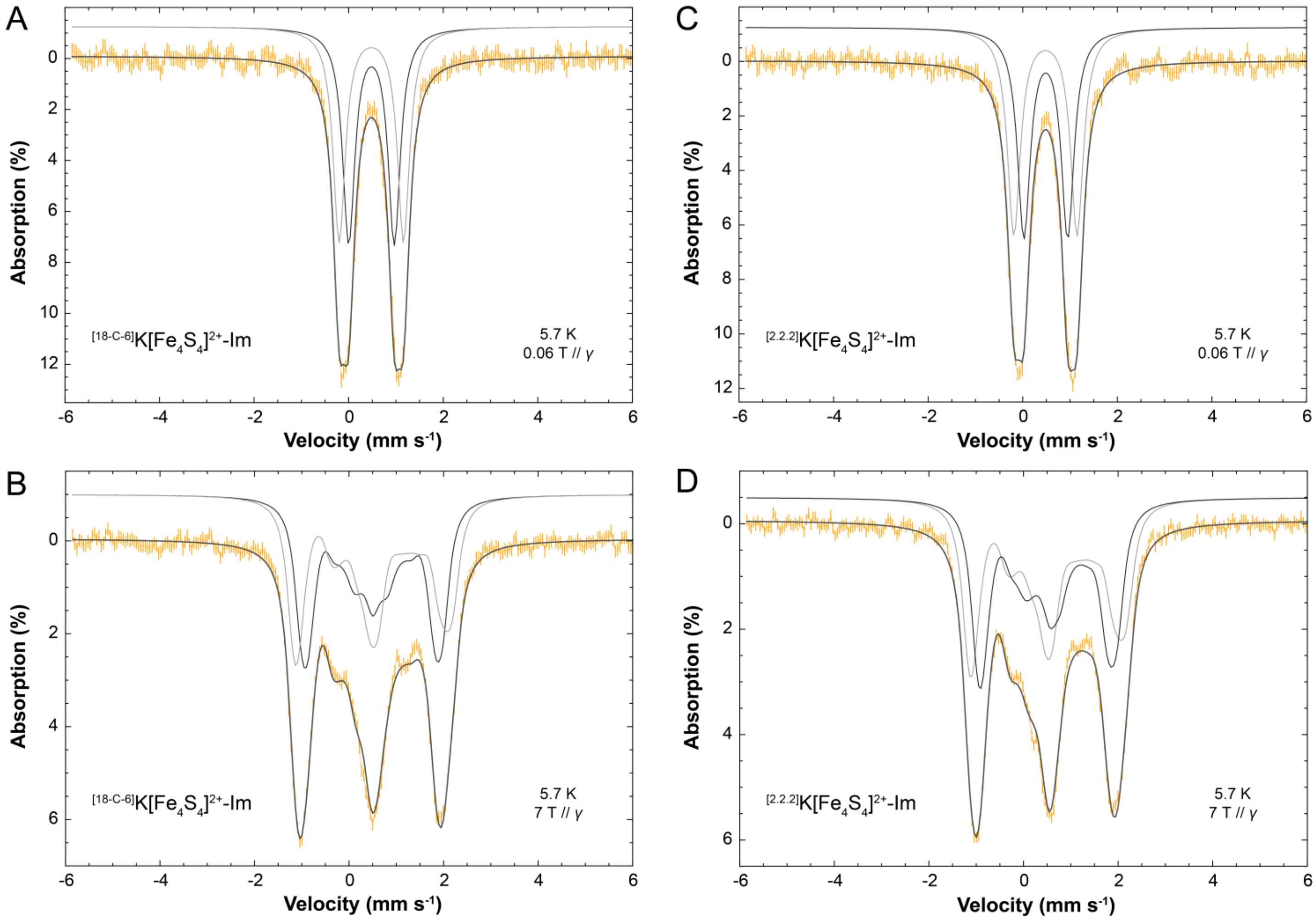


**Figure S32.** 6 K variable-field $^{57}$Fe Mössbauer spectra of $^{[18\text{-}C\text{-}6]}$K[$Fe_4S_4$(DmpS)$_3$(Im*)] (A,B) and $^{[2.2.2]}$K[$Fe_4S_4$(DmpS)$_3$(Im*)] (C,D), respectively, (*vertical bars*) recorded on powder samples at 0.06 (A,C) and 7 T (B,D) external magnetic fields applied parallel to the γ-rays. In all panels, simulations are overlaid as *bold dark grey lines*. For both compounds, two diamagnetic sites in a 1:1 ratio with the same linewidth were considered to reproduce the spectra. The individual sites' contributions are displayed above as *thin light grey* and *dark grey traces*, respectively. Simulation parameters are condensed in Table S14.

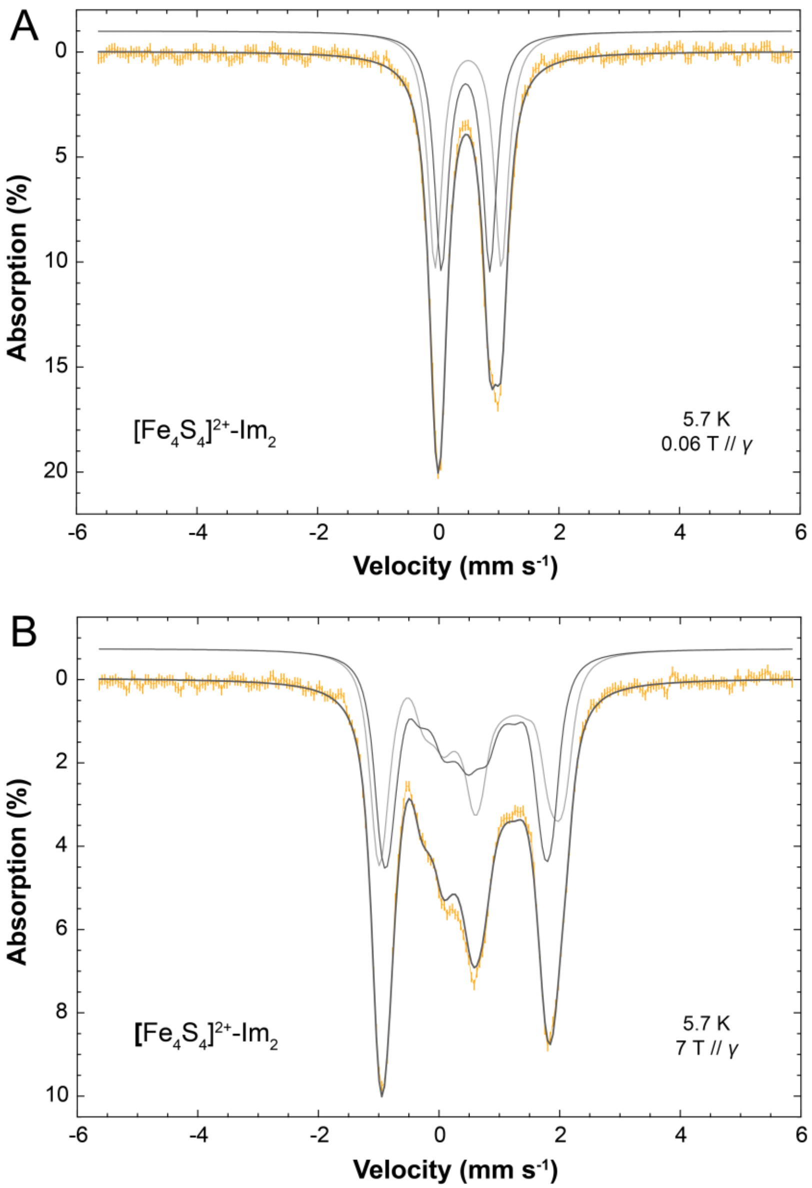


**Figure S33.** 6 K variable-field $^{57}Fe$ Mössbauer spectra of $[Fe_4S_4(DmpS)_2(Im^*)_2]$ (*vertical bars*) recorded on a powder sample at 0.06 (A) and 7 T (B) external magnetic fields applied parallel to the γ-rays. In both panels, simulations are overlaid as *bold dark grey lines*. Two diamagnetic sites in a 1:1 ratio with the same linewidth were considered to reproduce the spectra. The individual sites' contributions are displayed above as *thin light grey* and *dark grey traces*, respectively. Simulation parameters are condensed in Table S14.

**Table S14.** Parameters of the two equally contributing diamagnetic Fe sites considered to reproduce the experimental variable-field 6 K spectra of the powder samples of the X[$Fe_4S_4$(DmpS)$_3$(Im*)] clusters ($X^+$=$K^+$,[18-C-6]$K^+$,[2.2.2]$K^+$), which are shown in Figures S31-S32 and that of [$Fe_4S_4$(DmpS)$_2$(Im*)$_2$] (Figure S33). A common linewidth was assumed in all except that of K[$Fe_4S_4$(DmpS)$_3$(Im*)].

| | parameter | **K-[$Fe_4S_4$]$^{2+}$-Im** | **[18-C-6]K-[$Fe_4S_4$]$^{2+}$-Im** | **[2.2.2]K-[$Fe_4S_4$]$^{2+}$-Im** | **[$Fe_4S_4$]$^{2+}$-Im$_2$** |
|---|---|---|---|---|---|
| Site 1 (*light grey*) | $\delta$ (mm s$^{-1}$) | 0.48 | 0.48 | 0.48 | 0.50 |
| | $\Delta E_Q$ (mms$^{-1}$) | 1.23 | 1.36 | 1.34 | 1.10 |
| | $\eta$ | 0.9 | 0.5 | 0.5 | 0.5 |
| | $\Gamma_{fwhm}$ (mm s$^{-1}$) | 0.39 | 0.31 | 0.33 | 0.29 |
| Site 2 (*dark grey*) | $\delta$ (mm s$^{-1}$) | 0.46 | 0.48 | 0.49 | 0.45 |
| | $\Delta E_Q$ (mms$^{-1}$) | 0.75 | 0.96 | 0.93 | 0.80 |
| | $\eta$ | 0.9 | 0.9 | 0.6 | 0.9 |
| | $\Gamma_{fwhm}$ (mm s$^{-1}$) | 0.28 | 0.31 | 0.33 | 0.29 |
| | av. $\delta$ (mm s$^{-1}$) | 0.47 | 0.48 | 0.48 | 0.48 |

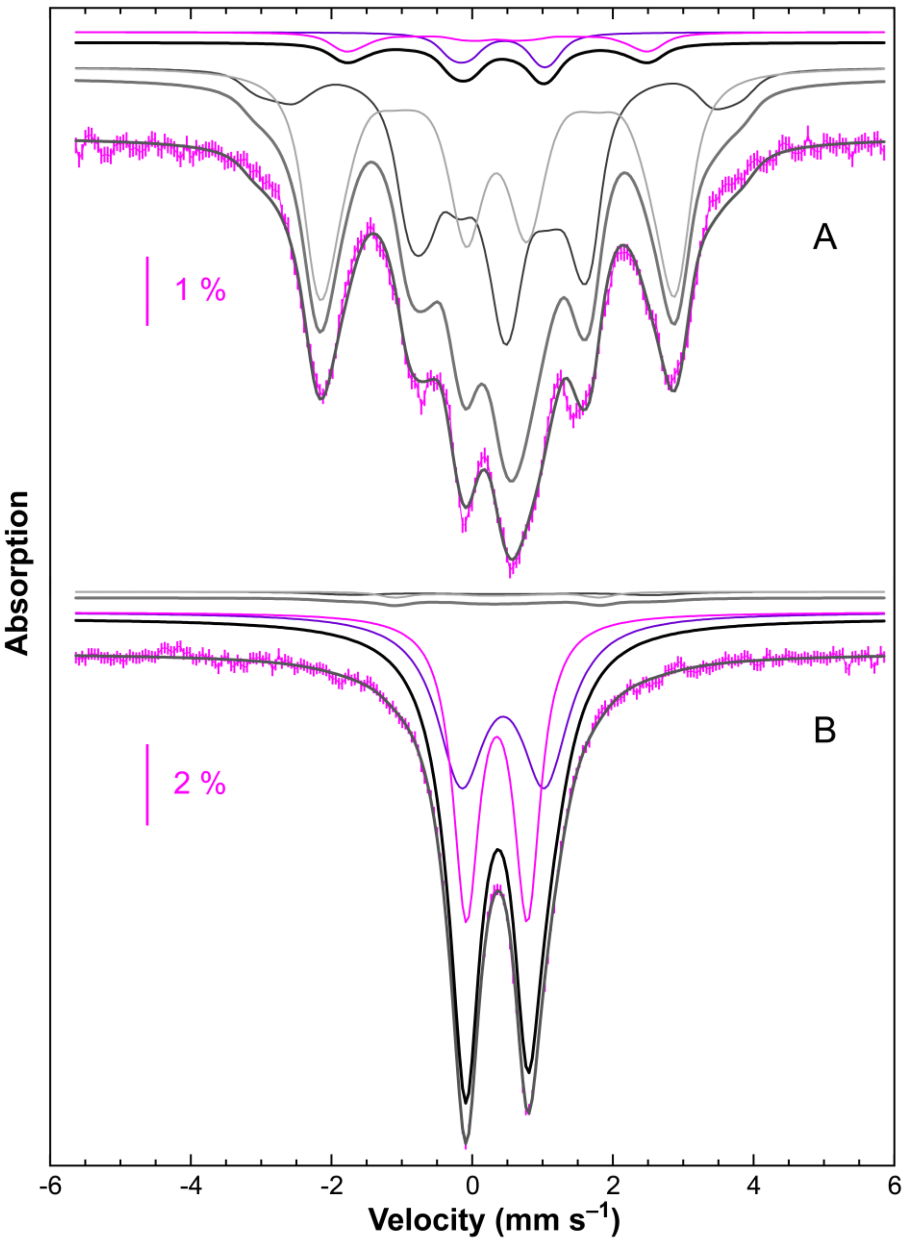


**Figure S34.** Experimental (pink vertical bars) 6 K Mössbauer powder spectrum of [$Fe_4S_4$(DmpS)$_3$(Im*)] recorded using a 7 T (A) and 0.06 T (B) magnetic field applied along the *γ*-rays direction. The simulations are overlaid as a *grey solid thick line* and parameters are listed in Table S15. A *S*=1/2 spin was considered. Two 1:1 slow relaxing and two 1:1 fast relaxing sites were considered. The same parameter set was considered for the first (resp. second) site of each system. The slow relaxing contributions are shown in *grey* (*medium grey thick line* for the sum, *dark* and *light grey* thin lines for each site) above the spectra whereas the fast ones are reproduced in color (*black thick line* for the sum, *magenta* and *purple thin lines* for each site). The parameters are listed in Table S15.

**Table S15.** List of parameters determined upon simulating the 6 K powder spectra of [$Fe_4S_4$(DmpS)$_3$(Im*)] when considering a $S$=1/2 ground state.

| parameter | Site 1 | | Site 2 | |
|---|---|---|---|---|
| $\delta$ (mm s$^{-1}$) | 0.35 | | 0.44 | |
| $\Delta E_Q$ (mm s$^{-1}$) | –0.87 | | –1.19 | |
| $\eta$ | 0.9 | | 1.0 | |
| $A_k$ / ($g_n$ $\mu_n$) ($k$ = $x$, $y$, $z$) (T) | 16 | | –21 | |
| | 15 | | –23 | |
| | 14 | | –25 | |
| $\Gamma_{fwhm}$ (mm s$^{-1}$) | 0.45/0.43[a] | | 0.83/0.39[a] | |
| relaxation | slow | fast | slow | fast |
| color | *light grey* | *magenta* | *dark grey* | *purple* |
| contribution (%) | 2/48[a] | 50/3[a] | 2/48[a] | 50/3[a] |

[a] The two values are for the spectra recorded at 0.06 and 7 T external magnetic field, respectively.

## $^{1}H$,$^{13}C$ NMR and VT $^{1}H$ NMR spectra

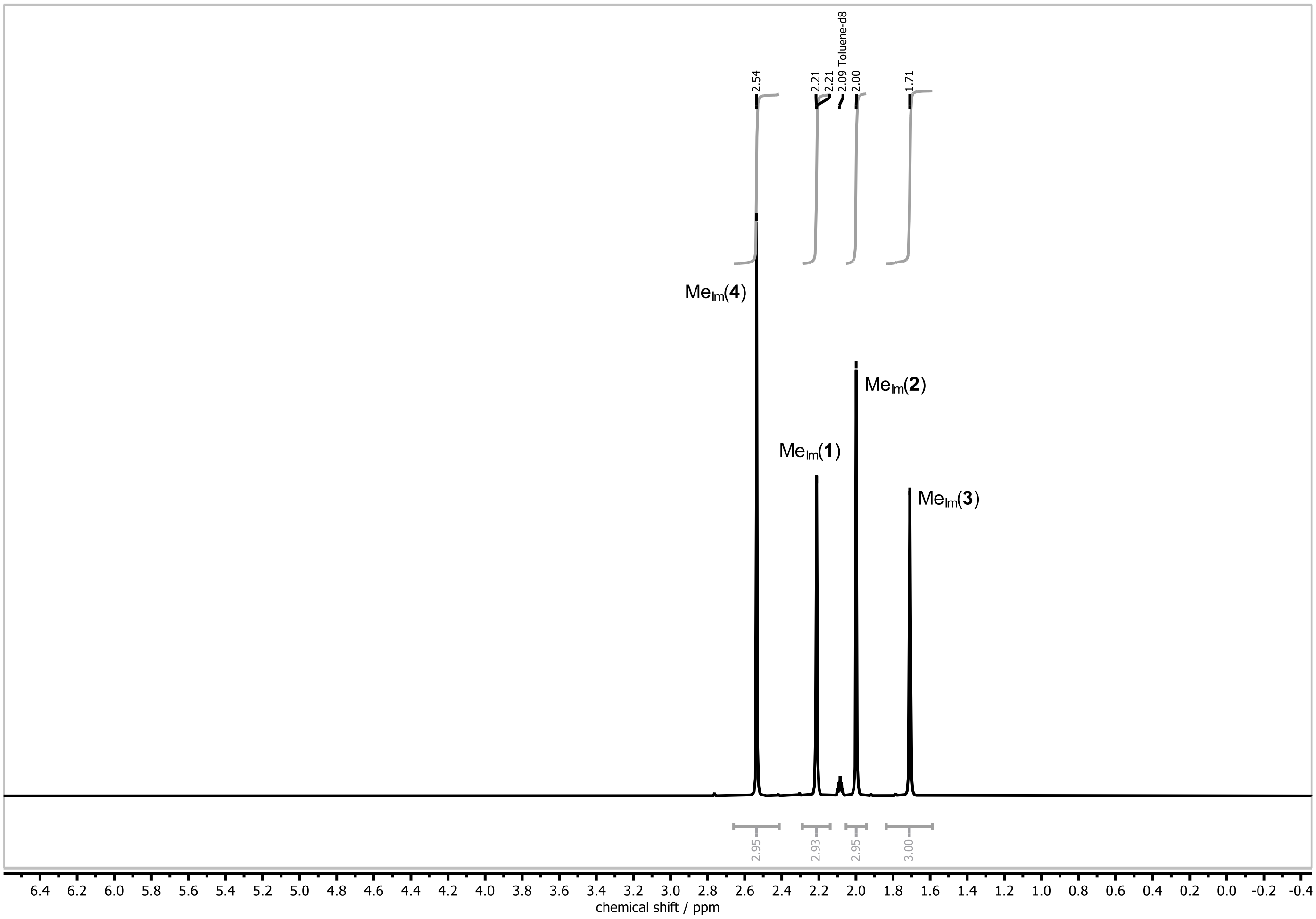


**Figure S35.** 300 MHz $^{1}H$ NMR spectrum of Im* recorded at room temperature in toluene-*d*8.

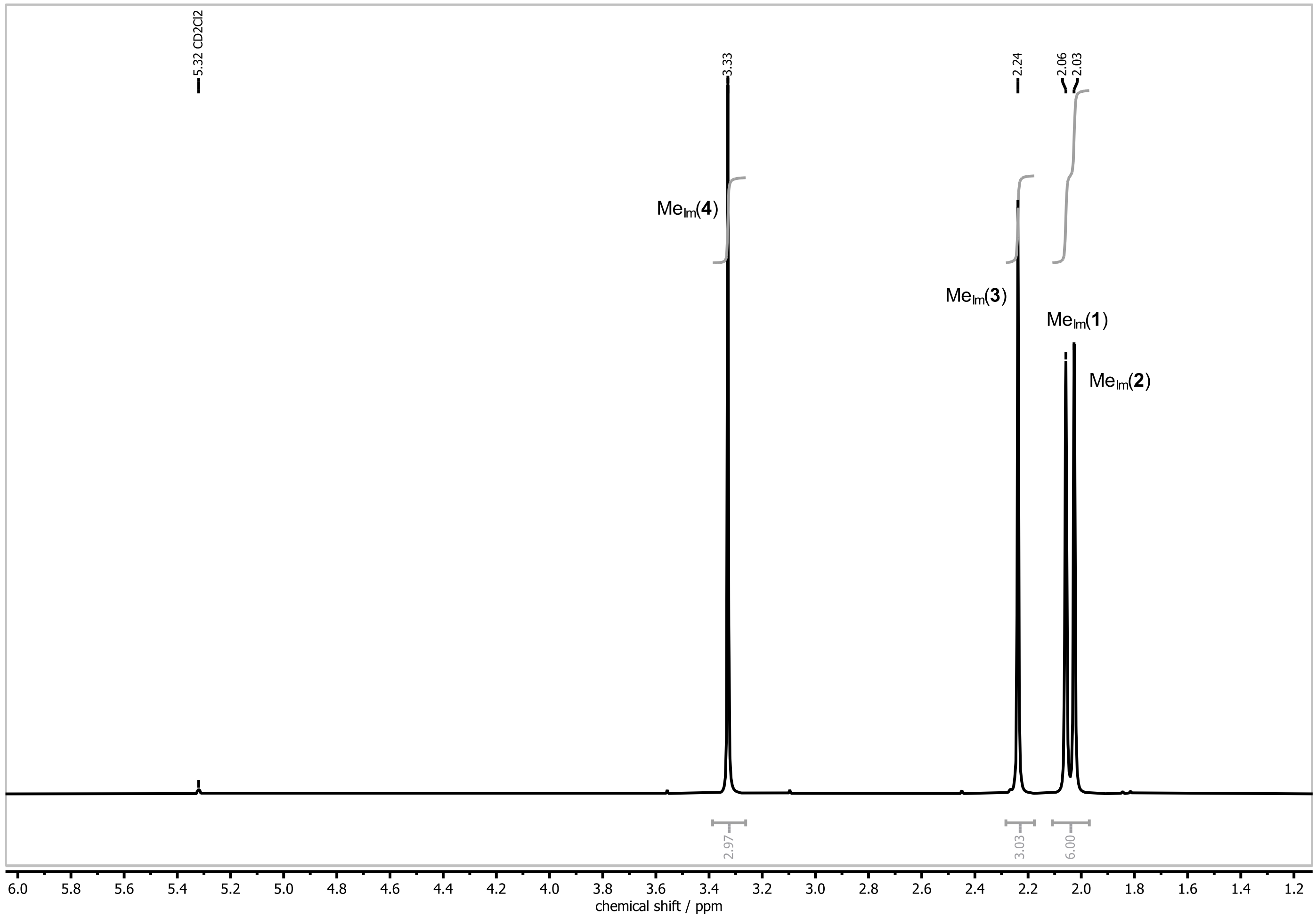


**Figure S36.** 300 MHz $^{1}$H NMR spectrum of Im* recorded at room temperature in $CD_2Cl_2$.

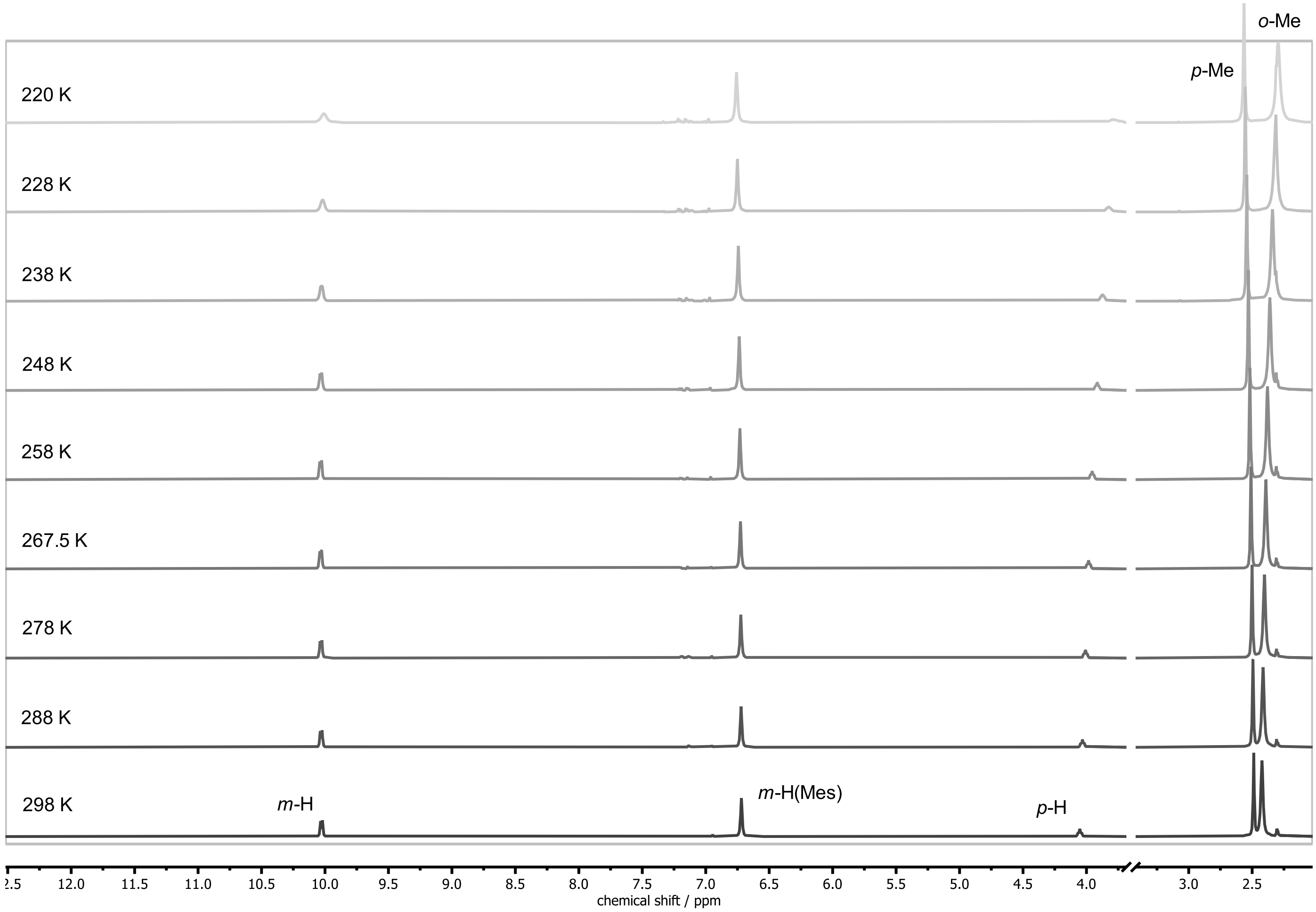


**Figure S37.** 500 MHz VT $^1$H NMR spectra of $K[Fe_4S_4(DmpS)_4]$ recorded at room temperature in THF-*d*8. For clarity, the regions of the solvent residual peaks were omitted.

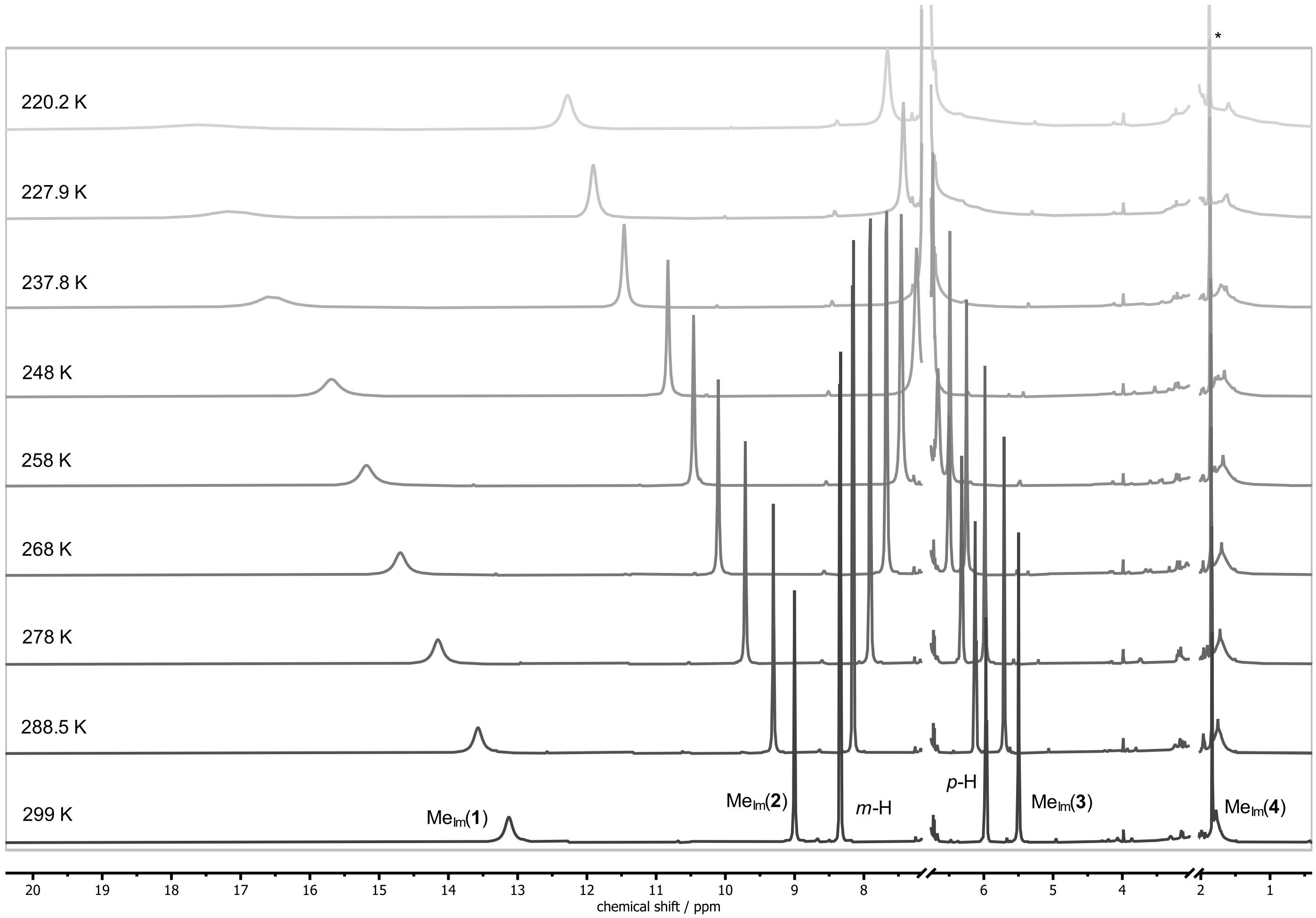


**Figure S38.** 500 MHz VT $^1$H NMR spectrum of [$Fe_4S_4$(DmpS)$_3$(Im*)] recorded in toluene-*d*8. An unknown impurity peak is marked by an asterisk (*). For clarity, the regions of the solvent residual peaks, *p*-Me, *o*-Me and *m*-H(Mes) were omitted.

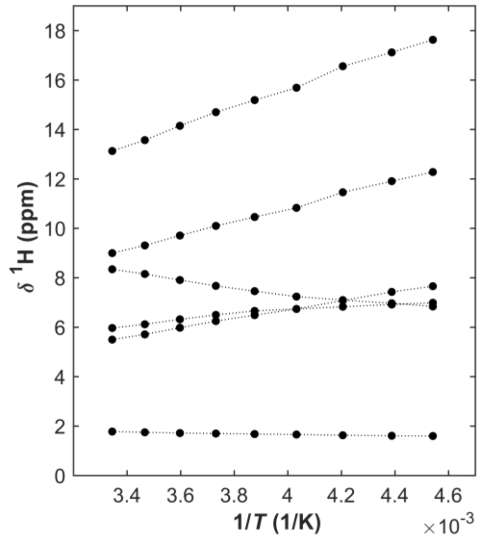


**Figure S39.** Plot of the chemical shift values in [$Fe_4S_4$(DmpS)$_3$(Im*)] versus 1/*T*.

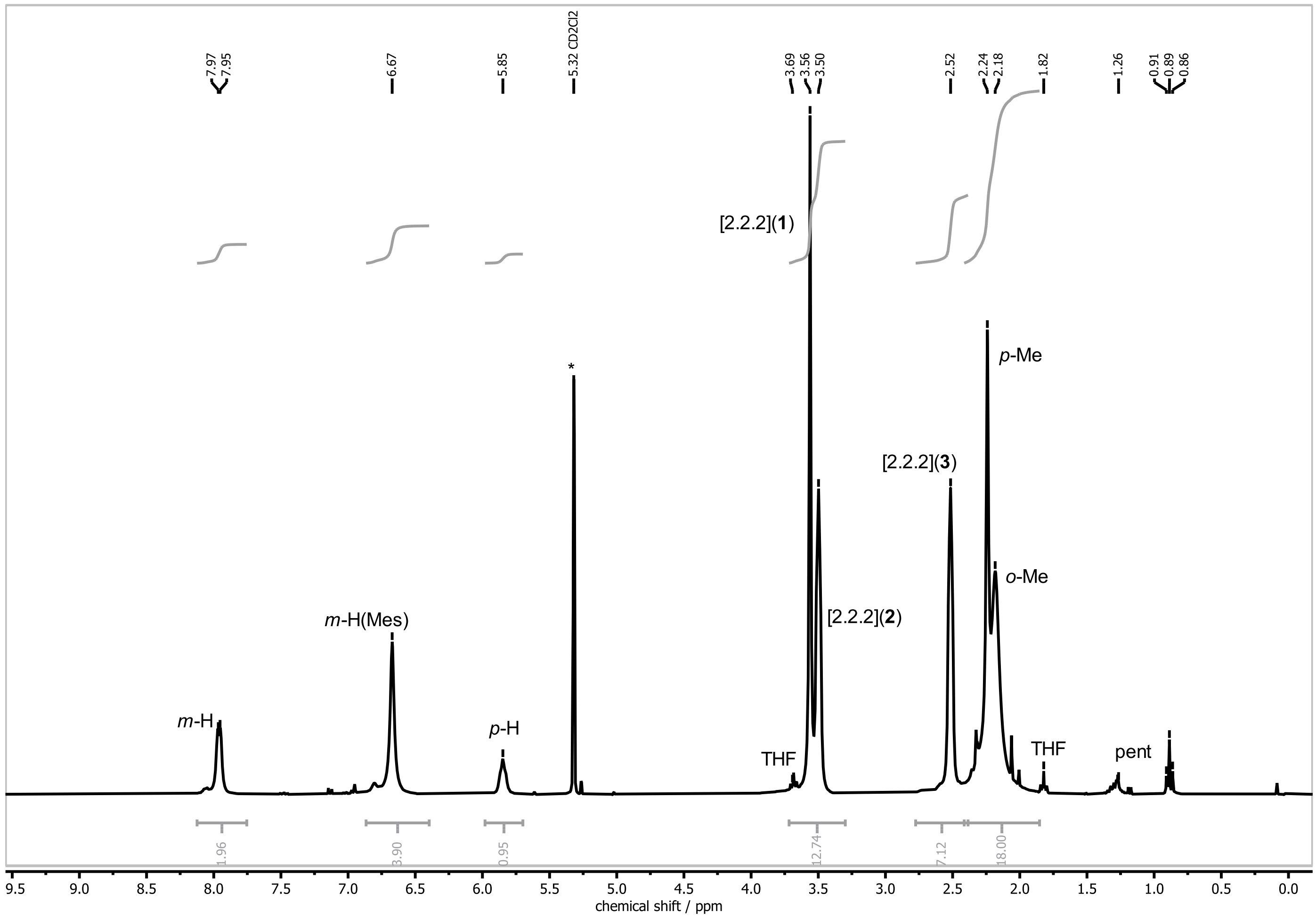


**Figure S40.** 300 MHz $^{1}H$ NMR spectrum of $^{[2.2.2]}K_2[Fe_4S_4(DmpS)_4]$ recorded at room temperature in $CD_2Cl_2$ (*).

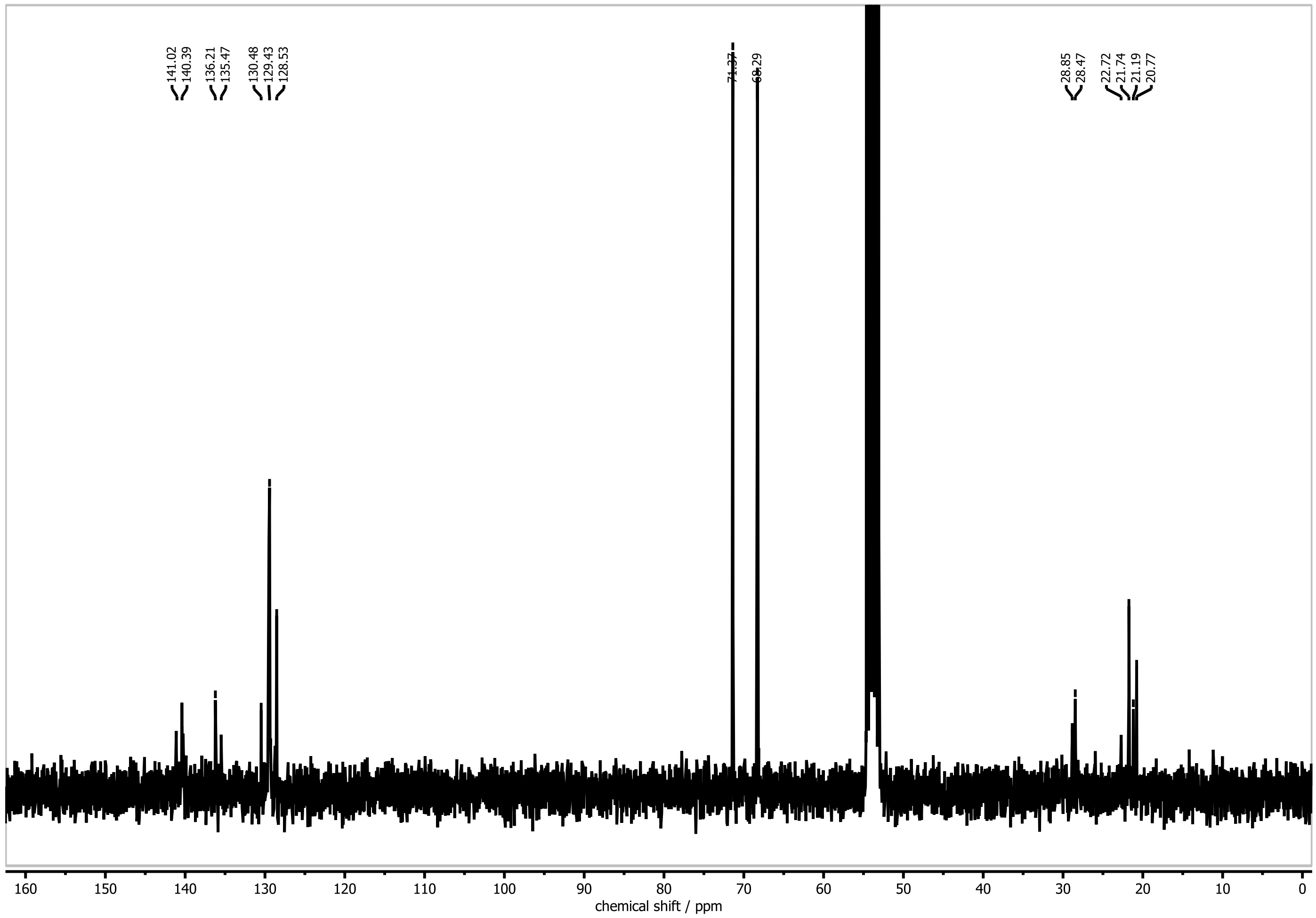


**Figure S41.** 75 MHz $^{13}C$ NMR spectrum of $^{[2.2.2]}K_2[Fe_4S_4(DmpS)_4]$ recorded at room temperature in $CD_2Cl_2$.

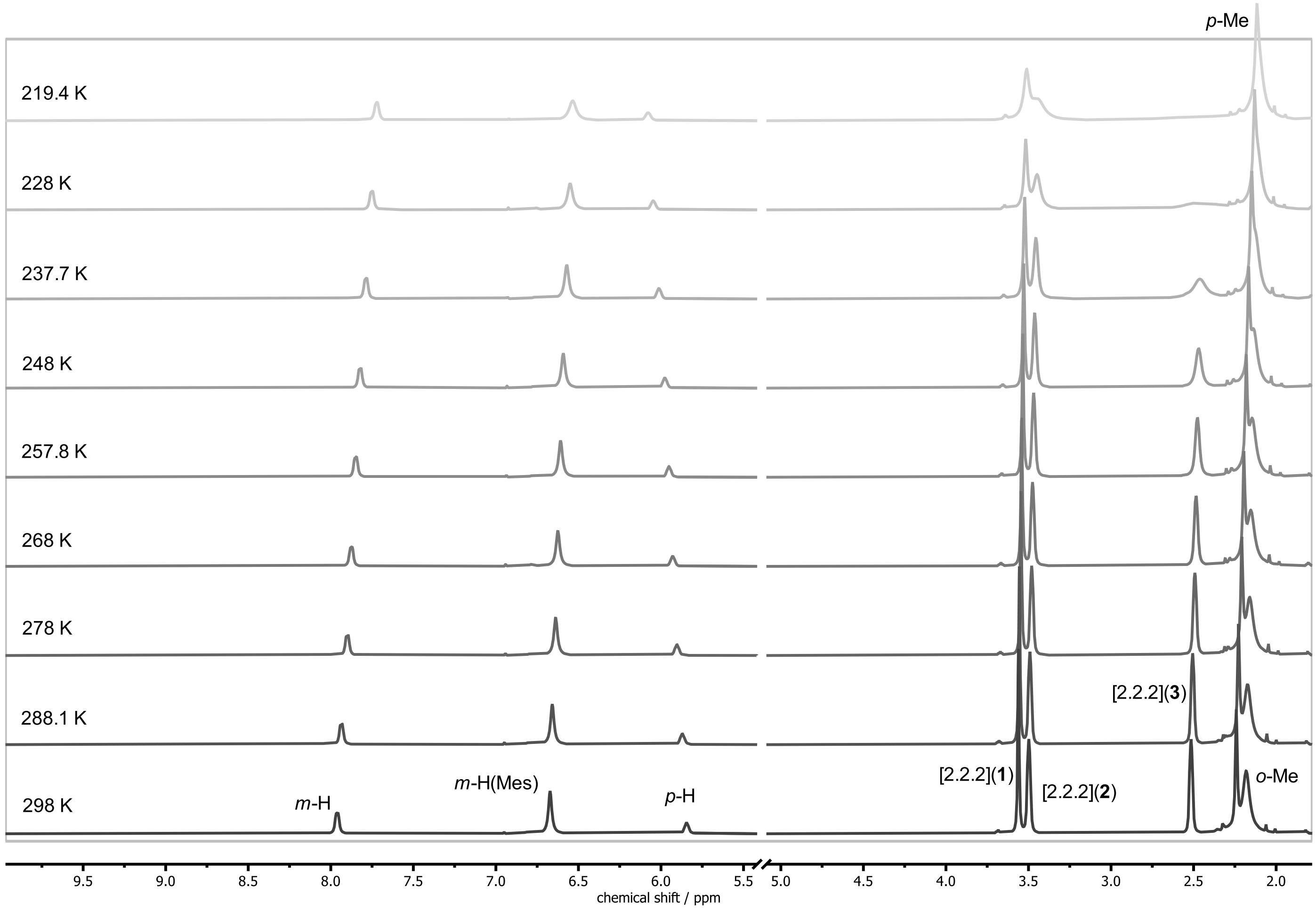


**Figure S42.** 500 MHz VT $^{1}H$ NMR spectrum of $^{[2.2.2]}K_2[Fe_4S_4(DmpS)_4]$ recorded at room temperature in $CD_2Cl_2$. For clarity, the region of the solvent residual peak was omitted.

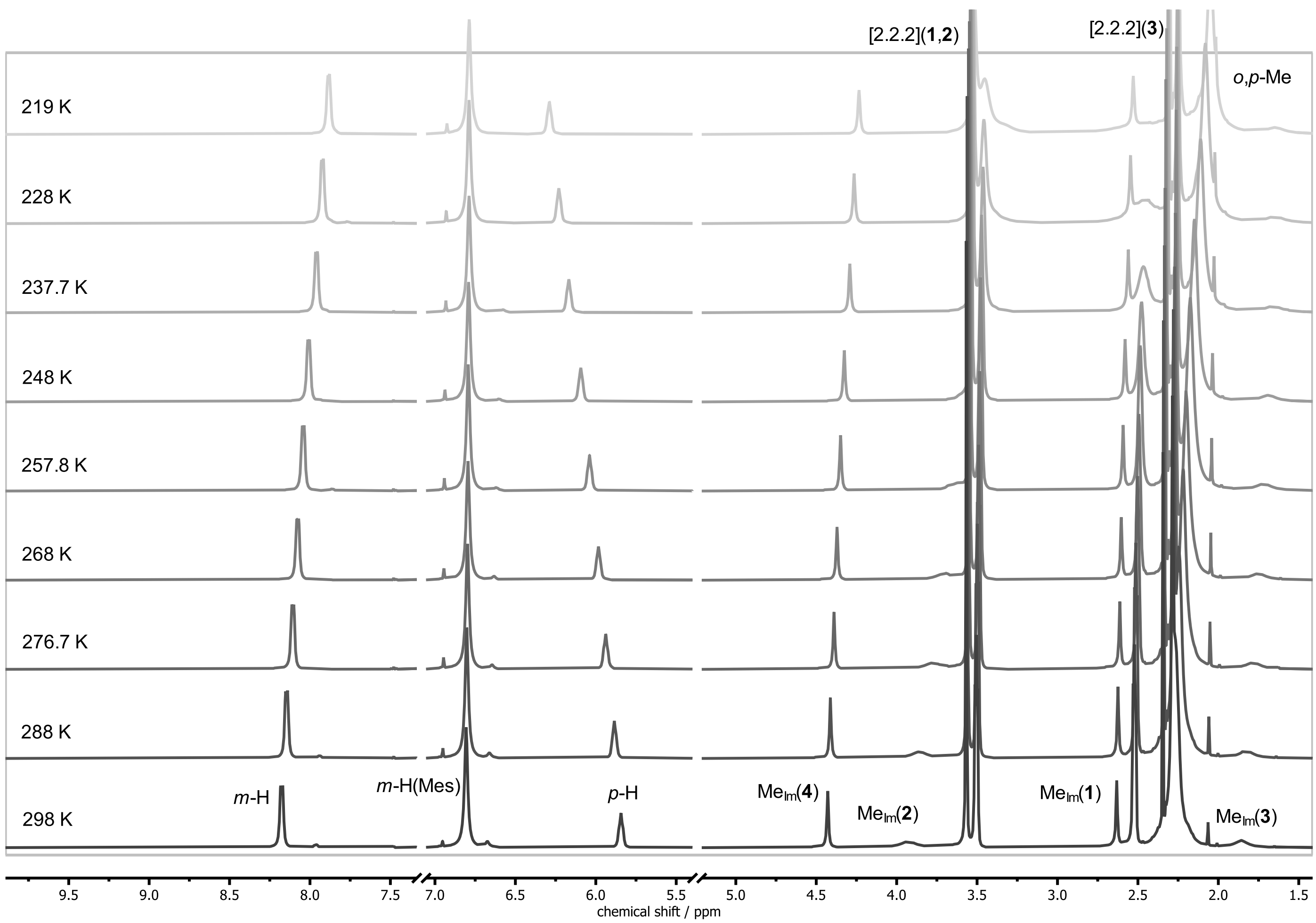


**Figure S43.** 500 MHz VT $^{1}H$ NMR spectrum of $^{[2.2.2]}K[Fe_4S_4(DmpS)_3(Im^*)]$ recorded at room temperature in $CD_2Cl_2$. For clarity, the region of the solvent residual peak was omitted.

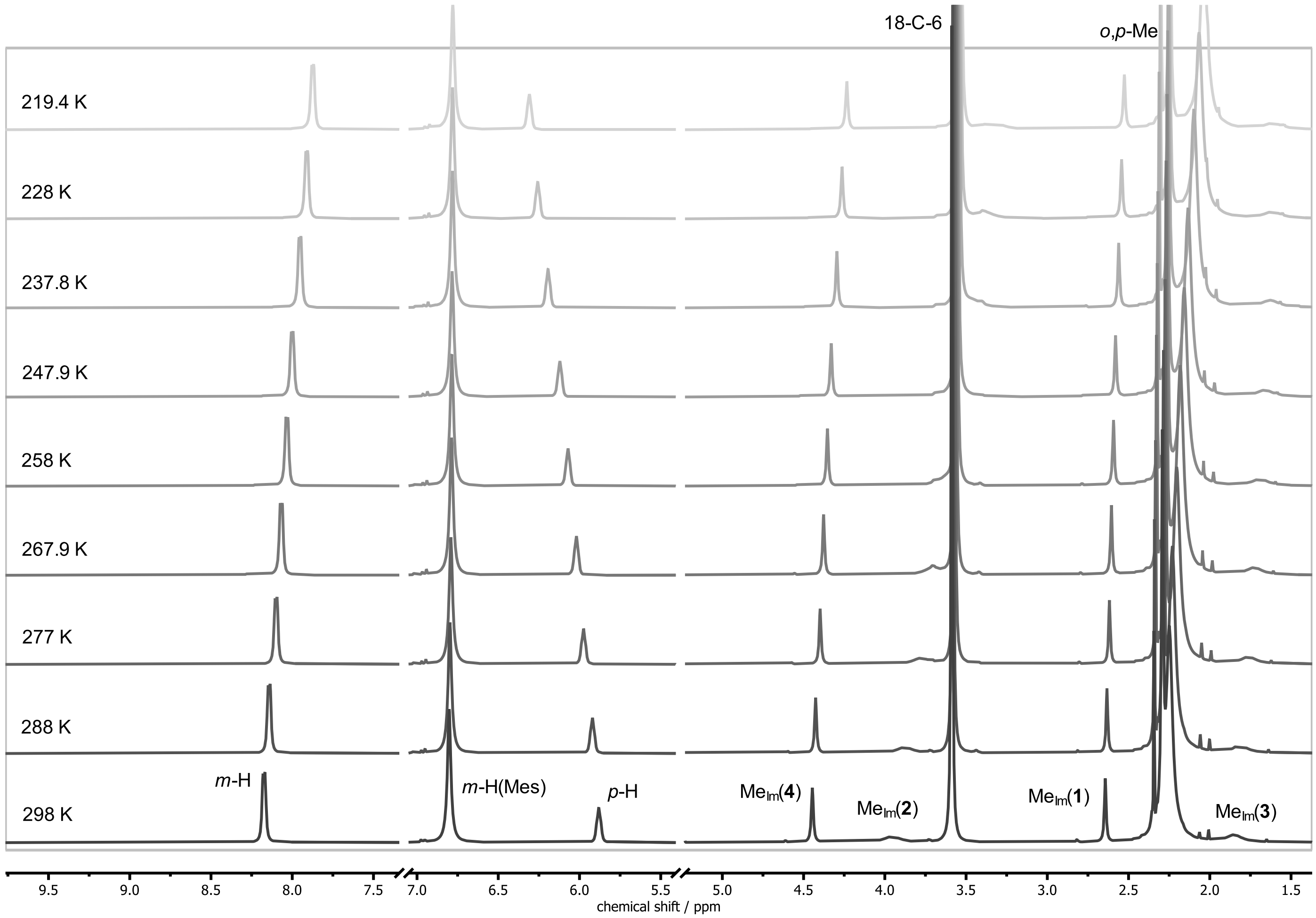


**Figure S44.** 500 MHz VT $^1H$ NMR spectrum of $^{(18\text{-}C\text{-}6)}K[Fe_4S_4(DmpS)_3(Im^*)]$ recorded at room temperature in $CD_2Cl_2$. For clarity, the region of the solvent residual peak was omitted.

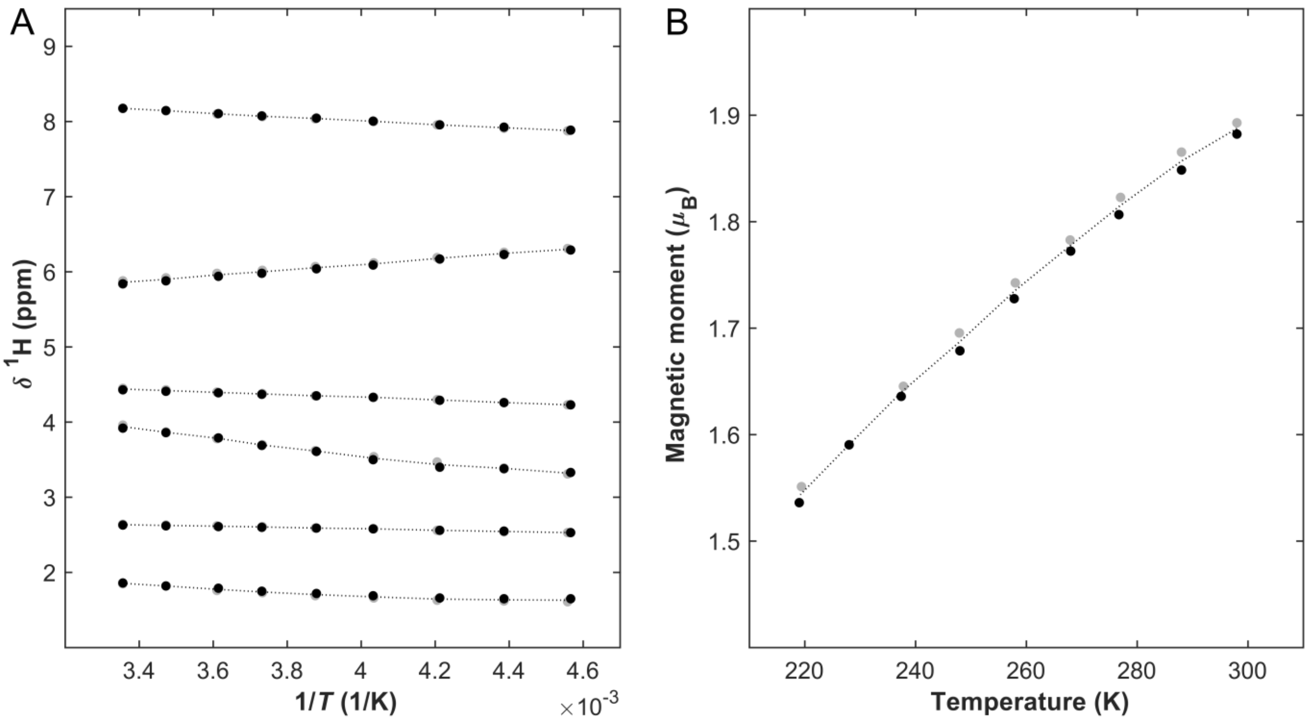


**Figure S45.** (A) Plot of the chemical shift values in $^{[2.2.2]}$K[$Fe_4S_4$(DmpS)$_3$(Im*)] (*black*) and $^{(18\text{-}C\text{-}6)}$K[$Fe_4S_4$(DmpS)$_3$(Im*)] (*grey*) versus 1/*T*. The dotted line follows the average value of the two datasets, which are basically identical. (B) Evan's solution-state magnetic moments of $^{[2.2.2]}$K[$Fe_4S_4$(DmpS)$_3$(Im*)] (*black*) and $^{(18\text{-}C\text{-}6)}$K[$Fe_4S_4$(DmpS)$_3$(Im*)] (*grey*) versus *T.* The dotted line follows the average value of the two datasets.

**Note S3.** As it is evident from the $^1$H NMR spectra of the two compounds as well as the Curie-plots of the chemical shifts and the solution-state magnetic moment, the congeners of [$Fe_4S_4$(DmpS)$_3$(Im*)]$^-$ bearing [2.2.2]($K^+$) or (18-C-6)($K^+$) cations exhibit nearly identical behavior. Thus, because [2.2.2]-cryptand is assumed to separate cation/anion pair better than 18-crown-6, we use the data recorded on $^{[2.2.2]}$K[$Fe_4S_4$(DmpS)$_3$(Im*)] for fitting of the VT $^1$H NMR data. However, either an average of the two datasets or the other dataset can also be used, producing qualitatively identical results.

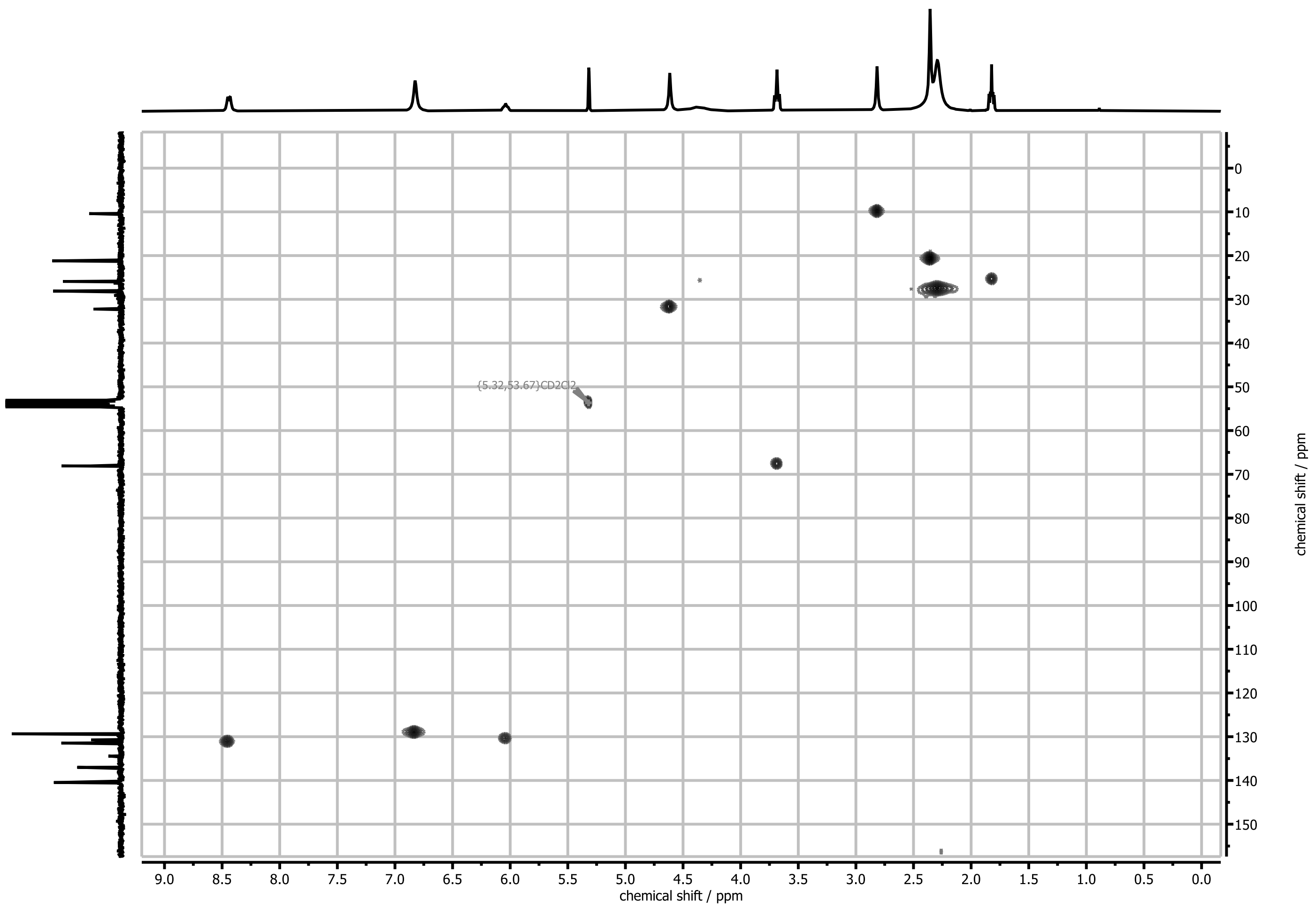


**Figure S46.** 300 MHz $^{1}H$-$^{13}C$ HSQC NMR spectrum of $[Fe_4S_4(DmpS)_2(Im^*)_2]$ recorded at room temperature in $CD_2Cl_2$ (*).

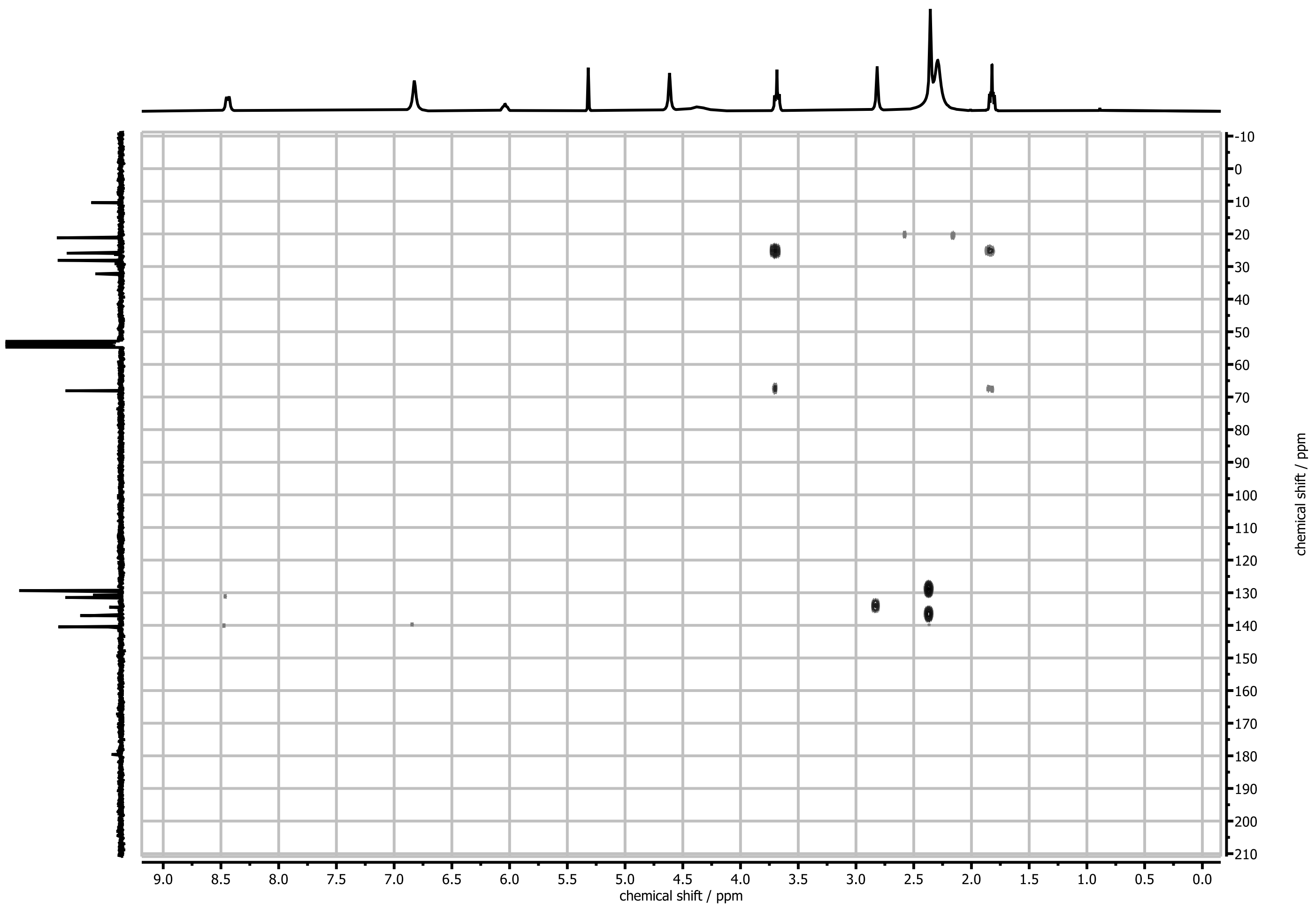


**Figure S47.** 300 MHz $^{1}H$-$^{13}C$ HMBC NMR spectrum of $[Fe_4S_4(DmpS)_2(Im^*)_2]$ recorded at room temperature in $CD_2Cl_2$ (*).

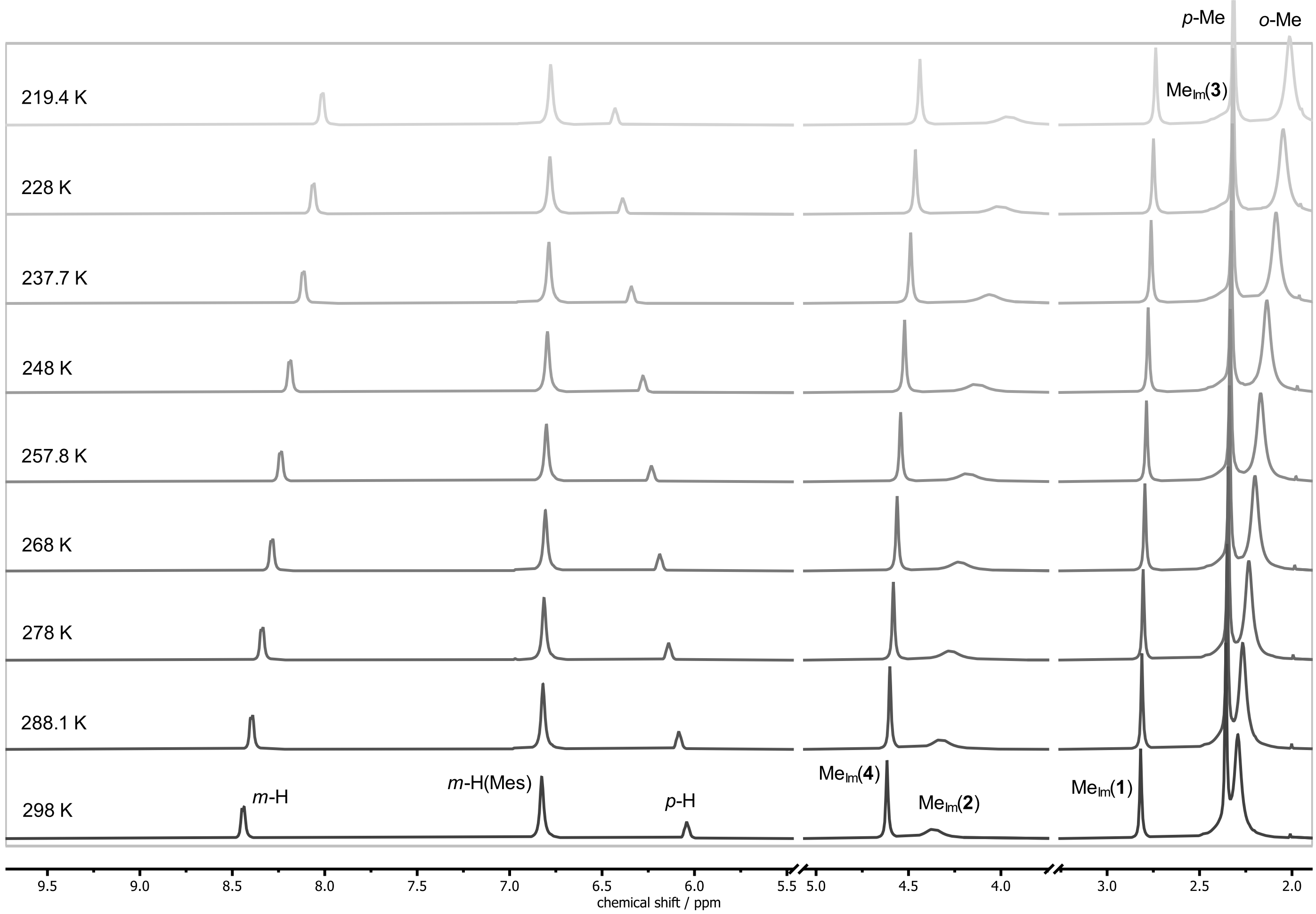


**Figure S48.** 500 MHz VT $^1$H NMR spectrum of [$Fe_4S_4$(DmpS)$_2$(Im*)$_2$] recorded at room temperature in $CD_2Cl_2$ (*). For clarity, the regions containing the solvent residual peak and the peaks of residual THF from the crystal lattice were omitted.

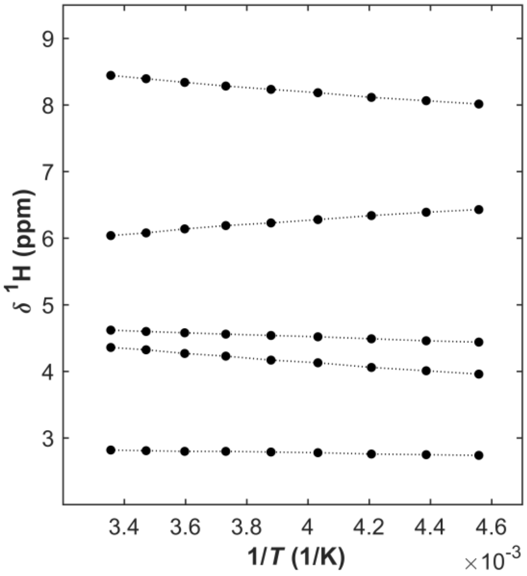


**Figure S49.** Plot of the chemical shift values in [$Fe_4S_4$(DmpS)$_2$(Im*)$_2$] versus 1/*T*.

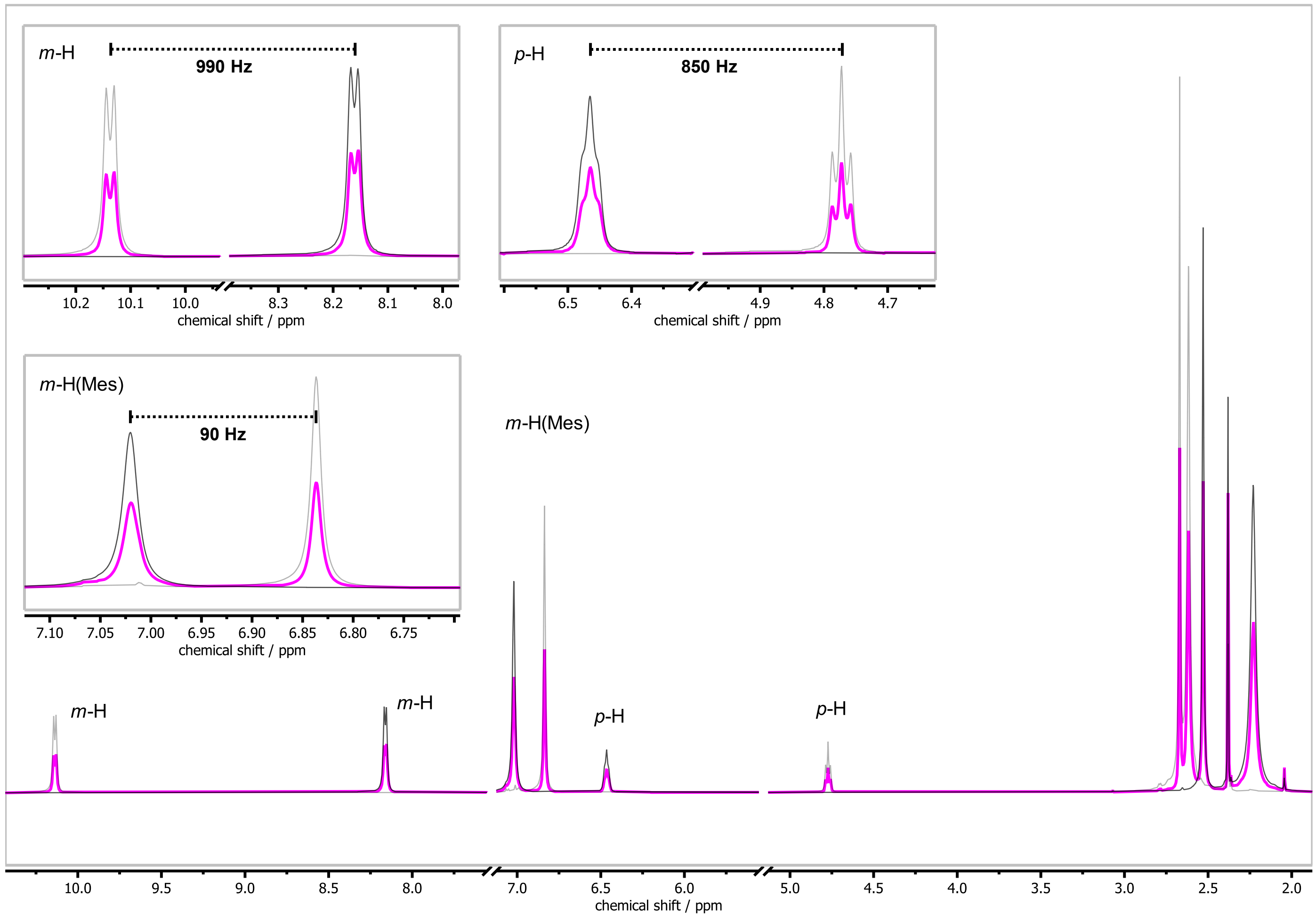


**Figure S50.** 300 K $^{1}$H NMR spectra of $K_2[Fe_4S_4(DmpS)_4]$ (*dark grey*), $K[Fe_4S_4(DmpS)_4]$ (*light grey*) and a redox mixture of the two (*magenta*) recorded at 500 MHz in $CD_2Cl_2$. Insets show zoom-ins on selected peaks, which are annotated with their respective frequency differences, *Δv*.

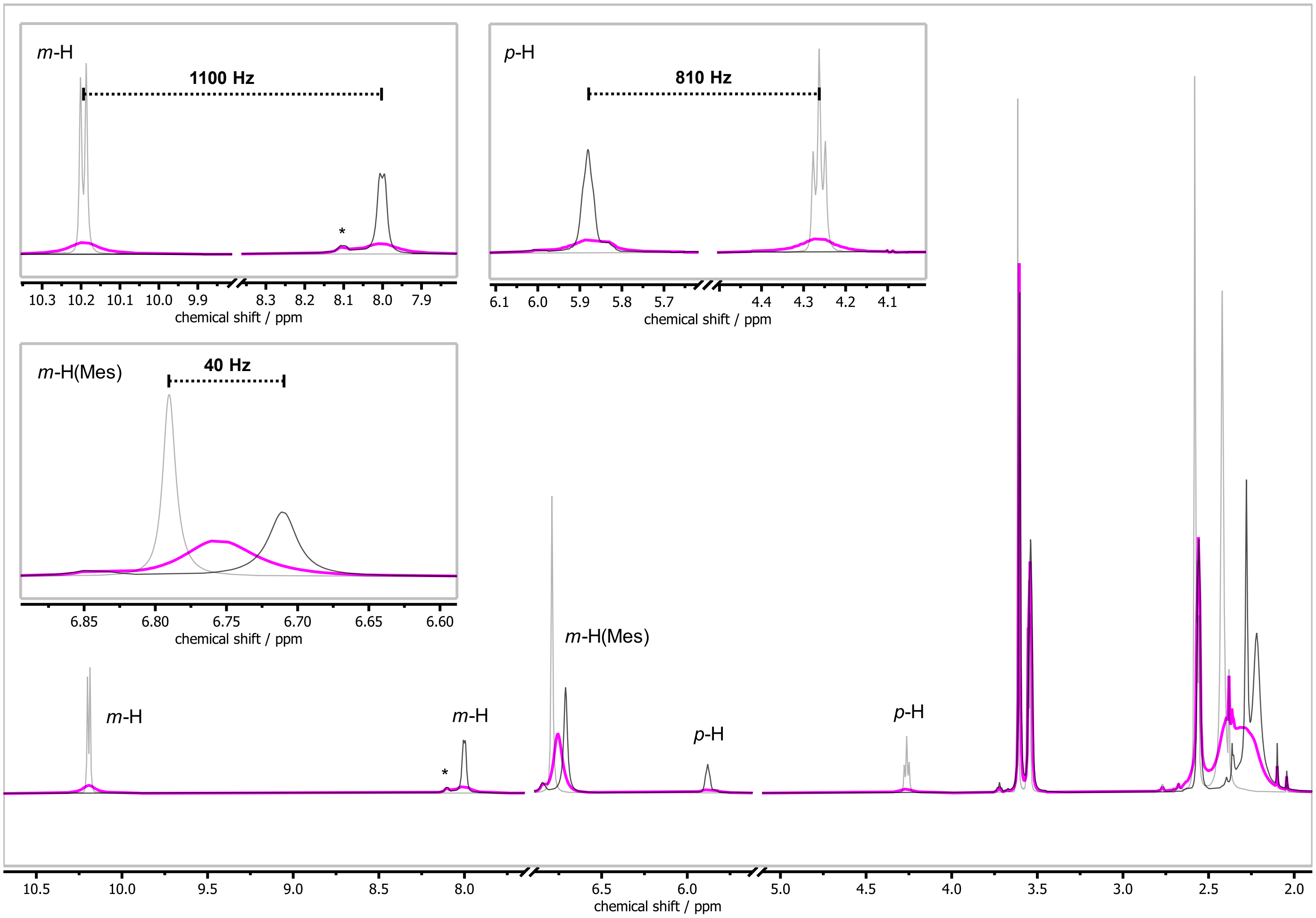


**Figure S51.** 300 K $^{1}$H NMR spectra of $^{[2.2.2]}K_2[Fe_4S_4(DmpS)_4]$ (*dark grey*), $^{[2.2.2]}K[Fe_4S_4(DmpS)_4]$ (*light grey*) and a redox mixture of the two (*magenta*) recorded at 500 MHz in $CD_2Cl_2$. Insets show zoom-ins on selected peaks, which are annotated with their respective frequency differences, $\Delta v$.

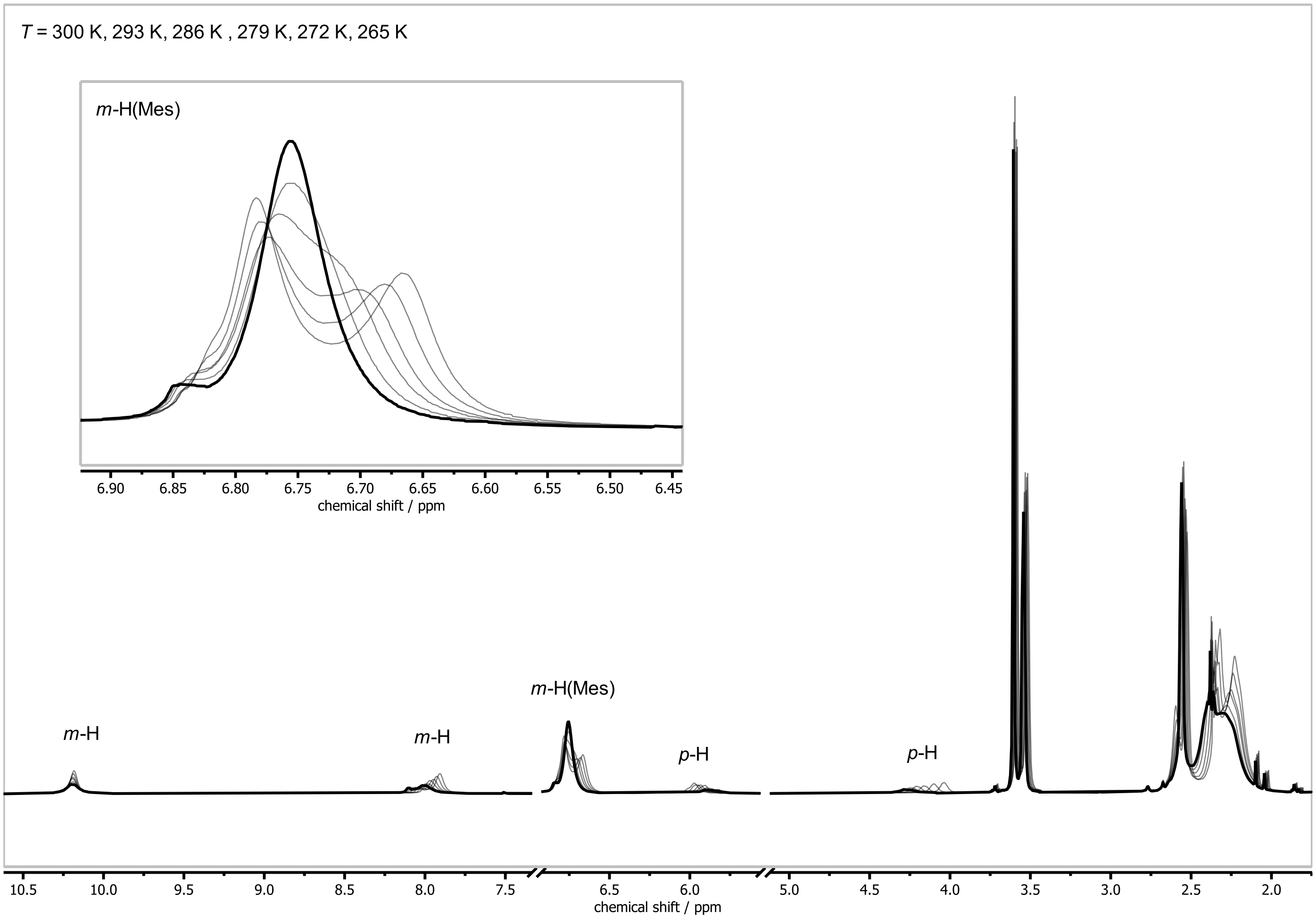


**Figure S52.** Variable-temperature $^1$H NMR spectra of the redox mixture of $^{[2.2.2]}K_2[Fe_4S_4(DmpS)_4]$, $^{[2.2.2]}K[Fe_4S_4(DmpS)_4]$ recorded at 500 MHz in $CD_2Cl_2$. The 300 K spectrum is shown as a *black line*, whereas the lower-T spectra are shown as *grey* ones. The inset highlights the coalescence of the *m*-H(Mes) peaks around 290-300 K.

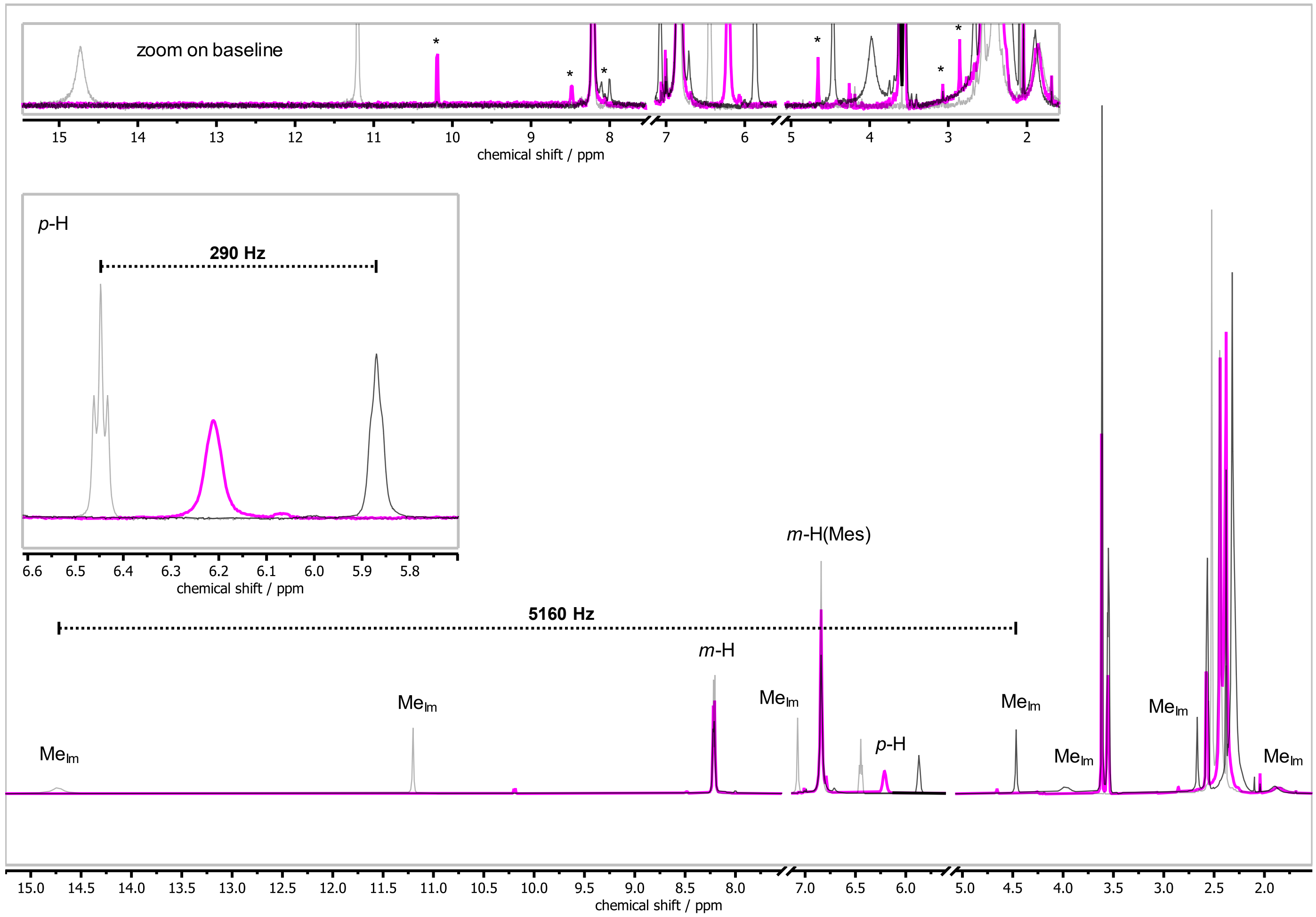


**Figure S53.** 300 K $^1$H NMR spectra of $^{[2.2.2]}$K[$Fe_4S_4$(DmpS)$_3$(Im*)] (*dark grey*), [$Fe_4S_4$(DmpS)$_3$(Im*)] (*light grey*) and a redox mixture of the two (*magenta*) recorded at 500 MHz in $CD_2Cl_2$. The bottoms inset shows a zoom-in on the *p*-H peaks, which are annotated with their respective frequency difference, *Δv*. The top inset shows a zoom-in on peaks hidden in the noise level.

**Note S4.** It will be elaborated briefly, how we arrived at the estimated ranges of $k_{se}$ for the **$^{[2.2.2]}K_n$-$[Fe_4S_4]^{2+/3+}$**, **$K_n$-$[Fe_4S_4]^{2+/3+}$** (where $n$=2,1) and **$^{[2.2.2]}K_m$-$[Fe_4S_4]^{2+/3+}$-Im*** (where $m$=1,0) systems, based on the phenomenological appearance of the (VT) NMR spectra of their respective redox mixtures (*vide supra*; Figures S50, S51 and S53).

Three distinct cases/regimes are known to exist for exchange phenomena affecting the NMR line shapes, namely.[23, 24]

(1) $k \ll \Delta v$, which leads to appearance of the NMR resonances of the two exchanging species as separate signals; each bearing the position and width of the native species unaffected by exchange. This is, *i.e.* the case for all signals observed in the redox mixture of **$K_n$-$[Fe_4S_4]^{2+/3+}$** (where $n$=2,1), leading us to conclude that ETse is either extremely slow or, which is in our view more likely, inhibited.

(2) $k \approx \Delta v$, which leads to exchange broadening of the resonances; sometimes even to the degree of being non-detectable. Though the signals have significantly broader linewidths, they are still located at the respective positions of the native species. The broadening effect is most pronounced, the more similar $k$ and $\Delta v$ are. This type of behavior is observed *i.e.* for all signals of the **$^{[2.2.2]}K_n$-$[Fe_4S_4]^{2+/3+}$** (where $n$=2,1) system.

(3) $k \gg \Delta v$, which leads to the averaging of the two resonance lines. The two signals then appear as a single broadened peak, which is located between the respective positions of the native species; its exact position depending on the concentration ratio between the two exchanging systems. The faster the exchange process is compared to $\Delta v$, the sharper the averaged line appears. Such behavior is observed for the *p*-H resonances in **$^{[2.2.2]}K_m$-$[Fe_4S_4]^{2+/3+}$-Im*** (where $m$=1,0).

**Summary of the Hamiltonian Description of the Odd-electron "Pair-of-Pairs" by Suess and co-workers**

The simplified pair-of-pairs Hamiltonians[25-27] for the two valence isomers (denoted "A" and "B") introduced in their work are summarized in equations S1 and S2, namely:

$$\hat{H}_{\mathrm{VI_A}} = \left[\sum_{1\leq p<q\leq 4} J_{\mathrm{A}}\vec{S}_p\cdot\vec{S}_q\right] - BV_{34}T_{34} + \frac{J_{\mathrm{A}}}{n}\left(\vec{S}_1\cdot\vec{S}_2\right) - \frac{J_{\mathrm{A}}}{n}\left(\vec{S}_3\cdot\vec{S}_4\right), \quad \text{(S1)}$$

and

$$\hat{H}_{\mathrm{VI_B}} = \left[\sum_{1\leq p<q\leq 4} J_{\mathrm{B}}\vec{S}_p\cdot\vec{S}_q\right] - BV_{12}T_{12} - \frac{J_{\mathrm{B}}}{n}\left(\vec{S}_1\cdot\vec{S}_2\right) + \frac{J_{\mathrm{B}}}{n}\left(\vec{S}_3\cdot\vec{S}_4\right), \quad \text{(S2)}$$

respectively.[28] Here, $J_{\mathrm{A,B}}$ denote the valence isomers' global $J$ values, and $B$ is the double exchange value, which together parameterize the six Fe-Fe superexchange interactions and the one double exchange interaction. The operator $V$ reproduces the correct spin dependencies for the energetic gain of double exchange, whereby $T_{ij}$ represents the (symmetric) transfer operator for the electron hopping from one of the two mixed-valent site to the other.[29, 30] It is further assumed that the exchange couplings of the reduced/oxidized $Fe_2S_2$ subunits have a fixed relationship to the global exchange coupling ($J$) *via* the parameter $n$:

$\Delta J_{\mathrm{ox}} = J/n$ ; and $\Delta J_{\mathrm{red}} = -J/n$ , respectively, (S3)

which effectively allows to parameterize the exchange coupling space of both valence isomers with four unique parameters, namely $J_{\mathrm{A}}$, $J_{\mathrm{B}}$, $B$ and $n$. By convention, the valence isomer energy difference, $\Delta E_{\mathrm{VI}}$, as such, then is:

$$\Delta E_{\mathrm{VI}} = E(\mathrm{VI_B}) - E(\mathrm{VI_A}) . \quad \text{(S4)}$$

In this work, we maintained the Suess group's convention, Fe no. 4 being the uniquely ligated Fe site, but for our $[Fe_4S_4]^{3+}$ system, we initially assigned $S_4$ to the minority oxidation state, $Fe^{2+}$, with $S$=2 (*i.e.* Fe no. 3 and Fe no. 4 constituting the lower-valent pair in isomer "A"). This means that positive values of $\Delta E_{\mathrm{VI}}$ favor localization of the minority Fe valence (effectively $Fe^{2.5+}$) at the unique site, while negative values favor the majority Fe valence ($Fe^{3+}$). Ultimately, the energies of the eigenstates ($E_i$) of these two Hamiltonians relate to the paramagnetic chemical shift values of a ligand *via* equation S5:[31]

$$\delta_{\mathrm{para}} = \frac{g_{\mathrm{e}}\mu_{\mathrm{B}}}{3\hbar\gamma_{\mathrm{I}}k_{\mathrm{B}}T} A_{\mathrm{m,eff}} \frac{\sum_i C_{i\mathrm{m}} S_i(S_i+1)(2S_i+1)e^{-E_i/(k_{\mathrm{B}}T)}}{\sum_i (2S_i+1)e^{-E_i/(k_{\mathrm{B}}T)}}, \quad \text{(S5)}$$

where $A_{\mathrm{m}}$ is the fictitious electron-nucleus hyperfine coupling constant and $C_{i\mathrm{m}}$ is a spin projection coefficient that describes the projection of metal site m's spin onto the total spin of a given eigenstate *i*.[19] Note that $A_{\mathrm{m,eff}}$ hereby also parameterizes asymmetry in the double-exchange, which traditionally goes by the variable $\beta$ ($0.5\geq\beta\geq 0$ for a $[Fe^{(3-\beta)}Fe^{(2+\beta)}S_2]^{1+}$ mixed-valent pair), to describe the asymmetry of exchange in $[Fe_2S_2]^{1+}$ pairs in which the Fe sites do not possess identical ligands. Furthermore, $A_{\mathrm{m,eff}}$ is unique for each nucleus and valence isomer. By expressing $\delta_{\mathrm{para}}$ in this way, Suess and co-workers describe the observed $\delta_{\mathrm{para}}$ as a result of the canonical ensemble description of all populated states of the two valence isomers, allowing, in turn, to model their respective spin-ladders based on VT $^1$H NMR data. To accommodate our system, only the variation of two additional chemical shift values had to be

considered, necessitating very little modification of their analytical routine.[19, 32] For transparency and clarity, the MATLAB codes we adapted and modified from their work to suit our purposes are reproduced in a section below. Naturally, we considered the correction the authors published to their code shortly afterward.[32]

**Modelling Valence Isomerism in the Non-Canonical $[Fe_4S_4]^{2+}$ Cubanes**

As anticipated based on their diamagnetism, the VT $^1H$ NMR spectra of $^{[2.2.2]}K_2[Fe_4S_4(DmpS)_4]$, $^{[2.2.2]}K[Fe_4S_4(DmpS)_3(Im^*)]$ and $[Fe_4S_4(DmpS)_2(Im^*)_2]$ do not display a strong variation of the $^1H$ chemical shifts with temperature (Figures S42-S44 and S48). However, we hypothesized that the mixed ligation in $[Fe_4S_4(DmpS)_3(Im^*)]^-$ and $[Fe_4S_4(DmpS)_2(Im^*)_2]$ could still provide a useful probe to evaluate the magnetic structure of the cubane, because the $DmpS^-$ resonances offer complementary information with regard to the Im* resonances about the local Fe sites. After all, VT NMR data has been used since the late 90s to model the magnetic parameters of (canonical) $[Fe_4S_4]^{2+}$ complexes, but as far as we are aware, it was never attempted to model the behavior of 3:1 or 2:2 site-differentiated $[Fe_4S_4]^{2+}$ clusters with this method.
Therefore, in this section of the Supporting Information, we describe our efforts in modelling the magnetic structure, of these two complexes, and, additionally, propose a hypothesis on how these models may be used to gain insight into the "internal" electron transfer thermodynamics of the cubanes based on fits to the experimental VT-NMR data.

Because the variation of the $\delta$-values is very small, and it is known to be challenging to differentiate the *B*-description *vs.* the *J*/*n*- or *ΔJ*-description for the favored localization of the two $[Fe_2S_2]^{1+}$ pairs in the $[Fe_4S_4]^{2+}$ oxidation state with fitting routines [29], we would like to emphasize that the quantitative results of the simulations presented in this section should be interpreted with caution. Circumnavigating at least some ambiguity in our results, we decided to fix the double-exchange term, *B*, to 400 $cm^{-1}$, a value that is grossly in between the ones determined on Suess's $[Fe_4S_4]^{1+}$ clusters[19, 32] and the one determined by us on $[Fe_4S_4(DmpS)_3(Im^*)]$ ($[Fe_4S_4]^{3+}$; *vide supra*), and also close to the values established by Mouesca and co-workers in the literature for canonical $[Fe_4S_4]^{2+}$ complexes.[29] Interestingly, $[Fe_4S_4(DmpS)_3(Im^*)]^-$ and $[Fe_4S_4(DmpS)_2(Im^*)_2]$ exhibit a significantly lower solution state magnetic moment than their canonical congener (Figure S54A and S56A). The following paragraphs will discuss our efforts in modelling the magnetic structure of these two systems, and the associated implications.
For the 3:1 substituted cubane, $[Fe_4S_4(DmpS)_3(Im^*)]^-$, we consider the traditional Hamiltonian given in equation S6:

$$\hat{H} = \left[\sum_{1\le p<q\le 4} J\vec{S}_p \cdot \vec{S}_q\right] - BV_{34}T_{34} - BV_{12}T_{12} + \frac{J}{n}\left(\vec{S}_1 \cdot \vec{S}_2\right) + \frac{J}{n}\left(\vec{S}_3 \cdot \vec{S}_4\right) . \quad \text{(S6)}$$

To simplify the parameter space further, and avoid overfitting, we also assume that, in the $[Fe_4S_4]^{2+}$ oxidation state, there is only one valence isomer, and thus only one effective superexchange coupling term, *J*. However, one of the pairs can be subject to asymmetric (polarized) double exchange, which influences the spin-projection factors for state *i* and metal center m, $C_{i\mathrm{m}}$, as summarized in equations S7 and S8:[31]

$$C_{i\mathrm{m}} = \frac{\alpha_\mathrm{m}\gamma_\mathrm{m}}{\Delta_\mathrm{m}} , \quad \text{(S7)}$$

where

$$\alpha_\mathrm{m} = \{\beta[S_\mathrm{mn}(S_\mathrm{mn}+1) + S_\mathrm{m}(S_\mathrm{m}+1) - S_\mathrm{n}(S_\mathrm{n}+1)] + (1-\beta)[S_\mathrm{mn}(S_\mathrm{mn}+1) - S_\mathrm{m}(S_\mathrm{m}+1) + S_\mathrm{n}(S_\mathrm{n}+1)]\}/2 ,$$

$$\gamma_\mathrm{m} = [S(S+1) + S_\mathrm{mn}(S_\mathrm{mn}+1) - S_\mathrm{kl}(S_\mathrm{kl}+1)]/2 \text{ ,and}$$

$$\Delta_{\mathrm{m}} = S_{\mathrm{mn}}(S_{\mathrm{mn}} + 1)S(S + 1) \;, \tag{S8}$$

respectively. Here, double exchange is symmetric for $\beta$=0.5 and asymmetric for any other value. If $\beta$=0 or $\beta$=1, there is no delocalization, and the transfer operator, $T$, in Equation S1 becomes zero, disabling exchange. The indices m, n, k and l refer to the four iron sites of the cubane. Therefore, on the basis of this model and to evaluate isomerism, double exchange is forced to be completely asymmetric in one of the two pairs ($[Fe^{(2+\beta)}Fe^{(3-\beta)}S_2]^{1+}$; $\beta$=0 or $\beta$=1), and the two valence isomers consequently constitute the two limiting cases. Fitting of this model to the data (Figure S54) produces the following parameters: $J$=323 cm$^{-1}$, $n$=–1.25, $B$=400 cm$^{-1}$ (fixed) and $\Delta E_{VI}$=±408 cm$^{-1}$ (inverse signs for $\beta$=0 or $\beta$=1, respectively) with a RMSE of 0.59. The thus estimated global $J$-coupling falls in between the corresponding values determined for the $[Fe_4S_4]^{1+}$ and $[Fe_4S_4]^{3+}$ complexes. Nonetheless, we would like to emphasize that a limitation of this model is the treatment of the (ficticious) valence isomers as the limiting cases of asymmetric double exchange. Whereas the model is—apparently—able to describe this data well, the physical reality is most likely of a more continuous nature, lying somewhere (or entirely) intermediately between these two extremes.

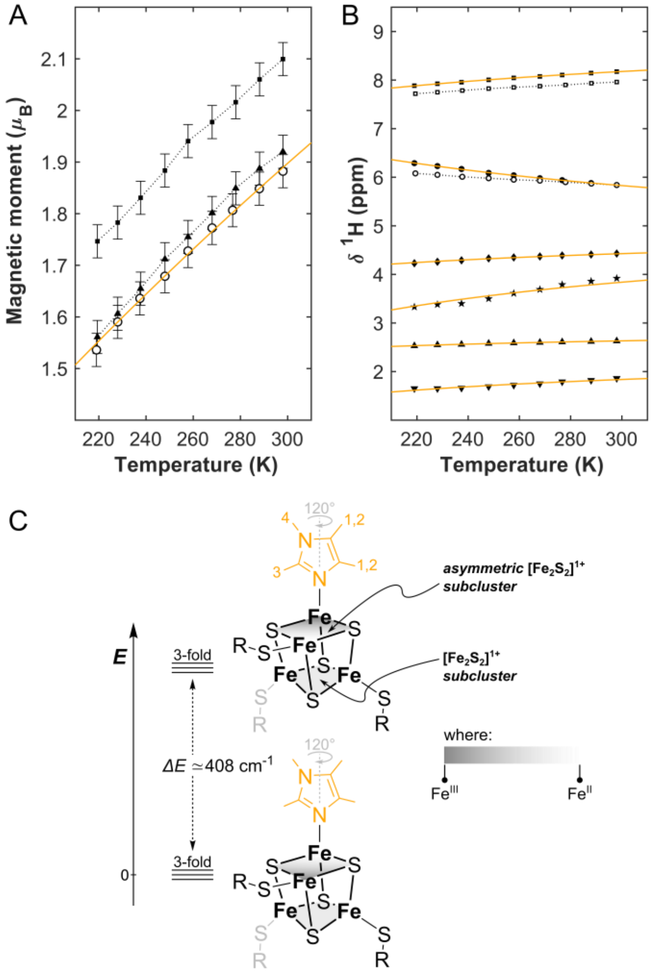


**Figure S54.** (A) Evans method magnetic moments for $^{[2.2.2]}K[Fe_4S_4(DmpS)_3(Im^*)]$ (*dots*) *vs.* temperature and best global fit of the data (*yellow trace*). For comparison, the data for $^{[2.2.2]}K_2[Fe_4S_4(DmpS)_4]$ (*squares*) and $[Fe_4S_4(DmpS)_2(Im^*)_2]$ (*triangles*) are also shown. (B) Plot of the DmpS⁻-ligand's aryl *para*-H and *meta*-H chemical

shifts as well as the Im* methyl group's protons chemical shifts (*solid black markers*) against temperature. *Yellow traces* indicate the best global fit. For comparison, the variation of the homoleptic system's DmpS⁻ ligand's aryl *para*-H and *meta*-H are shown as *hollow black markers*. The legend is the same as in Figure 11. (C) Schematic depiction of the ground state valence isomeric forms of $[Fe_4S_4(DmpS)_3(Im^*)]^-$ according to the model fitted to the VT $^1$H NMR data and described in the main text.

To illustrate this, we provide the fitted parameters for the system forced into completely symmetric double exchange ($\beta$=0.5), namely: $J$=323 cm$^{-1}$, $n$=–1.25, $B$=400 cm$^{-1}$ (fixed) and $\Delta E_{VI}$=1500 cm$^{-1}$ with identical RMSE of 0.59. Intriguingly, the magnetic and hyperfine parameters are very similar to those of the two limiting cases (for hyperfine parameters refer to Tables S16-S18), but $\Delta E_{VI}$ is very large. It is in fact so large, that the practical interpretation of this value is that it is effectively zero, because the (identical and fictitious) excited isomer cannot be significantly populated at the evaluated temperatures. This is supported by the fact that equivalent magnetic parameters can also be obtained if $\Delta E_{VI}$ is fixed to be zero, $\beta$=0.5, and only a single spin ladder is considered. While our analysis of the limiting cases of double exchange polarization clearly invokes $\Delta E_{VI}$>0, namely the favoured localization of $Fe^{2+}$ at the unique site, it remains unclear whether this type of isomerism really occurs in this system. Nonetheless, in the context of our efforts in modelling the data, we propose that this result may offer an alternative perspective on the physical interpretation of the $\Delta E_{VI}$-value of the 3:1 differentiated even-electron $[Fe_4S_4]^{2+}$ cubane obtained from these simulations. Namely being associated with the thermodynamic energy differences required for a discrete internal ET within the unique $[Fe_2S_2]^{1+}$ subcluster. Such an internal ET would polarize the cubane into a state that is "pre-activated" for a spatially directed ET. In contrast to the odd-electron systems in 3:1 symmetry, $\Delta E_{VI}$ of $[Fe_4S_4(DmpS)_3(Im^*)]^-$ therefore does not describe the polarization of the cubane in a persistent state. Instead, $\Delta E_{VI}$ may be interpreted as the hypothetical energy difference between the more localized states on the same potential energy surface, which could be populated fleetingly, en-route to the odd-electron redox states (Figure S55B), without altering the gross magnetic properties of the system. Of course, these considerations only apply under the assumption that internal electron transfer precedes outer-sphere electron transfer.

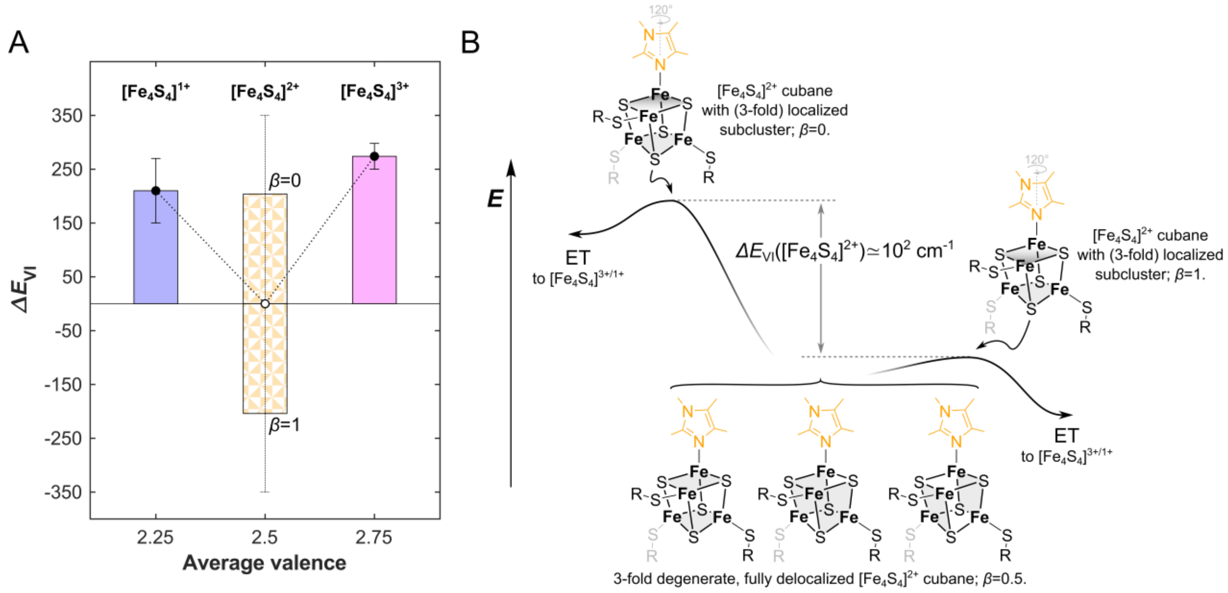


**Figure S55.** (A) Comparison between the (hypothetical) magnitudes of $\Delta E_{VI}$-values of 3:1 site-differentiated cubanes in the three oxidation states, $[Fe_4S_4]^{1+}$ (*blue*), $[Fe_4S_4]^{2+}$ (*yellow*) and $[Fe_4S_4]^{3+}$ (*magenta*), respectively. The black markers with error bars indicate the energetic position and its uncertainty, while the filled bars illustrate the absolute magnitude for ease of comparison and clarity. Because the $\Delta E_{VI}$-value of the $[Fe_4S_4]^{2+}$ cluster does

not have the same physical meaning as the other ones, it is represented with a checkered area and a hollow marker, and is associated with a large uncertainty. (B) Schematic illustration of the proposed interpretation of the $\Delta E_{VI}$-value of $[Fe_4S_4(DmpS)_3(Im^*)]^-$.

For 2:2 symmetry, we set-up the pair-of-pairs Hamiltonians of the two isomeric forms of $[Fe_4S_4(DmpS)_2(Im^*)_2]$ as equations S9 and S10:

$$\widehat{H}_{\mathrm{VI_A}} = \left[\sum_{1\le p<q\le 4} J\vec{S}_p \cdot \vec{S}_q\right] - BV_{34}T_{34} - BV_{12}T_{12} + \frac{J}{n}\left(\vec{S}_1 \cdot \vec{S}_2\right) + \frac{J}{n}\left(\vec{S}_3 \cdot \vec{S}_4\right) , \qquad \text{(S9)}$$

And

$$\widehat{H}_{\mathrm{VI_B}} = \left[\sum_{1\le p<q\le 4} J\vec{S}_p \cdot \vec{S}_q\right] - BV_{13}T_{13} - BV_{24}T_{24} + \frac{J}{n}\left(\vec{S}_1 \cdot \vec{S}_3\right) + \frac{J}{n}\left(\vec{S}_2 \cdot \vec{S}_4\right) , \qquad \text{(S10)}$$

respectively. In this formalism, like for the previous (3:1 symmetric) case of the $[Fe_4S_4]^{2+}$ cubane, the double exchange coupling, *B*, the global couplings, *J*, and the factor *n* are assumed to be common to both isomers. The parameter space is thus also smaller than the one in $[Fe_4S_4(DmpS)_3(Im^*)]$. Within this approach, we adopted the convention according to which the isomer at zero energy (isomer "A") is the non-degenerate one, *i.e.* the one whose individual Fe sites in the $[Fe_2S_2]^{1+}$ subclusters possess identical coordination spheres. To account for the mixed ligation of the $[Fe_2S_2]^{1+}$ subclusters in isomer "B", we attempted to implement a double-exchange polarization term, $\beta_B$. Like for the 3:1 case, we found that this model can describe the *T*-evolution of the $^1H$ paramagnetic shifts as well as the *T*-dependence of the solution state magnetic moment reasonably well for all limiting values of $\beta_B$. Among the many possible solutions, the exemplary set of parameters assuming symmetric double exchange ($\beta_B$=0.5) is *J*=316 cm$^{-1}$, *n*=–1.25, and *B*=400 cm$^{-1}$ (fixed) with an RMSE of 0.30 (Figure S56A and S56B). In this model $\Delta E_{VI}$ is estimated to be extremely large, so, in effect, ~0 cm$^{-1}$. Modelling internal electron transfers on this potential energy surface as in the 3:1 case *via* the implementation of double exchange polarization, and maintaining the gross magnetic properties, yields $\Delta E_{VI}>0$ if $Fe^{3+}$ is localized at imidazole, and, *vice-versa*, $\Delta E_{VI}<0$, if $Fe^{2+}$ is localized there (Figure S56C), while the values of *J*, *n* and the RMSE remain unchanged.

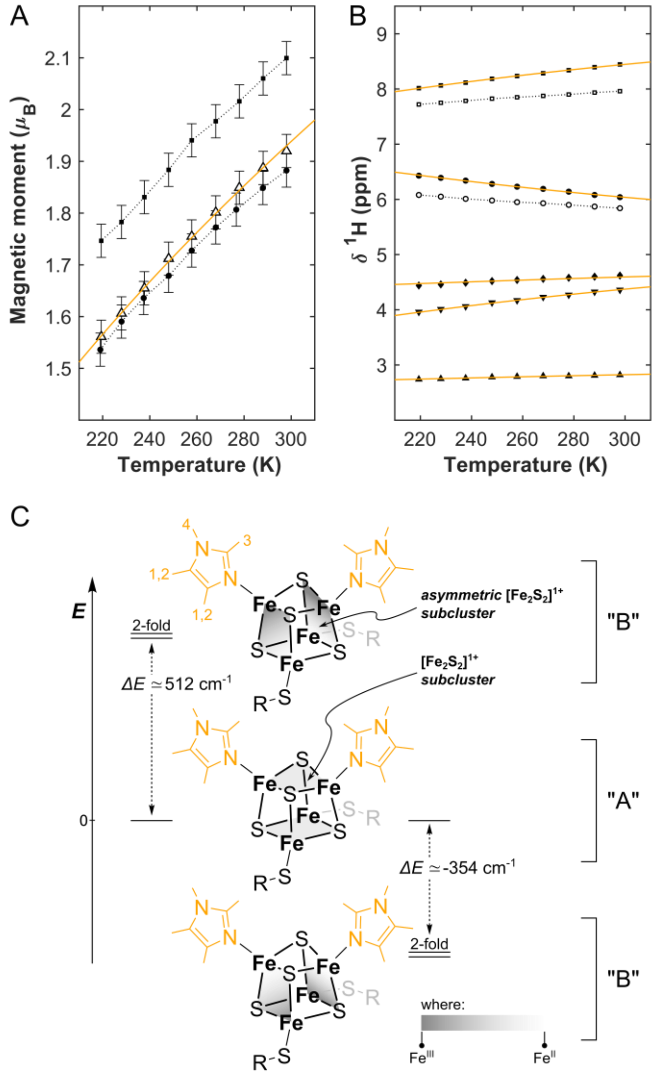


**Figure S56.** (A) Evans method magnetic moments for $[Fe_4S_4(DmpS)_2(Im^*)_2]$ (*triangles*) *vs.* temperature and best global fit of the data (*yellow trace*). For comparison, the data for $^{[2.2.2]}K_2[Fe_4S_4(DmpS)_4]$ (*squares*) and $[Fe_4S_4(DmpS)_3(Im^*)]^-$ (*dots*) are also shown. (B) Plot of the DmpS$^-$-ligand's aryl *para*-H and *meta*-H chemical shifts as well as the Im* methyl group's protons chemical shifts (*solid black markers*) against temperature. *Yellow traces* indicate the best global fit. For comparison, the variation of the homoleptic system's DmpS$^-$ ligand's aryl *para*-H and *meta*-H are shown as *hollow black markers*. The legend is the same as in Figure 11. (C) Schematic depiction of the ground state magnetic isomers "A" and "B" of $[Fe_4S_4(DmpS)_2(Im^*)_2]$ according to the model fitted to the VT $^1$H NMR data.

## Further Details and Results of the VT $^1$H NMR Fits

**Table S16.** Fitted hyperfine coupling constants for the protons in $[Fe_4S_4(DmpS)_3(Im^*)]$. Values are given with standard deviation.

| proton | $A(Fe^{3+})$ (MHz) | $A_{eff}(Fe^{2.5+})$ (MHz) |
|---|---:|---:|
| **$DmpS^-$ / *p*-H** | –0.537±0.016 | –1.462±0.049 |
| **$DmpS^-$ / *m*-H** | 0.727±0.016 | 2.001±0.056 |
| **Im* / $H^1$[a]** | 0.188±0.009[a] | –0.226±0.001[a] |
| **Im* / $H^2$[a]** | 0.074±0.010[a] | –0.163±0.001[a] |
| **Im* / $H^3$** | ~0.0 | –0.104±0.001 |
| **Im* / $H^4$** | –0.031±0.007 | 0.009±0.001 |

[a] Can be either proton 1 or proton 2 of the Im* ligand.[19]

**Table S17.** Fitted diamagnetic $^1$H NMR shift values and hyperfine coupling constants in $[Fe_4S_4(DmpS)_3(Im^*)]^-$.

| proton | $\delta_{dia}$ (ppm)[a] | | | $A(Fe^{2.5+})$ (MHz) | | |
|---|---:|---:|---:|---:|---:|---:|
| **$\beta$-value** | 0 | 0.5 | 1 | 0 | 0.5 | 1 |
| **$DmpS^-$ / *p*-H** | 6.41 | 6.45 | 6.41 | -0.327 | –0.329 | –0.327 |
| **$DmpS^-$ / *m*-H** | 8.59 | 8.54 | 8.59 | 0.209 | 0.210 | 0.209 |
| **Im* / $H^1$[a]** | 0.42[a] | 0.18[a] | 0.42[a] | 0.464[a] | 0.454[a] | 0.464[a] |
| **Im* / $H^2$[a]** | 0.68[a] | 0.55[a] | 0.63[a] | 0.161[a] | 0.157[a] | 0.161[a] |
| **Im* / $H^3$** | 2.09 | 2.05 | 2.09 | 0.072 | 0.070 | 0.072 |
| **Im* / $H^4$** | 3.33 | 3.25 | 3.33 | 0.147 | 0.144 | 0.147 |

[a] The shift corresponds to either proton 1 or 2 of the Im* ligand.[19]

**Table S18.** Fitted diamagnetic $^1$H NMR shift values and hyperfine coupling constants in $[Fe_4S_4(DmpS)_2(Im^*)_2]$.

| proton | $\delta_{dia}$ (ppm)[a] | | | $A(Fe^{2.5+})$ (MHz) | | |
|---|---:|---:|---:|---:|---:|---:|
| **$\beta$-value** | 0 | 0.5 | 1 | 0 | 0.5 | 1 |
| **$DmpS^-$ / *p*-H** | 6.16 | 5.77 | 6.09 | –0.231 | –0.288 | –0.427 |
| **$DmpS^-$ / *m*-H** | 8.12 | 8.48 | 8.18 | 0.253 | 0.315 | 0.467 |
| **Im* / $H^1$[a]**<br>**Im* / $H^2$[a]** | 2.29[a] | 1.84[a] | 2.29[a] | 0.227[a] | 0.297[a] | 0.464[a] |
| **Im* / $H^3$** | 2.34 | 2.32 | 2.33 | 0.055 | 0.058 | 0.063 |
| **Im* / $H^4$** | 3.69 | 3.50 | 3.70 | 0.101 | 0.132 | 0.222 |

[a] The shift corresponds to either proton 1 or 2 of the Im* ligand.[19]

## Computational Details for Simulation of $^{57}$Fe Mössbauer and EPR Parameters

All DFT calculations of $^{57}$Fe Mössbauer and EPR parameters were carried out with the ORCA 4.2.0 program package.[33, 34] Throughout this work, we apply the TPSS density functional[35] if not stated otherwise. This density functional was chosen after a careful benchmark against the PBE, PBE0, B3LYP, and TPSSh functionals.

All molecular structures discussed were fully optimized. For each structural model, we truncated the DmpS-ligands with MeS-moieties to reduce the overall computational cost.
The Ahlrichs' def2-TZVP basis set[36] was applied on all atoms, and the CP(PPP) core-polarized basis functions are applied for all iron atoms.[37]
Grimme's DFT-D3 dispersion correction,[38] complemented with the Becke-Johnson damping function[39] was employed in all calculations. All charges and spin populations were obtained from Löwdin population analyses.[40]
For calculations involving an implicit solvent model, the CPCM solvent model[41] for electrostatic screening with a dielectric constant of 2.4 (toluene) was used.
Relativistic effects were not included, since it was shown that they do not improve the quality of Mössbauer parameters.[37]

The relative directions and coupling of the individual site spin vectors at ferric or ferrous iron sites is an essential issue in generating the desired density functional solution, and six different valence isomers are possible. We generated broken-symmetry (BS) solutions corresponding to the procedure introduced by Noodleman and coworkers[42] and implemented in ORCA. The excess of $\alpha$- and $\beta$-spin density described by the $< S_{z,\mathrm{Fe}} >$ expectation value is calculated for all iron atoms to characterize the broken-spin symmetry wave function.[43]

*<u>Details on $^{57}$Fe Mössbauer Parameter Calculations with DFT</u>*

The quadrupole splitting was calculated according to:

$$\Delta E_Q = \frac{1}{2}\, q\, Q\, V_{zz} \left(1 + \frac{\eta^2}{3}\right)^{\frac{1}{2}} , \tag{S11}$$

where $q$ is the electrical charge of an electron and $Q$ is the nuclear quadrupole moment of an Fe nucleus (approximately 0.16 barns). $V_{xx}, V_{yy}$ and $V_{zz}$ are the electric field gradient tensors, and $\eta$ is an asymmetry parameter defined as:

$$\eta = \left| \frac{V_{xx} - V_{yy}}{V_{zz}} \right| . \tag{S12}$$

$^{57}$Fe Mössbauer isomer shifts $\delta$ are calculated according to:

$$\delta = \alpha\,(\rho - C) + \beta \tag{S13}$$

with the fit parameters $\alpha, \beta, C$ depending on the applied density functional. $\rho$ denotes the electron density at the absorbing Fe nucleus.

The following fit parameters were applied in this work:

**Table S19.** Fitting parameters for the evaluation of $\delta$ according to Eq. (S13) for the density functionals TPSS, TPSSh, PBE0 and B3LYP.

| | $\boldsymbol{\alpha}$ [mm s$^{-1}$bohr$^{3}$] | $\boldsymbol{\beta}$ [mm s$^{-1}$] | $\boldsymbol{C}$ [bohr$^{-3}$] | **reference** |
|---|---|---|---|---|
| **B3LYP** | -0.366 | 2.852 | 11810.0 | [44] |
| **TPSS** | -0.421 | 5.154 | 11810.0 | [44] |
| **TPSSh** | -0.376 | 4.13 | 11810.0 | [44] |
| **PBE0** | -0.36191 | 0.28881 | 11819.1 | [45] |

We assessed the sensitivity of calculated spin coupling patterns, that is the distribution of $\alpha/\beta$-spin excess density across the iron nuclei, and corresponding $^{57}$Fe Mössbauer parameters applying the fit parameters from Table S19 for different density functionals.
As we find TPSS to overall yield the best agreement with experimental Mössbauer data, this density functional will be applied in all further calculations.
Furthermore, we assessed the sensitivity of calculated $^{57}$Fe Mössbauer parameters on the inclusion of an implicitly described solvent. Our results show that solvent models do not need to be included in the calculations, as they have only minor effects on calculated Mössbauer parameters.

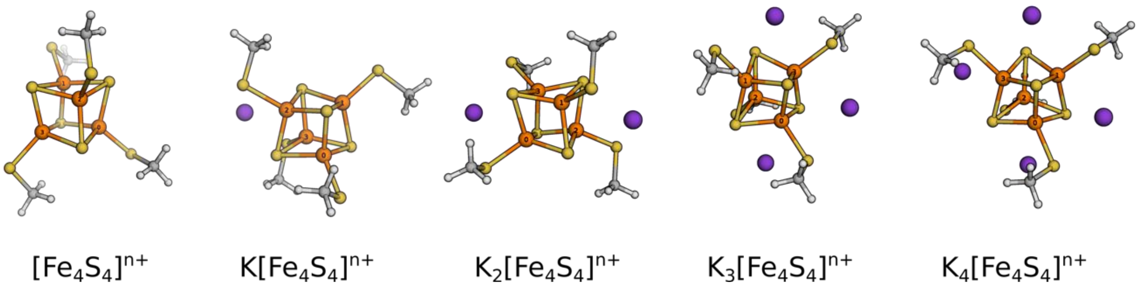


**Figure S57.** Overview of molecular structures studied computationally throughout this work (*n*=1,2,3).

**Table S20.** Summary of compounds characterized by means of DFT in this work, expected number of valence isomers, corresponding charges, spin multiplicities, and *g*-tensors calculated for the ground state valence isomer.

| **complex** | **approx. no. VI**[a] | **charge** | ***S*** | **($g_x$, $g_y$, $g_z$)** | **$g_{av}$** | ***g*-strains** |
|---|---|---|---|---|---|---|
| **K-[$Fe_4S_4$]$^{3+}$** | 3 | 0 | 1/2 | (1.978, 2.007, 2.035) | **2.006** | (0.029, 0.000, 0.029) |
| **[$Fe_4S_4$]$^{3+}$** | 1 | -1 | 1/2 | (2.014, 2.018, 2.029) | **2.020** | (0.007, 0.003, 0.008) |
| **$K_2$-[$Fe_4S_4$]$^{2+}$** | 2 | 0 | 0 | - | **-** | - |
| **[$Fe_4S_4$]$^{2+}$** | 1 | -2 | 0 | - | **-** | - |
| **$K_4$-[$Fe_4S_4$]$^{1+}$** | 1 | 1 | 1/2 | (1.959, 1.976, 2.026) | **1.986** | (0.028, 0.011, 0.039) |
| **$K_3$-[$Fe_4S_4$]$^{1+}$** | 3 | 0 | 1/2 | (1.947, 1.974, 2.021) | **1.980** | (0.034, 0.007, 0.040) |
| **$K_2$-[$Fe_4S_4$]$^{1+}$** | 2 | -1 | 1/2 | (1.959, 1.970, 2.016) | **1.982** | (0.023, 0.012, 0.034) |
| **K-[$Fe_4S_4$]$^{1+}$** | 3 | -2 | 1/2 | (1.957, 1.971, 2.017) | **1.981** | (0.025, 0.011, 0.036) |
| **[$Fe_4S_4$]$^{1+}$** | 1 | -3 | 1/2 | (1.965, 1.989, 2.013) | **1.989** | (0.023, 0.000, 0.024) |

[a] Based on the symmetry considerations outlined in Figure S1 (*vide supra*).

<u>*DFT-energy splitting of valence isomers, and calculated $^{57}$Fe Mössbauer and EPR parameters*</u>

**$[Fe_4S_4]^{2+}$**

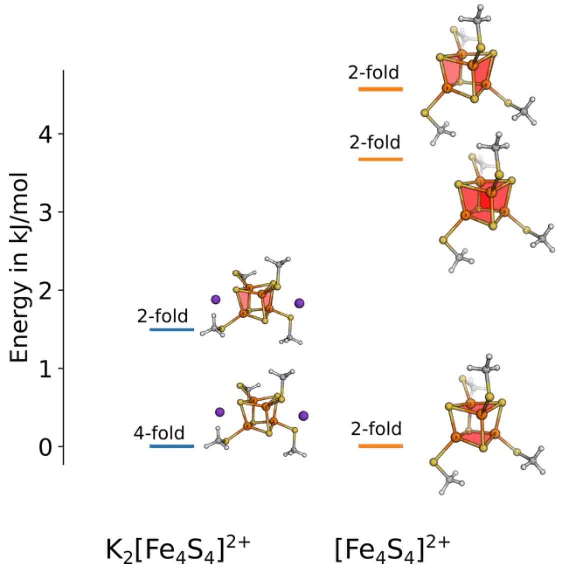


**Figure S58.** Energy-splitting of valence isomers in **$K_x$-$[Fe_4S_4]^{2+}$** with $x$=0,2. The Fe-pairs on which $\beta$-electron density is accumulated are indicated as shaded planes. For the ground state valence isomers in **$K_2$-$[Fe_4S_4]^{2+}$**, the Fe-pairs $Fe_1$-$Fe_3$, $Fe_2$-$Fe_4$, $Fe_1$-$Fe_2$, and $Fe_3$-$Fe_4$ are energetically equivalent, resulting in 4-fold degeneracy, and coloring of the planes is therefore omitted for better visibility.

**Table S21.** Calculated $^{57}$Fe Mössbauer parameters for the energetically lowest-lying valence isomers of **$K_x$-$[Fe_4S_4]^{2+}$** with $x$=0,2. Bold values indicate that $\beta$-spin excess density is located at these iron sites.

| | **complex** | **$Fe_1$** | **$Fe_2$** | **$Fe_3$** | **$Fe_4$** |
|---|---|---|---|---|---|
| $\eta$ | $K_2$-$[Fe_4S_4]^{2+}$ | 0.61 | 0.91 | 0.92 | 0.62 |
| | $[Fe_4S_4]^{2+}$ | 0.69 | 0.58 | 0.34 | 0.39 |
| $\delta$ [mm s$^{-1}$] | $K_2$-$[Fe_4S_4]^{2+}$ | 0.20 | 0.20 | 0.20 | 0.20 |
| | $[Fe_4S_4]^{2+}$ | 0.26 | 0.26 | 0.26 | 0.26 |
| $\Delta E_Q$ [mm s$^{-1}$] | $K_2$-$[Fe_4S_4]^{2+}$ | -1.08 | -1.06 | -1.05 | -1.09 |
| | $[Fe_4S_4]^{2+}$ | 0.87 | 0.93 | 0.86 | 0.90 |

**Table S22.** Calculated [57]Fe Mössbauer parameters for the energetically higher-lying valence isomers of **$K_2$-[$Fe_4S_4$]$^{2+}$**. Complexes are enumerated in ascending order depending on the energy gap to the ground state isomer listed in Table S21.

| Isomer | $\Delta E$ $[\mathrm{kJ\ mol^{-1}}]$ | Fe with $\beta$-spin density | $\eta$ | $\delta\ [\mathrm{mm\ s^{-1}}]$ | $\Delta E_Q\ [\mathrm{mm\ s^{-1}}]$ |
|---|---|---|---|---|---|
| 1 | + 0.0 | 2 / 4 | 0.61 / 0.92 / 0.92 / 0.61 | 0.21 / 0.20 / 0.20 / 0.20 | -1.08 / -1.06 / -1.05 / -1.09 |
| 2 | + 0.0 | 3 / 4 | 0.92 / 0.61 / 0.61 / 0.92 | 0.20 / 0.20 / 0.20 / 0.20 | -1.05 / -1.09 / -1.09 / -1.05 |
| 3 | + 0.0 | 1 / 2 | 0.92 / 0.61 / 0.61 / 0.92 | 0.20 / 0.20 / 0.20 / 0.20 | -1.05 / -1.10 / -1.09 / -1.05 |
| 4 | + 1.5 | 1 / 4 | 0.59 / 0.58 / 0.58 / 0.60 | 0.20 / 0.20 / 0.20 / 0.20 | -0.54 / -0.54 / -0.54 / -0.53 |
| 5 | + 1.5 | 2 / 3 | 0.59 / 0.58 / 0.58 / 0.60 | 0.20 / 0.20 / 0.20 / 0.20 | -0.54 / -0.54 / -0.54 / -0.53 |

**Table S23.** Calculated [57]Fe Mössbauer parameters for the energetically higher-lying valence isomers of **[$Fe_4S_4$]$^{2+}$**. Complexes are enumerated in ascending order depending on the energy gap to the ground state isomer listed in Table S21.

| Isomer | $\Delta E$ $[\mathrm{kJ\ mol^{-1}}]$ | Fe with $\beta$-spin density | $\eta$ | $\delta\ [\mathrm{mm\ s^{-1}}]$ | $\Delta E_Q\ [\mathrm{mm\ s^{-1}}]$ |
|---|---|---|---|---|---|
| 1 | + 0.0 | 2 / 3 | 0.69 / 0.58 / 0.34 / 0.30 | 0.26 / 0.26 / 0.26 / 0.26 | 0.87 / 0.94 / 0.85 / 0.90 |
| 2 | + 3.7 | 1 / 2 | 0.49 / 0.25 / 0.68 / 0.59 | 0.26 / 0.26 / 0.26 / 0.26 | 0.86 / 0.89 / 0.90 / 0.78 |
| 3 | + 3.7 | 3 / 4 | 0.49 / 0.25 / 0.68 / 0.59 | 0.26 / 0.26 / 0.26 / 0.26 | 0.86 / 0.89 / 0.90 / 0.78 |
| 4 | + 4.6 | 1 / 3 | 0.32 / 0.61 / 0.51 / 0.54 | 0.26 / 0.26 / 0.26 / 0.26 | 0.82 / 0.93 / 0.91 / 0.93 |
| 5 | + 4.6 | 2 / 4 | 0.32 / 0.62 / 0.52 / 0.54 | 0.26 / 0.26 / 0.26 / 0.26 | 0.83 / 0.93 / 0.92 / 0.92 |

## $[Fe_4S_4]^{3+}$

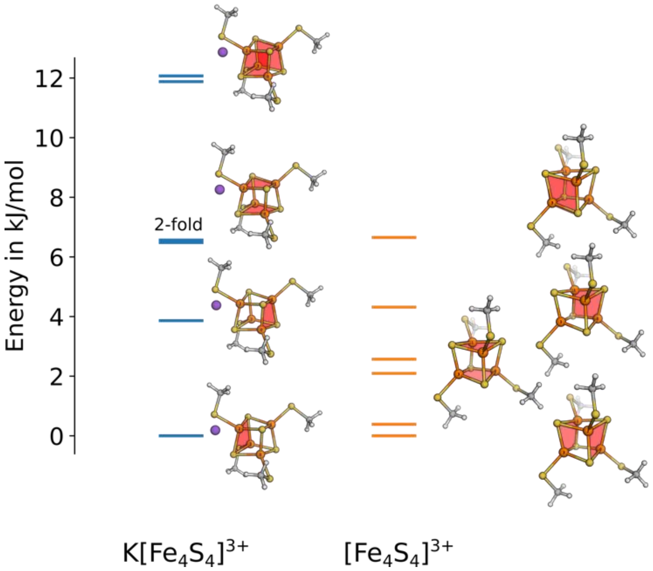


**Figure S59.** Energy-splitting of valence isomers in **$K_x$-$[Fe_4S_4]^{3+}$** with $x$=0, 1. The Fe-pairs on which $\beta$-electron density is accumulated are indicated as shaded planes.

**Table S24.** Calculated $^{57}$Fe Mössbauer parameters for the energetically lowest-lying valence isomers in **$K_x$-$[Fe_4S_4]^{3+}$** with $x$=0,1. Bold values indicate that beta spin excess density is located at these iron sites.

| | complex | $Fe_1$ | $Fe_2$ | $Fe_3$ | $Fe_4$ |
|---|---|---|---|---|---|
| $\eta$ | K-$[Fe_4S_4]^{3+}$ | 0.29 | 0.32 | **0.14** | **0.08** |
| | $[Fe_4S_4]^{3+}$ | **0.16** | 0.52 | **0.42** | 0.71 |
| $\delta$ [mm s$^{-1}$] | K-$[Fe_4S_4]^{3+}$ | 0.18 | 0.18 | **0.13** | **0.13** |
| | $[Fe_4S_4]^{3+}$ | **0.14** | 0.20 | **0.14** | 0.20 |
| $\Delta E_Q$ [mm s$^{-1}$] | K-$[Fe_4S_4]^{3+}$ | 1.36 | 1.36 | **0.84** | **0.84** |
| | $[Fe_4S_4]^{3+}$ | **-0.68** | 1.08 | **-0.64** | 1.14 |

**Table S25.** Calculated EPR parameters for all valence isomers in **K-[$Fe_4S_4$]$^{3+}$**. The complexes are enumerated in ascending order depending on the energy gap to the ground state isomer. $A_{xx}, A_{yy}, A_{zz}$ and $A_{iso}$ are given in MHz.

| Isomer | $\Delta E$ [$\mathbf{kJ\ mol^{-1}}$] | Fe with $\boldsymbol{\beta}$-spin density | parameter | $Fe_1$ | $Fe_2$ | $Fe_3$ | $Fe_4$ |
|---|---|---|---|---|---|---|---|
| 1 | 0.0 | 3 / 4 | $g_{x,y,z}$ | 1.978 / 2.005 / 2.034 | | | |
| | | | $g_{iso}$ | 2.0059 | | | |
| | | | *g*-strains | 0.0279 / 0.0009 / 0.0281 | | | |
| | | | $A_{xx}$ | -15.14 | -15.26 | 21.37 | 19.86 |
| | | | $A_{yy}$ | -27.18 | -26.95 | 23.22 | 22.16 |
| | | | $A_{zz}$ | -33.58 | -34.02 | 36.51 | 36.85 |
| | | | $A_{iso}$ | -25.30 | -25.41 | 27.03 | 26.29 |
| 2 | + 3.9 | 1 / 2 | $g_{x,y,z}$ | 2.009 / 2.026 / 2.065 | | | |
| | | | $g_{iso}$ | 2.033 | | | |
| | | | *g*-strains | 0.0244 / 0.0074 / 0.0316 | | | |
| | | | $A_{xx}$ | 10.03 | 10.48 | -29.31 | -28.55 |
| | | | $A_{yy}$ | 26.35 | 26.28 | -32.49 | -32.51 |
| | | | $A_{zz}$ | 37.90 | 38.56 | -40.08 | -39.73 |
| | | | $A_{iso}$ | 24.76 | 25.11 | -33.96 | -33.60 |
| 3 | + 6.5 | 2 / 3 | $g_{x,y,z}$ | 2.002/ 2.025 / 2.037 | | | |
| | | | $g_{iso}$ | 2.0210 | | | |
| | | | *g*-strains | 0.019 / 0.004 / 0.016 | | | |
| | | | $A_{xx}$ | -22.98 | 14.72 | 16.20 | -20.10 |
| | | | $A_{yy}$ | -30.92 | 22.41 | 30.81 | -29.90 |
| | | | $A_{zz}$ | -36.91 | 42.69 | 35.78 | -38.47 |
| | | | $A_{iso}$ | -30.27 | 26.61 | 27.60 | -29.49 |
| 4 | + 6.6 | 1 / 4 | $g_{x,y,z}$ | 2.000 / 2.021 / 2.033 | | | |
| | | | $g_{iso}$ | 2.0182 | | | |
| | | | *g*-strains | 0.0182 / 0.0028 / 0.0158 | | | |
| | | | $A_{xx}$ | 13.90 | -23.21 | -20.56 | 17.89 |
| | | | $A_{yy}$ | 21.18 | -31.26 | -29.94 | 30.86 |
| | | | $A_{zz}$ | 41.79 | -37.09 | -38.44 | 35.53 |
| | | | $A_{iso}$ | 25.62 | -30.52 | -29.68 | 28.09 |
| 5 | + 11.9 | 1 / 3 | $g_{x,y,z}$ | 1.993 / 2.022 / 2.034 | | | |
| | | | $g_{iso}$ | 2.0169 | | | |
| | | | *g*-strains | 0.023 / 0.005 / 0.018 | | | |
| | | | $A_{xx}$ | 15.13 | -24.87 | 15.83 | -17.91 |
| | | | $A_{yy}$ | 23.78 | -33.33 | 31.15 | -27.12 |
| | | | $A_{zz}$ | 43.90 | -38.20 | 33.24 | -38.40 |
| | | | $A_{iso}$ | 27.60 | -32.13 | 26.74 | -27.81 |
| 6 | + 12.1 | 2 / 4 | $g_{x,y,z}$ | 1.995 / 2.026 / 2.037 | | | |
| | | | $g_{iso}$ | 2.0193 | | | |
| | | | *g*-strains | 0.0243 / 0.0067 / 0.0177 | | | |
| | | | $A_{xx}$ | -25.44 | 14.73 | -18.05 | 16.68 |
| | | | $A_{yy}$ | -33.41 | 23.25 | -27.11 | 32.34 |
| | | | $A_{zz}$ | -37.88 | 43.99 | -38.61 | 33.38 |
| | | | $A_{iso}$ | -32.24 | 27.33 | -27.92 | 27.47 |

**$[Fe_4S_4]^{1+}$**

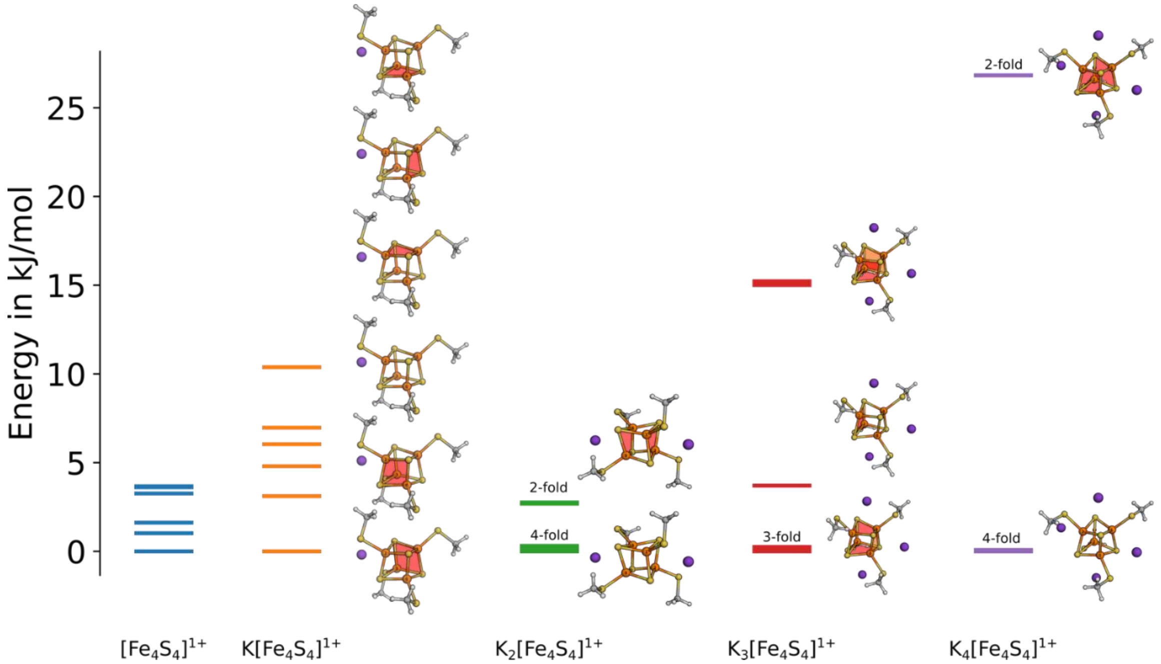


**Figure S60.** Energy-splitting of valence isomers in **$[Fe_4S_4]^{1+}$** in the presence and absence of $K^+$ cations. The Fe-pairs on which $\beta$-electron density is accumulated are indicated as shaded planes. Depiction of molecular clusters are omitted for **$[Fe_4S_4]^{1+}$** for better visibility.

**Table S26.** Calculated $^{57}$Fe Mössbauer parameters for the energetically lowest-lying valence isomers in **$K_x$-$[Fe_4S_4]^{1+}$** with $x$=0,1,2,3,4. Bold values indicate that $\beta$-spin excess density is located at these iron sites.

| | complex | $Fe_1$ | $Fe_2$ | $Fe_3$ | $Fe_4$ |
|---|---|---|---|---|---|
| $\eta$ | $K_4$-$[Fe_4S_4]^{1+}$ | 0.31 | 0.29 | **0.13** | **0.16** |
| | $K_3$-$[Fe_4S_4]^{1+}$ | **0.21** | 0.70 | 0.90 | **0.12** |
| | $K_2$-$[Fe_4S_4]^{1+}$ | 0.55 | 0.82 | **0.47** | **0.42** |
| | K-$[Fe_4S_4]^{1+}$ | 0.79 | **0.61** | 0.67 | **0.65** |
| | $[Fe_4S_4]^{1+}$ | **0.28** | 0.95 | 0.73 | **0.82** |
| $\delta$ [mm s$^{-1}$] | $K_4$-$[Fe_4S_4]^{1+}$ | 0.23 | 0.23 | **0.29** | **0.29** |
| | $K_3$-$[Fe_4S_4]^{1+}$ | **0.31** | 0.25 | 0.25 | **0.30** |
| | $K_2$-$[Fe_4S_4]^{1+}$ | 0.27 | 0.27 | **0.33** | **0.31** |
| | K-$[Fe_4S_4]^{1+}$ | 0.31 | **0.35** | 0.32 | **0.38** |
| | $[Fe_4S_4]^{1+}$ | **0.41** | 0.35 | 0.25 | **0.38** |
| $\Delta E_Q$ [mm s$^{-1}$] | $K_4$-$[Fe_4S_4]^{1+}$ | -0.70 | -0.71 | **0.93** | **0.95** |
| | $K_3$-$[Fe_4S_4]^{1+}$ | **1.05** | -1.16 | -1.10 | **0.81** |
| | $K_2$-$[Fe_4S_4]^{1+}$ | -1.01 | 0.83 | **1.40** | **1.01** |
| | K-$[Fe_4S_4]^{1+}$ | 0.64 | **1.58** | 1.14 | **1.90** |
| | $[Fe_4S_4]^{1+}$ | **2.40** | -0.92 | 0.71 | **1.60** |

**Table S27.** Calculated EPR parameters for all valence isomers in **K-[$Fe_4S_4$]$^{1+}$**. The complexes are enumerated in ascending order depending on the energy gap to the ground state isomer. $A_{xx}, A_{yy}, A_{zz}$and $A_{iso}$ are given in MHz.

| Isomer | $\Delta E$ $[\mathbf{kJ\ mol^{-1}}]$ | Fe with $\boldsymbol{\beta}$-spin density | parameter | $Fe_1$ | $Fe_2$ | $Fe_3$ | $Fe_4$ |
|---|---|---|---|---|---|---|---|
| 1 | 0.0 | 2 / 4 | $g_{x.y,z}$ | 1.957 / 1.972 / 2.018 | | | |
| | | | $g_{iso}$ | 1.9822 | | | |
| | | | *g*-strains | 0.0252 / 0.0102 / 0.0358 | | | |
| | | | $A_{xx}$ | -31.15 | 9.00 | -28.38 | -0.07 |
| | | | $A_{yy}$ | -36.97 | 34.53 | -36.88 | 32.51 |
| | | | $A_{zz}$ | -46.79 | 45.80 | -45.75 | 48.54 |
| | | | $A_{iso}$ | -38.3 | 29.78 | -37.00 | 26.99 |
| 2 | + 3.1 | 1 / 3 | $g_{x.y,z}$ | 1.970 / 1.980 / 2.020 | | | |
| | | | $g_{iso}$ | 1.9899 | | | |
| | | | *g*-strains | 0.0199 / 0.0099 / 0.0301 | | | |
| | | | $A_{xx}$ | 14.84 | -33.86 | -6.39 | -26.1 |
| | | | $A_{yy}$ | 33.88 | -41.21 | 30.09 | -36.87 |
| | | | $A_{zz}$ | 46.70 | -47.13 | 52.41 | -45.49 |
| | | | $A_{iso}$ | 31.81 | -40.73 | 25.37 | -36.15 |
| 3 | + 4.8 | 3 / 4 | $g_{x.y,z}$ | 1.969 / 1.978 / 2.024 | | | |
| | | | $g_{iso}$ | 1.9900 | | | |
| | | | *g*-strains | 0.021 / 0.012 / 0.034 | | | |
| | | | $A_{xx}$ | -29.95 | -26.56 | 22.65 | -6.2 |
| | | | $A_{yy}$ | -39.77 | -37.17 | 34.93 | 37.79 |
| | | | $A_{zz}$ | -45.28 | -44.81 | 46.23 | 45.78 |
| | | | $A_{iso}$ | -38.33 | -36.18 | 34.61 | 25.79 |
| 4 | + 6.0 | 2 / 3 | $g_{x.y,z}$ | 1.964 / 1.977 / 2.013 | | | |
| | | | $g_{iso}$ | 1.9848 | | | |
| | | | *g*-strains | 0.0208 / 0.0078 / 0.0282 | | | |
| | | | $A_{xx}$ | -33.52 | 6.87 | -0.88 | -28.19 |
| | | | $A_{yy}$ | -41.22 | 33.53 | 32.98 | -36.89 |
| | | | $A_{zz}$ | -48.94 | 46.64 | 47.45 | -46.07 |
| | | | $A_{iso}$ | -41.23 | 29.01 | 26.52 | -37.05 |
| 5 | + 6.9 | 1 / 2 | $g_{x.y,z}$ | 1.960 / 1.998 / 2.028 | | | |
| | | | $g_{iso}$ | 1.9954 | | | |
| | | | *g*-strains | 0.0354 / 0.0026 / 0.0326 | | | |
| | | | $A_{xx}$ | -14.20 | 7.39 | -33.24 | -33.03 |
| | | | $A_{yy}$ | 28.38 | 21.90 | -35.34 | -35.02 |
| | | | $A_{zz}$ | 53.19 | 48.27 | -43.55 | -41.12 |
| | | | $A_{iso}$ | 22.45 | 25.85 | -37.37 | -36.39 |
| 6 | + 10.4 | 1 / 4 | $g_{x.y,z}$ | 1.970 / 1.979 / 1.997 | | | |
| | | | $g_{iso}$ | 1.9818 | | | |
| | | | *g*-strains | 0.0118 / 0.0028 / 0.0152 | | | |
| | | | $A_{xx}$ | 17.76 | -34.28 | -28.06 | -8.74 |
| | | | $A_{yy}$ | 37.85 | -42.11 | -37.13 | 32.06 |
| | | | $A_{zz}$ | 49.43 | -48.33 | -46.84 | 48.77 |
| | | | $A_{iso}$ | 35.02 | -41.57 | -37.34 | 24.03 |

**Table S28.** Calculated EPR parameters for all valence isomers in **$K_2$-[$Fe_4S_4$]$^{1+}$**. The complexes are enumerated in ascending order depending on the energy gap to the ground state isomer. $A_{xx}, A_{yy}, A_{zz}$and $A_{iso}$ are given in MHz.

| Isomer | $\Delta E$ [$\mathbf{kJ\ mol^{-1}}$] | Fe with $\boldsymbol{\beta}$-spin density | parameter | $Fe_1$ | $Fe_2$ | $Fe_3$ | $Fe_4$ |
|---|---|---|---|---|---|---|---|
| 1 | 0.0 | 3 / 4 | $g_{x.y,z}$ | 1.961 / 1.973 / 2.016 | | | |
| | | | $g_{iso}$ | 1.9833 | | | |
| | | | *g*-strains | 0.0224 / 0.0104 / 0.0326 | | | |
| | | | $A_{xx}$ | -28.04 | -29.95 | -1.58 | 22.18 |
| | | | $A_{yy}$ | -31.99 | -31.87 | 29.99 | 30.06 |
| | | | $A_{zz}$ | -46.28 | -44.09 | 43.50 | 32.83 |
| | | | $A_{iso}$ | -35.42 | -35.3 | 23.97 | 28.36 |
| 2 | +0.0 | 1 / 2 | $g_{x.y,z}$ | 1.957 / 1.970 / 2.015 | | | |
| | | | $g_{iso}$ | 1.9806 | | | |
| | | | *g*-strains | 0.0236 / 0.0106 / 0.0344 | | | |
| | | | $A_{xx}$ | 22.33 | -1.42 | -30.02 | -27.99 |
| | | | $A_{yy}$ | 29.99 | 30.36 | -31.95 | -31.96 |
| | | | $A_{zz}$ | 32.72 | 43.11 | -44.41 | -46.40 |
| | | | $A_{iso}$ | 28.35 | 24.02 | -35.46 | -35.45 |
| 3 | +0.1 | 2 / 4 | $g_{x.y,z}$ | 1.958 / 1.971 / 2.016 | | | |
| | | | $g_{iso}$ | 1.9816 | | | |
| | | | *g*-strains | 0.0236 / 0.0106 / 0.0344 | | | |
| | | | $A_{xx}$ | -29.98 | 22.13 | -28.03 | -1.70 |
| | | | $A_{yy}$ | -31.82 | 30.10 | -32.05 | 29.89 |
| | | | $A_{zz}$ | -44.09 | 32.75 | -46.35 | 43.56 |
| | | | $A_{iso}$ | -35.3 | 28.32 | -35.48 | 23.92 |
| 4 | +0.3 | 1 / 3 | $g_{x.y,z}$ | 1.960 / 1.972 / 2.016 | | | |
| | | | $g_{iso}$ | 1.9827 | | | |
| | | | *g*-strains | 0.0227 / 0.0107 / 0.0333 | | | |
| | | | $A_{xx}$ | -1.99 | -27.75 | 22.03 | -29.99 |
| | | | $A_{yy}$ | 30.00 | -31.95 | 30.40 | -31.83 |
| | | | $A_{zz}$ | 43.72 | -46.26 | 33.01 | -44.01 |
| | | | $A_{iso}$ | 23.91 | -35.32 | 28.48 | -35.28 |
| 5 | +2.7 | 1 / 4 | $g_{x.y,z}$ | 1.955 / 1.995 / 2.039 | | | |
| | | | $g_{iso}$ | 1.9965 | | | |
| | | | *g*-strains | 0.0415 / 0.0015 / 0.0425 | | | |
| | | | $A_{xx}$ | -12.16 | -30.42 | -29.59 | 20.22 |
| | | | $A_{yy}$ | 30.90 | -33.31 | -33.76 | 27.57 |
| | | | $A_{zz}$ | 45.70 | -40.96 | -40.88 | 39.01 |
| | | | $A_{iso}$ | 21.48 | -34.90 | -34.74 | 28.93 |
| 6 | +2.8 | 2 / 3 | $g_{x.y,z}$ | 1.955 / 1.995 / 2.039 | | | |
| | | | $g_{iso}$ | 1.9961 | | | |
| | | | *g*-strains | 0.0411 / 0.0011 / 0.0429 | | | |
| | | | $A_{xx}$ | -30.20 | 19.97 | -12.33 | -29.85 |
| | | | $A_{yy}$ | -33.12 | 27.57 | 31.13 | -33.66 |
| | | | $A_{zz}$ | -40.72 | 39.06 | 45.86 | -40.92 |
| | | | $A_{iso}$ | -34.68 | 28.87 | 21.55 | -34.81 |

**Table S29.** Calculated EPR parameters for all valence isomers in **$K_3$-[$Fe_4S_4$]$^{1+}$**. The complexes are enumerated in ascending order depending on the energy gap to the ground state isomer. $A_{xx}, A_{yy}, A_{zz}$ and $A_{iso}$ are given in MHz.

| Isomer | $\Delta E$ [kJ mol$^{-1}$] | Fe with $\beta$-spin density | parameter | $Fe_1$ | $Fe_2$ | $Fe_3$ | $Fe_4$ |
|---|---|---|---|---|---|---|---|
| 1 | + 0.0 | 1 / 4 | $g_{x,y,z}$ | 1.945 / 1.972 / 2.020 | | | |
| | | | $g_{iso}$ | 1.9791 | | | |
| | | | *g*-strains | 0.0341 / 0.0071 / 0.0419 | | | |
| | | | $A_{xx}$ | 5.98 | -23.19 | -23.78 | 12.67 |
| | | | $A_{yy}$ | 27.75 | -28.23 | -28.25 | 27.08 |
| | | | $A_{zz}$ | 38.29 | -43.58 | -43.67 | 35.94 |
| | | | $A_{iso}$ | 24.01 | -31.66 | -31.90 | 25.23 |
| 2 | + 0.1 | 1 / 3 | $g_{x,y,z}$ | 1.963 / 1.995 / 2.024 | | | |
| | | | $g_{iso}$ | 1.9943 | | | |
| | | | *g*-strains | 0.0335 / 0.0185 / 0.0305 | | | |
| | | | $A_{xx}$ | -14.77 | -26.64 | 23.07 | -26.31 |
| | | | $A_{yy}$ | 30.41 | -33.03 | 26.84 | -28.66 |
| | | | $A_{zz}$ | 42.94 | -35.95 | 34.81 | -41.27 |
| | | | $A_{iso}$ | 19.53 | -31.87 | 28.24 | -32.08 |
| 3 | + 0.2 | 2 / 4 | $g_{x,y,z}$ | 1.963 / 1.994 / 2.024 | | | |
| | | | $g_{iso}$ | 1.9939 | | | |
| | | | *g*-strains | 0.0303 / 0.0007 / 0.0297 | | | |
| | | | $A_{xx}$ | -26.19 | 22.97 | -26.69 | -14.47 |
| | | | $A_{yy}$ | -28.33 | 27.12 | -33.07 | 30.43 |
| | | | $A_{zz}$ | -41.29 | 34.39 | -36.16 | 42.69 |
| | | | $A_{iso}$ | -31.94 | 28.16 | -31.98 | 19.55 |
| 4 | + 3.7 | 2 / 3 | $g_{x,y,z}$ | 1.969 / 1.979 / 2.029 | | | |
| | | | $g_{iso}$ | 1.9925 | | | |
| | | | *g*-strains | 0.0235 / 0.0135 / 0.0365 | | | |
| | | | $A_{xx}$ | -26.02 | 8.14 | 8.48 | -25.96 |
| | | | $A_{yy}$ | -31.99 | 28.09 | 27.97 | -32.07 |
| | | | $A_{zz}$ | -40.54 | 29.93 | 30.02 | -40.72 |
| | | | $A_{iso}$ | -32.85 | 22.05 | 22.16 | -32.92 |
| 5 | + 15.0 | 3 / 4 | $g_{x,y,z}$ | 1.973 / 1.980 / 2.001 | | | |
| | | | $g_{iso}$ | 1.9848 | | | |
| | | | *g*-strains | 0.0118 / 0.0048 / 0.0162 | | | |
| | | | $A_{xx}$ | -28.06 | -26.37 | 13.14 | -11.25 |
| | | | $A_{yy}$ | -33.79 | -30.12 | 25.09 | 27.74 |
| | | | $A_{zz}$ | -43.46 | -40.21 | 40.02 | 50.46 |
| | | | $A_{iso}$ | -35.11 | -32.23 | 26.08 | 22.31 |
| 6 | + 15.2 | 1 / 2 | $g_{x,y,z}$ | 1.973 / 1.979 / 2.003 | | | |
| | | | $g_{iso}$ | 1.9848 | | | |
| | | | *g*-strains | 0.0118 / 0.0058 / 0.0182 | | | |
| | | | $A_{xx}$ | -11.31 | 13.44 | -26.42 | -28.41 |
| | | | $A_{yy}$ | 27.67 | 25.08 | -30.10 | -33.92 |
| | | | $A_{zz}$ | 50.48 | 40.12 | -40.10 | -43.74 |
| | | | $A_{iso}$ | 22.28 | 26.22 | -32.21 | -35.36 |

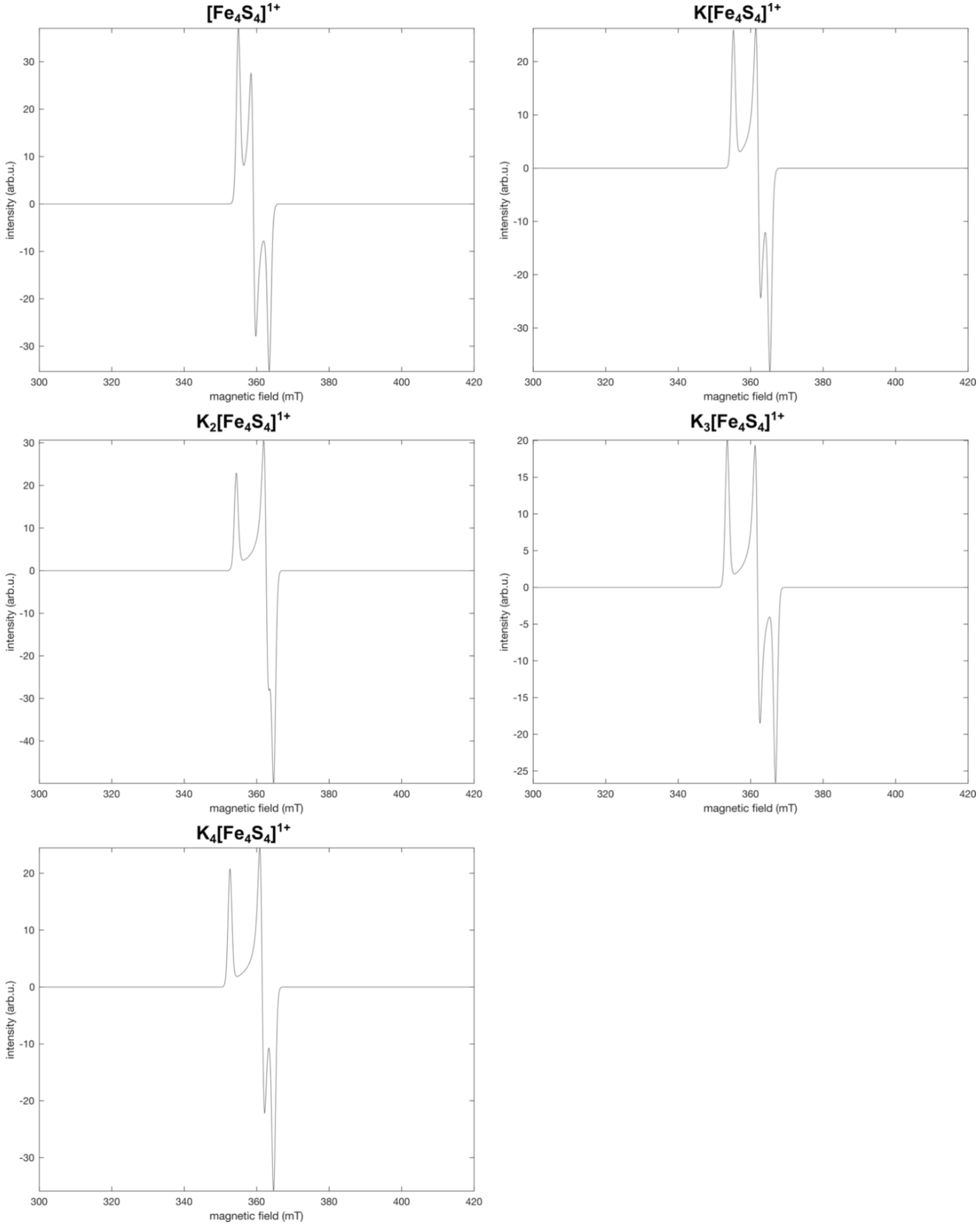


**Figure S61.** Calculated EPR spectra for the energetically lowest valence isomer of **$K_x$-$[Fe_4S_4]^{1+}$** with $x$=(0,1,2,3,4). The gaussian line width for isotropic broadening was set to 1.0. The spectrometer frequency was set to 10.0 GHz for X-band.

## DFT Simulations of $^{57}Fe$ NRVS

Initial coordinates for DFT simulation of $^{57}Fe$ NRVS for complexes **$K_2$-[$Fe_4S_4$]$^{2+}$** and **K-[$Fe_4S_4$]$^{3+}$**, as well as **$^{[2.2.2]}K_2$-[$Fe_4S_4$]$^{2+}$** and **$^{[2.2.2]}$K-[$Fe_4S_4$]$^{3+}$** were based on the X-ray structures determined previously.[1, 2] While the 4×DmpS$^-$ ligands to the [$Fe_4S_4$] cubane cores were included explicitly without truncations, the [2.2.2]-cryptand and co-crystallized solvent molecules from the crystal cells were excluded in these models, implied in their **$^{[2.2.2]}K_2$-[$Fe_4S_4$]$^{2+}$** to **[$Fe_4S_4$]$^{2+}$** and **$^{[2.2.2]}$K-[$Fe_4S_4$]$^{3+}$** to **[$Fe_4S_4$]$^{3+}$** re-designations. This produced systems of 208-210 atoms, depending on the number (0-2) of $K^+$ ions encapsulated in vicinity to the FeS core. The structural optimization and subsequent normal mode analysis were done using GAUSSIAN 16 Revision C.01,[46] based on the electron densities exported from single point calculations using JAGUAR 11.0,[47] the latter providing high-quality initial guess including broken-symmetry[42, 48, 49] (BS) states construction. The BP86[50, 51] functional customized with 5% admixture of Hartree-Fock (HF) exact exchange, indicated to provide optimal account of Fe-S bond covalency,[52] and the LACV3P** basis set as implemented in JAGUAR were employed. The model environment was considered using a self-consistent reaction field (SCRF) polarizable continuum model and integral equation formalism (IEF-PCM)[53] as implemented in GAUSSIAN, with the static dielectric constant set to ε=4.0, and the remaining IEF-PCM parameters at their default values for water. Inclusion of two-body D3 empirical dispersion correction[38] was tested, however the DFT scheme that omitted the D3 correction has been presently found to fit better the structural and spectral data from the experiment. Based on the normal mode outputs from GAUSSIAN, an in-house Q-SPECTOR tool successfully applied previously (e.g. refs.[54-56]) was utilized to generate the $^{57}Fe$ partial vibrational density of states (PVDOS). The resolution of the observed NRVS spectra was accounted for by convolution of the computed PVDOS intensities with a full width at half maximum (FWHM)=14 $cm^{-1}$ Lorentzian. For $^{57}Fe$-PVDOS, empirical scaling was applied to the calculated frequencies by ×1.04 in the ~350-430 $cm^{-1}$ (mostly Fe-S(thiolate)) and by ×1.05/1.10 for (**$K_2$-**)**[$Fe_4S_4$]$^{2+}$**/(**K-**)**[$Fe_4S_4$]$^{3+}$** respectively in the ~230-340 $cm^{-1}$ (mostly Fe-S($\mu^3$)) $^{57}Fe$ spectral regions.

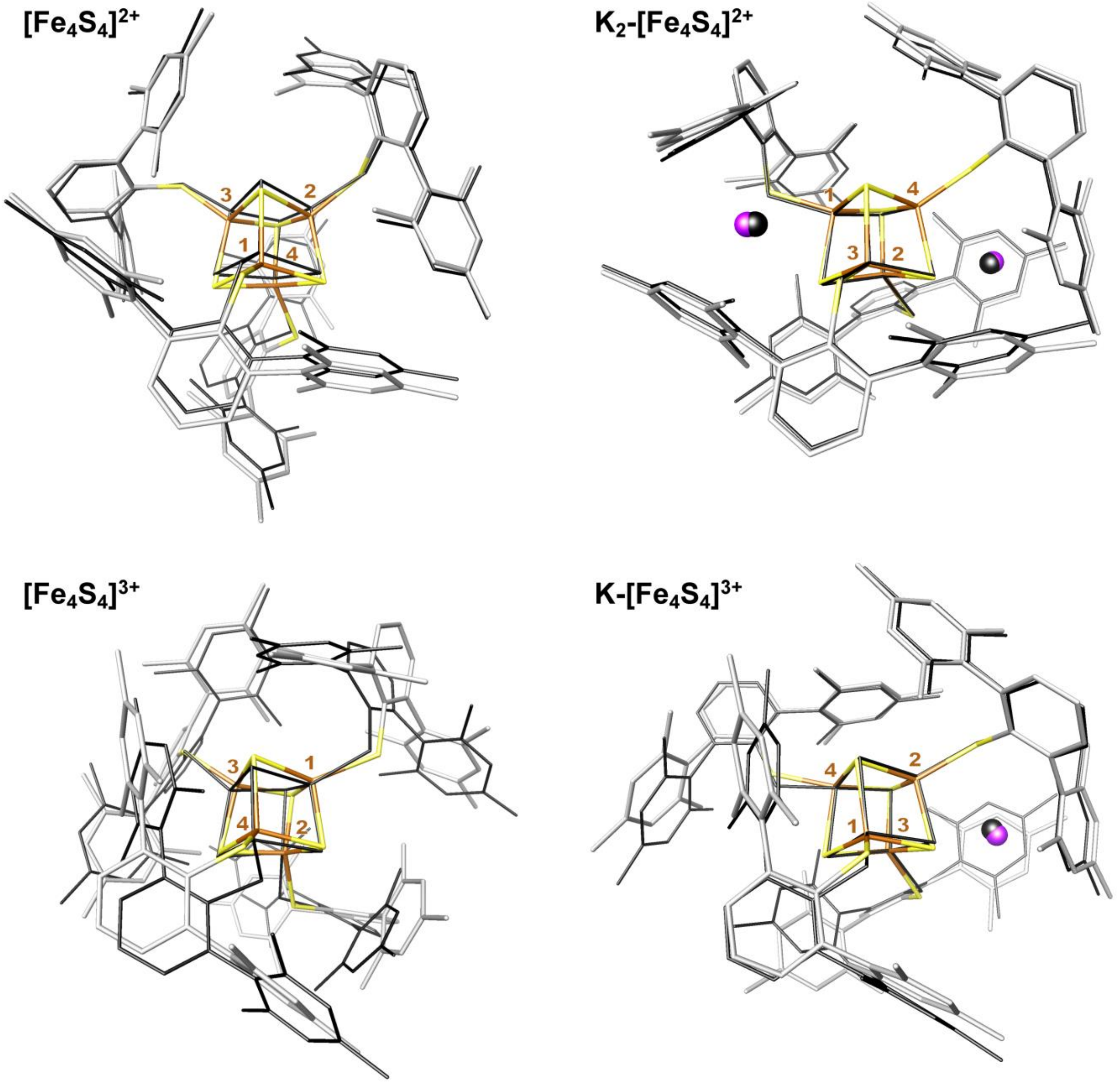


**Figure S62.** Structures of complexes **K*x*-[Fe$_4$S$_4$]$^{2+}$** (top; *x*=0 left / *x*=2 right) and **K*x*-[Fe$_4$S$_4$]$^{3+}$** (bottom; *x*=0 left / *x*=1 right) from the extended DFT modeling (element colors, tube representation), superimposed with their X-ray references[1, 2] (black wire). The K$^+$ ions are shown as balls. Hydrogen atoms are omitted for clarity. The Fe atoms are numbered (1-4) following their crystallographic order. In all molecular orientations shown, the two Fe sites characterized by excess *α*-electron density (for the ground-state spin isomer, Table S30) occupy the front-left side of the cubane.

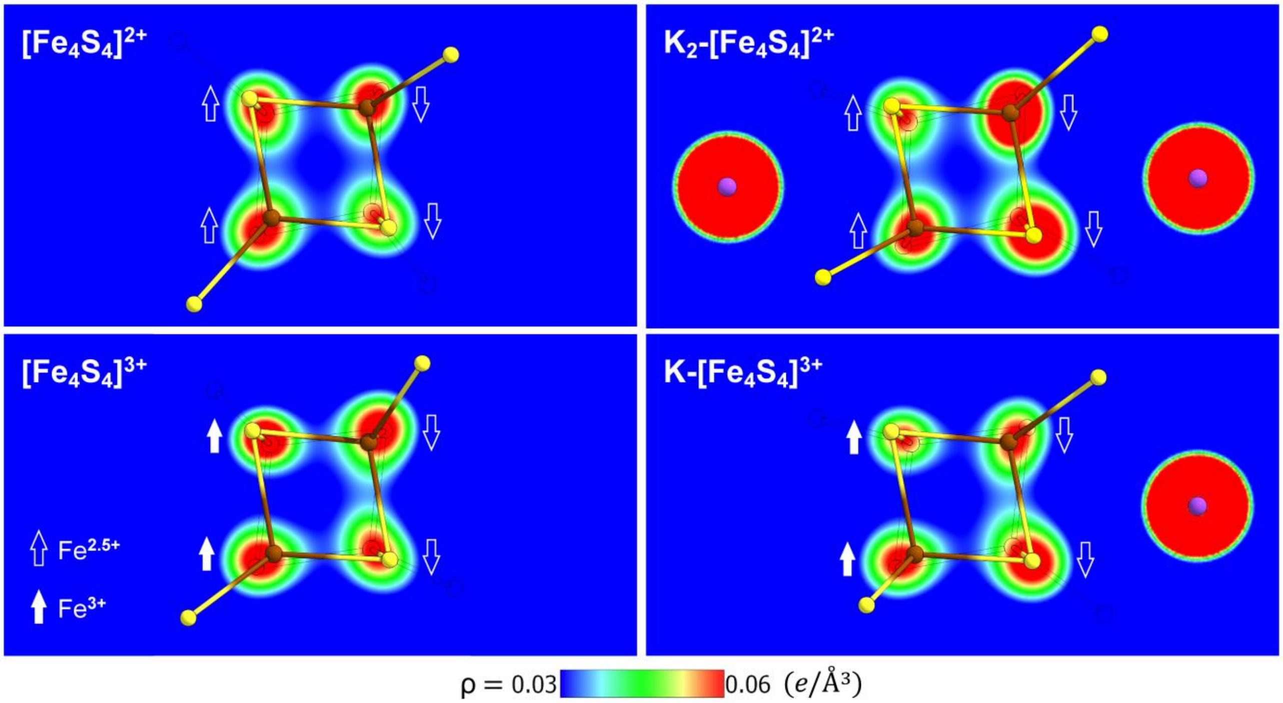


**Figure S63.** Total electron density in complexes **$K_x$-$[Fe_4S_4]^{2+}$** (top; $x = 0$ left / $x = 2$ right) and **$K_x$-$[Fe_4S_4]^{3+}$** (bottom; $x = 0$ left / $x = 1$ right) from the extended DFT modeling, mapped onto a plane dividing the cubane core in two halves. The plane contains a very approximate $C_2$ symmetry axis of the FeS core, passing through the $K^+$ ions when encapsulated. Only the Fe/S/K nuclei are shown for clarity, with the molecular fragment on the rear side of the plane displayed in thin lines. In all four panels, the two Fe sites characterized by excess $\alpha$-electron density occupy the left side of the cubane, cf. Figure S62 and Table S30. The spin-up/-down and mixed-valence ($Fe^{2.5+}$) / ferric ($Fe^{3+}$) sites are indicated using custom arrows. The color map (bottom) differentiates electron densities in the 0.03 (blue) < ρ < 0.06 (red) range ($e/Å^3$), where the contribution from Fe core electrons ($1s^2$–$3p^6$) is excluded. The mapped density areas at ρ ≈ 0.04 $e/Å^3$ (cyan) in between the Fe sites are indicative of Fe···Fe interactions.

**Table S30.** Relative energies (kJ $mol^{-1}$) of valence isomers in complexes **$K_x$-$[Fe_4S_4]^{2+}$** ($x$=0,2) and **$K_x$-$[Fe_4S_4]^{3+}$** ($x$=0,1) based on the extended DFT modeling. "BS*ab*" implies a broken-symmetry (BS) state with excess $\alpha$-electron density accumulated at the two Fe sites *a* and *b*, as designated in their crystallographic order and shown in Figure S62. The low-to-high energies are sorted top-to-bottom, with the ground-state BS isomer energy set to zero.

| **$[Fe_4S_4]^{2+}$** | | **$K_2$-$[Fe_4S_4]^{2+}$** | | **$[Fe_4S_4]^{3+}$** | | **$K$-$[Fe_4S_4]^{3+}$** | |
|---|---|---|---|---|---|---|---|
| BS13 (=BS24) | 0.0 | BS13 (=BS24) | 0.0 | BS34 | 0.0 | BS14 | 0.0 |
| BS14 (=BS23) | 5.2 | BS14 (=BS23) | 3.2 | BS12 | 1.6 | BS24 | 7.2 |
| BS12 (=BS34) | 7.4 | BS12 (=BS34) | 5.1 | BS13 | 7.4 | BS13 | 7.4 |
| | | | | BS23 | 11.8 | BS34 | 7.7 |
| | | | | BS14 | 16.9 | BS12 | 12.3 |
| | | | | BS24 | 27.1 | BS23 | 21.5 |

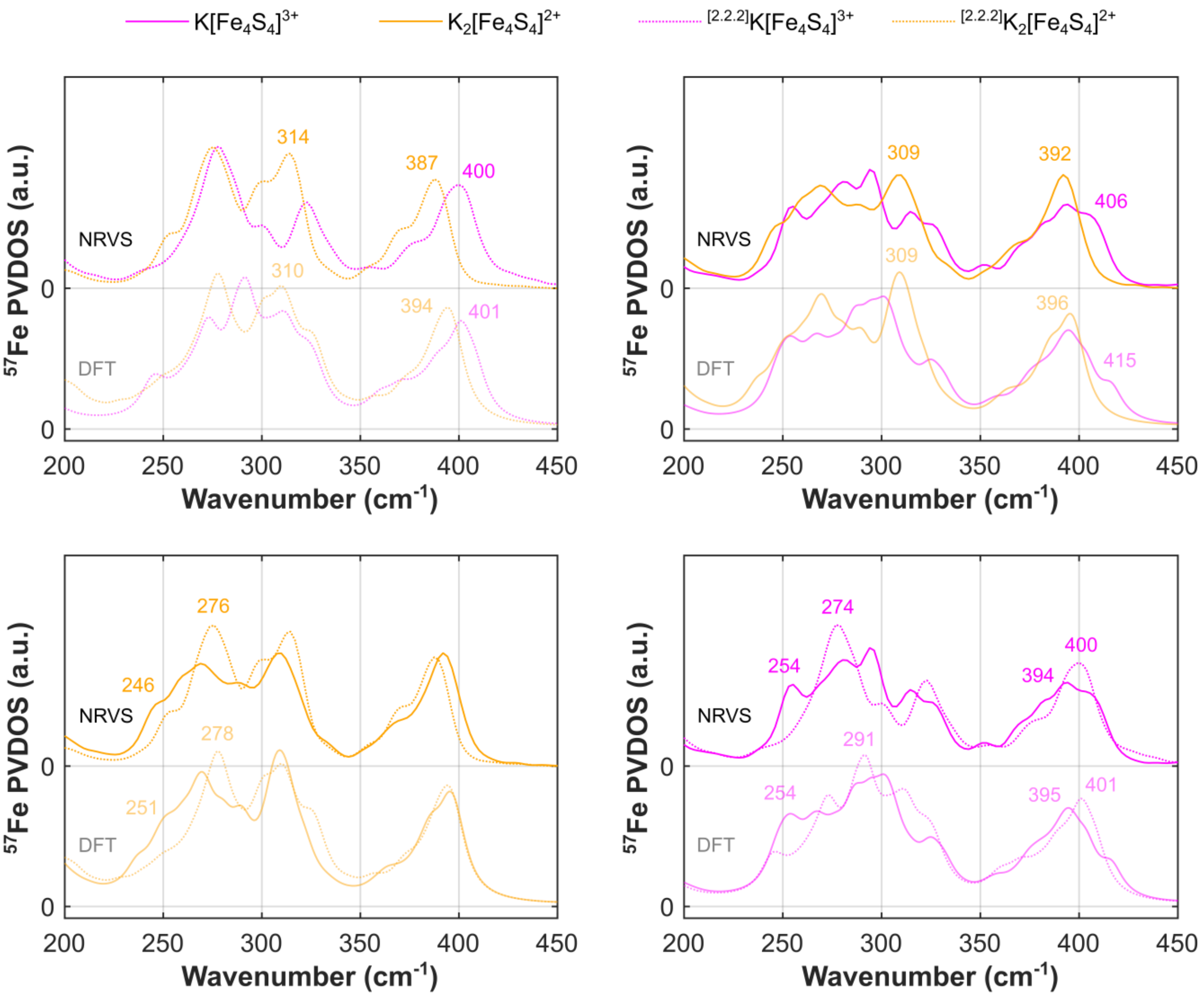


**Figure S64.** $^{57}$Fe-PVDOS spectra for the complexes **$^{[2.2.2]}K_2$-$[Fe_4S_4]^{2+}$/$^{[2.2.2]}$K-$[Fe_4S_4]^{3+}$** (*dotted lines*) and **$K_2$-$[Fe_4S_4]^{2+}$/K-$[Fe_4S_4]^{3+}$** (*solid lines*). The data from the set of 2×2 complexes is overlayed in 2×2 panels, displaying either (*top*; *left* and *right*) redox-dependent changes in the $K^+$-free and $K^+$-encapsulating variants, respectively, or (*bottom*) $K^+$ encapsulation dependent changes in panels on the left and right side, for the $[Fe_4S_4]^{2+}$/$[Fe_4S_4]^{3+}$ systems, correspondingly. Inside each panel, spectra from the NRVS experiment (*normal lines*, *top*) and extended DFT modeling (*fainted lines*, *bottom*) are shown. Selected band top or shoulder positions are labelled to highlight differences between the overlayed spectra.

## $^{57}$Fe PVDOS Spectra of Canonical *versus* Site-Differentiated Cubanes based on NRVS

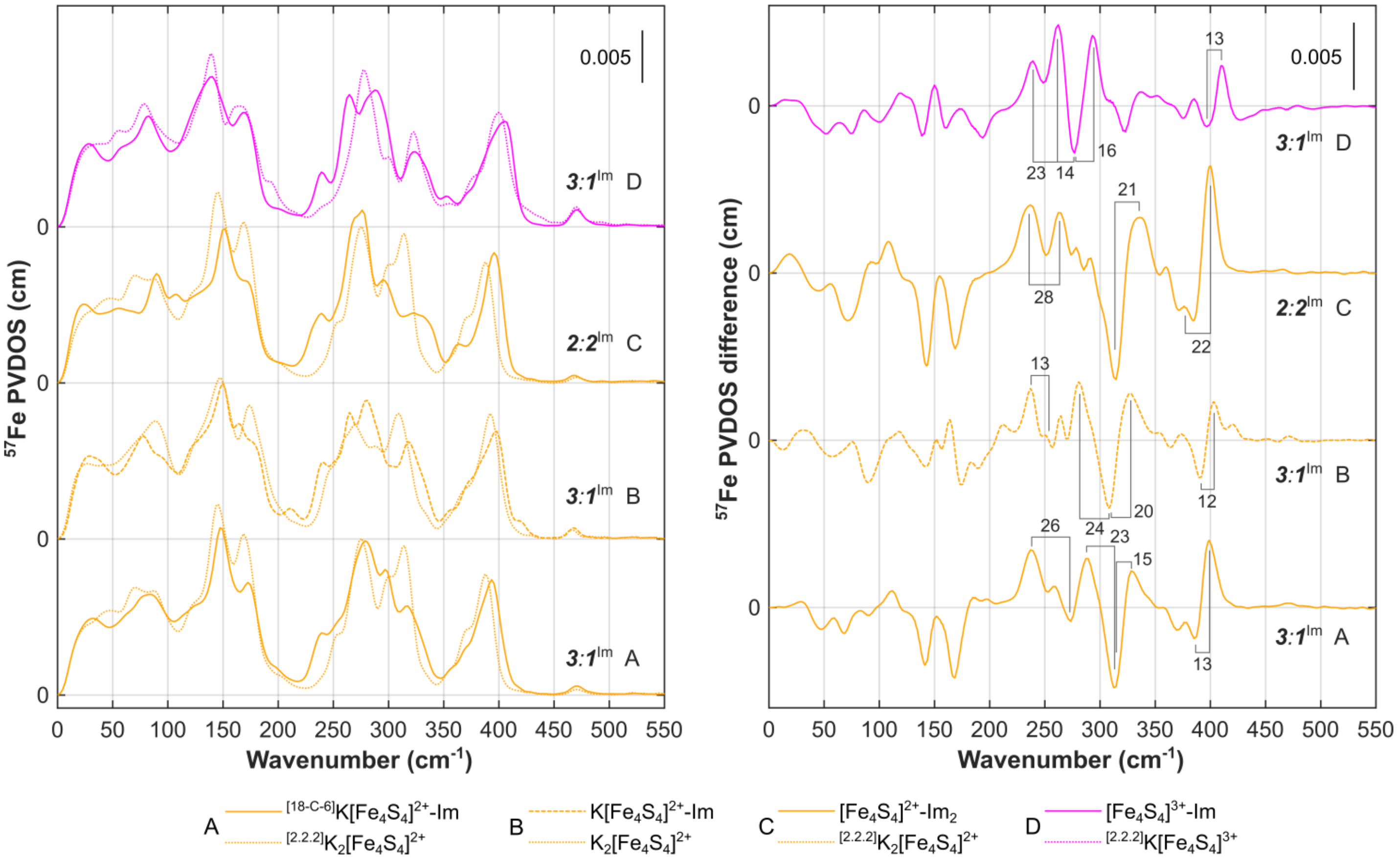


**Figure S65.** (*Left*) $^{57}$Fe NRVS PVDOS spectra of (A) **$^{(18\text{-}C\text{-}6)}$K-[$Fe_4S_4$]$^{2+}$-Im** and **$^{[2.2.2]}$K$_2$-[$Fe_4S_4$]$^{2+}$**, (B) **K-[$Fe_4S_4$]$^{2+}$-Im** and **K$_2$-[$Fe_4S_4$]$^{2+}$**, (C) **[$Fe_4S_4$]$^{2+}$-Im$_2$** and **$^{[2.2.2]}$K$_2$-[$Fe_4S_4$]$^{2+}$**, as well as, (D), **[$Fe_4S_4$]$^{3+}$-Im** and **$^{[2.2.2]}$K-[$Fe_4S_4$]$^{3+}$**. Spectra were recorded at 30-40 K on powdered samples. (*Right*) $^{57}$Fe NRVS PVDOS difference spectra upon site-substitution of the [$Fe_4S_4$]$^{2+}$ and [$Fe_4S_4$]$^{3+}$ cubanes according to the data as it is shown in the left panel. Selected energy differences have been annotated by *grey lines*.

## UV-vis Electronic Absorption Spectra of Canonical versus Site-Differentiated Cubanes

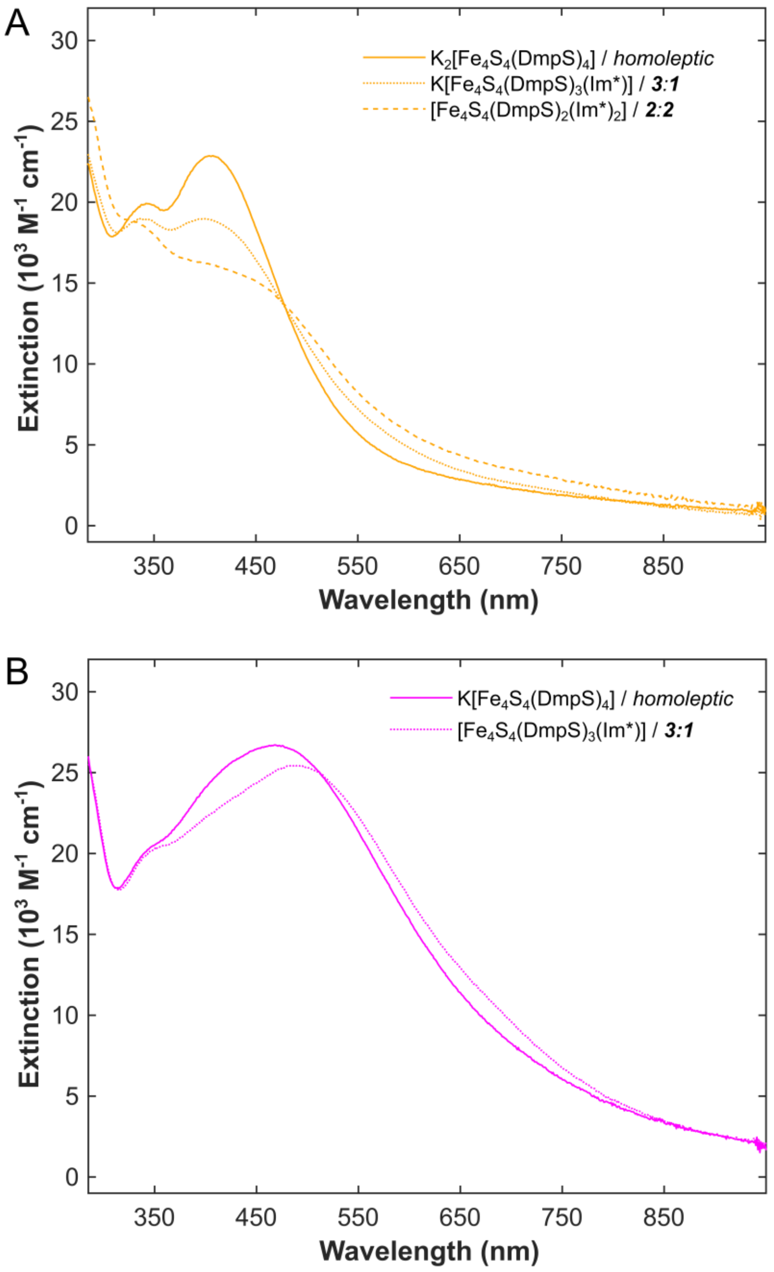


**Figure S66.** (A) UV-vis electronic absorption spectra of $K_2[Fe_4S_4(DmpS)_4]$ (*solid yellow line*), $K[Fe_4S_4(DmpS)_3(Im^*)]$ (*dotted yellow line*) and $[Fe_4S_4(DmpS)_3(Im^*)_2]$ (*dashed yellow line*) recorded in $1{\cdot}10^{-4}$ M toluene solution at room temperature, and, (B) of $K[Fe_4S_4(DmpS)_4]$ (*solid magenta line*) and $[Fe_4S_4(DmpS)_3(Im^*)]$ (*dotted magenta line*) likewise recorded in $1{\cdot}10^{-4}$ M toluene solution at room temperature. These spectra are reproduced from ref.[3].

## Adapted MatLab Scripts for Fitting the VT NMR Data

*Spin-ladder generation (taken from ref.[19])*

```
function [spin_states] = Fe4S4_spin_config(S_1,S_2,S_3,S_4,J,dJ_12,dJ_34,B,B12_on,B34_on)

%Input details:
%S_1: the spin of iron center 1
%S_2: the spin of iron center 2
%S_3: the spin of iron center 3
%S_4: the spin of iron center 4
%J: the global J value in cm-1 (see Hamiltonian in Inorg. Chem. 1988, 27, 3677.)
%dJ_12: the deviation in J coupling for the iron 1 - iron 2 pair in cm-1
%dJ_34: the deviation in J coupling for the iron 3 - iron 4 pair in cm-1
%B: double exchange term for the iron 1-iron 2 and/or the iron3-iron 4 pair in cm-1
%B12_on: boolean value; true if there is double exchange between iron 1 and
%iron 2, false otherwise
%B34_on: boolean value; true if there is double exchange between iron 3 and
%iron 4, false otherwise
%Output details:
%Columns 1-4 are S_1 - S_4, column 5 is S_12, column 6 is S_34, column 7 is
%S_tot, and column 8 is the energy of the configuration. Note that
%everything is sorted from lowest to highest energy, and has been
%normalized such that the minimum energy is zero.
%Energy levels from Inorg. Chem. 1988, 27, 3677.

S_12_range = [abs(S_1 - S_2):abs(S_1 + S_2)]; %define the range of spins for the 12 pair
S_34_range = [abs(S_3 - S_4):abs(S_3 + S_4)]; %define the range of spins for the 34 pair
spin_states = [];
for S_12 = S_12_range
    for S_34 = S_34_range
        for S_tot = abs(S_12 - S_34):(S_12 + S_34)
            if B12_on
                if B34_on
                    for pm1 = [1 -1]
                        for pm2 = [1 -1]
                        energy = ((J/2)*(S_tot*(S_tot+1))) + pm1*(B*(S_12 + 0.5)) + pm2*(B*(S_34 +
0.5)) + ((dJ_12/2)*(S_12*(S_12+1))) + ((dJ_34/2)*(S_34*(S_34+1)));
                        spin_states = [spin_states; S_1 S_2 S_3 S_4 S_12 S_34 S_tot energy];
                        end
                    end
                else
                    for pm = [1 -1]
                    energy = ((J/2)*(S_tot*(S_tot+1))) + pm*(B*(S_12 + 0.5)) +
((dJ_12/2)*(S_12*(S_12+1))) + ((dJ_34/2)*(S_34*(S_34+1)));
                    spin_states = [spin_states; S_1 S_2 S_3 S_4 S_12 S_34 S_tot energy];
                    end
                end
                elseif B34_on
                    for pm = [1 -1]
                    energy = ((J/2)*(S_tot*(S_tot+1))) + pm*(B*(S_34 + 0.5)) +
((dJ_12/2)*(S_12*(S_12+1))) + ((dJ_34/2)*(S_34*(S_34+1)));
                    spin_states = [spin_states; S_1 S_2 S_3 S_4 S_12 S_34 S_tot energy];
                    end
                else
```

```
                energy = ((J/2)*(S_tot*(S_tot+1))) + ((dJ_12/2)*(S_12*(S_12+1))) +
((dJ_34/2)*(S_34*(S_34+1)));
                spin_states = [spin_states; S_1 S_2 S_3 S_4 S_12 S_34 S_tot energy];
                end
            end
        end
    end
    min_energy = min(spin_states(:,8)); %calculate the minimum energy
    spin_states(:,8) = spin_states(:,8) - min_energy; %set the minimum energy to zero
    spin_states = sortrows(spin_states,8); %sort the output by energy
end
```

*Fit objective function for [$Fe_4S_4(DmpS)_3(Im^*)$] (adapted from ref.[19])*

```
function pshift_out = pShift_Fe4S4_3_to_1_fitadapted(x,T)
%Input details:
%x is a vector of parameters which determine the chemical shifts of
%nuclei coupled to a 3:1 site differentiated spin system with magnetic
%structure given by the "pair-of-pairs" Hamiltonian. T is a vector
%containing all the temperatures of interest.
%J: the global J value in cm-1 (see Hamiltonian in Inorg. Chem. 1988, 27, 3677.)
%dJ_12: the deviation in J coupling for the iron 1 - iron 2 pair in cm-1 (parameterized here by
the factor n)
%dJ_34: the deviation in J coupling for the iron 3 - iron 4 pair in cm-1 (parameterized here by
the factor n)
%B: double exchange term for the iron 1 - iron 2 and/or the iron 3 - iron 4 pair in cm-1
%T: temperature (in Kelvin)
%A: hyperfine coupling
%
%Output details:
%A matrix (pshift_out) with 7 columns and a number of rows equal to the length of the
%input temperature vector. The first column corresponds to the total
%chemical shifts of protons coupled to the three equivalent sites (the
%DmpS para/meta protons in this work, denoted here as H_A/H_B, respectively). The next three
%columns correspond to the chemical shifts of three chemically distinct protons
%bound to the unique site (denoted H_C, H_D, H_E and H_F). The final column contains
%the magnetic moment of the cluster multiplied by 10, in Bohr magnetons.
%Expressions for spin projection coefficients and NMR shifts have been taken
%from Inorg. Chem., 1991, 30, 4517.

J_1 = x(1); %J: the global J value for valence isomer A in cm-1
J_2 = x(2); %J: the global J value for valence isomer B in cm-1
B_1 = x(3); %The double exchange term for the mixed valence pair in cm-1

A_1_Fe3 = x(4); %The effective hyperfine coupling of H_A to a magnetically isolated Fe3+ ion
A_2_Fe3 = x(5); %The effective hyperfine coupling of H_B to a magnetically isolated Fe3+ ion
A_3_Fe3 = x(6); %The effective hyperfine coupling of H_C to a magnetically isolated Fe3+ ion
A_4_Fe3 = x(7); %The effective hyperfine coupling of H_D to a magnetically isolated Fe3+ ion
A_5_Fe3 = x(8); %The effective hyperfine coupling of H_E to a magnetically isolated Fe3+ ion
A_6_Fe3 = x(9); %The effective hyperfine coupling of H_F to a magnetically isolated Fe3+ ion

offset = x(10); %The offset energy
beta_3 = x(11); %A parameter controlling the extent of double exchange polarization. No
delocalization = 1; delocalization = 0.5

A_1_Fe2_5 = x(12); %The effective hyperfine coupling of H_A to a fictitious magnetically isolated
Fe2.5+ ion
A_2_Fe2_5 = x(13); %The effective hyperfine coupling of H_B to a fictitious magnetically isolated
Fe2.5+ ion
A_3_Fe2_5 = x(14); %The effective hyperfine coupling of H_C to a fictitious magnetically isolated
Fe2.5+ ion
A_4_Fe2_5 = x(15); %The effective hyperfine coupling of H_D to a fictitious magnetically isolated
Fe2.5+ ion
A_5_Fe2_5 = x(16); %The effective hyperfine coupling of H_E to a fictitious magnetically isolated
Fe2.5+ ion
A_6_Fe2_5 = x(17); %The effective hyperfine coupling of H_F to a fictitious magnetically isolated
Fe2.5+ ion
```

```
dia_1 = x(18); %The diamagnetic part of the total chemical shift for H_A
dia_2 = x(19); %The diamagnetic part of the total chemical shift for H_B
dia_3 = x(20); %The diamagnetic part of the total chemical shift for H_C
dia_4 = x(21); %The diamagnetic part of the total chemical shift for H_D
dia_5 = x(22); %The diamagnetic part of the total chemical shift for H_E
dia_6 = x(23); %The diamagnetic part of the total chemical shift for H_F

frac_1 = x(24); %The ratio of delta J to J in valence isomer A
frac_2 = x(24); %The ratio of delta J to J in valence isomer B (taken as equal to that of valence
isomer A)
S_1 = 2.5; %The local spin state of iron site 1 (Fe3+)
S_2 = 2.5; %The local spin state of iron site 2 (Fe3+)
S_3 = 2.5; %The local spin state of iron site 3 (Fe3+)
S_4 = 2; %The local spin state of iron site 4 (Fe2+)

%Generate the spin spectrum for the first valence isomer family
spin_spectrum = Fe4S4_spin_config(S_1,S_2,S_3,S_4,J_1,J_1*frac_1,-J_1*frac_1,B_1,false,true);

%Generate the spin spectrum for the second valence isomer family
spin_spectrum_3 = Fe4S4_spin_config(S_3,S_4,S_1,S_2,J_2,-J_2*frac_2,J_2*frac_2,B_1,true,false);

%Offset the energy of the second valence isomer's spin ladder
spin_spectrum_3(:,8) = spin_spectrum_3(:,8) + 1*offset;

%Merge the spin ladders
spin_spectrum_total = [spin_spectrum;spin_spectrum_3];
g_e = -2.00231930436256; %electron g-value
mu_b = 9.274009994E-24; %Bohr magneton (J/T)
h_bar = 1.054571817E-34; %reduced Planck constant (J*S)
gamma_I = 267.5E6; %nuclear larmor frequency (Hz/T; 267.5E6 for 1H)
k_B = 1.38064852E-23; %boltzmann constant (J/K)
prefactor = -(1./T)*(2*pi*g_e*mu_b)/(gamma_I*3*k_B); %Compute the prefactor which appears in the
expression for paramagnetic NMR shift
beta_1 = 0.5; %Fix double exchange between chemically equivalent sites to be symmetric
beta_2 = 1 - beta_1; %Constants used to control double exchange
beta_4 = 1 - beta_3; %Constants used to control double exchange

%Initialize matrices which will be used to compute chemical shifts for
%individual spin ladders.
numerator_1 = zeros(length(T),4);
denominator_1 = zeros(length(T),1);
numerator_2 = zeros(length(T),4);
denominator_2 = zeros(length(T),1);

%Initialize an output vector
pshift = zeros(length(T),7);

for x = 1:length(spin_spectrum) %Loop over the set of states for the first valence isomer
S_1 = spin_spectrum(x,1); %Read in the spin of metal center 1 in the x-th state
S_2 = spin_spectrum(x,2); %Read in the spin of metal center 2 in the x-th state
S_3 = spin_spectrum(x,3); %Read in the spin of metal center 3 in the x-th state
S_4 = spin_spectrum(x,4); %Read in the spin of metal center 4 in the x-th state
S_12 = spin_spectrum(x,5); %Read in the spin of the Fe1-Fe2 pair in the x-th state
S_34 = spin_spectrum(x,6); %Read in the spin of the Fe3-Fe4 pair in the x-th state
S_tot = spin_spectrum(x,7); %Read in the total spin of the x-th state
```

```
energy = spin_spectrum(x,8)/5.03445E22; %Read in the relative energy of the x-th state and convert
it from cm-1 to J
denominator_1 = denominator_1 + (2*S_tot+1)*exp(-energy./(k_B.*T)); %Add a multiplicity- weighted
term to the denominator for the first spin ladder (Boltzmann sum)

%Generate the spin-projection factors for metal center 1
alpha_1 = 0.5*((beta_1*((S_12*(S_12 + 1)) + (S_1*(S_1 + 1)) - (S_2*(S_2 + 1)))) +
(beta_2*((S_12*(S_12 + 1)) - (S_1*(S_1 + 1)) + (S_2*(S_2 + 1)))));
gamma_1 = 0.5*((S_tot*(S_tot + 1)) + (S_12*(S_12 + 1)) - (S_34*(S_34 + 1)));
delta_1 = (S_12*(S_12 + 1))*(S_tot*(S_tot + 1));
C_i1 = alpha_1*gamma_1/delta_1;
if isnan(C_i1)
C_i1 = 0;
end
numerator_1(:,1) = numerator_1(:,1) + (1E6*(S_tot*(S_tot + 1))*(2*S_tot+1)*exp(-
energy./(k_B.*T)))*C_i1; %Add a multiplicity and spin projection coefficient weighted Boltzmann
term to the numerator associated with metal site 1

%Generate the spin-projection factors for metal center 2
alpha_2 = 0.5*((beta_1*((S_12*(S_12 + 1)) + (S_2*(S_2 + 1)) - (S_1*(S_1 + 1)))) +
(beta_2*((S_12*(S_12 + 1)) - (S_2*(S_2 + 1)) + (S_1*(S_1 + 1)))));
gamma_2 = 0.5*((S_tot*(S_tot + 1)) + (S_12*(S_12 + 1)) - (S_34*(S_34 + 1)));
delta_2 = (S_12*(S_12 + 1))*(S_tot*(S_tot + 1));
C_i2 = alpha_2*gamma_2/delta_2;
if isnan(C_i2)
C_i2 = 0;
end
numerator_1(:,2) = numerator_1(:,2) + (1E6*(S_tot*(S_tot + 1))*(2*S_tot+1)*exp(-
energy./(k_B.*T)))*C_i2; %Add a multiplicity and spin projection coefficient weighted Boltzmann
term to the numerator associated with metal site 2

%Generate the spin-projection factors for metal center 3
alpha_3 = 0.5*((beta_3*((S_34*(S_34 + 1)) + (S_3*(S_3 + 1)) - (S_4*(S_4 + 1)))) +
(beta_4*((S_34*(S_34 + 1)) - (S_3*(S_3 + 1)) + (S_4*(S_4 + 1)))));
gamma_3 = 0.5*((S_tot*(S_tot + 1)) + (S_34*(S_34 + 1)) - (S_12*(S_12 + 1)));
delta_3 = (S_34*(S_34 + 1))*(S_tot*(S_tot + 1));
C_i3 = alpha_3*gamma_3/delta_3;
if isnan(C_i3)
C_i3 = 0;
end
numerator_1(:,3) = numerator_1(:,3) + ((1E6*(S_tot*(S_tot + 1))*(2*S_tot+1))*exp(-
energy./(k_B.*T)))*C_i3; %Add a multiplicity and spin projection coefficient weighted Boltzmann
term to the numerator associated with metal site 3

%Generate the spin-projection factors for metal center 4
alpha_4 = 0.5*((beta_3*((S_34*(S_34 + 1)) + (S_4*(S_4 + 1)) - (S_3*(S_3 + 1)))) +
(beta_4*((S_34*(S_34 + 1)) - (S_4*(S_4 + 1)) + (S_3*(S_3 + 1)))));
gamma_4 = 0.5*((S_tot*(S_tot + 1)) + (S_34*(S_34 + 1)) - (S_12*(S_12 + 1)));
delta_4 = (S_34*(S_34 + 1))*(S_tot*(S_tot + 1));
C_i4 = alpha_4*gamma_4/delta_4;
if isnan(C_i4)
C_i4 = 0;
end
numerator_1(:,4) = numerator_1(:,4) + (1E6*(S_tot*(S_tot + 1))*(2*S_tot+1)*exp(-
energy./(k_B.*T)))*C_i4; %Add a multiplicity and spin projection coefficient weighted Boltzmann
term to the numerator associated with metal site 4
```

```
end

for x = 1:length(spin_spectrum_3) %loop over the states associated with the second family of
valence isomers
S_1 = spin_spectrum_3(x,1); %Read in the spin of metal center 1 in the x-th state
S_2 = spin_spectrum_3(x,2); %Read in the spin of metal center 2 in the x-th state
S_3 = spin_spectrum_3(x,3); %Read in the spin of metal center 3 in the x-th state
S_4 = spin_spectrum_3(x,4); %Read in the spin of metal center 4 in the x-th state
S_12 = spin_spectrum_3(x,5); %Read in the spin of the Fe1-Fe2 pair in the x-th state
S_34 = spin_spectrum_3(x,6); %Read in the spin of the Fe3-Fe4 pair in the x-th state
S_tot = spin_spectrum_3(x,7); %Read in the total spin of the x-th state
energy = spin_spectrum_3(x,8)/5.03445E22; %Read in the relative energy of the x-th state and
convert it from cm-1 to J
denominator_2 = denominator_2 + (2*S_tot+1)*exp(-energy./(k_B.*T)); %Add a multiplicity-weighted
term to the denominator for the second spin ladder (Boltzmann sum)

%Generate the spin-projection factors for metal center 1
alpha_1 = 0.5*((beta_1*((S_12*(S_12 + 1)) + (S_1*(S_1 + 1)) - (S_2*(S_2 + 1)))) +
(beta_2*((S_12*(S_12 + 1)) - (S_1*(S_1 + 1)) + (S_2*(S_2 + 1)))));
gamma_1 = 0.5*((S_tot*(S_tot + 1)) + (S_12*(S_12 + 1)) - (S_34*(S_34 + 1)));
delta_1 = (S_12*(S_12 + 1))*(S_tot*(S_tot + 1));
C_i1 = alpha_1*gamma_1/delta_1;
if isnan(C_i1)
C_i1 = 0;
end
numerator_2(:,1) = numerator_2(:,1) + (1E6*(S_tot*(S_tot + 1))*(2*S_tot+1)*exp(-
energy./(k_B.*T)))*C_i1; %Add a multiplicity and spin projection coefficient weighted Boltzmann
term to the numerator associated with metal site 1

%Generate the spin-projection factors for metal center 2
alpha_2 = 0.5*((beta_1*((S_12*(S_12 + 1)) + (S_2*(S_2 + 1)) - (S_1*(S_1 + 1)))) +
(beta_2*((S_12*(S_12 + 1)) - (S_2*(S_2 + 1)) + (S_1*(S_1 + 1)))));
gamma_2 = 0.5*((S_tot*(S_tot + 1)) + (S_12*(S_12 + 1)) - (S_34*(S_34 + 1)));
delta_2 = (S_12*(S_12 + 1))*(S_tot*(S_tot + 1));
C_i2 = alpha_2*gamma_2/delta_2;
if isnan(C_i2)
C_i2 = 0;
end

numerator_2(:,2) = numerator_2(:,2) + (1E6*(S_tot*(S_tot + 1))*(2*S_tot+1)*exp(-
energy./(k_B.*T)))*C_i2; %Add a multiplicity and spin projection coefficient weighted Boltzmann
term to the numerator associated with metal site 2
%Generate the spin-projection factors for metal center 3
alpha_3 = 0.5*((beta_3*((S_34*(S_34 + 1)) + (S_3*(S_3 + 1)) - (S_4*(S_4 + 1)))) +
(beta_4*((S_34*(S_34 + 1)) - (S_3*(S_3 + 1)) + (S_4*(S_4 + 1)))));
gamma_3 = 0.5*((S_tot*(S_tot + 1)) + (S_34*(S_34 + 1)) - (S_12*(S_12 + 1)));
delta_3 = (S_34*(S_34 + 1))*(S_tot*(S_tot + 1));
C_i3 = alpha_3*gamma_3/delta_3;
if isnan(C_i3)
C_i3 = 0;
end
numerator_2(:,3) = numerator_2(:,3) + ((1E6*(S_tot*(S_tot + 1))*(2*S_tot+1))*exp(-
energy./(k_B.*T)))*C_i3; %Add a multiplicity and spin projection coefficient weighted Boltzmann
term to the numerator associated with metal site 3

%Generate the spin-projection factors for metal center 4
```

```
alpha_4 = 0.5*((beta_3*((S_34*(S_34 + 1)) + (S_4*(S_4 + 1)) - (S_3*(S_3 + 1)))) +
(beta_4*((S_34*(S_34 + 1)) - (S_4*(S_4 + 1)) + (S_3*(S_3 + 1)))));
gamma_4 = 0.5*((S_tot*(S_tot + 1)) + (S_34*(S_34 + 1)) - (S_12*(S_12 + 1)));
delta_4 = (S_34*(S_34 + 1))*(S_tot*(S_tot + 1));
C_i4 = alpha_4*gamma_4/delta_4;
if isnan(C_i4)
C_i4 = 0;
end
numerator_2(:,4) = numerator_2(:,4) + (1E6*(S_tot*(S_tot + 1))*(2*S_tot+1)*exp(-
energy./(k_B.*T)))*C_i4; %Add a multiplicity and spin projection coefficient weighted Boltzmann
term to the numerator associated with metal site 4
end

pshift(:,1) = pshift(:,1) + 1E6.*prefactor.*(A_1_Fe3*numerator_1(:,1) +
A_1_Fe2_5*numerator_2(:,1))./(denominator_1+denominator_2); %Generate the total paramagnetic part
of the chemical shift (in ppm) the nucleus coupled to metal site 1, considering all spin states
pshift(:,2) = pshift(:,2) + 1E6.*prefactor.*(A_1_Fe3*numerator_1(:,2) +
A_1_Fe2_5*numerator_2(:,2))./(denominator_1+denominator_2); %Generate the total paramagnetic part
of the chemical shift (in ppm) the nucleus coupled to metal site 2, considering all spin states
pshift(:,3) = pshift(:,3) + 1E6.*prefactor.*(A_1_Fe2_5*numerator_1(:,3) +
A_1_Fe3*numerator_2(:,3))./(denominator_1+denominator_2); %Generate the total paramagnetic part of
the chemical shift (in ppm) the nucleus coupled to metal site 3, considering all spin states
averageshift1 = (1/3).*(pshift(:,1) + pshift(:,2) + pshift(:,3)); %Average the shifts of the three
chemically equivalent sites to give a single chemical shift for H_A

pshift(:,4) = pshift(:,4) + 1E6.*prefactor.*(A_2_Fe3*numerator_1(:,1) +
A_2_Fe2_5*numerator_2(:,1))./(denominator_1+denominator_2); %Generate the total paramagnetic part
of the chemical shift (in ppm) the nucleus coupled to metal site 1, considering all spin states
pshift(:,5) = pshift(:,5) + 1E6.*prefactor.*(A_2_Fe3*numerator_1(:,2) +
A_2_Fe2_5*numerator_2(:,2))./(denominator_1+denominator_2); %Generate the total paramagnetic part
of the chemical shift (in ppm) the nucleus coupled to metal site 2, considering all spin states
pshift(:,6) = pshift(:,6) + 1E6.*prefactor.*(A_2_Fe2_5*numerator_1(:,3) +
A_2_Fe3*numerator_2(:,3))./(denominator_1+denominator_2); %Generate the total paramagnetic part of
the chemical shift (in ppm) the nucleus coupled to metal site 3, considering all spin states
averageshift2 = (1/3).*(pshift(:,4) + pshift(:,5) + pshift(:,6)); %Average the shifts of the three
chemically equivalent sites to give a single chemical shift for H_B

pshift_out(:,1) = averageshift1 + dia_1; %Generate the total chemical shift for the three
chemically equivalent sites by adding the relevant diamagnetic contribution
pshift_out(:,2) = averageshift2 + dia_2; %Generate the total chemical shift for the three
chemically equivalent sites by adding the relevant diamagnetic contribution
pshift_out(:,3) = pshift(:,7) + 1E6.*prefactor.*(A_3_Fe2_5*numerator_1(:,4) +
A_3_Fe3*numerator_2(:,4))./(denominator_1+denominator_2) + dia_3; %Generate the total paramagnetic
part of the chemical shift (in ppm) one of the nuclei coupled to metal site 4, considering all
spin states, and add a diamagnetic contribution to give the shift of H_C
pshift_out(:,4) = pshift(:,7) + 1E6.*prefactor.*(A_4_Fe2_5*numerator_1(:,4) +
A_4_Fe3*numerator_2(:,4))./(denominator_1+denominator_2) + dia_4; %Generate the total paramagnetic
part of the chemical shift (in ppm) one of the nuclei coupled to metal site 4, considering all
spin states, and add a diamagnetic contribution to give the shift of H_D
pshift_out(:,5) = pshift(:,7) + 1E6.*prefactor.*(A_5_Fe2_5*numerator_1(:,4) +
A_5_Fe3*numerator_2(:,4))./(denominator_1+denominator_2) + dia_5; %Generate the total paramagnetic
part of the chemical shift (in ppm) one of the nuclei coupled to metal site 4, considering all
spin states, and add a diamagnetic contribution to give the shift of H_E
pshift_out(:,6) = pshift(:,7) + 1E6.*prefactor.*(A_6_Fe2_5*numerator_1(:,4) +
A_6_Fe3*numerator_2(:,4))./(denominator_1+denominator_2) + dia_6; %Generate the total paramagnetic
```

```
part of the chemical shift (in ppm) one of the nuclei coupled to metal site 4, considering all
spin states, and add a diamagnetic contribution to give the shift of H_F

%Initialize the numerator and denominator values for a magnetic moment
%calculation
num = zeros(length(T),1);
denom = zeros(length(T),1);
for n = 1:length(spin_spectrum_total)
num = num +
((2*spin_spectrum_total(n,7)+1)).*((((spin_spectrum_total(n,7).*(spin_spectrum_total(n,7)+1)))).*e
xp(-spin_spectrum_total(n,8)./(0.695.*T)));
denom = denom + ((2*spin_spectrum_total(n,7)+1)).*(exp(-spin_spectrum_total(n,8)./(0.695.*T)));
end
pred_mag_moment = abs(g_e)*((num./denom)).^0.5; %Compute the magnetic moment as a function of
temperature.
pshift_out(:,7) = 10*pred_mag_moment; %Assign the 7th and final column of the output matrix as 10
x the computed magnetic moments, in Bohr magnetons.
end
```

*__Fitting script for [$Fe_4S_4$(DmpS)$_3$(Im*)] (adapted from ref.[19])__*

```
clear all

%Fitting script, for [Fe4S4(DmpS)3(Im*)]
%Set the range of J values for the first family of valence isomers
J_1_min = 100;
J_1_max = 400;
J_1_initial = 200;

%Set the range of J values for the second family of valence isomers
J_2_min = 200;
J_2_max = 900;
J_2_initial = 600;

%Set the range of B values for all valence isomers
B_1_min = 50;
B_1_max = 1000;
B_1_initial = 300;

%Set the range of values for effective hyperfine coupling between the DmpS
%para backbone protons and an Fe3+ ion (negative!)
Fe3_hyperfine_pDmpS_backbone_min = -3;
Fe3_hyperfine_pDmpS_backbone_max = 0;
Fe3_hyperfine_pDmpS_backbone_initial = -2;

%Set the range of values for effective hyperfine coupling between the DmpS
%meta backbone protons and an Fe3+ ion (positive!)
Fe3_hyperfine_mDmpS_backbone_min = 0;
Fe3_hyperfine_mDmpS_backbone_max = 3;
Fe3_hyperfine_mDmpS_backbone_initial = 1.5;

%Set the range of values for effective hyperfine coupling between the Im*
%1 protons and an Fe3+ ion, restricting the values to be positive
Fe3_hyperfine_Im1_min = 0;
Fe3_hyperfine_Im1_max = 0.5;
Fe3_hyperfine_Im1_initial = 0.3;

%Set the range of values for effective hyperfine coupling between the Im*
%1 protons and an Fe3+ ion, restricting the values to be positive
Fe3_hyperfine_Im2_min = 0;
Fe3_hyperfine_Im2_max = 0.5;
Fe3_hyperfine_Im2_initial = 0.3;

%Set the range of values for effective hyperfine coupling between the Im*
%3 protons and an Fe3+ ion, restricting the values to be positive
Fe3_hyperfine_Im3_min = 0;
Fe3_hyperfine_Im3_max = 0.3;
Fe3_hyperfine_Im3_initial = 0.13;

%Set the range of values for effective hyperfine coupling between the Im*
%4 proton and an Fe3+ ion, restricting the values to be negative
Fe3_hyperfine_Im4_min = -0.1;
Fe3_hyperfine_Im4_max = 0;
Fe3_hyperfine_Im4_initial = -0.01;
```

```
%-------------------------------------

%Set the range of values for effective hyperfine coupling between the DmpS
%para backbone protons and an Fe2.5+ ion
Fe2_5_hyperfine_pDmpS_backbone_min = Fe3_hyperfine_pDmpS_backbone_min;
Fe2_5_hyperfine_pDmpS_backbone_max = Fe3_hyperfine_pDmpS_backbone_max;
Fe2_5_hyperfine_pDmpS_backbone_initial = Fe3_hyperfine_pDmpS_backbone_initial;

%Set the range of values for effective hyperfine coupling between the DmpS
%meta backbone protons and an Fe2.5+ ion
Fe2_5_hyperfine_mDmpS_backbone_min = Fe3_hyperfine_mDmpS_backbone_min;
Fe2_5_hyperfine_mDmpS_backbone_max = Fe3_hyperfine_mDmpS_backbone_max;
Fe2_5_hyperfine_mDmpS_backbone_initial = Fe3_hyperfine_mDmpS_backbone_initial;

%Set the range of values for effective hyperfine coupling between the Im*
%1 protons and an Fe2.5+ ion, restricting the values to be inverse
Fe2_5_hyperfine_Im1_min = -Fe3_hyperfine_Im1_max;
Fe2_5_hyperfine_Im1_max = -Fe3_hyperfine_Im1_min;
Fe2_5_hyperfine_Im1_initial = -Fe3_hyperfine_Im1_initial;

%Set the range of values for effective hyperfine coupling between the Im*
%2 protons and an Fe2.5+ ion, restricting the values to be inverse
Fe2_5_hyperfine_Im2_min = -Fe3_hyperfine_Im2_max;
Fe2_5_hyperfine_Im2_max = -Fe3_hyperfine_Im2_min;
Fe2_5_hyperfine_Im2_initial = -Fe3_hyperfine_Im2_initial;

%Set the range of values for effective hyperfine coupling between the Im*
%3 protons and an Fe2.5+ ion, restricting the values to be inverse
Fe2_5_hyperfine_Im3_min = -Fe3_hyperfine_Im3_max;
Fe2_5_hyperfine_Im3_max = -Fe3_hyperfine_Im3_min;
Fe2_5_hyperfine_Im3_initial = -Fe3_hyperfine_Im3_initial;

%Set the range of values for effective hyperfine coupling between the Im*
%4 proton and an Fe2.5+ ion, restricting the values to be inverse
Fe2_5_hyperfine_Im4_min = -Fe3_hyperfine_Im4_max;
Fe2_5_hyperfine_Im4_max = -Fe3_hyperfine_Im4_min;
Fe2_5_hyperfine_Im4_initial = -Fe3_hyperfine_Im4_initial;

%Set the initial range for the offset energy
offset_min = -500;
offset_max = 500;
offset_initial = 0;

%Enforce symmetric double exchange
beta = 0.5;

%Set the diamagnetic shift values
pDmpS_dia = 7.24;
mDmpS_dia = 7.03;
Im1_dia = 2.21;
Im2_dia = 2.00;
Im3_dia = 1.71;
Im4_dia = 2.54;

tic; %time the fit routine
iter = 1; %initialize a variable to count the number of iterations
```

```
fits = []; %initialize a matrix to hold all parameters
macro_iter = 20; %Set the number of random diamagnetic shift values
micro_iter = 50; %Set the number of initial fit guesses
clf;
rng('default'); %set the random number generator (for reproducibility)
rng(1);
residuals = []; %initialize a matrix to hold fit RMSE values

%Input raw chemical shift data

%input the total chemical shifts for the Im* 1 resonances as a function
%of temperature
H_1 = [13.13; 13.57; 14.15; 14.70; 15.19; 15.69; 16.56; 17.12; 17.63];

%input the total chemical shifts for the Im* 3 resonance as a function
%of temperature
H_2 = [9.00; 9.31; 9.71; 10.10; 10.46; 10.83; 11.46; 11.91; 12.28];

%input the total chemical shifts for the Im* 3 resonance as a function
%of temperature
H_3 = [5.50; 5.71; 5.98; 6.25; 6.49; 6.74; 7.08; 7.43; 7.66];

%input the total chemical shifts for the Im* 4 resonance as a function
%of temperature
H_4 = [1.78; 1.75; 1.72; 1.70; 1.68; 1.66; 1.63; 1.61; 1.60];

%input the total chemical shifts for the DmpS meta resonance as a function
%of temperature
L_m = [8.345; 8.155; 7.91; 7.675; 7.46; 7.24; 7.10; 6.97; 6.84];

%input the total chemical shifts for the DmpS para resonance as a function
%of temperature
L_p = [5.97; 6.12; 6.32; 6.50; 6.66; 6.74; 6.83; 6.92; 6.99];

%input the magnetometry data
mag_data = [2.9669;2.9351;2.8999;2.8738;2.8399;2.7914;2.7537;2.7092;2.6794];

for n = 1:macro_iter %Loop for the desired number of random diamagnetic shift values
disp(['Iteration ' num2str(iter) ' of ' num2str(macro_iter)]); %display the current iteration
tempdata = [299; 288.5; 278; 268; 258; 248; 237.8; 227.9; 220.2]; %input the absolute temperature
(in K)

%read in the total chemical shifts for the Im* 1 resonances as a function
%of temperature, and add a normally distributed error with variance +/-0.1 ppm
H_1_work = 0.1*randn(1,1) + H_1;

%read in the total chemical shifts for the Im* 2 resonances as a function
%of temperature, and add a normally distributed error with variance +/-0.1 ppm
H_2_work = 0.1*randn(1,1) + H_2;

%read in the total chemical shifts for the Im* 3 resonance as a function
%of temperature, and add a normally distributed error with variance +/-0.1 ppm
H_3_work = 0.1*randn(1,1) + H_3;

%read in the total chemical shifts for the Im* 4 resonance as a function
%of temperature, and add a normally distributed error with variance +/-0.1 ppm
```

```
H_4_work = 0.1*randn(1,1) + H_4;

%read in the total chemical shifts for the Dmps meta resonance as a function
%of temperature, and add a normally distributed error with variance +/-0.1 ppm
L_m_work = 0.1*randn(1,1) + L_m;

%read in the total chemical shifts for the DmpS para resonance as a function
%of temperature, and add a normally distributed error with variance +/-0.1 ppm
L_p_work = 0.1*randn(1,1) + L_p;

%read the magnetometry data and scale it by a factor of 10
mag_data_work = 10*mag_data;

%Collect all of the chemical shift/magnetometry data into a single
%matrix
ydata = zeros(length(L_p_work),6);
ydata(:,7) = mag_data_work;
ydata(:,6) = H_4_work;
ydata(:,5) = H_3_work;
ydata(:,4) = H_2_work;
ydata(:,3) = H_1_work;
ydata(:,2) = L_m_work;
ydata(:,1) = L_p_work;
normalization_factors = 1./sqrt(abs(ydata(1,1:6)-ydata(end,1:6))); %Compute the weighting factors
for the NMR data
ydata(:,1:6) = ydata(:,1:6).*normalization_factors; %Scale the NMR data by the weighting factors

%Enter values for the initial parameters
x0 = [J_1_initial J_2_initial B_1_initial Fe3_hyperfine_pDmpS_backbone_initial
Fe3_hyperfine_mDmpS_backbone_initial Fe3_hyperfine_Im1_initial Fe3_hyperfine_Im2_initial
Fe3_hyperfine_Im3_initial Fe3_hyperfine_Im4_initial offset_initial beta
Fe2_5_hyperfine_pDmpS_backbone_initial Fe2_5_hyperfine_mDmpS_backbone_initial
Fe2_5_hyperfine_Im1_initial Fe2_5_hyperfine_Im2_initial Fe2_5_hyperfine_Im3_initial
Fe2_5_hyperfine_Im4_initial pDmpS_dia mDmpS_dia Im1_dia Im2_dia Im3_dia Im4_dia 0];

%Enter the lower bounds for all parameters
lb = [J_1_min J_2_min B_1_min Fe3_hyperfine_pDmpS_backbone_min Fe3_hyperfine_mDmpS_backbone_min
Fe3_hyperfine_Im1_min Fe3_hyperfine_Im2_min Fe3_hyperfine_Im3_min Fe3_hyperfine_Im4_min offset_min
beta Fe2_5_hyperfine_pDmpS_backbone_min Fe2_5_hyperfine_mDmpS_backbone_min Fe2_5_hyperfine_Im1_min
Fe2_5_hyperfine_Im2_min Fe2_5_hyperfine_Im3_min Fe2_5_hyperfine_Im4_min pDmpS_dia mDmpS_dia
Im1_dia Im2_dia Im3_dia Im4_dia -1];

%Enter the upper bounds for all parameters
ub = [J_1_max J_2_max B_1_max Fe3_hyperfine_pDmpS_backbone_max Fe3_hyperfine_mDmpS_backbone_max
Fe3_hyperfine_Im1_max Fe3_hyperfine_Im2_max Fe3_hyperfine_Im3_max Fe3_hyperfine_Im4_max offset_max
beta Fe2_5_hyperfine_pDmpS_backbone_max Fe2_5_hyperfine_mDmpS_backbone_max Fe2_5_hyperfine_Im1_max
Fe2_5_hyperfine_Im2_max Fe2_5_hyperfine_Im3_max Fe2_5_hyperfine_Im4_max pDmpS_dia mDmpS_dia
Im1_dia Im2_dia Im3_dia Im4_dia 1];

%Scale the hyperfine parameters by the appropriate values (to be
%renormalized later)
x0(4:9) = x0(4:9).*normalization_factors;
x0(12:17) = x0(12:17).*normalization_factors;
x0(18:23) = x0(18:23).*normalization_factors;
lb(4:9) = lb(4:9).*normalization_factors;
lb(12:17) = lb(12:17).*normalization_factors;
```

```
lb(18:23) = lb(18:23).*normalization_factors;
ub(4:9) = ub(4:9).*normalization_factors;
ub(12:17) = ub(12:17).*normalization_factors;
ub(18:23) = ub(18:23).*normalization_factors;

%Set a temperature range to plot the fits over
temprange = (200:310)*1;

%Set the least squares options
opts = optimoptions('lsqcurvefit','MaxIterations',40000,'OptimalityTolerance',1e-
20,'MaxFunctionEvaluations',40000,'FunctionTolerance',1e-100,'StepTolerance',1e-10);

%Set the objective function and specify the parallel use of MATLABs
%multistart function
problem =
createOptimProblem('lsqcurvefit','x0',x0,'objective',@pShift_Fe4S4_3_to_1_fitadapted,'lb',lb,'ub',
ub,'xdata',tempdata,'ydata',ydata,'options',opts);
ms = MultiStart('UseParallel',true,'Display','iter'); %Use parallel computation. Set to false to
disable.

%Perform the nonlinear least squares
[xmulti,errormulti,eflag,output,manymins] = run(ms,problem,micro_iter);
fit = xmulti;

%Generate the fitted shifts and rescale the values
shifts = pShift_Fe4S4_3_to_1_fitadapted(xmulti,temprange');
shifts(:,1:6) = shifts(:,1:6)./normalization_factors;

%Renormalize the hyperfine couplings to their true (unscaled) values
fit(4:9) = fit(4:9)./normalization_factors;
fit(12:17) = fit(12:17)./normalization_factors;
fit(18:23) = fit(18:23)./normalization_factors;

%for consistency & comparison, plot the VT-NMR shifts of the homoleptic
%system
tempdata_homo = [298; 288; 278; 267.6; 258; 248; 238; 228; 220];
L_m_homo = [10.025; 10.03; 10.03; 10.035; 10.035; 10.03; 10.03; 10.02; 10.01];
L_p_homo = [4.05; 4.03; 4.01; 3.98; 3.95; 3.92; 3.87; 3.83; 3.78];

%Plot the NMR fits
figure(1);
clf;
hold on;
set(gca, 'FontSize', 12, 'FontName', 'Arial')
set(gca, 'Linewidth', 0.8)
set(gca, 'DefaultLineLinewidth', 0.8)
plot(tempdata,L_p_work,'o','MarkerFaceColor',[0 0 0],'MarkerEdgeColor',[0 0 0],'MarkerSize',3)
plot(tempdata,L_m_work,'s','MarkerFaceColor',[0 0 0],'MarkerEdgeColor',[0 0 0],'MarkerSize',3)
plot(tempdata_homo,L_p_homo,':o','color','k','MarkerFaceColor',[1 1 1],'MarkerEdgeColor',[0 0
0],'MarkerSize',3)
plot(tempdata_homo,L_m_homo,':s','color','k','MarkerFaceColor',[1 1 1],'MarkerEdgeColor',[0 0
0],'MarkerSize',3)
plot(tempdata,H_1_work,'d','MarkerFaceColor',[0 0 0],'MarkerEdgeColor',[0 0 0],'MarkerSize',3)
plot(tempdata,H_2_work,'p','MarkerFaceColor',[0 0 0],'MarkerEdgeColor',[0 0 0],'MarkerSize',3)
plot(tempdata,H_3_work,'^','MarkerFaceColor',[0 0 0],'MarkerEdgeColor',[0 0 0],'MarkerSize',3)
plot(tempdata,H_4_work,'v','MarkerFaceColor',[0 0 0],'MarkerEdgeColor',[0 0 0],'MarkerSize',3)
```

```
plot(temprange',shifts(:,1),'color','m')
plot(temprange',shifts(:,2),'color','m')
plot(temprange',shifts(:,3),'color','m')
plot(temprange',shifts(:,4),'color','m')
plot(temprange',shifts(:,5),'color','m')
plot(temprange',shifts(:,6),'color','m')
xlabel('{\bf Temperature (K)}');
ylabel('{\bf \delta ^1H (ppm)}');
legend('{\itp}-H / {\bf\it3:1}', '{\itm}-H / {\bf\it3:1}', '{\itp}-H / {\ithomoleptic}', '{\itm}-H
/ {\ithomoleptic}', 'H^1', 'H^2', 'H^3', 'H^4', 'fit', 'FontSize', 11, 'Location', 'BestOutSide',
'Box', 'off')
box on;
xlim([210 310]);
xticks([220:10:300]);
ylim([-1 23]);
yticks([0:2:22]);

%Plot the magnetic moment fits
figure(2);
clf;
hold on;
set(gca, 'FontSize', 12, 'FontName', 'Arial')
set(gca, 'Linewidth', 0.8)
set(gca, 'DefaultLineLinewidth', 0.8)
errorbar(tempdata,mag_data_work./10,2.326*0.0138*ones(length(tempdata),1)','LineStyle','none','Col
or','k');
plot(tempdata,mag_data_work./10,'o','MarkerFaceColor',[1 1 1],'MarkerEdgeColor',[0 0
0],'MarkerSize',5)
plot(temprange',shifts(:,7)'./10,'color','m')
xlabel('{\bf Temperature (K)}');
ylabel('{\bf Magnetic moment (\mu_B)}');
xlim([210 310]);
xticks([220:10:300]);
ylim([2.6 3.1]);
yticks([2.65:0.05:3.05]);
box on;
fits(iter,:) = fit; %Store the best fit
residuals(iter,:) = errormulti;
iter = iter + 1; %Increment the iteration variable
toc; %Report the time elapsed
end
outliers = isoutlier(residuals); %Test for outliers based on fit RMSE
outlier_fits = []; %Initialize a matrix to store any outlier fits
for n = flip(1:length(residuals))
if outliers(n)
outlier_fits = [outlier_fits; fits(n,:)]; %Add the outlier fit to the outlier list
fits(n,:) = []; %Remove the outlier fit from the tabulated fit list
end
end

%Generate an output file with all relevant information
t = datetime;
t_string = datestr(t);
fileID = fopen('DmpS3-Im_fit_parameters-26.txt','w');
fprintf(fileID,['Fit performed on: ' t_string '\n']);
fprintf(fileID,'Fit parameters for DmpS3Fe4S4Im*\n');
```

```
fprintf(fileID,'\nFitting Details:\n');
fprintf(fileID,['\nMacro iterations: ' num2str(macro_iter)]);
fprintf(fileID,['\nMicro iterations: ' num2str(micro_iter) '\n']);
fprintf(fileID,'\nInitial conditions:\n');
fprintf(fileID,['\nJ min (valence isomer I): ' num2str(J_1_min) ' cm-1']);
fprintf(fileID,['\nJ max (valence isomer I): ' num2str(J_1_max) ' cm-1']);
fprintf(fileID,['\nJ min (valence isomer II): ' num2str(J_2_min) ' cm-1']);
fprintf(fileID,['\nJ max (valence isomer II): ' num2str(J_2_max) ' cm-1']);
fprintf(fileID,['\nB min: ' num2str(B_1_min) ' cm-1']);
fprintf(fileID,['\nB max: ' num2str(B_1_max) ' cm-1']);
fprintf(fileID,['\nOffset min: ' num2str(offset_min) ' cm-1']);
fprintf(fileID,['\nOffset max: ' num2str(offset_max) ' cm-1']);
fprintf(fileID,['\nPara-DmpS diamagnetic shift: ' num2str(pDmpS_dia) ' ppm']);
fprintf(fileID,['\nMeta-DmpS diamagnetic shift: ' num2str(mDmpS_dia) ' ppm']);
fprintf(fileID,['\n1 Im H diamagnetic shift: ' num2str(Im1_dia) ' ppm']);
fprintf(fileID,['\n2 Im H diamagnetic shift: ' num2str(Im2_dia) ' ppm']);
fprintf(fileID,['\n3 Im H diamagnetic shift: ' num2str(Im3_dia) ' ppm']);
fprintf(fileID,['\n4 Im H diamagnetic shift: ' num2str(Im4_dia) ' ppm\n']);
fprintf(fileID,'\nBest fit magnetic parameters:\n');
fprintf(fileID,['\nJ (valence isomer I): ' num2str(mean(fits(:,1))) ' (' num2str(std(fits(:,1)))
') cm-1']);
fprintf(fileID,['\nJ (valence isomer II): ' num2str(mean(fits(:,2))) ' (' num2str(std(fits(:,2)))
') cm-1']);
fprintf(fileID,['\nJ fraction (global): ' num2str(1./mean(fits(:,24))) ' ('
num2str(std(1./fits(:,24))) ')']);
fprintf(fileID,['\nB: ' num2str(mean(fits(:,3))) ' (' num2str(std(fits(:,3))) ') cm-1']);
fprintf(fileID,['\nOffset: ' num2str(mean(fits(:,10))) ' (' num2str(std(fits(:,10))) ') cm-1\n']);
fprintf(fileID,'\nBest fit hyperfine parameters:\n');
fprintf(fileID,['\npDmpSH (Fe3+): ' num2str(mean(fits(:,4))) ' (' num2str(std(fits(:,4))) ')
MHz']);
fprintf(fileID,['\npDmpSH (Fe2.5+): ' num2str(mean(fits(:,12))) ' (' num2str(std(fits(:,12))) ')
MHz']);
fprintf(fileID,['\nmDmpSH (Fe3+): ' num2str(mean(fits(:,5))) ' (' num2str(std(fits(:,5))) ')
MHz']);
fprintf(fileID,['\nmDmpSH (Fe2.5+): ' num2str(mean(fits(:,13))) ' (' num2str(std(fits(:,13))) ')
MHz']);
fprintf(fileID,['\n1 Im* H (Fe3+): ' num2str(mean(fits(:,6))) ' (' num2str(std(fits(:,6))) ')
MHz']);
fprintf(fileID,['\n1 Im* H (Fe2.5+): ' num2str(mean(fits(:,14))) ' (' num2str(std(fits(:,14))) ')
MHz']);
fprintf(fileID,['\n2 Im* H (Fe3+): ' num2str(mean(fits(:,7))) ' (' num2str(std(fits(:,7))) ')
MHz']);
fprintf(fileID,['\n2 Im* H (Fe2.5+): ' num2str(mean(fits(:,15))) ' (' num2str(std(fits(:,14))) ')
MHz']);
fprintf(fileID,['\n3 Im* H (Fe3+): ' num2str(mean(fits(:,8))) ' (' num2str(std(fits(:,8))) ')
MHz']);
fprintf(fileID,['\n3 Im* H (Fe2.5+): ' num2str(mean(fits(:,16))) ' (' num2str(std(fits(:,16))) ')
MHz']);
fprintf(fileID,['\n4 Im* H (Fe3+): ' num2str(mean(fits(:,9))) ' (' num2str(std(fits(:,9))) ')
MHz']);
fprintf(fileID,['\n4 Im* H (Fe2.5+): ' num2str(mean(fits(:,17))) ' (' num2str(std(fits(:,17))) ')
MHz']);
fprintf(fileID,['\n\nRMSE: ' num2str(mean(residuals)) '\n']);
fprintf(fileID,'\nRaw data:\n');
fprintf(fileID,'\nTemp pDmpS mDmpS H12 H3 H4 Mag Moment\n');
```

```
fprintf(fileID,'\n%.2f %.2f %.2f %.2f %.2f %.2f %.2f %.2f' ,[tempdata'; L_p'; L_m'; H_1'; H_2';
H_3'; H_4'; mag_data']);
fprintf(fileID,'\n\nRaw fit data:\n');
fprintf(fileID,['\nRejected ' num2str(sum(outliers)) ' outlier fits of ' num2str(macro_iter) '
total fits\n']);
fprintf(fileID,'\nJ1 J2 B A1(Fe3+) A2(Fe3+) A3(Fe3+) A4(Fe3+) A5(Fe3+) A6(Fe3+) Offset Beta
A1(Fe2.5+) A2(Fe2.5+) A3(Fe2.5+) A4(Fe2.5+) A5(Fe2.5+) A6(Fe2.5+) Dia_1 Dia_2 Dia_3 Dia_4 Dia_5
Dia_6 1/n');
fprintf(fileID,'\n%.2f %.2f %.2f %.5f %.5f %.5f %.5f %.5f %.5f %.2f %.2f %.5f %.5f %.5f %.5f %.5f
%.5f %.2f %.2f %.2f %.2f %.2f %.2f %.2f' ,fits');
fprintf(fileID,['\n\nOutlier Fits:\n']);
fprintf(fileID,'\nJ1 J2 B A1(Fe3+) A2(Fe3+) A3(Fe3+) A4(Fe3+) A5(Fe3+) A6(Fe3+) Offset Beta
A1(Fe2.5+) A2(Fe2.5+) A3(Fe2.5+) A4(Fe2.5+) A5(Fe2.5+) A6(Fe2.5+) Dia_1 Dia_2 Dia_3 Dia_4 Dia_5
Dia_6 J_frac');
fprintf(fileID,'\n%.2f %.2f %.2f %.5f %.5f %.5f %.5f %.5f %.5f %.2f %.2f %.5f %.5f %.5f %.5f %.5f
%.5f %.2f %.2f %.2f %.2f %.2f %.2f %.2f' ,outlier_fits');

fclose(fileID);
```

## SI References


(1) Grunwald, L.; Clémancey, M.; Klose, D.; Dubois, L.; Gambarelli, S.; Jeschke, G.; Wörle, M.; Blondin, G.; Mougel, V. A complete biomimetic iron-sulfur cubane redox series. *Proceedings of the National Academy of Sciences* **2022**, *119* (31), e2122677119. DOI: 10.1073/pnas.2122677119.

(2) Grunwald, L.; Inoue, M.; Carril, P. C.; Wörle, M.; Mougel, V. Gated electron transfers at synthetic iron-sulfur cubanes. *Chem* **2024**, *10* (1), 365–387. DOI: 10.1016/j.chempr.2023.09.023.

(3) Grunwald, L.; Weber, M. L.; Seng, H.; Clemancey, M.; Wang, H.; Wörle, M.; Yoda, Y.; Cramer, S. P.; Blondin, G.; Mougel, V. Stepwise and Reversible Assembly of 2Fe–2S Rhombs to 8Fe–8S Clusters and Their Topological Interconversions. *ChemRxiv* **2024**, 10.26434/chemrxiv–22024–pnftw. DOI: 10.26434/chemrxiv-2024-pnftw.

(4) Fischer, J. W. A.; Stropp, J.; Tschaggelar, R.; Oberhänsli, O.; Alaniva, N.; Inoue, M.; Mashima, K.; Barnes, A. B.; Jeschke, G.; Klose, D. Design and performance of an oversized-sample 35 GHz EPR resonator with an elevated Q value. *Magn. Reson.* **2024**, *5* (2), 143–152. DOI: 10.5194/mr-5-143-2024.

(5) Stoll, S.; Schweiger, A. An adaptive method for computing resonance fields for continuous-wave EPR spectra. *Chemical Physics Letters* **2003**, *380* (3), 464–470. DOI: 10.1016/j.cplett.2003.09.043.

(6) Carboni, M.; Clémancey, M.; Molton, F.; Pécaut, J.; Lebrun, C.; Dubois, L.; Blondin, G.; Latour, J. M. Biologically Relevant Heterodinuclear Iron–Manganese Complexes. *Inorganic Chemistry* **2012**, *51* (19), 10447–10460. DOI: 10.1021/ic301725z.

(7) Abbott, D.; Grunwald, L.; Singh-Morgan, A. Combined X-ray Absorption and X-ray Emission Spectroscopy Study of Synthetic [$Fe_4S_4$] Cubane Complexes at the S K-edge. In *Combined X-ray Absorption and X-ray Emission Spectroscopy Study of Synthetic [$Fe_4S_4$] Cubane Complexes at the S K-edge*, Facility, E. S. R., Ed.; European Synchrotron Radiation Facility, 2026.

(8) Rovezzi, M.; Harris, A.; Detlefs, B.; Bohdan, T.; Svyazhin, A.; Santambrogio, A.; Degler, D.; Baran, R.; Reynier, B.; Noguera Crespo, P.; et al. TEXS: in-vacuum tender X-ray emission spectrometer with 11 Johansson crystal analyzers. *Journal of Synchrotron Radiation* **2020**, *27* (3), 813–826. DOI: doi:10.1107/S160057752000243X.

(9) Solé, V. A.; Papillon, E.; Cotte, M.; Walter, P.; Susini, J. A multiplatform code for the analysis of energy-dispersive X-ray fluorescence spectra. *Spectrochimica Acta Part B: Atomic Spectroscopy* **2007**, *62* (1), 63–68. DOI: 10.1016/j.sab.2006.12.002.

(10) Grunwald, L.; Abbott, D. F.; Mougel, V. Gauging Iron–Sulfur Cubane Reactivity from Covalency: Trends with Oxidation State. *JACS Au* **2024**, *4* (4), 1315–1322. DOI: 10.1021/jacsau.4c00213.

(11) Guo, Y.; Wang, H.; Xiao, Y.; Vogt, S.; Thauer, R. K.; Shima, S.; Volkers, P. I.; Rauchfuss, T. B.; Pelmenschikov, V.; Case, D. A.; et al. Characterization of the Fe Site in Iron−Sulfur Cluster-Free Hydrogenase (Hmd) and of a Model Compound via Nuclear Resonance Vibrational Spectroscopy (NRVS). *Inorganic Chemistry* **2008**, *47* (10), 3969–3977. DOI: 10.1021/ic701251j.

(12) Kamali, S.; Wang, H.; Mitra, D.; Ogata, H.; Lubitz, W.; Manor, B. C.; Rauchfuss, T. B.; Byrne, D.; Bonnefoy, V.; Jenney Jr, F. E.; et al. Observation of the Fe-CN and Fe-CO Vibrations in the Active Site of [NiFe] Hydrogenase by Nuclear Resonance Vibrational Spectroscopy. *Angewandte Chemie International Edition* **2013**, *52* (2), 724–728. DOI: 10.1002/anie.201204616.

(13) Ogata, H.; Krämer, T.; Wang, H.; Schilter, D.; Pelmenschikov, V.; van Gastel, M.; Neese, F.; Rauchfuss, T. B.; Gee, L. B.; Scott, A. D.; et al. Hydride bridge in [NiFe]-hydrogenase observed by nuclear resonance vibrational spectroscopy. *Nature Communications* **2015**, *6* (1), 7890. DOI: 10.1038/ncomms8890.

(14) Wang, H.; Braun, A.; Cramer, S. P.; Gee, L. B.; Yoda, Y. Nuclear Resonance Vibrational Spectroscopy: A Modern Tool to Pinpoint Site-Specific Cooperative Processes. *Crystals* **2021**, *11* (8), 909. DOI: 10.3390/cryst11080909.

(15) Gee, L. B.; Wang, H.; Cramer, S. P. 9. Nuclear resonance vibrational spectroscopy. In *Bioorganometallic Chemistry*, Weigand, W., Apfel, U.-P. Eds.; De Gruyter, 2020; pp 353–394.

(16) Sturhahn, W. CONUSS and PHOENIX: Evaluation of nuclear resonant scattering data. *Hyperfine Interactions* **2000**, *125* (1), 149–172. DOI: 10.1023/A:1012681503686.

(17) Johnson, D. W.; Spence, J. C. H. Determination of the single-scattering probability distribution from plural-scattering data. *Journal of Physics D: Applied Physics* **1974**, *7* (6), 771. DOI: 10.1088/0022-3727/7/6/304.

(18) Wang, H.; Yoda, Y.; Kamali, S.; Zhou, Z.-H.; Cramer, S. P. Real sample temperature: a critical issue in the experiments of nuclear resonant vibrational spectroscopy on biological samples. *Journal of Synchrotron Radiation* **2012**, *19* (2), 257–263. DOI: 10.1107/S0909049512001380.

(19) Skeel, B. A.; Suess, D. L. M. Exploiting Molecular Symmetry to Quantitatively Map the Excited-State Landscape of Iron–Sulfur Clusters. *Journal of the American Chemical Society* **2023**, *145* (18), 10376–10395. DOI: 10.1021/jacs.3c02412.

(20) Rose, K.; Shadle, S. E.; Glaser, T.; de Vries, S.; Cherepanov, A.; Canters, G. W.; Hedman, B.; Hodgson, K. O.; Solomon, E. I. Investigation of the Electronic Structure of 2Fe−2S Model Complexes and the Rieske Protein Using Ligand K-Edge X-ray Absorption Spectroscopy. *Journal of the American Chemical Society* **1999**, *121* (11), 2353–2363. DOI: 10.1021/ja983455l.

(21) Shadle, S. E.; Hedman, B.; Hodgson, K. O.; Solomon, E. I. Ligand K-edge x-ray absorption spectroscopic studies: metal-ligand covalency in a series of transition metal tetrachlorides. *Journal of the American Chemical Society* **1995**, *117* (8), 2259–2272. DOI: 10.1021/ja00113a015.

(22) Glaser, T.; Rose, K.; Shadle, S. E.; Hedman, B.; Hodgson, K. O.; Solomon, E. I. S K-edge X-ray Absorption Studies of Tetranuclear Iron−Sulfur Clusters: μ-Sulfide Bonding and Its Contribution to Electron Delocalization. *Journal of the American Chemical Society* **2001**, *123* (3), 442–454. DOI: 10.1021/ja002183v.

(23) Bain, A. D. Chemical exchange in NMR. *Progress in Nuclear Magnetic Resonance Spectroscopy* **2003**, *43* (3), 63–103. DOI: 10.1016/j.pnmrs.2003.08.001.

(24) Kleckner, I. R.; Foster, M. P. An introduction to NMR-based approaches for measuring protein dynamics. *Biochimica et Biophysica Acta (BBA) - Proteins and Proteomics* **2011**, *1814* (8), 942–968. DOI: 10.1016/j.bbapap.2010.10.012.

(25) Girerd, J. J.; Papaefthymiou, V.; Surerus, K. K.; Munck, E. Double exchange in iron-sulfur clusters and a proposed spin-dependent transfer mechanism. *Pure and Applied Chemistry* **1989**, *61* (5), 805–816. DOI: doi:10.1351/pac198961050805.

(26) Papaefthymiou, G. C.; Laskowski, E. J.; Frota-Pessoa, S.; Frankel, R. B.; Holm, R. H. Antiferromagnetic exchange interactions in $[Fe_4S_4(SR)_4]^{2-,3-}$ clusters. *Inorganic Chemistry* **1982**, *21* (5), 1723–1728. DOI: 10.1021/ic00135a005.

(27) Noodleman, L. A model for the spin states of high-potential iron-sulfur $[Fe_4S_4]^{3+}$ proteins. *Inorganic Chemistry* **1988**, *27* (20), 3677–3679. DOI: 10.1021/ic00293a051.

(28) We prefer this notation of the Hamiltonian over the ones commonly presented, because it avoids mixing energy terms, into the exchange Hamiltonians.

(29) Crozet, M.; Chaussade, M.; Bardet, M.; Emsley, L.; Lamotte, B.; Mouesca, J.-M. Carbon-13 Solid-State NMR Studies on Synthetic Model Compounds of [4Fe−4S] Clusters in the 2+ State. *The Journal of Physical Chemistry A* **2000**, *104* (44), 9990–10000. DOI: 10.1021/jp002005o.

(30) Papaefthymiou, V.; Girerd, J. J.; Moura, I.; Moura, J. J. G.; Muenck, E. Moessbauer study of D. gigas ferredoxin II and spin-coupling model for $Fe_3S_4$ cluster with valence delocalization. *Journal of the American Chemical Society* **1987**, *109* (15), 4703–4710. DOI: 10.1021/ja00249a037.

(31) Banci, L.; Bertini, I.; Briganti, F.; Luchinat, C.; Scozzafava, A.; Oliver, M. V. Proton NMR spectra of oxidized high-potential iron-sulfur protein (HiPIP) from Rhodocyclus gelatinosus. A model for oxidized HiPIPs. *Inorganic Chemistry* **1991**, *30* (24), 4517–4524. DOI: 10.1021/ic00024a011.

(32) Skeel, B. A.; Suess, D. L. M. Correction to "Exploiting Molecular Symmetry to Quantitatively Map the Excited-State Landscape of Iron–Sulfur Clusters". *Journal of the American Chemical Society* **2024**, *146* (30), 21190–21191. DOI: 10.1021/jacs.4c08423.

(33) Neese, F. The ORCA program system. *WIREs Computational Molecular Science* **2012**, *2* (1), 73–78. DOI: 10.1002/wcms.81.

(34) Neese, F.; Wennmohs, F.; Becker, U.; Riplinger, C. The ORCA quantum chemistry program package. *The Journal of Chemical Physics* **2020**, *152* (22), 224108. DOI: 10.1063/5.0004608.

(35) Tao, J.; Perdew, J. P.; Staroverov, V. N.; Scuseria, G. E. Climbing the Density Functional Ladder: Nonempirical Meta-Generalized Gradient Approximation Designed for Molecules and Solids. *Physical Review Letters* **2003**, *91* (14), 146401. DOI: 10.1103/PhysRevLett.91.146401.

(36) Schäfer, A.; Huber, C.; Ahlrichs, R. Fully optimized contracted Gaussian basis sets of triple zeta valence quality for atoms Li to Kr. *The Journal of Chemical Physics* **1994**, *100* (8), 5829–5835. DOI: 10.1063/1.467146 (acccessed 11/20/2023).

(37) Neese, F. Prediction and interpretation of the $^{57}Fe$ isomer shift in Mössbauer spectra by density functional theory. *Inorganica Chimica Acta* **2002**, *337*, 181–192. DOI: 10.1016/S0020-1693(02)01031-9.

(38) Grimme, S.; Antony, J.; Ehrlich, S.; Krieg, H. A consistent and accurate ab initio parametrization of density functional dispersion correction (DFT-D) for the 94 elements H-Pu. *The Journal of Chemical Physics* **2010**, *132* (15), 154104. DOI: 10.1063/1.3382344.

(39) Grimme, S.; Ehrlich, S.; Goerigk, L. Effect of the damping function in dispersion corrected density functional theory. *Journal of Computational Chemistry* **2011**, *32* (7), 1456–1465. DOI: 10.1002/jcc.21759.

(40) Löwdin, P. O. On the Non-Orthogonality Problem Connected with the Use of Atomic Wave Functions in the Theory of Molecules and Crystals. *The Journal of Chemical Physics* **2004**, *18* (3), 365–375. DOI: 10.1063/1.1747632.

(41) Barone, V.; Cossi, M. Quantum Calculation of Molecular Energies and Energy Gradients in Solution by a Conductor Solvent Model. *The Journal of Physical Chemistry A* **1998**, *102* (11), 1995–2001. DOI: 10.1021/jp9716997.

(42) Noodleman, L.; Case, D. A.; Aizman, A. Broken symmetry analysis of spin coupling in iron-sulfur clusters. *Journal of the American Chemical Society* **1988**, *110* (4), 1001–1005. DOI: 10.1021/ja00212a003.

(43) Bergeler, M.; Stiebritz, M. T.; Reiher, M. Structure–Property Relationships of $Fe_4S_4$ Clusters. *ChemPlusChem* **2013**, *78* (9), 1082–1098. DOI: 10.1002/cplu.201300186.

(44) Römelt, M.; Ye, S.; Neese, F. Calibration of Modern Density Functional Theory Methods for the Prediction of $^{57}$Fe Mössbauer Isomer Shifts: Meta-GGA and Double-Hybrid Functionals. *Inorganic Chemistry* **2009**, *48* (3), 784–785. DOI: 10.1021/ic801535v.

(45) Proppe, J.; Reiher, M. Reliable Estimation of Prediction Uncertainty for Physicochemical Property Models. *Journal of Chemical Theory and Computation* **2017**, *13* (7), 3297–3317. DOI: 10.1021/acs.jctc.7b00235.

(46) Frisch, M. J.; Trucks, G. W.; Schlegel, H. B.; Scuseria, G. E.; Robb, M. A.; Cheeseman, J. R.; Scalmani, G.; Barone, V.; Petersson, G. A.; Nakatsuji, H.; et al. Gaussian 16 Rev C.01. *Gaussian 16, Revision C.01, Gaussian Inc., Wallingford CT, 2019*.

(47) Jaguar. *version 11.0, Schrodinger, Inc., New York, NY, 2020*.

(48) Noodleman, L. Valence Bond Description of Anti-Ferromagnetic Coupling in Transition-Metal Dimers. *J. Chem. Phys.* **1981**, *74* (10), 5737–5743. DOI: Doi 10.1063/1.440939.

(49) Noodleman, L.; Case, D. A. Density-Functional Theory of Spin Polarization and Spin Coupling in Iron-Sulfur Clusters. *Adv. Inorg. Chem.* **1992**, *38*, 423–470.

(50) Becke, A. D. Density-Functional Exchange-Energy Approximation with Correct Asymptotic-Behavior. *Phys. Rev. A* **1988**, *38* (6), 3098–3100. DOI: Doi 10.1103/Physreva.38.3098.

(51) Perdew, J. P. Density-Functional Approximation for the Correlation-Energy of the Inhomogeneous Electron-Gas. *Phys Rev B* **1986**, *33* (12), 8822–8824. DOI: Doi 10.1103/Physrevb.33.8822.

(52) Harris, T. V.; Szilagyi, R. K. Iron–sulfur bond covalency from electronic structure calculations for classical iron–sulfur clusters. *Journal of Computational Chemistry* **2014**, *35* (7), 540–552. DOI: 10.1002/jcc.23518.

(53) Tomasi, J.; Mennucci, B.; Cammi, R. Quantum mechanical continuum solvation models. *Chemical Reviews* **2005**, *105* (8), 2999–3093.

(54) Pelmenschikov, V.; Birrell, J. A.; Pham, C. C.; Mishra, N.; Wang, H.; Sommer, C.; Reijerse, E.; Richers, C. P.; Tamasaku, K.; Yoda, Y.; et al. Reaction Coordinate Leading to H2 Production in [FeFe]-Hydrogenase Identified by Nuclear Resonance Vibrational Spectroscopy and Density Functional Theory. *J. Am. Chem. Soc.* **2017**, *139* (46), 16894–16902. DOI: 10.1021/jacs.7b09751.

(55) Pelmenschikov, V.; Gee, L. B.; Wang, H.; MacLeod, K. C.; McWilliams, S. F.; Skubi, K. L.; Cramer, S. P.; Holland, P. L. High-Frequency Fe–H Vibrations in a Bridging Hydride Complex Characterized by NRVS and DFT. *Angew. Chem. Int. Ed.* **2018**, *130* (30), 9511–9515. DOI: doi:10.1002/ange.201804601.

(56) Pelmenschikov, V.; Guo, Y.; Wang, H.; Cramer, S. P.; Case, D. A. Fe–H/D stretching and bending modes in nuclear resonant vibrational, Raman and infrared spectroscopies: Comparisons of density functional theory and experiment. *Faraday Discuss.* **2011**, *148*, 409–420. DOI: 10.1039/c004367m.
